# Joint Effects of Wildfire Smoke and Extreme Heat on Hospitalizations in California, 2011–2020

**DOI:** 10.1029/2024GH001237

**Published:** 2025-06-06

**Authors:** Caitlin G. Jones‐Ngo, Rebecca J. Schmidt, Erwan Monier, Sara Ludwick, Mohammad Z. Al‐Hamdan, Jason Vargo, Kathryn C. Conlon

**Affiliations:** ^1^ Department of Public Health Sciences University of California Davis CA USA; ^2^ Scripps Institution of Oceanography University of California San Diego CA USA; ^3^ Department of Land Air and Water Resources University of California Davis CA USA; ^4^ Department of Environmental Science and Policy University of California Davis CA USA; ^5^ National Center for Computational Hydroscience and Engineering (NCCHE) School of Engineering University of Mississippi Oxford MS USA; ^6^ Department of Civil Engineering School of Engineering University of Mississippi Oxford MS USA; ^7^ Independent Researcher San Francisco CA USA

**Keywords:** joint effects, wildfire smoke, extreme heat, compound events, climate hazards

## Abstract

Wildfire smoke and extreme heat events are worsening in California, but their combined health effects are not well understood. This study estimates joint effects of extreme heat and wildfire smoke on hospitalizations in California, 2011–2020. We used a case crossover design with time‐stratified controls and conditional logistic regression to estimate these effects at multiplicative and additive scales. Exposures were assessed for 16 combinations of exposure lags (0–3 days) for extreme heat and wildfire influenced fine particulate matter. Among over 28 million cases of all‐natural cause morbidity, the majority were adults aged 65 and older (41.4%), English speakers (85.1%), and White, non‐Hispanic (49.7%), mostly residing in urban areas (97.2%). The study found roughly 8% of respiratory morbidities (95% CI, 2.4%–13.8%) were attributable to the interaction of wildfire smoke and extreme heat. Significant joint effects were also observed for cardiovascular (5.5%) and renal morbidities (6.2%). Subgroup analyses revealed stronger effects: Respiratory (19.2%, 95% CI 6.5%–32.1%) and cerebrovascular morbidities (15.7%, 95% CI 4%–27.4%) were most pronounced in Black individuals; older adults (50–64 years) showed strong effects for renal morbidities (15.4%, 95% CI −1.6%−32.6%); and cardiovascular effects were highest among females (9.8%, 95% CI 2.9%–16.7%). Effects on all‐natural cause morbidity were generally null. The interaction of wildfire smoke and extreme heat within a short exposure window (4 days) increases hospitalizations; highlighting the need for joint heat and wildfire smoke interventions that target populations at greater risk.

## Key Points

- Compound wildfire smoke and extreme heat is associated with synergistic impacts on morbidity in California from 2011 to 2019
- Effects were more pronounced within population subgroups characterized by individual and community level sociodemographic factors
- Synergistic effects were observed for exposures to co‐occurring days and short‐term windows (4 days) of wildfire smoke and extreme heat

## 1 Introduction

California has incurred repeated record‐breaking seasons of wildfire and extreme heat in recent years. Consequences of climate change have led to increasing frequencies and magnitudes for these climate hazards. Since the 1970s, the state has seen average summer day temperatures increase by approximately 1.4°C amidst a fivefold increase in landscape burned by wildfire (Westerling et al., 2006; Williams et al., 2019). Longer and more severe wildfire seasons are driving increasing compound wildfire and extreme heat events, that is, the combination of multiple hazards contributing to environmental or public health risks (Masri et al., 2022; Rosenthal et al., 2022). However, the resulting health impacts of compound wildfire smoke and extreme heat exposures are not well understood.

Adverse health impacts of wildfire emissions and extreme heat events have been studied extensively in single hazard assessments. Associations with wildfire smoke have been studied in relationship to multiple air pollutants, such as ozone, NO, and particulate matter, but the primary public health concern has focused on fine particulate matter (PM_2.5_) (Cascio, 2018; Reid, Brauer, et al., 2016; U.S. EPA, 2019). Exposure to PM_2.5_ due to wildfires increases risks of adverse respiratory and cardiovascular outcomes, including asthma, chronic obstructive pulmonary disease (COPD), and cardiac arrest (Cascio, 2018; Chen et al., 2021; Reid, Brauer, et al., 2016). Similarly, studies of extreme heat show high ambient temperatures increase risks of acute health conditions, such as heat stroke, renal failure, and fluid and electrolyte disorders (Bi et al., 2011; Bobb et al., 2014; Guirguis et al., 2014), as well as exacerbation of underlying illnesses, including respiratory and cardiovascular conditions (Schmeltz et al., 2016); although a variety of definitions are often used to characterize extreme heat (Bi et al., 2011; Bobb et al., 2014; Guirguis et al., 2014; Schmeltz et al., 2016). We focus on the associated effects of single days of extreme temperatures, rather than consecutive days, that is, heat waves, to examine different combinations of wildfire smoke and extreme heat exposures using the same temporal unit.

Recent studies show joint effects of air pollution and heat, including ambient air pollutants and limited evidence on wildfire emissions (Analitis et al., 2018; Anenberg et al., 2020; Chen et al., 2024; Patel et al., 2019; Rahman et al., 2022; Schwarz et al., 2021). A recent review by Anenberg et al. (2020) on synergistic joint health effects of ambient air pollution, temperature, and pollen exposure found sufficient evidence for synergistic all‐cause mortality, cardiovascular, and respiratory effects of air pollution and heat (particularly for ozone and particulate matter) (Anenberg et al., 2020). Another study on the burden of compound heat and air pollution found peaks in air pollution exposures were linked to wildfire events and high heat index (Austin et al., 2021). Similarly, other studies also suggest wildfires are key to driving exposures and impacts from compound PM_2.5_ and extreme heat (Masri et al., 2022; Patel et al., 2019; Rosenthal et al., 2022). The few studies on joint effects of wildfire smoke and heat show effects for emergency department admissions in Australia (Patel et al., 2019), and for hospitalizations (Chen et al., 2024) and all‐cause, respiratory, and cardiovascular mortalities in California (Rahman et al., 2022). Our study extends this research by investigating additional outcomes related to independent effects of wildfire smoke and extreme heat that have yet to be explored in the compound hazards context, including renal and cerebrovascular morbidity. Moreover, prior studies have primarily focused on the effects of exposure to wildfire smoke and extreme heat on the same day, and this work takes a novel approach by examining various exposure lag patterns to better elucidate the temporal dynamics and potential synergistic effects of these hazards over short‐term periods.

Findings from previous epidemiologic studies of ambient air pollutants and extreme heat also indicate joint health effects can be variable across specific social and place‐based vulnerabilities (Qin et al., 2017; Schwarz et al., 2021; Simpson et al., 2023). Schwarz et al. (2021) did not observe additive interaction between ozone and heat exposures in the general population, but these joint effects were strong in subpopulation groups such as census tracts with lower median income or higher unemployment. Similarly, Chen et al. (2024) show the synergistics joint effects of exposure to wildfire smoke and extreme heat on the same day varies spatially. They found larger effects in communities with greater socioeconomic and place‐based disadvantages, for example, lower income, lower education, and reduced green space. We aim to investigate the joint effects of exposure to compound wildfire smoke and extreme heat on hospitalizations in California from 2011 to 2020 and characterize populations with greater risks of adverse impacts. We examine multiple relationships of wildfire smoke and extreme heat exposures within a short‐term exposure window. This study seeks to provide a better understanding of the increasing threats of wildfire and extreme heat events to improve public health adaptations amidst a changing climate.

## 2 Materials and Methods

### 2.1 Population and Health Data

We obtained hospital admissions data for California, 2011–2020, from the California Department of Health Care Access and Information (HCAI). International Classification of Disease, Ninth Edition (ICD‐9) and Tenth Edition (ICD‐10) were used to classify outcomes of interest. Diagnoses were included for adults age ≥18 years and excluded accidental conditions due to trauma, injury, or poison. Diagnoses meeting these criteria (e.g., circulatory diseases, respiratory diseases, infectious diseases, metabolic disorders, cancer, mental and neurodevelopmental disorders, and complications of pregnancy or childbirth) were classified as all‐natural cause morbidity. Cause‐specific morbidities were also defined for respiratory‐cause morbidity (ICD‐9 codes 460–466, 471, 472, 477, 478, 480–487, 490–496, 511, 513–519, 786; ICD‐10 codes J00‐J06, J12‐J18, J20‐J22, J30, J31, J33, J34, J38, J39, J40‐J47, J80‐J86, J90‐J92, J94, J96‐J99, R04‐R07, R09), cardiovascular‐cause morbidity (ICD‐9 codes 390–398, 401–417, 420–429, 440–449; ICD‐10 codes I00‐I02, I05‐I16, I20‐I28, I30‐I5A, I70‐I79), cerebrovascular‐cause morbidity (ICD‐9 codes 430–438, ICD‐10 codes I60‐I69), and renal‐cause morbidity (ICD‐9 codes 580–589, 591–599, 788; ICD‐10 codes N00‐N08, N10‐N13, N15‐N23, N25‐N29, R30‐R39).

HCAI also provides individual level data for sex, age, preferred language spoken, and race and ethnicity. We characterized preferred language as English, Spanish or other. Additionally, race and ethnic groups were collapsed due to a small number of cases; therefore, race and ethnicity were defined as White, Hispanic, Black, Asian, and other. Other race includes individuals identifying as Native Hawaiian or Pacific Islander, American Indian or Alaska Native, multiracial, or other in the HCAI reports. We also obtained geographic and temporal data for date of admission, hospital ZIP Code, and patient's residential ZIP Code.

ZIP Code Tabulation Area (ZCTA) level characteristics for social and place‐based disadvantages were obtained from US Census 5‐year American Community Survey, 2015–2019. We acquired measures of poverty, educational attainment, and rurality to examine community level factors relating to exposure and outcome. Community level factors were operationalized as binary variables for stratified analyses. We characterized higher risk populations for educational attainment as ZCTAs where 50% or more of the adult population has a high school education or less; for poverty as ZCTAs where 25% or more of households are living under poverty; and for rural as ZCTAs with majority (50% or more) rural composition. Additionally, we calculated ZCTA‐level, population‐weighted exposure estimates (see Section 2.3) using 1‐km gridded population data from the Gridded Population of the World V4 (Doxsey‐Whitfield et al., 2015).

### 2.2 Environmental Data

Daily 4‐km gridded maximum temperature (*T*_max_) was obtained from the gridMET data set for our study period and region (Abatzoglou, 2013). This data set combines data from the Parameter‐elevation Regressions on Independent Slopes Model (PRISM) and interpolated data from the NASA North American Land Data Assimilation System (NLDAS) Phase 2.

Wildfire smoke and air quality data are a combination of geostatistical modeled estimates of daily PM_2.5_ and wildfire‐specific smoke products (Jones‐Ngo et al., 2024). Modeled daily PM_2.5_ estimates, 3‐km spatial resolution gridded over the state of California, is from the NASA Health and Air Quality Applied Sciences Team (HAQAST) (Al‐Hamdan et al. (2019, 2014, 2009); Freedman et al. (2021, 2017); Diao et al. (2019); O’Neill et al. (2021)). This product uses a geostatistical surfacing algorithm combining ground monitored data with satellite information on aerosol optical depth as described in detail in Al‐Hamdan et al. (2019, 2014, 2009). Daily gridded PM_2.5_ estimates were then combined with smoke plume data from NOAA's Hazard Mapping System (HMS) SMOKE Product to estimate the influence of wildfires on PM_2.5_, as described in Jones‐Ngo et al. (2024). Expected smoke‐free estimates of PM_2.5_ were modeled for each grid and day of year based on estimates for the same day of year from 2011 to 2020 without HMS smoke plume present. The HMS data includes spatial vectors of smoke plumes from satellite imagery, thus, if a smoke plume overlapped a grid cell and total PM_2.5_ exceeded the expected smoke‐free value, then WF‐Influenced PM_2.5_ estimates were calculated by subtracting the expected smoke‐free value from the total PM_2.5_ estimate.

### 2.3 Exposure Definitions

We classified exposures at the ZCTA level for wildfire smoke and extreme heat. Gridded estimates were resampled to 1‐km and combined with 1‐km population data. Then, the product of hazard estimates, *T*_max_ and WF‐Influenced PM_2.5_, and proportion of ZCTA population within the grid cell were aggregated to the ZCTA‐level. ZCTA‐level estimates reflect exposure across the population distribution within the ZCTA.

For the primary analysis, we selected definitions of extreme heat days using the 95th percentile threshold, as detailed below, (referred to as extreme heat hereafter) and wildfire smoke as continuous WF‐Influenced PM_2.5_ per 1 μg/m^3^ (referred to as wildfire smoke hereafter). We also tested the sensitivity of different exposure definitions for wildfire smoke and extreme heat. Daily *T*_max_ estimates were dichotomized to define single extreme heat days using month‐ and ZCTA‐specific thresholds at the 90th, 95th, and 99th percentiles. We tested continuous daily *T*_max_ and health effects per 5°F increments of *T*_max_. For wildfire smoke, we tested cutoffs using WF‐Influenced PM_2.5_ greater than 0 and greater than 12 μg/m^3^, which corresponds to the cutoff for concentrations of heavy density HMS smoke plumes, shown with the WF‐Influenced PM_2.5_ metric. Heavy density smoke plumes have been shown to elicit stronger associations with adverse health outcomes (Jones et al., 2020).

### 2.4 Statistical Analysis

We examined the relationship between wildfire smoke and extreme heat using a time‐stratified case crossover design. This approach is commonly used for epidemiologic studies of air pollution and extreme heat as it controls for individual level factors, such as demographics and comorbidities, and temporal variation, such as day of week effects and seasonality (Bateson & Schwartz, 2001; Carracedo‐Martínez et al., 2010; Janes et al., 2005). The date of hospital admission was considered the case day, and control days were matched by day of week in the same month and year.

Each case and control were assigned daily, ZCTA‐level exposure estimates for lag days 0–3. Exposure on lag day 0 is the case or control day, where exposure on lag day 1 is 1 day prior to the case or control day, lag day 2 is 2 days prior, and lag day 3 is 3 days prior. Exposure was assigned using patient's ZIP Code of residence matched to ZCTA. When patient ZIP Code was unmatched (*N* = 964 case and controls), we used hospital ZIP Code instead. Additionally, two ZCTAs were missing exposure data and excluded from the analysis (*N* = 1,745 cases and controls).

We estimated the joint effects of WF‐Influenced PM_2.5_ and extreme heat days on all‐natural, respiratory, cardiovascular, cerebrovascular, and renal morbidity risks using conditional logistic regression. Joint effects studies have been used extensively in environmental epidemiology (Analitis et al., 2018; Anenberg et al., 2020; Chen et al., 2024; Davalos et al., 2017; Patel et al., 2019; Rahman et al., 2022), and additive scale interaction is recognized as a more relevant measure for public health (Rothman et al., 1980; VanderWeele & Knol, 2014). Additive measures provide insights into the absolute difference in hospitalizations due to the interaction of wildfire smoke and extreme heat, while multiplicative measures indicate how much more likely someone is to be hospitalized when exposed to both hazards. Therefore, we present primary results on the additive scale. The conditional logistic regression models provide results on the multiplicative scale for interaction (${\text{OR}}_{11}$), as well as independent effects of wildfire smoke (${\text{OR}}_{10}$) and extreme heat (${\text{OR}}_{01}$). We then calculated additive scale interaction *post hoc* by measuring the attributable proportion due to interaction (AP; Equation 1), calculated based on the relative excess risk due to interaction (RERI; Equation 2). This approach provides empirical evidence for the number of hospital admissions attributable to the interaction of wildfire smoke and extreme heat effects.

(1) $$\text{AP}=\frac{\text{RERI}}{{\text{OR}}_{11}-1}$$

(2) $${\text{RERI}=\text{OR}}_{11}-\left({\text{OR}}_{10}+{\text{OR}}_{01}-1\right)$$

We modeled each outcome and combination of wildfire smoke and extreme heat exposure lag days separately to explore how these two exposures interact over time. Given the complex and potentially delayed physiological responses, as well as social and behavioral factors (e.g., access to care or willingness to seek care), the effects of these exposures may vary depending on the timing of exposure relative to the onset of illness. By modeling each combination of exposure lags, we capture potential synergistic effects that may differ based on when the exposures occur in relation to each other, allowing for a more granular understanding of the interaction. This approach, while exploratory, provides insights to the nuanced interactions between these two hazards that could be missed with more aggregate modeling strategies. Combinations of exposure lags for compound extreme heat and wildfire smoke exposure, CHWF lag_X,Y_, are described using “X,Y” notation representing heat lag day *X* and smoke lag day Y. Additionally, we conducted stratified models for population subgroups of individual level factors—sex, age group, preferred language spoken, and race and ethnicity—and community factors at the ZCTA level—household income below poverty, low educational attainment, and rurality.

The model used the following conditional logistic regression formulation:

(3) $$\log\left(\frac{\mathrm{Pr}\left(Y=1|X\right)}{1-\mathrm{Pr}\left(Y=1|X\right)}\right)=\beta_{0i}+\beta_{H}X_{\text{HEAT}}+\beta_{S}X_{\text{SMOKE}}+\beta_{\text{HS}}\left(X_{\text{HEAT}}*X_{\text{SMOKE}}\right)$$

Where:

- *Y* is the binary outcome of interest (e.g., hospitalization for a specific condition).
- *X*_HEAT_ is a binary indicator for heat exposure, specific to the corresponding lag day.
- *X*_SMOKE_ is the continuous measure of wildfire smoke exposure, also specific to the corresponding lag day.
- *X*_HEAT_ * *X*_SMOKE_ represents the interaction term between heat and smoke exposures, allowing for the evaluation of joint effects of both exposures.
- *β*_0*i*_ is the intercept term, representing the case crossover strata.
- *Β*_H_, *β*_S_, and *β*_HS_ are the coefficients for heat exposure, smoke exposure, and the interaction term, respectively.

The novel coronavirus (COVID‐19) pandemic had significant effects in California in 2020. The state issued a mandatory stay at home order starting March 2020, increasing the proportion of population staying at home (Dave et al., 2021; Zanocco et al., 2021). Subsequently, the Pandemic changed hospital utilization during this period (Bhatt et al., 2020; Ojetti et al., 2020). Thus, we excluded 2020 from the analysis; however, we conducted sensitivity analyses for data from 2011 to 2020 and 2020, separately. Additionally, we further tested models to examine the change in ICD classifications (from ICD‐9 to ICD‐10) for each outcome of interest. Renal diagnoses changed meaningfully in annual analyses of admissions data, from 2015 to 2016. Consequently, when ICD coding systems changed, diagnoses of renal conditions changed as well, as described by another study (Watzlaf et al., 2007). Therefore, we tested the sensitivity of models for cause‐specific end points for 2011 to 2015 and 2016 to 2019, separately. Models were also restricted to events during May through November, when compound wildfire smoke and extreme heat exposures primarily occurred, and additional sensitivity analyses were conducted examining all months.

All analyses were performed using *R* statistical software (version 4.3.1). The InteractionR package was used to get estimates for multiplicative and additive scale interaction terms, using the Delta method to estimate CIs. This research was reviewed and approved by the California Health and Human Services Agency's Committee for the Protection of Human Subjects (CPHS) (#2022‐130) and the University of California, Davis IRB Administration (#1943974‐1).

## 3 Results

In California from 2011 to 2019 May through November, there were over 15 million hospital admissions for all‐natural cause morbidity (Table 1). There were slight differences in the demographics among the outcomes of interest. Notably, cases of cause‐specific end points were older with higher proportions of elderly aged 65 years and older compared to all‐natural cause cases. Other demographic factors were comparable across outcomes with a sample predominantly composed of persons identifying as White and English speakers. Exposures were similar among all outcomes of interest. The highest *T*_max_ exposures among all cases occurred in 2016 (123.1°F), while the maximum WF‐Influenced PM_2.5_ exposure was in 2017 (309.4 μg/m^3^).

**Table 1:** Demographics and Exposure by Outcome of Interest, Including Individual Level Case Demographics and ZIP Code Tabulation Area (ZCTA) Characteristics, for California From 2011 to 2019, May Through November

	All‐natural	Cardiovascular	Cerebrovascular	Renal	Respiratory
Case Counts	15,014,852	33,277	47,322	18,930	24,453
Sex					
Male (%)	42.4	56.2	50.5	44.1	46.8
Female (%)	57.6	43.8	49.5	55.9	53.2
Age Group					
18–49 years (%)	36.0	11.3	9.7	19.3	16.6
50–64 years (%)	23.8	27.5	26.0	23.4	29.7
65+ yr (%)	40.2	61.2	64.3	57.2	53.7
Preferred Language					
English (%)	85.2	84.4	82.7	82.8	84.6
Spanish (%)	10.5	10.0	10.9	12.1	9.9
Other (%)	4.3	5.5	6.4	5.1	5.4
Race and Ethnicity					
White (%)	49.8	54.2	51.3	52.4	52.2
Black (%)	9.2	10.6	9.6	9.1	12.4
Hispanic (%)	27.7	21.9	22.7	26.5	22.7
Asian (%)	8.7	8.6	11.4	8.2	8.4
American Indian, Alaska Native (%)	0.3	0.3	0.3	0.3	0.3
Hawaiian Native, Pacific Islander (%)	3.0	3.2	3.2	2.8	3.0
Multiracial (%)	0.1	0.1	0.1	0.0	0.1
Other (%)	0.4	0.4	0.5	0.3	0.4
Rurality					
Majority urban (%)	97.2	96.8	97.0	97.4	96.9
Majority rural (%)	2.8	3.2	3.0	2.6	3.1
Poverty					
Lesser poverty (%)	81.2	81.8	82.6	80.9	79.6
Higher poverty (%)	18.8	18.2	17.4	19.1	20.4
Educational Attainment					
Higher education (%)	68.1	68.0	69.3	67.6	65.4
Lower education (%)	31.8	31.8	30.6	32.3	34.5
Exposure					
Mean, Max temperature, °F	75.5	75.3	75.3	76.5	74.5
No. cases heat exposed	12,50,476	2861	3,857	1,623	2,216
% cases heat exposed (%)	8.3	8.6	8.2	8.6	9.1
Mean, Smoke PM2.5, μg/m^3^	0.086	0.082	0.091	0.078	0.073
No. cases smoke exposed	558,972	1,218	1,835	566	756
% cases smoke exposed (%)	3.7	3.7	3.9	3.0	3.1

**Figure 1:**
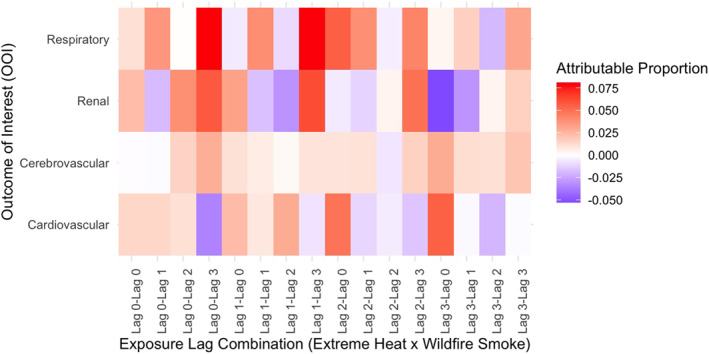
Heat map of attributable proportion of hospital admissions due to the interaction of extreme heat and wildfire smoke by outcome of interest in California, May through November 2011–2019. Different combinations of exposure lags were modeled separately.

Our results show the joint effects of compound wildfire smoke and extreme heat differ for all‐natural, respiratory‐, cardiovascular‐, cerebrovascular‐, and renal‐cause morbidities (Figure 1). Effects differed by combination of exposure lag days and outcomes of interest. Our primary analysis of extreme heat and wildfire smoke shows strong multiplicative and additive interaction for respiratory, cardiovascular, and renal morbidities, though inconsistent for all exposure lag combinations. The largest additive effect shows around 8.1% of respiratory morbidities (CHWF lag_0,3_) is due to the interaction of wildfire smoke and extreme heat effects (95% CI 2.3%–13.8%, *p* = 0.003; Table S1 in Supporting Information S1). Cardiovascular and renal morbidities also showed significant and near‐significant joint effects, respectively. However, we did not observe joint effects for cerebrovascular morbidity in the total case population.

Effect estimates for all‐natural cause morbidity were small and close to null across all analyses. In the primary analysis, significant additive effects (attributable proportion) ranged from −0.5% to 0.46% across the different exposure lag combinations. While many of these results show statistically significant p‐values, this is likely due to the large sample size; the effect sizes are too small to have practical significance for public health practice (Khalilzadeh & Tasci, 2017). The lack of meaningful effects suggests that the combined effects of wildfire smoke and extreme heat do not substantially contribute to morbidity in this broad category. Results for all‐natural cause morbidity are presented in supplementary tables, including tables of additive effects (Table S1 in Supporting Information S1) and multiplicative effects (Table S2 in Supporting Information S1).

### 3.1 Stratified Analyses

**Figure 2:**
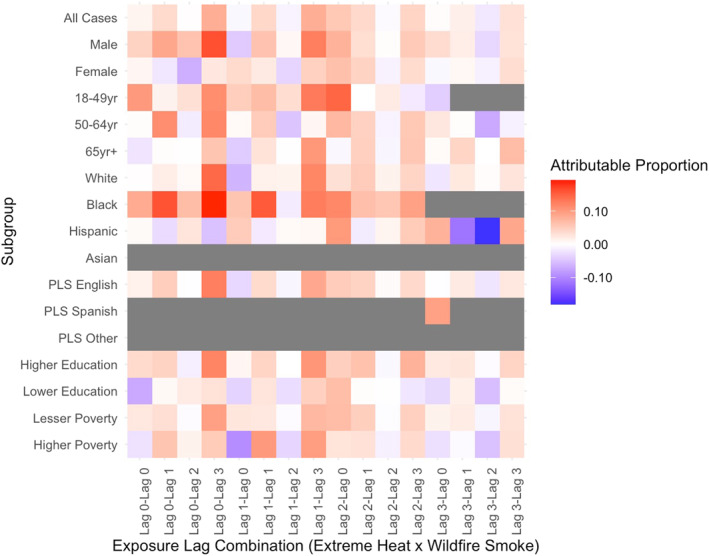
Heat map of attributable proportion of respiratory morbidity due to the interaction of extreme heat and wildfire smoke by outcome of interest in California, May through November 2011–2019. Models were stratified by individual and community level characteristics. Different combinations of exposure lags were modeled separately. Preferred language spoken is abbreviated as PLS. Estimates with less than 10 cases exposed were suppressed, shown in gray.

**Figure 3:**
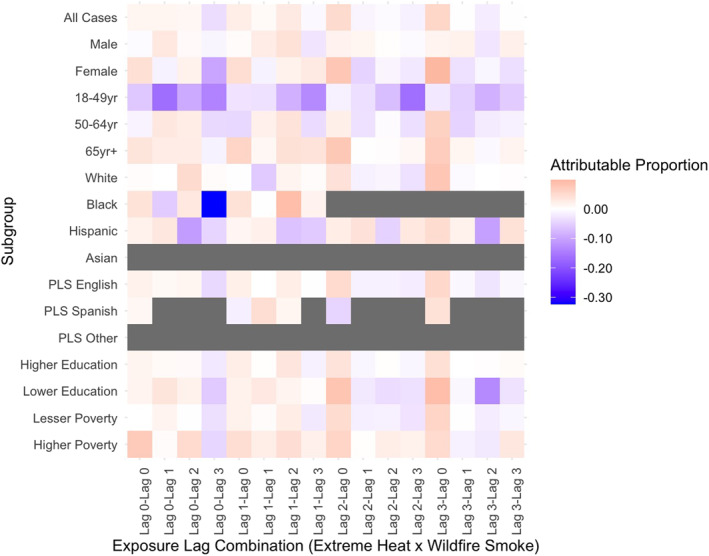
Heat map of attributable proportion of cardiovascular morbidity due to the interaction of extreme heat and wildfire smoke by outcome of interest in California, May through November 2011–2019. Models were stratified by individual and community level characteristics. Different combinations of exposure lags were modeled separately. Preferred language spoken is abbreviated as PLS. Estimates with less than 10 cases exposed were suppressed, shown in gray.

**Figure 4:**
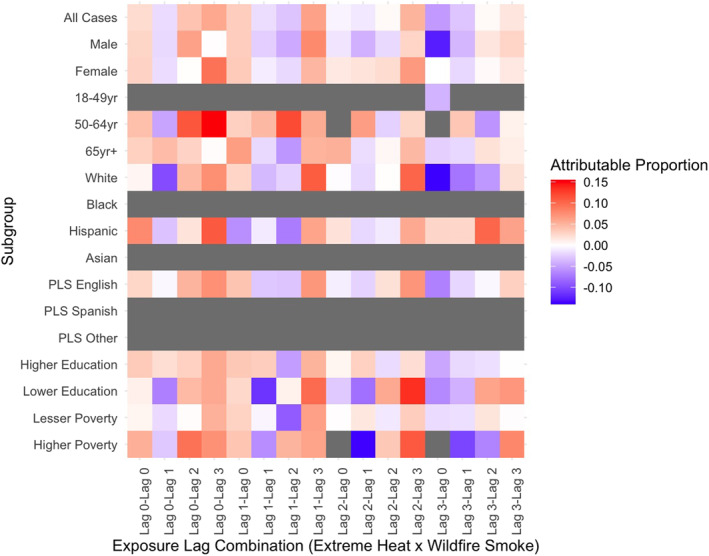
Heat map of attributable proportion of renal morbidity due to the interaction of extreme heat and wildfire smoke by outcome of interest in California, May through November 2011–2019. Models were stratified by individual and community level characteristics. Different combinations of exposure lags were modeled separately. Preferred language spoken is abbreviated as PLS. Estimates with less than 10 cases exposed were suppressed, shown in gray.

**Figure 5:**
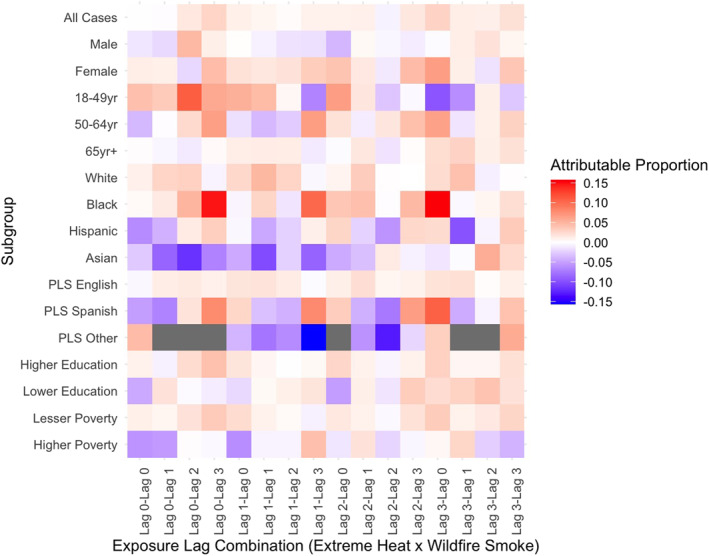
Heat map of attributable proportion of cerebrovascular morbidity due to the interaction of extreme heat and wildfire smoke by outcome of interest in California, May through November 2011–2019. Models were stratified by individual and community level characteristics. Different combinations of exposure lags were modeled separately. Preferred language spoken is abbreviated as PLS. Estimates with less than 10 cases exposed were suppressed, shown in gray.

Stratified results revealed that subpopulations experienced significant joint effects of wildfire smoke and extreme heat at both additive (Table S3 in Supporting Information S1) and multiplicative (Table S4 in Supporting Information S1) scales. The outcomes and exposure lags varied across stratum‐specific risks, and not all effects persisted at the multiplicative scale. Notable differences in effects were observed between individual and community‐level factors, health outcomes, and exposure lags. For example, the attributable proportion of morbidity due to the interaction of wildfire smoke and extreme heat was larger and statistically significant in certain subgroups compared to the total case population for respiratory (Figure 2), cardiovascular (Figure 3), and renal (Figure 4) morbidities. Additionally, significant joint effects for cerebrovascular morbidities (Figure 5) were observed in some strata, which were not present in the total case population. These results, stratified by individual and community factors, are discussed in greater detail in the following sections.

In some instances, the case population was predominantly composed of a specific characteristic (e.g., preferred language spoken or rural/urban ZCTA classification), which limits the results in certain subgroups. Subgroups with fewer than 10 cases exposed were suppressed.

#### 3.1.1 Sex

Sex‐stratified estimates showed males and females had significant joint effects for wildfire smoke and extreme heat that differed by health outcome and exposure lags. The strongest sex‐stratified additive effects showed roughly 16% of respiratory morbidities in males exposed to heat on the day of hospital admission (lag 0) and wildfire smoke 3 days prior (lag 3) were attributable to interaction (95% CI 7.7%–24.6%, *p* < 0.001; Table S3 in Supporting Information S1). This effect was significantly larger in males (16.2%) compared to females (2.3%). In contrast, additive effects for cardiovascular, cerebrovascular, and renal morbidity were larger and significant among females. Synergistic effects were significantly higher for females compared to males for cerebrovascular morbidity (6.6% for females compared to non‐significant −0.2% for males on CHWF lag_3,0_) and differences were near‐significant for cardiovascular morbidity (9.8% for females compared to non‐significant 1.5% for males on CHWF lag_3,0_).

#### 3.1.2 Age Group

There were differences in joint effects across age groups, but some relationships varied by exposure lags. For instance, additive effects for respiratory morbidity were largest for young to middle‐aged adults, 18–49 years of age (AP 14.6%, 95% CI 3.3%–25.9%, *p* = 0.0056; Table S3 in Supporting Information S1) on CHWF lag_2,0_. This effect was significantly higher compared to the oldest group, age 65 years or older, with the same exposure lags (−0.7%, non‐significant). However, individuals aged 65 years or older did show significant joint effects for respiratory morbidity on CHWF lag_1,3_ (AP 10.3%, 95% CI 2.3%–18.3%, *p* = 0.006) that was not significantly less than the effect for 18–49 years of age group (AP 12.9%, 95% CI −1−27%, *p* = 0.035) and much higher than the 50–64 year old age group (AP 1%, non‐significant). On CHWF lag_3,0_, older adults, 50–64 years of age, showed a significant proportion of respiratory morbidity was attributable to interaction of wildfire smoke and extreme heat (AP 11.7%, 95% CI 1.4%–22.1%, *p* = 0.013), which was similar among the other age groups, 18–49 years (11.1% 95% CI −3.4%−25.6%, *p* = 0.067) and ≥65 years (AP 5.9% 95% CI −1.9%–13.7%, *p* = 0.070).

Joint effects for cerebrovascular morbidity were higher among older adults, age 50–64 years, on CHWF lag_1,3_ (AP 6.6%, 95% CI −0.6%−13.8%, *p* = 0.036) compared to the oldest group, 65 years of age and over, (−1.1%, non‐significant) and young adults 18–49 years of age (−6.7%, non‐significant). Individuals aged 50–64 years consistently showed significant joint effects for cerebrovascular morbidity across other combinations of exposure lag as well, with a tendency to increase in effect size from smoke lag 0 to 3. On the other hand, joint effects for cardiovascular morbidity were largest among the oldest age group, 65 years and older (AP, CHWF lag_2,0_ 7.4%, 95% CI 2.3%–12.5%, *p* = 0.002). Additionally, a significantly higher proportion of cardiovascular morbidities were attributable to interaction within this age group, adults ≥65 years (5.7%, 95% CI 1.1%–10.4%, *p* = 0.008), on CHWF lag_3,0_ compared to adults aged 50–64 years (−4.3%, non‐significant). The additive effects for renal morbidities differed between adults aged 50–64 years and ≥65 years; however, due to small numbers, results for renal morbidity in the 18–49 years age group were suppressed. Among the two age groups compared, the proportion of renal morbidities attributable to interaction was larger for adults aged 50–64 years (AP, CHWF lag_0,3_ 15.5% 95% CI −1.6%−32.6%, *p* = 0.038 compared to a non‐significant 0.2% among adults ≥65 years of age).

#### 3.1.3 Preferred Language Spoken

We also examined joint effects by patient's preferred language reported at hospital admission. The case population was predominantly English speakers (85.1%; Table 1); thus, there were few cases exposed to wildfire smoke and extreme heat within the Spanish and Other language groups. We suppressed results with less than 10 exposed cases within the subgroup, which limits many of the results reported for Other and Spanish language preference. The effects within the English‐speaking subpopulation generally reflect the effects showed among total study population and comparison to other language strata is limited due to small numbers. However, we do show the proportion of cerebrovascular morbidity attributable to interaction of wildfire smoke and extreme heat is significant among Spanish speakers on multiple exposure lag combinations, and effects are much larger compared to English speakers. For example, on CHWF lag_3,0_, 10.7% of cerebrovascular morbidities were attributable to the interaction among Spanish speakers (95% CI −1.5%−22.9%, *p* = 0.0428; Table S3 in Supporting Information S1) compared to a non‐significant 1.9% among English speakers.

#### 3.1.4 Race and Ethnicity

There were race groups with too few individuals to measure effects within all cause‐specific endpoints and exposure groups. Results for the “Other” race group are not reported due to small cells. Additionally, results for cardiovascular, renal, and respiratory morbidities in Asian (*N* = 47 effect estimates) and Black (*N* = 27 effect estimates) subgroups were suppressed. We found that the joint effects of wildfire smoke and extreme heat were most pronounced for Black individuals. This subgroup experienced the largest additive effects for respiratory (AP, CHWF lag_0,3_ 19.2%, 95% CI 6.5%–32.1%, *p* = 0.0016; Table S3 in Supporting Information S1) and cerebrovascular morbidities (AP, CHWF lag_0,3_ 15.7%, 95% CI 4%–27.4%, *p* = 0.0042; Table S3 in Supporting Information S1) across all analyses. In comparison, cerebrovascular effects were significantly smaller for White, non‐Hispanic individuals (2.3%) and respiratory effects were smaller for Hispanic individuals (−5.3%).

Patterns for cardiovascular morbidities suggest that Black individuals were primarily exposed to heat during lags 0 and 1, with limited exposure on lags 2 and 3. This may be due to harvesting effects, where individuals who will get sick succumb to cardiovascular morbidities sooner following a heat exposure. As a result, the burden of cardiovascular morbidity may change over time as individuals have already succumbed to illness. Similarly, respiratory morbidities were not observed for heat lag 3, particularly for exposures to smoke prior to hospitalization.

In contrast, the pattern for cerebrovascular morbidities shows a distinct increase in effects across smoke lag 0 to 3, suggesting exposure to wildfire smoke 3 days prior is a potential driver in this synergistic effect within the Black subgroup. Additionally, our findings indicate a significant protective effect for cardiovascular morbidities among Black individuals on CHWF lag_0,3_ (AP, −32.3%, 95% CI −68.4%–3.7%, *p* = 0.0394; Table S3 in Supporting Information S1). The additive effect has a wide confidence interval, although this effect does persist on the multiplicative scale as well (Multiplicative, 0.75, 95% CI 0.58–0.99, *p* = 0.045; Table S4 in Supporting Information S1). In this instance, the effect of extreme heat, without wildfire smoke, shows a strong, significantly increased risk of cardiovascular morbidity by roughly 22% (OR 1.22, 95% CI 1.02–1.46, *p* = 0.0293; Table S5 in Supporting Information S1). Yet, when exploring the opposite: the effect of wildfire smoke without extreme heat, and the interaction between extreme heat and wildfire smoke, the findings were null effects. The data may be insufficient to fully estimate the joint effects of extreme heat and wildfire smoke on cardiovascular morbidity among Black individuals, who represent approximately 10.5% of total cardiovascular cases.

Both White (CHWF lag_1,3_ and lag_2,3_) and Hispanic (CHWF lag_3,2_) subgroups showed that roughly 10% of renal morbidities were attributable to the interaction of wildfire smoke and extreme heat. Additionally, the additive effects for cardiovascular morbidity on CHWF lag_3,0_ were slightly higher for White individuals (7.7%) compared to Hispanic individuals (4.8%), though the difference was not statistically significant.

#### 3.1.5 Household Poverty, ZCTA Level

Significant additive effects for ZCTAs with higher proportion of households living under poverty (≥25%) were found for cardiovascular (AP, CHWF lag_0,0_ 7.2%, 95% CI −1.1%−15.5%, *p* = 0.045) and respiratory (AP, CHWF lag_1,1_ 10%, 95% CI 0.02%–20%, *p* = 0.025) morbidity, limited to a few combinations of exposure lags. The cardiovascular and respiratory effects were smaller (0.07% and 2.3%, respectively) for ZCTAs with lesser poverty (<25% of households living under poverty), although not significantly different. Most cases (81.2%) resided in ZCTAs with lesser poverty.

#### 3.1.6 Educational Attainment, ZCTA Level

Nearly 70% of cases resided in ZCTAs where more than 50% of the population had higher than a high school education. Findings in this group were different than those in ZCTAs having lower education. For instance, higher education ZCTAs showed larger additive effects for respiratory morbidity (AP, CHWF lag_0,3_ 12% 95% CI 4.5%–19.4%, *p* < 0.001) compared to lower education ZCTAs (AP, CHWF lag_0,3_ 3%, non‐significant) across multiple exposure lag combinations. In contrast, lower education ZCTAs showed larger and significant joint effects for cardiovascular and renal morbidity. Around 9% of cardiovascular morbidities in lower education ZCTAs were attributed to interaction of wildfire smoke and extreme heat, compared to 4.3% in higher education ZCTAs for the same exposure lags. Furthermore, renal morbidity risks were significantly larger among the lower education group (13.4%) compared to higher education group (2.1%).

#### 3.1.7 Rural and Urban Typology, ZCTA Level

Most cases resided in predominantly urban ZCTAs (97.2%); thus, the data was not sufficient to estimate joint effects of wildfire smoke and extreme heat in rural populations.

### 3.2 Sensitivity Analyses

We examined different definitions for extreme heat and wildfire smoke exposure, including binary and continuous estimates. Table 2 summarizes the distribution of cases for binary indicators. Models testing the different cutoffs for extreme heat days (90th, 95th, and 99th percentiles) showed effects of extreme heat followed a dose response, with larger effects for higher temperature exposure days. Effects were diminished for the 90th percentile, particularly for renal morbidities, suggesting risks may be underestimated. On the other hand, the 99th percentile cutoffs were too conservative for the range of *T*_max_. There were few cases with exposure to wildfire smoke above 12 μg/m^3^. Using a continuous estimate of WF‐Influenced PM_2.5_ with heat defined by the 95th percentile provided tighter confidence intervals for effect estimates compared to a binary indicator where WF‐Influenced PM_2.5_ is greater than zero.

**Table 2:** The Number of Exposed Cases and the Corresponding Percentage of Total Cases for Each Outcome Group That Were Exposed by Definitions for Extreme Heat (Heat) and Wildfire Smoke (Smoke)

	Number of exposed cases (Percentage of total cases exposed)				
	All‐natural	Respiratory	Cardiovascular	Cerebrovascular	Renal
Heat defintions					
90th percentile	2,861,563 (10.1%)	4,828 (9.6%)	6,548 (10.2%)	9,078 (10.1%)	3,449 (10.4%)
95th percentile	1,463,144 (5.2%)	2,442 (4.9%)	3,305 (5.2%)	4,559 (5.1%)	1,775 (5.3%)
99th percentile	340,093 (1.2%)	560 (1.1%)	779 (1.2%)	1,036 (1.2%)	408 (1.2%)
Smoke defintions					
>0 μg/m^3^	703,709 (2.5%)	894 (1.8%)	1,508 (2.4%)	2,278 (2.5%)	662 (2.0%)
>12 μg/m^3^	36,065 (0.1%)	38 (0.1%)	79 (0.1%)	114 (0.1%)	28 (0.1%)

*Note.* Cutoffs include the month‐ and ZCTA‐specific 90th, 95th, and 99th percentile thresholds for heat and WF‐Influenced PM2.5 values above 0 and 12 μg/m^3^.

We further tested the sensitivity of our models to meaningful differences during the study period. While ICD coding systems changed from 2015 to 2016, there were additional disruptions in exposure and outcome characterization: medical diagnoses for renal morbidities were systematically refined (Watzlaf et al., 2007), and there were different patterns of extreme heat and wildfire smoke activity. Models subset to 2011 to 2015 and 2016 to 2019 both show additive effects for respiratory morbidity, though the effects are larger in 2011–2015. We found significant additive effects for cardiovascular morbidity from 2016 to 2019 and significant additive effects for cerebrovascular morbidity from 2011 to 2015. However, the patterns of effects across exposure lags were analogous. These results do not fully control for all differences between these two timeframes, that is, change in coding, diagnostic practices, and exposures. Nonetheless, these findings provide a general sense that the relationships between the outcomes and exposures are robust.

Exposures to compound hazards were highest in 2017 and 2020. However, the COVID‐19 pandemic beginning in 2020 potentially influenced risk behaviors, health care utilization, and ambient air pollution. Results testing models for these years separately showed significant sub‐additive interactions for cerebrovascular, renal, and respiratory morbidities in 2020. In contrast, we found significant super‐additive effects for cardiovascular and respiratory morbidities in 2017. For example, during the pandemic we show a sub‐additive effect of −12% (95% CI –26%–0%, *p* = 0.030 for respiratory morbidity on CHWF lag_0,1_), while in 2017 the same exposure lag and outcome showed a super‐additive effect of 8% (95% CI 0%–16%, *p* = 0.030 for respiratory morbidity on CHWF lag_0,1_). Pandemic‐related factors, such as change in healthcare utilization, adoption of health protective behaviors, and sheltering in place, likely contribute to the difference in joint effects of wildfire smoke and extreme heat. We lack individual level data on these factors; thus, models for primary analysis were restricted to 2011 to 2019.

We also tested the sensitivity of restricting the analysis to wildfire and extreme heat seasons, May through November, by examining effects for all months. The pattern of effects across exposure lags and outcomes of interest were preserved, although effects in the seasonal months were slightly stronger (Tables S1 and S6 in Supporting Information S1). For example, the same respiratory effect on CHWF lag_0,3_ shows the attributable proportion due to interaction (AP) is slightly higher at 8.1%, while the estimate for all months, 5.5%, is still well within the 95% confidence interval range, 2.3%–13.8% (*p* = 0.003; Table S1 in Supporting Information S1).

Analyzing effects within summer months improved characterization of extreme heat events. The average *T*_max_ on days above the 95th percentile cutoff for all months from 2011 to 2020 was 76.4° Fahrenheit, whereas average *T*_max_ above this cutoff when restricting to May through November was 82.9°. Other studies suggest joint effects may be inconsistent across all months due to differences in adaptive behaviors or hazards, and burdens are greatest during the summer season (Analitis et al., 2018; Austin et al., 2021).

## 4 Discussion

California is confronting an emerging threat of compounding climate hazards. Longer and more severe wildfire seasons are increasingly exposing populations to both extreme heat and wildfire smoke (Masri et al., 2022; Rosenthal et al., 2022). Our study found that compound wildfire smoke and extreme heat synergistically impact health, with impacts varying by cause‐specific outcome, exposure lags, and population subgroups. This area of compound hazard research has been advanced by estimation of wildfire‐related PM_2.5_. For instance, our study uses a direct estimate of the influence of wildfire smoke on PM_2.5_, (i.e., WF‐Influenced PM_2.5_), which was modeled based on empirical knowledge of wildfire smoke (HMS Smoke product) and exceedances of expected smoke‐free concentrations. There are limited studies on the effects of wildfire smoke and extreme heat, and fewer that account for the specific contributions of wildfire smoke to PM_2.5_ estimates.

Chen et al. (2024) similarly found synergistic effects on cardiorespiratory hospitalizations in California from 2006 to 2019. Authors examined exposures to wildfire smoke and extreme heat on the same day using a similar measure of wildfire‐specific PM_2.5_, which combines estimates from air quality monitors, meteorological data, and the HMS smoke product for wildfire smoke contributions (Aguilera et al., 2023). In contrast, other studies have shown joint effects of wildfire‐related air pollution and extreme heat for all‐cause, cardiovascular, and respiratory mortalities (Rahman et al., 2022), as well as emergency department admissions (Patel et al., 2019) by examining high concentrations of PM_2.5_, which authors suggest are attributable to wildfire activity. One study goes so far as to correlate days with high concentrations of PM_2.5_ with wildfire events (Rahman et al., 2022).

Additionally, prior studies on the combination of wildfire smoke and extreme heat exposures during short‐term exposure lag windows have only assessed exposures on the same lag days. We thoroughly examined compound exposures within a short‐term exposure lag window, testing 16 different combinations of exposure lags. Importantly, multiple hazard exposure on the same day does not necessarily dictate the effects. Rather, different combinations of exposure within a short‐term window can have joint health effects. The inconsistency in findings across lag combinations is likely reflective of the natural variability in the co‐occurrence of heat and smoke exposures, which do not follow a predictable pattern. Unlike independent exposures, where a clear physiologic or behavioral response dictates the timing between exposure and health event, the combined effects of heat and smoke on health may be influenced by complex, less predictable interactions.

For instance, we note that effects of extreme heat, without wildfire smoke, were most often significant on heat lag days 0 and 2. This pattern was particularly apparent among renal morbidities. We also observed significant effects of wildfire smoke, without extreme heat, often on smoke lag days 2 and 3. The differences in the associations of lag days for these individual effects suggest it is important to consider the different combinations of exposure lags when estimating joint effects. Our results show significant effects of different combinations of wildfire smoke and extreme heat exposure and cause‐specific morbidities. These findings indicate that exposure consideration should not be limited to days when both hazards have occurred, but rather short‐term periods that capture multiple hazards.

This study provides evidence for the joint effects of wildfire smoke and extreme heat on both multiplicative and additive scales for cardiovascular, cerebrovascular, renal, and respiratory morbidities. Effects for all‐natural cause morbidity were all small and close to null. However, we found that cause‐specific end points showed joint effects that differed across exposure lags and population subgroups. Prior literature shows these outcomes have varied associations when looking at wildfire smoke and extreme heat exposures independently.

There is extensive literature showing significantly increased risk for hospitalizations of respiratory morbidities associated with wildfire smoke exposure (Cascio, 2018; Reid, Brauer, et al., 2016). Similarly, hot days have been linked to increased risk of respiratory morbidity, as noted in a review and meta‐analysis of extreme temperatures and cardiorespiratory morbidity (Turner et al., 2012). Our findings extend this knowledge by demonstrating that when hazards compound, the effects of wildfire smoke and extreme heat lead to super additive and multiplicative interactions that further elevate the risk of respiratory morbidities. Furthermore, a study of extreme heat in New York projects the burden of respiratory admissions due to extreme heat will increase by roughly 2 to 6 times in 2080–2099 compared to 1991–2004 (Lin et al., 2012). While few studies estimate future respiratory health impacts of wildfire smoke under climate change scenarios, current evidence suggests increasing exposures to wildfire smoke will lead to growing respiratory impacts (Reid & Maestas, 2019). This underscores the need to consider how climate change might affect the observed joint effects of wildfire smoke and extreme heat.

In contrast to respiratory morbidities, Turner's meta‐analysis found the relationship between extreme temperatures and cardiovascular morbidity was less clear, finding few studies, which show no effects (Turner et al., 2012). A more recent review and meta‐analysis from 2016 found the relationship between heat exposure, using heat thresholds like the metric in our study, and cardiovascular morbidities was inconsistent, although significant risks from heat waves were reported (Phung et al., 2016). Other studies have shown more consistent links between extreme heat and cardiovascular mortality compared to morbidity (Phung et al., 2016; Åström et al., 2011). Although cardiovascular morbidity risk is not commonly reported in heat studies, a recent review from Chen et al. (2021) on wildfire smoke and cardiovascular health found many studies (25 out of the 38 retrieved) showing increased risks for cardiovascular morbidities. Our results add emphasis to this by identifying synergistic effects of extreme heat and wildfire smoke on cardiovascular morbidities.

Regarding renal health, there is a lack of evidence for the impact of wildfire smoke on renal health. One study shows increased mortality in a vulnerable population of hemodialysis patients with end stage kidney disease exposed to wildfire PM_2.5_ (Xi et al., 2020). To our knowledge, no studies have been published exploring the risk of wildfire smoke and renal morbidity in the general population. There is, however, extensive evidence of the impact extreme heat and heat waves have on renal health (Johnson et al., 2019). The kidneys serve a critical role in protecting individuals from heat and dehydration. They do this by maintaining adequate blood volume. When a person experiences heat stress or heatstroke, the kidneys can become overwhelmed, leading to decreased circulating blood volume, resulting in kidney dysfunction. Our study found near‐significant joint effects of wildfire smoke and extreme heat on renal morbidity, with more pronounced effects revealed in subgroup analyses by race and ethnicity, preferred language, and educational attainment.

Lastly, while there is limited evidence on the association between wildfire smoke and cerebrovascular risks, Wettstein et al. (2023) identified increased cerebrovascular risks associated with wildfire smoke in a similar population of California hospitalizations. Evidence for cerebrovascular health associations with extreme heat is more extensive (Åström et al., 2011; Bunker et al., 2016; Zhang et al., 2014), although not always consistent (Zorrilla‐Vaca et al., 2017). Our results highlight the importance of further investigating the differential impacts of wildfire smoke and extreme heat on cerebrovascular morbidities, particularly within population subgroups.

Joint effects differed by individual and community level factors. For example, effect estimates for renal morbidity were stronger and significant for the oldest age group, English speakers, White and Hispanic individuals, and ZCTAs with lower educational attainment and higher proportions of households living under poverty. We also did not find joint effects of wildfire smoke and extreme heat on cerebrovascular morbidities among the study population but found significant super additive and multiplicative interactions within subgroups. This includes a moderate portion of cerebrovascular morbidities attributable to interaction for the Black subpopulation. This analysis also shows significant joint effects on cerebrovascular morbidities for White, young to middle aged and older adult, and English preferred language groups.

Cardiovascular and respiratory effects differed in stratified analyses as well. For instance, joint effects for cardiovascular morbidity were stronger within the Black and female subgroups, whereas respiratory joint effects were stronger in males. Prior single hazard assessments have also shown disparate impacts for these health outcomes across similar sociodemographic characteristics, including sex, age, education, and income (Rappold et al., 2012; Reid et al., 2009, 2016b). Our study shows joint effects of wildfire smoke and extreme heat differ across individual and community level risk factors. Differences may be driven or modified by underlying vulnerabilities, social or place‐based disadvantages, and risk behaviors.

Sex‐differences found in our study show females had higher joint effects for cardiovascular morbidity. Cardiovascular disease is the leading cause of death for women, and with a higher prevalence than men (Appelman et al., 2015). Female‐specific biologic risk factors, including preeclampsia, gestational diabetes and menopause onset, and differences in the effects of behavioral risk factors contribute to increased cardiovascular risks for women (Appelman et al., 2015). Males, however, were found to have higher joint effects for risk of respiratory morbidity in our study. Shin et al. (2022) also found higher risk of respiratory hospitalization due to air pollution exposure in males compared to females during the summer months (Shin et al., 2022). These findings suggest that differences may be due to many factors, including physiologic structures, lifestyle, and underlying inflammatory diseases. For instance, a survey of activity patterns found men spend more time in outdoor environments (Matz et al., 2014), which may suggest greater exposures to outdoor air pollutants and extreme temperatures.

Differences in biologic risk and risk behaviors may also explain differential joint effects by age. There is substantial literature indicating that older populations have higher risks of health impacts to extreme heat and wildfire smoke, independently (Åström et al., 2011; Cascio, 2018; Liu et al., 2017; Zhang et al., 2014). The findings of joint effects for young to middle aged and older adults are also of interest. Increased risks for respiratory morbidities were present across all age groups. However, it is plausible that risk behaviors, underlying comorbidities, and physiologic health are contributing to this effect differently among age groups (Deeks et al., 2009).

It is also possible that differential effects by individual characteristics, such as language preference and race and ethnicity, may serve as proxies for social disadvantages. For instance, language preferences can influence access to risk information, an important predictor for the adoption of health protective behaviors. However, interpretations of effects for individuals with a language preference of Spanish and other than English or Spanish in our study are limited due to small number of exposed cases. We are not able to determine if this plays a role in the joint effects of wildfire smoke and extreme heat.

However, race and ethnicity effects are also deeply influenced by socioeconomic status and often interpreted in environmental epidemiologic literature to be driven by inequalities among race groups and systemic racism. Minority populations often carry a higher burden of environmental exposures and health impacts. Our results show the strongest joint effects of wildfire smoke and extreme heat within the Black subpopulation; although, some of the effects for Black and other minority race groups had to be suppressed, and thus the interpretations are limited.

Neighborhood‐level characteristics can also shed light on the relationship of joint effects of wildfire smoke and extreme heat with socioeconomic conditions and often related, the capacity to adapt. Chen et al. (2024) found spatial heterogeneity in the joint effects of wildfire smoke and extreme heat on cardiorespiratory morbidity. Similarly, they show higher risks in California ZCTAs with greater disadvantages, such as lower income, lower educational attainment, higher minority race populations, and reduced green spaces. This spatial heterogeneity in effects has also been shown for respiratory health impacts of wildfire smoke across California ZCTAs (Do et al., 2024). Educational attainment and household poverty status are frequently associated with greater environmental exposure burdens and impacts (Bao et al., 2015; Bell & Keita, 2012; Curriero et al., 2002; Rappold et al., 2012; Reid et al., 2009). Those living in poverty often do not have the capacity to afford mitigations (Bao et al., 2015). We did not observe significant differences in the joint effects among communities living in ZCTAs with more households living under the poverty. Our results, however, do show a moderate effect on renal morbidity for ZCTAs where 50% or more of the population obtained a high school education or less. Prior research shows education is a key determinant of renal health outcomes as it can predict occupation, income, and adoption of health protective behaviors (Lombardi et al., 2021; Mirowsky & Ross, 2015). Our results for the lower education group suggest socioeconomic conditions may be a key determinant for the joint effects of wildfire smoke and extreme heat on renal morbidity. However, differences relating to social and place‐based disadvantages should be investigated further.

Further, we investigated the joint effects of wildfire smoke and extreme heat by rural and urban typologies. Exposure predominantly covered urban areas; thus, we could not estimate effects in rural areas. Urban areas may be more susceptible to these compound events due to the urban heat island effect (Chapman et al., 2017). Additionally, densely populated urban areas present a critical concern for exposure risks.

There are some limitations to this study that should be considered. Exposure misclassification may occur when assigning exposure to residential ZCTA as it roughly approximates exposure and the individual's location during exposure periods. Additionally, the ZCTA geography is a spatial unit that provides information regarding where individuals live (Graham et al., 2015), but primarily serves as an arbitrary unit for postal routes. There is wide variation in the geographic size and population density between ZCTAs. Similar to our approach, other environmental epidemiologic studies have used ZIP Code level analyses in California with adjustments for population distribution within the ZIP Code (Delfino et al., 2009; Guirguis et al., 2018; Knowlton et al., 2009; Riley et al., 2018). We aimed to minimize exposure misclassification by estimating exposure at the ZCTA level based on population distribution.

Furthermore, ZCTA characteristics such as educational attainment, percent living in poverty, and rural and urban typology may serve as proximate indicators of vulnerability, influencing both exposure and health outcomes. For example, ZCTAs with lower educational attainment or higher poverty levels may have different vulnerability profiles to environmental hazards, which could be confounded by other factors not captured in this study (e.g., access to healthcare, housing quality, or local environmental conditions). We used binary stratification of ZCTAs based on these characteristics to examine joint effects, but future research should consider more granular or individualized measures of exposure and account for these potentially confounding factors.

We also cannot assess whether individuals adopted health protective behaviors to mitigate exposure. For instance, staying indoors, reducing outdoor physical activity, or using air filtration and air conditioning devices are examples of behavioral modifications that may influence an individual's exposure to extreme heat or wildfire smoke. Additionally, this study examines single days of extreme temperature and wildfire smoke, rather than consecutive days, that is, heat waves and smoke waves. Rosenthal et al. (2022) found co‐occurrence of heat waves and smoke waves captured a higher degree of exposure, thus, health effects may be stronger. Further research could investigate how health effects may differ with consecutive or cumulative days of exposure.

We also did not observe meaningful effects for all‐natural cause morbidity, which may be due to the broad range of conditions included in this category, each of which may have different relationships to wildfire smoke and extreme heat. Additionally, the type of hospital admission (e.g., emergency, urgent, or prescheduled) could influence these outcomes. Prescheduled hospitalizations, in particular, may have a different relationship with exposure, as individuals may already have planned admissions, and while they could still experience exposure, the timing and context of exposure (e.g., in a hospital setting rather than in the community) may alter its impact compared to emergency or urgent admissions. Lastly, joint effects analysis requires larger sample sizes than independent association analyses. Despite some of the highest combined exposures to wildfire smoke and extreme heat in 2020, we excluded 2020 data from the primary and stratified analyses due to effects of the COVID‐19 pandemic. Thus, exposures within some population subgroups were too small to estimate joint effects. With increasing wildfire activity and extreme heat events, researchers should investigate more recent years of compound wildfire and extreme heat events in California.

Future investigations could explore the relationship between apparent temperature, which accounts for humidity, wildfire smoke, and health outcomes, given that California's climate is becoming more humid (Gershunov & Guirguis, 2012). Likewise, air pollutants, like ozone, may also play a role in the joint effects for extreme heat and wildfire smoke. Incorporating ozone into these analyses may further explain some of the effects we observed. Lastly, we examined wildfire smoke and extreme heat events compounding within a short‐term period. Future studies could consider different spatial and temporal components of compound effects, including the effects of events compounding response resources at larger geographic scales rather than just among compounding exposed populations.

## 5 Conclusions

This study shows worsening wildfire and extreme heat events in California are leading to stronger morbidity effects due to compound wildfire smoke and extreme heat. Disparities in the joint effects of wildfire smoke and extreme heat highlight the need to target interventions for populations at greater risk, which varies by outcome. It is also important to consider how these effects may be influenced by a changing climate. These extremes are expected to increase; thus, attention should focus on assessing strategies to jointly mitigate risks.

## Conflict of Interest

The authors declare no conflicts of interest relevant to this study.

## Supporting information

Supporting Information S1

## Data Availability

The climate exposure data used in this study for California, 2011–2020, are referenced in text and available in Jones‐Ngo et al. (2024) for daily wildfire‐influenced PM_2.5_ at 3‐km resolution; Abatzoglou (2013) for daily maximum temperature, 4‐km resolution; and Doxsey‐Whitfield et al. (2015) for 1‐km gridded population estimates. Confidential health data from the California Department of Health Care Access and Information is available to eligible requestors via https://hcai.ca.gov/data/request‐data/. Statistical analyses were computed using *R* (version 2023.03.0, build 386). *R* is an open‐source statistical computing software available at https://www.r‐project.org/.
