# Supplementary material for: Joint Effects of Wildfire Smoke and Extreme Heat on Hospitalizations in California, 2011–2020
Source: Geohealth. 2025 Jun 6;9(6):e2024GH001237. doi: 10.1029/2024GH001237 (PMC12143023; doi:10.1029/2024GH001237)
Supplement: Supplementary file 1 — Supporting Information S1 [file GH2-9-e2024GH001237-s001.pdf]

Supporting Information for

Joint effects of wildfire smoke and extreme heat on hospitalizations in California, 2011-2020

Caitlin G. Jones-Ngo[1,2], Rebecca J. Schmidt[1], Erwan Monier[3], Sara Ludwick[4], Mohammad Z. Al-Hamdan[5,6], Jason Vargo[7], and Kathryn C. Conlon[1]

[1]Department of Public Health Sciences, University of California, Davis, CA, USA.

[2]Scripps Institution of Oceanography, University of California, San Diego, CA, USA.

[3]Department of Land, Air and Water Resources, University of California, Davis, CA, USA.

[4]Department of Environmental Science and Policy, University of California, Davis, CA, USA.

[5]National Center for Computational Hydroscience and Engineering (NCCHE), School of Engineering, University of Mississippi, Oxford, MS, USA.

[6]Department of Civil Engineering, School of Engineering, University of Mississippi, Oxford, MS, USA.

[7]Independent Researcher.

Contents in this file

Tables S1 to S6

Introduction

This supporting information provides effect estimates seen in the main text with 95% confidence intervals (CI) and p-values for attributable proportion due to interaction. This also include tables for the multiplicative interaction effects, stratified effects, and independent effects of wildfire smoke and extreme heat.

Table S1 Attributable proportion due to interaction (AP) with upper and lower limits (UL and LL, respectively) for 95% confidence intervals (CIs) and p-values (bolded indicates significant $p < 0.05$) for the joint effects of wildfire smoke and extreme heat, 2011-2019, May to November. Separate models were run for combinations of lag 0-3 and each outcome of interest: all-natural cause, cardiovascular, cerebrovascular, renal, and respiratory morbidity.

OOI	Extreme Hea	Wildfire Smoke	AP	CI LL	CI UL	P-value
All-natural	Lag 0	Lag 0	0.002	0.000	0.004	**0.028**
		Lag 1	0.000	-0.002	0.002	0.368
		Lag 2	-0.004	-0.006	-0.001	**0.003**
		Lag 3	0.001	-0.002	0.003	0.226
	Lag 1	Lag 0	0.003	0.001	0.005	**0.000**
		Lag 1	-0.001	-0.003	0.001	0.084
		Lag 2	-0.003	-0.005	-0.001	**0.002**
		Lag 3	0.001	-0.002	0.003	0.342
	Lag 2	Lag 0	0.004	0.002	0.006	**0.000**
		Lag 1	-0.002	-0.003	0.000	**0.032**
		Lag 2	-0.005	-0.007	-0.003	**0.000**
		Lag 3	-0.002	-0.004	0.000	**0.022**
	Lag 3	Lag 0	0.005	0.002	0.007	**0.000**
		Lag 1	0.002	0.000	0.003	**0.046**
		Lag 2	-0.004	-0.006	-0.002	**0.000**
		Lag 3	-0.001	-0.003	0.001	0.116
Cardiovascular	Lag 0	Lag 0	0.014	-0.025	0.052	0.244
		Lag 1	0.014	-0.025	0.052	0.245
		Lag 2	0.011	-0.048	0.069	0.361
		Lag 3	-0.035	-0.096	0.026	0.129
	Lag 1	Lag 0	0.023	-0.014	0.061	0.113
		Lag 1	0.008	-0.029	0.044	0.334
		Lag 2	0.028	-0.014	0.071	0.094
		Lag 3	-0.007	-0.063	0.048	0.397
	Lag 2	Lag 0	0.049	0.009	0.088	**0.008**
		Lag 1	-0.011	-0.044	0.022	0.259
		Lag 2	-0.005	-0.046	0.036	0.405
		Lag 3	-0.015	-0.058	0.027	0.240
	Lag 3	Lag 0	0.055	0.008	0.102	**0.011**
		Lag 1	-0.002	-0.034	0.031	0.457
		Lag 2	-0.020	-0.060	0.021	0.170
		Lag 3	-0.001	-0.040	0.038	0.477
Cerebrovascular	Lag 0	Lag 0	-0.001	-0.034	0.033	0.482
		Lag 1	-0.001	-0.034	0.032	0.473
		Lag 2	0.015	-0.031	0.061	0.262

		Lag 3	0.028	-0.016	0.072	0.109
	Lag 1	Lag 0	0.010	-0.022	0.042	0.267
		Lag 1	0.006	-0.022	0.035	0.331
		Lag 2	0.002	-0.035	0.039	0.456
		Lag 3	0.009	-0.035	0.054	0.340
	Lag 2	Lag 0	0.009	-0.027	0.046	0.309
		Lag 1	0.010	-0.015	0.034	0.217
		Lag 2	-0.007	-0.038	0.025	0.339
		Lag 3	0.015	-0.017	0.048	0.178
	Lag 3	Lag 0	0.028	-0.011	0.068	0.078
		Lag 1	0.012	-0.016	0.039	0.202
		Lag 2	0.010	-0.015	0.035	0.207
		Lag 3	0.020	-0.008	0.048	0.084
Renal	Lag 0	Lag 0	0.023	-0.027	0.073	0.185
		Lag 1	-0.019	-0.090	0.052	0.303
		Lag 2	0.039	-0.033	0.111	0.145
		Lag 3	0.057	-0.026	0.141	0.089
	Lag 1	Lag 0	0.032	-0.015	0.079	0.089
		Lag 1	-0.017	-0.084	0.050	0.309
		Lag 2	-0.030	-0.111	0.051	0.233
		Lag 3	0.062	-0.011	0.134	**0.048**
	Lag 2	Lag 0	-0.006	-0.068	0.057	0.429
		Lag 1	-0.012	-0.081	0.057	0.369
		Lag 2	0.004	-0.061	0.068	0.455
		Lag 3	0.050	-0.006	0.105	**0.039**
	Lag 3	Lag 0	-0.053	-0.138	0.032	0.111
		Lag 1	-0.029	-0.120	0.061	0.262
		Lag 2	0.004	-0.052	0.059	0.449
		Lag 3	0.016	-0.033	0.065	0.264
Respiratory	Lag 0	Lag 0	0.010	-0.051	0.072	0.368
		Lag 1	0.036	-0.020	0.092	0.104
		Lag 2	0.001	-0.068	0.070	0.494
		Lag 3	0.081	0.024	0.138	**0.003**
	Lag 1	Lag 0	-0.005	-0.069	0.058	0.435
		Lag 1	0.039	-0.011	0.089	0.062
		Lag 2	-0.010	-0.067	0.047	0.368
		Lag 3	0.080	0.020	0.140	**0.005**
	Lag 2	Lag 0	0.056	0.006	0.105	**0.014**
		Lag 1	0.038	0.001	0.075	**0.021**
		Lag 2	-0.004	-0.058	0.049	0.437
		Lag 3	0.043	-0.014	0.100	0.071
	Lag 3	Lag 0	0.003	-0.061	0.067	0.461

	Lag 1	0.016	-0.015	0.047	0.158
	Lag 2	-0.019	-0.077	0.039	0.260
	Lag 3	0.031	-0.021	0.083	0.123

Table S2 Multiplicative odds ratio with upper and lower limits (UL and LL, respectively) for 95% confidence intervals (CIs) and p-values (bolded indicates significant p < 0.05) for the joint effects of wildfire smoke and extreme heat. Separate models were run for combinations of lag 0-3 and each outcome of interest: all-natural cause, cardiovascular, cerebrovascular, renal, and respiratory morbidity.

OOI	Extreme Heat	Wildfire Smoke	Multiplicative	CI LL	CI UL	P-value
All-natural	Lag 0	Lag 0	1.002	1.000	1.004	0.057
		Lag 1	1.000	0.998	1.002	0.734
		Lag 2	0.996	0.994	0.999	**0.007**
		Lag 3	1.001	0.999	1.003	0.443
	Lag 1	Lag 0	1.003	1.001	1.005	**0.001**
		Lag 1	0.999	0.997	1.001	0.170
		Lag 2	0.997	0.995	0.999	**0.004**
		Lag 3	1.001	0.998	1.003	0.673
	Lag 2	Lag 0	1.004	1.002	1.006	**0.000**
		Lag 1	0.998	0.997	1.000	0.065
		Lag 2	0.995	0.993	0.997	**0.000**
		Lag 3	0.998	0.996	1.000	**0.045**
	Lag 3	Lag 0	1.005	1.002	1.007	**0.000**
		Lag 1	1.002	1.000	1.003	0.092
		Lag 2	0.996	0.994	0.998	**0.000**
		Lag 3	0.999	0.997	1.001	0.238
Cardiovascular	Lag 0	Lag 0	1.014	0.975	1.054	0.492
		Lag 1	1.014	0.975	1.054	0.499
		Lag 2	1.011	0.952	1.073	0.724
		Lag 3	0.966	0.911	1.025	0.250
	Lag 1	Lag 0	1.024	0.985	1.064	0.235
		Lag 1	1.008	0.971	1.046	0.683
		Lag 2	1.029	0.985	1.075	0.194
		Lag 3	0.993	0.940	1.049	0.796
	Lag 2	Lag 0	1.051	1.008	1.096	**0.018**
		Lag 1	0.989	0.957	1.022	0.507
		Lag 2	0.995	0.955	1.036	0.810
		Lag 3	0.985	0.944	1.027	0.479
	Lag 3	Lag 0	1.058	1.007	1.112	**0.025**
		Lag 1	0.998	0.966	1.031	0.906
		Lag 2	0.981	0.942	1.020	0.336
		Lag 3	0.999	0.961	1.038	0.956
Cerebrovascula	Lag 0	Lag 0	0.999	0.966	1.033	0.961
		Lag 1	0.999	0.966	1.032	0.944
		Lag 2	1.015	0.969	1.064	0.526
		Lag 3	1.029	0.983	1.077	0.223

	Lag 1	Lag 0	1.010	0.978	1.044	0.540
		Lag 1	1.006	0.978	1.036	0.665
		Lag 2	1.002	0.965	1.040	0.910
		Lag 3	1.010	0.965	1.056	0.677
	Lag 2	Lag 0	1.009	0.972	1.048	0.626
		Lag 1	1.010	0.985	1.035	0.438
		Lag 2	0.993	0.963	1.025	0.681
		Lag 3	1.016	0.983	1.050	0.354
	Lag 3	Lag 0	1.029	0.988	1.072	0.163
		Lag 1	1.012	0.984	1.040	0.407
		Lag 2	1.011	0.985	1.036	0.416
		Lag 3	1.020	0.991	1.050	0.170
Renal	Lag 0	Lag 0	1.024	0.973	1.078	0.366
		Lag 1	0.982	0.916	1.053	0.618
		Lag 2	1.040	0.965	1.121	0.307
		Lag 3	1.061	0.971	1.160	0.191
	Lag 1	Lag 0	1.034	0.985	1.086	0.177
		Lag 1	0.984	0.921	1.051	0.637
		Lag 2	0.970	0.896	1.050	0.446
		Lag 3	1.066	0.986	1.152	0.107
	Lag 2	Lag 0	0.995	0.934	1.059	0.865
		Lag 1	0.989	0.923	1.059	0.751
		Lag 2	1.003	0.940	1.071	0.923
		Lag 3	1.052	0.993	1.115	0.086
	Lag 3	Lag 0	0.950	0.876	1.030	0.213
		Lag 1	0.972	0.890	1.061	0.523
		Lag 2	1.003	0.949	1.061	0.907
		Lag 3	1.016	0.967	1.068	0.531
Respiratory	Lag 0	Lag 0	1.010	0.950	1.075	0.741
		Lag 1	1.038	0.979	1.100	0.215
		Lag 2	1.001	0.934	1.072	0.984
		Lag 3	1.088	1.022	1.158	**0.008**
	Lag 1	Lag 0	0.995	0.934	1.060	0.871
		Lag 1	1.041	0.988	1.097	0.131
		Lag 2	0.990	0.935	1.048	0.733
		Lag 3	1.087	1.018	1.160	**0.013**
	Lag 2	Lag 0	1.059	1.005	1.116	**0.032**
		Lag 1	1.040	1.001	1.080	**0.046**
		Lag 2	0.996	0.944	1.050	0.872
		Lag 3	1.045	0.984	1.109	0.151
	Lag 3	Lag 0	1.003	0.941	1.070	0.919
		Lag 1	1.016	0.984	1.049	0.323

	Lag 2	0.981	0.926	1.039	0.512
	Lag 3	1.032	0.978	1.089	0.256

Table S3 Stratified results for attributable proportion due to interaction (AP) with upper and lower limits (UL and LL, respectively) for 95% confidence intervals (CIs) and p-values (bolded indicates significant p < 0.05) for the joint effects of wildfire smoke and extreme heat, 2011-2019, May to Nov. Subgroups of individual and community level factors were tested in stratified models. Separate models were run for combinations of lag 0-3 and each outcome of interest: all-natural cause, cardiovascular, cerebrovascular, renal, and respiratory morbidity. Results with fewer than 10 exposed cases are suppressed.

Subgroup	OOI	Extreme Heat	Wildfire Smoke	AP	CI LL	CI UL	P-value	Exposed Case Counts
Male	All-natural	Lag 0	Lag 0	0.003	0.000	0.006	**0.027**	26016
			Lag 1	0.000	-0.003	0.003	0.440	23718
			Lag 2	-0.001	-0.005	0.003	0.314	23468
			Lag 3	0.002	-0.002	0.006	0.134	23296
		Lag 1	Lag 0	0.005	0.002	0.007	**0.000**	25713
			Lag 1	-0.002	-0.004	0.001	0.121	25405
			Lag 2	-0.001	-0.004	0.002	0.195	25439
			Lag 3	0.002	-0.002	0.006	0.126	25484
		Lag 2	Lag 0	0.006	0.003	0.009	**0.000**	23047
			Lag 1	-0.002	-0.004	0.001	0.088	25442
			Lag 2	-0.003	-0.006	0.000	**0.013**	25563
			Lag 3	-0.001	-0.004	0.002	0.229	25702
		Lag 3	Lag 0	0.007	0.004	0.011	**0.000**	23314
			Lag 1	0.002	-0.001	0.005	0.094	23420
			Lag 2	-0.004	-0.006	-0.001	**0.002**	23683
			Lag 3	0.000	-0.003	0.002	0.376	23935
	Cardiovascular	Lag 0	Lag 0	-0.004	-0.056	0.047	0.434	55
			Lag 1	0.031	-0.016	0.078	0.100	63
			Lag 2	0.006	-0.075	0.088	0.442	59
			Lag 3	-0.011	-0.078	0.056	0.374	56
		Lag 1	Lag 0	0.005	-0.049	0.059	0.431	57
			Lag 1	0.025	-0.020	0.071	0.140	62
			Lag 2	0.040	-0.017	0.097	0.086	59
			Lag 3	-0.029	-0.104	0.046	0.226	58
		Lag 2	Lag 0	0.016	-0.047	0.079	0.305	58
			Lag 1	0.014	-0.027	0.054	0.254	71
			Lag 2	0.002	-0.053	0.057	0.473	67
			Lag 3	-0.006	-0.058	0.047	0.414	63
		Lag 3	Lag 0	0.015	-0.056	0.087	0.336	69
			Lag 1	0.017	-0.023	0.057	0.196	60
			Lag 2	-0.027	-0.083	0.028	0.170	54
			Lag 3	0.020	-0.026	0.066	0.200	51
	Cerebrovascular	Lag 0	Lag 0	-0.013	-0.062	0.036	0.305	94
			Lag 1	-0.020	-0.076	0.037	0.249	92
			Lag 2	0.047	-0.014	0.108	0.065	91
			Lag 3	0.011	-0.055	0.076	0.372	93

		Lag 1	Lag 0	0.001	-0.046	0.048	0.483	99
			Lag 1	-0.007	-0.054	0.039	0.379	99
			Lag 2	-0.015	-0.071	0.041	0.299	99
			Lag 3	-0.016	-0.083	0.051	0.319	101
		Lag 2	Lag 0	-0.039	-0.107	0.030	0.135	75
			Lag 1	0.004	-0.032	0.040	0.407	106
			Lag 2	-0.005	-0.044	0.034	0.404	105
			Lag 3	-0.009	-0.057	0.038	0.349	108
		Lag 3	Lag 0	-0.002	-0.059	0.055	0.473	87
			Lag 1	0.012	-0.025	0.048	0.267	102
			Lag 2	0.019	-0.010	0.048	0.103	103
			Lag 3	0.006	-0.032	0.044	0.375	104
	Renal	Lag 0	Lag 0	0.027	-0.045	0.098	0.231	32
			Lag 1	-0.019	-0.120	0.081	0.353	36
			Lag 2	0.062	-0.031	0.154	0.096	34
			Lag 3	0.001	-0.148	0.149	0.496	34
		Lag 1	Lag 0	0.032	-0.042	0.107	0.198	28
			Lag 1	-0.025	-0.123	0.073	0.308	36
			Lag 2	-0.044	-0.171	0.082	0.247	36
			Lag 3	0.078	-0.026	0.183	0.071	36
		Lag 2	Lag 0	-0.014	-0.107	0.079	0.386	17
			Lag 1	-0.041	-0.148	0.066	0.227	26
			Lag 2	-0.019	-0.116	0.077	0.347	27
			Lag 3	0.028	-0.057	0.113	0.261	27
		Lag 3	Lag 0	-0.126	-0.282	0.030	0.057	23
			Lag 1	-0.038	-0.176	0.100	0.295	28
			Lag 2	0.016	-0.080	0.113	0.371	29
			Lag 3	0.027	-0.055	0.110	0.257	28
	Respiratory	Lag 0	Lag 0	0.045	-0.047	0.136	0.169	39
			Lag 1	0.088	0.013	0.163	**0.011**	40
			Lag 2	0.060	-0.028	0.148	0.089	40
			Lag 3	0.162	0.077	0.247	**0.000**	39
		Lag 1	Lag 0	-0.044	-0.163	0.075	0.236	29
			Lag 1	0.060	-0.010	0.130	**0.047**	43
			Lag 2	0.007	-0.063	0.077	0.423	43
			Lag 3	0.125	0.038	0.213	**0.003**	42
		Lag 2	Lag 0	0.077	0.002	0.152	**0.023**	42
			Lag 1	0.033	-0.025	0.091	0.134	43
			Lag 2	0.002	-0.069	0.073	0.479	46
			Lag 3	0.054	-0.035	0.142	0.118	44
		Lag 3	Lag 0	0.036	-0.052	0.125	0.212	43
			Lag 1	0.018	-0.020	0.056	0.176	30
			Lag 2	-0.033	-0.130	0.064	0.251	33
			Lag 3	0.028	-0.073	0.130	0.293	31
Female	All-natural	Lag 0	Lag 0	0.001	-0.001	0.004	0.203	34010
			Lag 1	0.001	-0.002	0.004	0.289	31215
			Lag 2	-0.006	-0.009	-0.002	**0.001**	30865

		Lag 3	0.000	-0.003	0.003	0.484	30480
	Lag 1	Lag 0	0.002	-0.001	0.004	0.098	32905
		Lag 1	-0.001	-0.004	0.001	0.205	33417
		Lag 2	-0.004	-0.007	-0.002	**0.001**	33523
		Lag 3	-0.001	-0.004	0.003	0.314	33483
	Lag 2	Lag 0	0.002	-0.001	0.004	0.088	29774
		Lag 1	-0.001	-0.004	0.001	0.099	32884
		Lag 2	-0.007	-0.009	-0.004	**0.000**	33052
		Lag 3	-0.003	-0.005	0.000	**0.020**	33075
	Lag 3	Lag 0	0.002	-0.001	0.005	0.053	30912
		Lag 1	0.001	-0.001	0.004	0.139	30692
		Lag 2	-0.004	-0.007	-0.002	**0.000**	31006
		Lag 3	-0.002	-0.004	0.001	0.095	31233
Cardiovascular	Lag 0	Lag 0	0.042	-0.017	0.102	0.081	56
		Lag 1	-0.014	-0.088	0.060	0.357	52
		Lag 2	0.019	-0.066	0.104	0.331	51
		Lag 3	-0.098	-0.229	0.032	0.070	51
	Lag 1	Lag 0	0.044	-0.009	0.098	0.052	54
		Lag 1	-0.014	-0.078	0.051	0.338	53
		Lag 2	0.018	-0.046	0.082	0.291	53
		Lag 3	0.029	-0.053	0.112	0.245	53
	Lag 2	Lag 0	0.077	0.023	0.131	**0.003**	55
		Lag 1	-0.047	-0.116	0.022	0.090	55
		Lag 2	-0.011	-0.072	0.050	0.362	55
		Lag 3	-0.024	-0.097	0.049	0.258	55
	Lag 3	Lag 0	0.098	0.029	0.167	**0.003**	63
		Lag 1	-0.031	-0.098	0.035	0.176	52
		Lag 2	-0.009	-0.070	0.052	0.385	53
		Lag 3	-0.034	-0.109	0.041	0.185	53
Cerebrovascular	Lag 0	Lag 0	0.011	-0.035	0.057	0.316	89
		Lag 1	0.010	-0.031	0.051	0.315	87
		Lag 2	-0.021	-0.093	0.052	0.289	90
		Lag 3	0.044	-0.016	0.105	0.076	91
	Lag 1	Lag 0	0.019	-0.026	0.064	0.201	95
		Lag 1	0.016	-0.021	0.052	0.203	110
		Lag 2	0.019	-0.033	0.071	0.235	116
		Lag 3	0.033	-0.028	0.094	0.142	117
	Lag 2	Lag 0	0.039	-0.006	0.084	**0.043**	85
		Lag 1	0.015	-0.019	0.048	0.194	98
		Lag 2	-0.012	-0.065	0.042	0.336	102
		Lag 3	0.045	-0.002	0.092	**0.031**	102
	Lag 3	Lag 0	0.066	0.010	0.122	**0.010**	86
		Lag 1	0.011	-0.030	0.052	0.301	76
		Lag 2	-0.015	-0.066	0.036	0.285	79
		Lag 3	0.037	-0.005	0.080	**0.043**	76
Renal	Lag 0	Lag 0	0.028	-0.044	0.100	0.221	39
		Lag 1	-0.018	-0.118	0.082	0.364	28

			Lag 2	0.001	-0.119	0.121	0.494	28
			Lag 3	0.093	-0.008	0.195	**0.036**	29
		Lag 1	Lag 0	0.034	-0.028	0.096	0.139	38
			Lag 1	-0.009	-0.101	0.083	0.425	31
			Lag 2	-0.019	-0.125	0.086	0.360	34
			Lag 3	0.048	-0.054	0.149	0.178	36
		Lag 2	Lag 0	0.015	-0.076	0.105	0.376	33
			Lag 1	0.018	-0.078	0.113	0.357	32
			Lag 2	0.023	-0.065	0.111	0.304	34
			Lag 3	0.067	-0.006	0.141	**0.036**	38
		Lag 3	Lag 0	0.000	-0.103	0.102	0.498	33
			Lag 1	-0.020	-0.140	0.100	0.371	29
			Lag 2	0.003	-0.067	0.072	0.467	32
			Lag 3	0.014	-0.049	0.077	0.330	32
	Respiratory	Lag 0	Lag 0	0.010	-0.076	0.095	0.411	46
			Lag 1	-0.019	-0.114	0.075	0.343	36
			Lag 2	-0.067	-0.179	0.046	0.122	36
			Lag 3	0.023	-0.060	0.106	0.291	35
		Lag 1	Lag 0	0.037	-0.039	0.113	0.171	49
			Lag 1	0.021	-0.051	0.093	0.283	38
			Lag 2	-0.034	-0.129	0.061	0.241	38
			Lag 3	0.047	-0.037	0.131	0.136	37
		Lag 2	Lag 0	0.063	-0.005	0.131	**0.035**	54
			Lag 1	0.044	-0.005	0.092	**0.038**	48
			Lag 2	-0.011	-0.093	0.072	0.401	49
			Lag 3	0.036	-0.039	0.111	0.176	47
		Lag 3	Lag 0	-0.006	-0.101	0.088	0.450	56
			Lag 1	0.008	-0.057	0.074	0.401	35
			Lag 2	-0.012	-0.085	0.061	0.375	39
			Lag 3	0.035	-0.026	0.096	0.132	39
Age 18-49 year	All-natural	Lag 0	Lag 0	0.001	-0.002	0.004	0.233	21442
			Lag 1	-0.002	-0.006	0.002	0.133	19677
			Lag 2	-0.008	-0.012	-0.003	**0.000**	19427
			Lag 3	0.000	-0.005	0.004	0.424	19164
		Lag 1	Lag 0	0.001	-0.002	0.004	0.173	20621
			Lag 1	-0.004	-0.008	-0.001	**0.004**	20970
			Lag 2	-0.009	-0.012	-0.005	**0.000**	21004
			Lag 3	0.000	-0.004	0.004	0.477	20948
		Lag 2	Lag 0	0.002	-0.001	0.005	0.103	18757
			Lag 1	-0.002	-0.005	0.001	0.070	20318
			Lag 2	-0.009	-0.012	-0.006	**0.000**	20397
			Lag 3	0.000	-0.003	0.004	0.418	20392
		Lag 3	Lag 0	0.002	-0.002	0.006	0.145	19793
			Lag 1	0.003	0.000	0.006	**0.045**	19032
			Lag 2	-0.007	-0.010	-0.004	**0.000**	19163
			Lag 3	0.000	-0.003	0.003	0.484	19285
	Cardiovascular	Lag 0	Lag 0	-0.059	-0.212	0.094	0.226	14

		Lag 1	-0.165	-0.428	0.098	0.109	11
		Lag 2	-0.093	-0.304	0.118	0.195	10
		Lag 3	-0.137	-0.375	0.101	0.129	10
	Lag 1	Lag 0	-0.030	-0.143	0.084	0.304	17
		Lag 1	-0.031	-0.139	0.078	0.289	14
		Lag 2	-0.085	-0.254	0.084	0.163	13
		Lag 3	-0.131	-0.338	0.076	0.108	14
	Lag 2	Lag 0	-0.015	-0.126	0.096	0.395	18
		Lag 1	-0.035	-0.124	0.055	0.225	16
		Lag 2	-0.070	-0.235	0.095	0.203	17
		Lag 3	-0.164	-0.395	0.067	0.082	17
	Lag 3	Lag 0	-0.025	-0.179	0.130	0.376	16
		Lag 1	-0.050	-0.161	0.061	0.188	13
		Lag 2	-0.084	-0.242	0.074	0.148	13
		Lag 3	-0.054	-0.190	0.081	0.216	14
Cerebrovascular	Lag 0	Lag 0	0.042	-0.046	0.130	0.177	16
		Lag 1	0.036	-0.073	0.144	0.259	16
		Lag 2	0.109	0.000	0.219	**0.025**	16
		Lag 3	0.058	-0.058	0.175	0.164	16
	Lag 1	Lag 0	0.053	-0.038	0.144	0.128	18
		Lag 1	0.045	-0.052	0.141	0.184	21
		Lag 2	0.005	-0.110	0.120	0.466	22
		Lag 3	-0.067	-0.250	0.116	0.236	22
	Lag 2	Lag 0	0.066	-0.047	0.179	0.127	16
		Lag 1	0.016	-0.081	0.113	0.372	22
		Lag 2	-0.030	-0.147	0.087	0.308	24
		Lag 3	-0.003	-0.128	0.122	0.481	22
	Lag 3	Lag 0	-0.096	-0.341	0.149	0.221	10
		Lag 1	-0.062	-0.199	0.075	0.188	13
		Lag 2	0.011	-0.113	0.134	0.433	14
		Lag 3	-0.029	-0.166	0.107	0.336	11
Renal	Lag 0	Lag 0	-	-	-	-	< 10
		Lag 1	-	-	-	-	< 10
		Lag 2	-	-	-	-	< 10
		Lag 3	-	-	-	-	< 10
	Lag 1	Lag 0	-	-	-	-	< 10
		Lag 1	-	-	-	-	< 10
		Lag 2	-	-	-	-	< 10
		Lag 3	-	-	-	-	< 10
	Lag 2	Lag 0	-	-	-	-	< 10
		Lag 1	-	-	-	-	< 10
		Lag 2	-	-	-	-	< 10
		Lag 3	-	-	-	-	< 10
	Lag 3	Lag 0	-0.040	-0.200	0.120	0.313	10
		Lag 1	-	-	-	-	< 10
		Lag 2	-	-	-	-	< 10
		Lag 3	-	-	-	-	< 10

	Respiratory	Lag 0	Lag 0	0.100	-0.040	0.240	0.080	18
			Lag 1	0.013	-0.148	0.174	0.438	12
			Lag 2	0.032	-0.197	0.260	0.393	12
			Lag 3	0.111	-0.034	0.257	0.067	11
		Lag 1	Lag 0	0.050	-0.118	0.217	0.280	18
			Lag 1	0.067	-0.070	0.203	0.170	16
			Lag 2	0.034	-0.114	0.182	0.325	16
			Lag 3	0.129	-0.011	0.270	**0.035**	16
		Lag 2	Lag 0	0.146	0.033	0.259	**0.006**	24
			Lag 1	0.000	-0.110	0.110	0.499	15
			Lag 2	0.020	-0.139	0.179	0.403	16
			Lag 3	-0.018	-0.171	0.136	0.410	16
		Lag 3	Lag 0	-0.040	-0.205	0.125	0.316	20
			Lag 1	-	-	-	-	< 10
			Lag 2	-0.012	-0.139	0.115	0.427	10
			Lag 3	-0.024	-0.183	0.134	0.382	10
Age 50-64 year	All-natural	Lag 0	Lag 0	0.001	-0.003	0.005	0.319	14664
			Lag 1	-0.002	-0.006	0.003	0.218	13520
			Lag 2	-0.001	-0.006	0.005	0.420	13405
			Lag 3	0.002	-0.003	0.007	0.208	13277
		Lag 1	Lag 0	0.002	-0.001	0.006	0.092	14349
			Lag 1	-0.003	-0.007	0.001	0.064	14309
			Lag 2	0.000	-0.005	0.004	0.409	14351
			Lag 3	0.002	-0.003	0.007	0.218	14369
		Lag 2	Lag 0	0.001	-0.003	0.005	0.314	12914
			Lag 1	-0.006	-0.010	-0.003	**0.000**	14183
			Lag 2	-0.007	-0.010	-0.003	**0.000**	14260
			Lag 3	-0.002	-0.006	0.002	0.172	14293
		Lag 3	Lag 0	0.002	-0.003	0.007	0.193	13140
			Lag 1	-0.002	-0.006	0.002	0.176	13361
			Lag 2	-0.004	-0.007	0.000	**0.013**	13545
			Lag 3	-0.001	-0.005	0.002	0.243	13639
	Cardiovascular	Lag 0	Lag 0	-0.012	-0.100	0.077	0.398	23
			Lag 1	0.032	-0.057	0.121	0.240	36
			Lag 2	0.024	-0.084	0.132	0.331	34
			Lag 3	-0.040	-0.157	0.076	0.248	33
		Lag 1	Lag 0	-0.043	-0.143	0.057	0.198	26
			Lag 1	0.020	-0.068	0.108	0.327	29
			Lag 2	0.038	-0.041	0.117	0.176	29
			Lag 3	-0.038	-0.143	0.067	0.240	29
		Lag 2	Lag 0	0.023	-0.070	0.116	0.315	24
			Lag 1	-0.032	-0.116	0.052	0.226	32
			Lag 2	-0.004	-0.083	0.076	0.465	31
			Lag 3	-0.034	-0.121	0.053	0.222	31
		Lag 3	Lag 0	0.062	-0.042	0.165	0.122	37
			Lag 1	-0.046	-0.149	0.056	0.188	28
			Lag 2	-0.020	-0.093	0.053	0.295	28

		Lag 3	-0.015	-0.095	0.064	0.353	29
Cerebrovascular	Lag 0	Lag 0	-0.037	-0.125	0.051	0.206	42
		Lag 1	-0.001	-0.076	0.074	0.486	47
		Lag 2	0.025	-0.067	0.116	0.299	48
		Lag 3	0.065	-0.010	0.140	**0.046**	47
	Lag 1	Lag 0	-0.016	-0.096	0.063	0.344	49
		Lag 1	-0.038	-0.116	0.041	0.174	45
		Lag 2	-0.027	-0.108	0.055	0.260	45
		Lag 3	0.066	-0.006	0.138	**0.036**	47
	Lag 2	Lag 0	0.020	-0.054	0.093	0.300	41
		Lag 1	-0.009	-0.063	0.044	0.364	52
		Lag 2	0.017	-0.039	0.072	0.280	51
		Lag 3	0.043	-0.010	0.096	0.056	53
	Lag 3	Lag 0	0.062	-0.007	0.132	**0.040**	55
		Lag 1	-0.014	-0.075	0.047	0.327	52
		Lag 2	0.010	-0.033	0.053	0.323	53
		Lag 3	0.030	-0.019	0.078	0.116	54
Renal	Lag 0	Lag 0	0.042	-0.061	0.145	0.213	16
		Lag 1	-0.047	-0.196	0.102	0.266	16
		Lag 2	0.111	-0.043	0.266	0.079	15
		Lag 3	0.155	-0.016	0.326	**0.038**	15
	Lag 1	Lag 0	0.031	-0.055	0.116	0.242	11
		Lag 1	0.047	-0.049	0.142	0.168	19
		Lag 2	0.119	-0.031	0.268	0.060	20
		Lag 3	0.055	-0.107	0.216	0.254	21
	Lag 2	Lag 0	-	-	-	-	< 10
		Lag 1	0.064	-0.033	0.161	0.098	20
		Lag 2	-0.024	-0.184	0.137	0.385	21
		Lag 3	0.027	-0.137	0.191	0.375	22
	Lag 3	Lag 0	-	-	-	-	< 10
		Lag 1	0.037	-0.113	0.187	0.315	15
		Lag 2	-0.057	-0.222	0.109	0.251	17
		Lag 3	0.009	-0.143	0.160	0.455	17
Respiratory	Lag 0	Lag 0	0.002	-0.091	0.096	0.481	30
		Lag 1	0.112	0.017	0.208	**0.011**	31
		Lag 2	-0.016	-0.120	0.089	0.384	30
		Lag 3	0.117	0.014	0.221	**0.013**	30
	Lag 1	Lag 0	0.006	-0.084	0.096	0.446	28
		Lag 1	0.052	-0.042	0.146	0.138	26
		Lag 2	-0.049	-0.154	0.057	0.181	26
		Lag 3	0.010	-0.112	0.132	0.439	26
	Lag 2	Lag 0	0.071	-0.005	0.146	**0.034**	35
		Lag 1	0.046	-0.033	0.124	0.128	35
		Lag 2	-0.010	-0.104	0.084	0.419	36
		Lag 3	0.055	-0.042	0.151	0.134	37
	Lag 3	Lag 0	0.025	-0.076	0.125	0.316	34
		Lag 1	0.002	-0.097	0.101	0.484	23

			Lag 2	-0.070	-0.204	0.063	0.152	24
			Lag 3	-0.011	-0.110	0.088	0.410	25
Age 65+ year	All-natural	Lag 0	Lag 0	0.003	0.000	0.006	**0.027**	23923
			Lag 1	0.003	0.000	0.007	**0.016**	21739
			Lag 2	-0.002	-0.006	0.002	0.208	21503
			Lag 3	0.001	-0.002	0.005	0.236	21337
		Lag 1	Lag 0	0.005	0.002	0.007	**0.000**	23651
			Lag 1	0.002	-0.001	0.005	0.076	23546
			Lag 2	0.000	-0.003	0.003	0.478	23609
			Lag 3	0.000	-0.004	0.004	0.499	23652
		Lag 2	Lag 0	0.006	0.003	0.009	**0.000**	21151
			Lag 1	0.001	-0.001	0.004	0.163	23829
			Lag 2	-0.001	-0.004	0.002	0.282	23961
			Lag 3	-0.004	-0.007	-0.001	**0.004**	24095
		Lag 3	Lag 0	0.008	0.005	0.011	**0.000**	21294
			Lag 1	0.002	0.000	0.005	**0.039**	21721
			Lag 2	-0.002	-0.004	0.001	0.082	21982
			Lag 3	-0.002	-0.005	0.001	0.101	22245
	Cardiovascular	Lag 0	Lag 0	0.035	-0.012	0.082	0.072	74
			Lag 1	0.025	-0.021	0.070	0.142	68
			Lag 2	0.025	-0.050	0.100	0.257	66
			Lag 3	-0.014	-0.089	0.060	0.354	64
		Lag 1	Lag 0	0.057	0.011	0.104	**0.008**	68
			Lag 1	0.011	-0.033	0.055	0.315	72
			Lag 2	0.041	-0.011	0.094	0.062	70
			Lag 3	0.037	-0.034	0.107	0.153	68
		Lag 2	Lag 0	0.074	0.023	0.125	**0.002**	71
			Lag 1	-0.001	-0.041	0.039	0.481	78
			Lag 2	0.003	-0.047	0.053	0.453	74
			Lag 3	0.010	-0.040	0.061	0.343	70
		Lag 3	Lag 0	0.067	0.010	0.125	**0.011**	79
			Lag 1	0.014	-0.024	0.051	0.234	71
			Lag 2	-0.007	-0.060	0.046	0.397	66
			Lag 3	0.015	-0.033	0.064	0.266	61
	Cerebrovascular	Lag 0	Lag 0	0.001	-0.038	0.041	0.473	125
			Lag 1	-0.005	-0.044	0.035	0.408	116
			Lag 2	-0.011	-0.073	0.051	0.364	117
			Lag 3	0.003	-0.060	0.066	0.462	121
		Lag 1	Lag 0	0.012	-0.027	0.050	0.275	127
			Lag 1	0.012	-0.020	0.045	0.227	143
			Lag 2	0.012	-0.033	0.057	0.301	148
			Lag 3	-0.011	-0.072	0.049	0.359	149
		Lag 2	Lag 0	-0.002	-0.049	0.045	0.466	103
			Lag 1	0.016	-0.013	0.045	0.145	130
			Lag 2	-0.015	-0.056	0.026	0.242	132
			Lag 3	0.002	-0.044	0.047	0.473	135
		Lag 3	Lag 0	0.023	-0.027	0.072	0.187	108

			Lag 1	0.029	-0.003	0.061	**0.040**	113
			Lag 2	0.011	-0.021	0.043	0.255	115
			Lag 3	0.020	-0.016	0.056	0.141	115
	Renal	Lag 0	Lag 0	0.030	-0.030	0.090	0.161	48
			Lag 1	0.044	-0.041	0.130	0.156	42
			Lag 2	0.029	-0.057	0.115	0.255	41
			Lag 3	0.002	-0.121	0.124	0.489	42
		Lag 1	Lag 0	0.065	0.005	0.125	**0.016**	51
			Lag 1	-0.020	-0.114	0.075	0.341	43
			Lag 2	-0.056	-0.160	0.048	0.146	45
			Lag 3	0.050	-0.050	0.150	0.162	46
		Lag 2	Lag 0	0.052	-0.017	0.121	0.070	34
			Lag 1	-0.017	-0.131	0.097	0.386	31
			Lag 2	0.005	-0.073	0.083	0.452	33
			Lag 3	0.047	-0.026	0.119	0.104	36
		Lag 3	Lag 0	-0.024	-0.135	0.087	0.334	37
			Lag 1	-0.020	-0.140	0.100	0.371	34
			Lag 2	0.019	-0.047	0.084	0.288	35
			Lag 3	0.011	-0.047	0.069	0.355	34
	Respiratory	Lag 0	Lag 0	-0.022	-0.124	0.081	0.338	37
			Lag 1	0.002	-0.081	0.085	0.481	33
			Lag 2	-0.001	-0.102	0.101	0.496	34
			Lag 3	0.059	-0.020	0.137	0.070	33
		Lag 1	Lag 0	-0.042	-0.150	0.066	0.223	32
			Lag 1	0.028	-0.038	0.095	0.200	39
			Lag 2	-0.001	-0.079	0.077	0.492	39
			Lag 3	0.103	0.023	0.183	**0.006**	37
		Lag 2	Lag 0	-0.007	-0.099	0.085	0.441	37
			Lag 1	0.047	0.000	0.095	**0.026**	41
			Lag 2	-0.008	-0.083	0.067	0.416	43
			Lag 3	0.056	-0.026	0.139	0.090	38
		Lag 3	Lag 0	0.005	-0.093	0.102	0.463	45
			Lag 1	0.042	-0.022	0.106	0.098	33
			Lag 2	0.000	-0.074	0.075	0.497	38
			Lag 3	0.068	0.000	0.136	**0.026**	35
English, preferred language	All-natural	Lag 0	Lag 0	0.002	0.000	0.004	0.064	51173
			Lag 1	-0.001	-0.003	0.002	0.257	46427
			Lag 2	-0.005	-0.008	-0.002	**0.001**	45897
			Lag 3	0.001	-0.002	0.004	0.192	45400
		Lag 1	Lag 0	0.003	0.001	0.005	**0.001**	50170
			Lag 1	-0.003	-0.005	-0.001	**0.005**	50102
			Lag 2	-0.004	-0.006	-0.002	**0.000**	50221
			Lag 3	0.000	-0.002	0.003	0.422	50217
		Lag 2	Lag 0	0.004	0.002	0.006	**0.000**	45298
			Lag 1	-0.002	-0.004	-0.001	**0.005**	49702
			Lag 2	-0.006	-0.008	-0.004	**0.000**	49944
			Lag 3	-0.002	-0.005	0.000	**0.013**	50088

	Lag 3	Lag 0	0.004	0.002	0.006	**0.000**	46350
		Lag 1	0.002	0.000	0.003	0.061	46285
		Lag 2	-0.005	-0.006	-0.003	**0.000**	46775
		Lag 3	-0.001	-0.003	0.001	0.283	47171
Cardiovascular	Lag 0	Lag 0	0.017	-0.022	0.057	0.198	97
		Lag 1	0.009	-0.032	0.050	0.335	100
		Lag 2	0.014	-0.050	0.078	0.337	96
		Lag 3	-0.040	-0.108	0.028	0.125	94
	Lag 1	Lag 0	0.021	-0.020	0.062	0.159	94
		Lag 1	0.000	-0.041	0.042	0.492	99
		Lag 2	0.023	-0.026	0.072	0.176	96
		Lag 3	-0.001	-0.062	0.060	0.482	96
	Lag 2	Lag 0	0.050	0.006	0.093	**0.013**	97
		Lag 1	-0.016	-0.053	0.022	0.208	111
		Lag 2	-0.015	-0.061	0.032	0.267	107
		Lag 3	-0.020	-0.067	0.028	0.209	104
	Lag 3	Lag 0	0.053	0.000	0.107	**0.026**	114
		Lag 1	-0.008	-0.045	0.029	0.335	95
		Lag 2	-0.028	-0.074	0.019	0.120	90
		Lag 3	-0.008	-0.051	0.036	0.365	88
Cerebrovascular	Lag 0	Lag 0	-0.004	-0.043	0.034	0.417	143
		Lag 1	0.012	-0.023	0.046	0.249	146
		Lag 2	0.014	-0.038	0.066	0.296	147
		Lag 3	0.010	-0.047	0.066	0.367	149
	Lag 1	Lag 0	0.017	-0.020	0.054	0.185	155
		Lag 1	0.018	-0.012	0.048	0.122	172
		Lag 2	0.013	-0.028	0.053	0.268	177
		Lag 3	-0.002	-0.056	0.052	0.472	178
	Lag 2	Lag 0	0.011	-0.031	0.053	0.304	128
		Lag 1	0.021	-0.005	0.048	0.056	167
		Lag 2	0.007	-0.026	0.040	0.344	170
		Lag 3	0.009	-0.029	0.048	0.317	171
	Lag 3	Lag 0	0.019	-0.029	0.066	0.221	136
		Lag 1	0.021	-0.008	0.050	0.081	150
		Lag 2	0.002	-0.029	0.033	0.451	152
		Lag 3	0.010	-0.024	0.044	0.278	149
Renal	Lag 0	Lag 0	0.026	-0.025	0.077	0.156	61
		Lag 1	-0.004	-0.074	0.066	0.454	56
		Lag 2	0.049	-0.028	0.125	0.106	54
		Lag 3	0.073	-0.016	0.163	0.055	55
	Lag 1	Lag 0	0.039	-0.009	0.086	0.055	55
		Lag 1	-0.029	-0.103	0.045	0.223	55
		Lag 2	-0.030	-0.117	0.057	0.247	58
		Lag 3	0.069	-0.012	0.150	**0.047**	60
	Lag 2	Lag 0	-0.009	-0.073	0.055	0.394	41
		Lag 1	-0.023	-0.099	0.053	0.276	48
		Lag 2	0.020	-0.047	0.086	0.282	50

			Lag 3	0.070	0.011	0.129	**0.010**	54
		Lag 3	Lag 0	-0.068	-0.161	0.026	0.079	48
			Lag 1	-0.021	-0.114	0.071	0.326	47
			Lag 2	-0.004	-0.066	0.058	0.448	50
			Lag 3	0.031	-0.020	0.081	0.118	49
	Respiratory	Lag 0	Lag 0	0.015	-0.052	0.082	0.335	75
			Lag 1	0.050	-0.008	0.107	**0.046**	68
			Lag 2	-0.001	-0.076	0.073	0.486	68
			Lag 3	0.125	0.061	0.188	**0.000**	66
		Lag 1	Lag 0	-0.033	-0.110	0.045	0.204	66
			Lag 1	0.036	-0.018	0.091	0.096	71
			Lag 2	-0.008	-0.071	0.054	0.395	71
			Lag 3	0.087	0.022	0.151	**0.004**	69
		Lag 2	Lag 0	0.052	-0.004	0.107	**0.035**	82
			Lag 1	0.045	0.004	0.086	**0.017**	80
			Lag 2	0.004	-0.053	0.060	0.448	84
			Lag 3	0.038	-0.022	0.099	0.107	80
		Lag 3	Lag 0	-0.002	-0.070	0.067	0.481	82
			Lag 1	0.021	-0.015	0.058	0.125	60
			Lag 2	-0.022	-0.083	0.040	0.244	67
			Lag 3	0.024	-0.031	0.078	0.200	65
Spanish, preferred language	All-natural	Lag 0	Lag 0	-0.001	-0.007	0.005	0.404	6227
			Lag 1	-0.001	-0.007	0.006	0.413	6083
			Lag 2	-0.001	-0.008	0.006	0.373	6037
			Lag 3	-0.003	-0.009	0.004	0.225	6006
		Lag 1	Lag 0	-0.001	-0.007	0.005	0.345	5960
			Lag 1	-0.003	-0.009	0.003	0.191	6011
			Lag 2	-0.003	-0.009	0.003	0.168	6027
			Lag 3	0.000	-0.006	0.007	0.458	6040
		Lag 2	Lag 0	-0.005	-0.011	0.002	0.086	5296
			Lag 1	-0.002	-0.008	0.004	0.244	5983
			Lag 2	-0.005	-0.011	0.001	**0.046**	6018
			Lag 3	0.000	-0.006	0.006	0.477	6030
		Lag 3	Lag 0	0.008	0.000	0.015	**0.022**	5701
			Lag 1	0.002	-0.005	0.008	0.296	5518
			Lag 2	-0.005	-0.011	0.001	**0.038**	5559
			Lag 3	-0.005	-0.011	0.001	0.060	5605
	Cardiovascular	Lag 0	Lag 0	0.011	-0.183	0.204	0.457	10
			Lag 1	-	-	-	-	< 10
			Lag 2	-	-	-	-	< 10
			Lag 3	-	-	-	-	< 10
		Lag 1	Lag 0	-0.017	-0.199	0.166	0.428	13
			Lag 1	0.045	-0.089	0.178	0.255	10
			Lag 2	0.012	-0.108	0.132	0.424	10
			Lag 3	-	-	-	-	< 10
		Lag 2	Lag 0	-0.045	-0.256	0.167	0.339	11
			Lag 1	-	-	-	-	< 10

			Lag 2	-	-	-	-	< 10
			Lag 3	-	-	-	-	< 10
		Lag 3	Lag 0	0.040	-0.166	0.245	0.353	14
			Lag 1	-	-	-	-	< 10
			Lag 2	-	-	-	-	< 10
			Lag 3	-	-	-	-	< 10
	Cerebrovascular	Lag 0	Lag 0	-0.052	-0.181	0.078	0.217	21
			Lag 1	-0.069	-0.202	0.065	0.156	24
			Lag 2	0.018	-0.109	0.144	0.391	25
			Lag 3	0.078	-0.004	0.160	**0.032**	25
		Lag 1	Lag 0	0.027	-0.071	0.124	0.296	22
			Lag 1	-0.033	-0.151	0.085	0.293	22
			Lag 2	-0.041	-0.178	0.096	0.279	23
			Lag 3	0.079	-0.007	0.165	**0.036**	24
		Lag 2	Lag 0	0.033	-0.091	0.157	0.303	22
			Lag 1	-0.043	-0.149	0.063	0.215	24
			Lag 2	-0.074	-0.206	0.058	0.136	25
			Lag 3	0.065	-0.015	0.144	0.055	26
		Lag 3	Lag 0	0.107	-0.015	0.229	**0.043**	25
			Lag 1	-0.045	-0.160	0.071	0.224	18
			Lag 2	-0.006	-0.073	0.061	0.430	20
			Lag 3	0.040	-0.036	0.117	0.151	20
	Renal	Lag 0	Lag 0	-	-	-	-	< 10
			Lag 1	-	-	-	-	< 10
			Lag 2	-	-	-	-	< 10
			Lag 3	-	-	-	-	< 10
		Lag 1	Lag 0	-	-	-	-	< 10
			Lag 1	-	-	-	-	< 10
			Lag 2	-	-	-	-	< 10
			Lag 3	-	-	-	-	< 10
		Lag 2	Lag 0	-	-	-	-	< 10
			Lag 1	-	-	-	-	< 10
			Lag 2	-	-	-	-	< 10
			Lag 3	-	-	-	-	< 10
		Lag 3	Lag 0	-	-	-	-	< 10
			Lag 1	-	-	-	-	< 10
			Lag 2	-	-	-	-	< 10
			Lag 3	-	-	-	-	< 10
	Respiratory	Lag 0	Lag 0	-	-	-	-	< 10
			Lag 1	-	-	-	-	< 10
			Lag 2	-	-	-	-	< 10
			Lag 3	-	-	-	-	< 10
		Lag 1	Lag 0	-	-	-	-	< 10
			Lag 1	-	-	-	-	< 10
			Lag 2	-	-	-	-	< 10
			Lag 3	-	-	-	-	< 10
		Lag 2	Lag 0	-	-	-	-	< 10

			Lag 1	-	-	-	-	< 10
			Lag 2	-	-	-	-	< 10
			Lag 3	-	-	-	-	< 10
		Lag 3	Lag 0	0.094	-0.111	0.299	0.185	11
			Lag 1	-	-	-	-	< 10
			Lag 2	-	-	-	-	< 10
			Lag 3	-	-	-	-	< 10
Other, preferred language	All-natural	Lag 0	Lag 0	0.007	0.000	0.014	**0.018**	2629
			Lag 1	0.014	0.007	0.022	**0.000**	2426
			Lag 2	0.009	-0.003	0.020	0.066	2401
			Lag 3	0.004	-0.005	0.014	0.186	2372
		Lag 1	Lag 0	0.009	0.003	0.015	**0.002**	2491
			Lag 1	0.016	0.009	0.022	**0.000**	2712
			Lag 2	0.010	0.003	0.018	**0.004**	2716
			Lag 3	0.005	-0.006	0.016	0.209	2712
		Lag 2	Lag 0	0.011	0.004	0.018	**0.001**	2228
			Lag 1	0.008	0.002	0.013	**0.004**	2645
			Lag 2	0.009	0.002	0.015	**0.004**	2656
			Lag 3	-0.001	-0.008	0.007	0.418	2662
		Lag 3	Lag 0	0.010	0.002	0.018	**0.007**	2176
			Lag 1	0.002	-0.005	0.008	0.330	2311
			Lag 2	0.003	-0.003	0.008	0.196	2356
			Lag 3	-0.003	-0.010	0.004	0.215	2393
	Cardiovascular	Lag 0	Lag 0	-	-	-	-	< 10
			Lag 1	-	-	-	-	< 10
			Lag 2	-	-	-	-	< 10
			Lag 3	-	-	-	-	< 10
		Lag 1	Lag 0	-	-	-	-	< 10
			Lag 1	-	-	-	-	< 10
			Lag 2	-	-	-	-	< 10
			Lag 3	-	-	-	-	< 10
		Lag 2	Lag 0	-	-	-	-	< 10
			Lag 1	-	-	-	-	< 10
			Lag 2	-	-	-	-	< 10
			Lag 3	-	-	-	-	< 10
		Lag 3	Lag 0	-	-	-	-	< 10
			Lag 1	-	-	-	-	< 10
			Lag 2	-	-	-	-	< 10
			Lag 3	-	-	-	-	< 10
	Cerebrovascular	Lag 0	Lag 0	0.045	-0.032	0.121	0.126	19
			Lag 1	-	-	-	-	< 10
			Lag 2	-	-	-	-	< 10
			Lag 3	-0.013	-0.193	0.167	0.444	10
		Lag 1	Lag 0	-0.040	-0.152	0.073	0.244	17
			Lag 1	-0.077	-0.203	0.050	0.117	15
			Lag 2	-0.063	-0.224	0.098	0.220	15
			Lag 3	-0.157	-0.426	0.112	0.127	16

		Lag 2	Lag 0	-0.014	-0.121	0.093	0.398	10
			Lag 1	-0.059	-0.174	0.056	0.158	13
			Lag 2	-0.130	-0.324	0.064	0.095	12
			Lag 3	-0.021	-0.146	0.104	0.370	13
		Lag 3	Lag 0	0.031	-0.058	0.121	0.246	12
			Lag 1	-0.027	-0.131	0.077	0.306	10
			Lag 2	0.048	-0.012	0.108	0.057	10
			Lag 3	0.057	-0.019	0.132	0.070	11
	Renal	Lag 0	Lag 0	-	-	-	-	< 10
			Lag 1	-	-	-	-	< 10
			Lag 2	-	-	-	-	< 10
			Lag 3	-	-	-	-	< 10
		Lag 1	Lag 0	-	-	-	-	< 10
			Lag 1	-	-	-	-	< 10
			Lag 2	-	-	-	-	< 10
			Lag 3	-	-	-	-	< 10
		Lag 2	Lag 0	-	-	-	-	< 10
			Lag 1	-	-	-	-	< 10
			Lag 2	-	-	-	-	< 10
			Lag 3	-	-	-	-	< 10
		Lag 3	Lag 0	-	-	-	-	< 10
			Lag 1	-	-	-	-	< 10
			Lag 2	-	-	-	-	< 10
			Lag 3	-	-	-	-	< 10
	Respiratory	Lag 0	Lag 0	-	-	-	-	< 10
			Lag 1	-	-	-	-	< 10
			Lag 2	-	-	-	-	< 10
			Lag 3	-	-	-	-	< 10
		Lag 1	Lag 0	-	-	-	-	< 10
			Lag 1	-	-	-	-	< 10
			Lag 2	-	-	-	-	< 10
			Lag 3	-	-	-	-	< 10
		Lag 2	Lag 0	-	-	-	-	< 10
			Lag 1	-	-	-	-	< 10
			Lag 2	-	-	-	-	< 10
			Lag 3	-	-	-	-	< 10
		Lag 3	Lag 0	-	-	-	-	< 10
			Lag 1	-	-	-	-	< 10
			Lag 2	-	-	-	-	< 10
			Lag 3	-	-	-	-	< 10
White	All-natural	Lag 0	Lag 0	0.002	-0.001	0.004	0.141	29020
			Lag 1	0.000	-0.003	0.003	0.445	25632
			Lag 2	-0.006	-0.010	-0.002	**0.002**	25284
			Lag 3	0.002	-0.002	0.006	0.220	24938
		Lag 1	Lag 0	0.002	0.000	0.005	**0.041**	28772
			Lag 1	-0.004	-0.007	-0.001	**0.001**	28242
			Lag 2	-0.005	-0.009	-0.002	**0.001**	28284

		Lag 3	-0.001	-0.005	0.003	0.281	28256
	Lag 2	Lag 0	0.002	-0.001	0.005	0.068	26330
		Lag 1	-0.004	-0.006	-0.001	**0.001**	28421
		Lag 2	-0.007	-0.010	-0.005	**0.000**	28582
		Lag 3	-0.004	-0.007	-0.001	**0.009**	28650
	Lag 3	Lag 0	0.004	0.001	0.007	**0.005**	26605
		Lag 1	0.001	-0.002	0.003	0.257	27009
		Lag 2	-0.005	-0.008	-0.003	**0.000**	27290
		Lag 3	-0.001	-0.003	0.002	0.302	27472
Cardiovascular	Lag 0	Lag 0	0.004	-0.057	0.064	0.452	62
		Lag 1	0.000	-0.064	0.064	0.500	70
		Lag 2	0.049	-0.032	0.130	0.118	66
		Lag 3	0.005	-0.067	0.077	0.446	65
	Lag 1	Lag 0	-0.001	-0.062	0.059	0.483	61
		Lag 1	-0.056	-0.134	0.022	0.080	66
		Lag 2	0.015	-0.057	0.088	0.339	64
		Lag 3	0.004	-0.074	0.082	0.455	63
	Lag 2	Lag 0	0.039	-0.024	0.101	0.112	67
		Lag 1	-0.015	-0.059	0.029	0.254	78
		Lag 2	-0.010	-0.069	0.048	0.365	75
		Lag 3	-0.032	-0.093	0.029	0.154	73
	Lag 3	Lag 0	0.077	0.011	0.143	**0.012**	80
		Lag 1	-0.006	-0.046	0.035	0.395	67
		Lag 2	0.000	-0.057	0.058	0.494	63
		Lag 3	0.001	-0.048	0.050	0.484	61
Cerebrovascular	Lag 0	Lag 0	0.010	-0.034	0.054	0.331	99
		Lag 1	0.028	-0.017	0.074	0.111	88
		Lag 2	0.030	-0.035	0.095	0.182	91
		Lag 3	-0.006	-0.085	0.074	0.443	91
	Lag 1	Lag 0	0.026	-0.017	0.069	0.121	106
		Lag 1	0.048	0.009	0.086	**0.007**	110
		Lag 2	0.028	-0.022	0.077	0.137	116
		Lag 3	-0.004	-0.080	0.072	0.460	115
	Lag 2	Lag 0	0.007	-0.047	0.061	0.396	78
		Lag 1	0.034	-0.005	0.072	**0.043**	96
		Lag 2	0.000	-0.044	0.045	0.492	102
		Lag 3	0.000	-0.057	0.057	0.499	102
	Lag 3	Lag 0	0.023	-0.033	0.080	0.208	78
		Lag 1	0.041	0.001	0.081	**0.022**	89
		Lag 2	-0.008	-0.053	0.036	0.358	93
		Lag 3	0.001	-0.044	0.046	0.488	91
Renal	Lag 0	Lag 0	0.006	-0.061	0.072	0.436	32
		Lag 1	-0.099	-0.235	0.038	0.078	30
		Lag 2	0.046	-0.057	0.150	0.191	30
		Lag 3	0.074	-0.058	0.207	0.136	30
	Lag 1	Lag 0	0.027	-0.029	0.084	0.169	31
		Lag 1	-0.036	-0.126	0.054	0.216	36

			Lag 2	-0.023	-0.133	0.088	0.343	40
			Lag 3	0.108	0.001	0.215	**0.024**	41
		Lag 2	Lag 0	-0.002	-0.074	0.071	0.483	24
			Lag 1	-0.020	-0.114	0.073	0.336	31
			Lag 2	0.001	-0.084	0.086	0.490	34
			Lag 3	0.102	0.021	0.183	**0.007**	36
		Lag 3	Lag 0	-0.140	-0.305	0.025	**0.048**	25
			Lag 1	-0.075	-0.213	0.064	0.145	28
			Lag 2	-0.055	-0.158	0.047	0.146	30
			Lag 3	0.020	-0.042	0.081	0.264	29
	Respiratory	Lag 0	Lag 0	0.001	-0.094	0.096	0.492	42
			Lag 1	0.018	-0.058	0.094	0.319	38
			Lag 2	0.006	-0.092	0.104	0.455	37
			Lag 3	0.143	0.061	0.226	**0.000**	36
		Lag 1	Lag 0	-0.062	-0.174	0.051	0.141	37
			Lag 1	0.015	-0.052	0.081	0.333	40
			Lag 2	0.012	-0.063	0.088	0.376	39
			Lag 3	0.119	0.031	0.206	**0.004**	38
		Lag 2	Lag 0	0.032	-0.039	0.103	0.191	46
			Lag 1	0.051	-0.001	0.103	**0.027**	47
			Lag 2	0.013	-0.058	0.084	0.356	48
			Lag 3	0.043	-0.053	0.139	0.189	45
		Lag 3	Lag 0	-0.020	-0.110	0.069	0.327	48
			Lag 1	0.023	-0.022	0.068	0.162	34
			Lag 2	0.003	-0.066	0.072	0.464	38
			Lag 3	0.026	-0.049	0.101	0.247	35
Black	All-natural	Lag 0	Lag 0	-0.003	-0.008	0.002	0.123	5850
			Lag 1	-0.005	-0.011	0.001	**0.038**	5603
			Lag 2	-0.002	-0.009	0.005	0.322	5574
			Lag 3	0.000	-0.007	0.007	0.488	5550
		Lag 1	Lag 0	0.001	-0.004	0.006	0.306	5580
			Lag 1	0.000	-0.005	0.005	0.467	5929
			Lag 2	-0.002	-0.008	0.003	0.193	5969
			Lag 3	-0.001	-0.008	0.006	0.388	5990
		Lag 2	Lag 0	0.006	0.001	0.012	**0.013**	4870
			Lag 1	0.000	-0.005	0.004	0.471	5585
			Lag 2	-0.004	-0.009	0.001	**0.049**	5605
			Lag 3	-0.005	-0.011	0.000	**0.027**	5623
		Lag 3	Lag 0	0.005	-0.001	0.012	0.061	5043
			Lag 1	0.003	-0.002	0.008	0.130	4930
			Lag 2	-0.004	-0.008	0.001	0.055	4976
			Lag 3	-0.003	-0.008	0.002	0.138	5022
	Cardiovascular	Lag 0	Lag 0	0.038	-0.035	0.111	0.155	14
			Lag 1	-0.055	-0.176	0.066	0.186	14
			Lag 2	0.031	-0.101	0.163	0.322	14
			Lag 3	-0.323	-0.684	0.037	**0.039**	14
		Lag 1	Lag 0	0.039	-0.037	0.116	0.156	10

		Lag 1	-0.001	-0.098	0.096	0.491	14
		Lag 2	0.089	0.007	0.171	**0.016**	14
		Lag 3	0.016	-0.116	0.149	0.404	15
	Lag 2	Lag 0	-	-	-	-	< 10
		Lag 1	-	-	-	-	< 10
		Lag 2	-	-	-	-	< 10
		Lag 3	-	-	-	-	< 10
	Lag 3	Lag 0	-	-	-	-	< 10
		Lag 1	-	-	-	-	< 10
		Lag 2	-	-	-	-	< 10
		Lag 3	-	-	-	-	< 10
Cerebrovascular	Lag 0	Lag 0	0.004	-0.102	0.110	0.474	19
		Lag 1	0.014	-0.073	0.101	0.376	23
		Lag 2	0.049	-0.068	0.166	0.206	23
		Lag 3	0.148	0.041	0.254	**0.003**	23
	Lag 1	Lag 0	-0.005	-0.113	0.103	0.463	20
		Lag 1	0.027	-0.054	0.108	0.253	25
		Lag 2	-0.014	-0.139	0.111	0.414	25
		Lag 3	0.102	-0.004	0.208	**0.030**	25
	Lag 2	Lag 0	0.038	-0.067	0.143	0.239	21
		Lag 1	0.042	-0.022	0.105	0.098	25
		Lag 2	-0.001	-0.086	0.083	0.490	25
		Lag 3	0.047	-0.024	0.117	0.096	25
	Lag 3	Lag 0	0.157	0.040	0.274	**0.004**	23
		Lag 1	-0.004	-0.076	0.069	0.462	22
		Lag 2	0.007	-0.057	0.071	0.416	21
		Lag 3	0.022	-0.045	0.088	0.263	21
Renal	Lag 0	Lag 0	-	-	-	-	< 10
		Lag 1	-	-	-	-	< 10
		Lag 2	-	-	-	-	< 10
		Lag 3	-	-	-	-	< 10
	Lag 1	Lag 0	-	-	-	-	< 10
		Lag 1	-	-	-	-	< 10
		Lag 2	-	-	-	-	< 10
		Lag 3	-	-	-	-	< 10
	Lag 2	Lag 0	-	-	-	-	< 10
		Lag 1	-	-	-	-	< 10
		Lag 2	-	-	-	-	< 10
		Lag 3	-	-	-	-	< 10
	Lag 3	Lag 0	-	-	-	-	< 10
		Lag 1	-	-	-	-	< 10
		Lag 2	-	-	-	-	< 10
		Lag 3	-	-	-	-	< 10
Respiratory	Lag 0	Lag 0	0.084	-0.033	0.201	0.079	19
		Lag 1	0.160	0.034	0.287	**0.006**	16
		Lag 2	0.066	-0.071	0.204	0.172	17
		Lag 3	0.193	0.065	0.321	**0.002**	17

		Lag 1	Lag 0	0.059	-0.062	0.179	0.171	15
			Lag 1	0.155	0.024	0.287	**0.010**	16
			Lag 2	-0.016	-0.158	0.126	0.412	17
			Lag 3	0.127	0.004	0.250	**0.022**	17
		Lag 2	Lag 0	0.116	-0.014	0.247	**0.040**	14
			Lag 1	0.064	-0.038	0.166	0.108	16
			Lag 2	0.056	-0.058	0.171	0.166	17
			Lag 3	0.094	-0.029	0.217	0.067	18
		Lag 3	Lag 0	0.078	-0.120	0.277	0.219	10
			Lag 1	-	-	-	-	< 10
			Lag 2	-	-	-	-	< 10
			Lag 3	-	-	-	-	< 10
Hispanic	All-natural	Lag 0	Lag 0	0.003	-0.001	0.007	0.068	17224
			Lag 1	0.000	-0.004	0.004	0.465	16433
			Lag 2	-0.002	-0.007	0.002	0.178	16322
			Lag 3	0.001	-0.004	0.005	0.402	16218
		Lag 1	Lag 0	0.002	-0.002	0.005	0.151	16664
			Lag 1	-0.002	-0.006	0.002	0.123	16697
			Lag 2	-0.004	-0.008	0.000	**0.020**	16758
			Lag 3	0.001	-0.003	0.006	0.274	16781
		Lag 2	Lag 0	0.001	-0.003	0.005	0.235	14871
			Lag 1	-0.002	-0.006	0.001	0.120	16582
			Lag 2	-0.007	-0.011	-0.003	**0.000**	16642
			Lag 3	0.001	-0.003	0.005	0.307	16666
		Lag 3	Lag 0	0.003	-0.002	0.007	0.109	15821
			Lag 1	0.001	-0.003	0.005	0.334	15157
			Lag 2	-0.007	-0.010	-0.003	**0.000**	15308
			Lag 3	-0.001	-0.005	0.003	0.276	15464
	Cardiovascular	Lag 0	Lag 0	0.019	-0.097	0.135	0.374	23
			Lag 1	0.033	-0.069	0.134	0.265	18
			Lag 2	-0.109	-0.266	0.047	0.085	17
			Lag 3	-0.045	-0.171	0.080	0.241	15
		Lag 1	Lag 0	0.011	-0.105	0.128	0.425	27
			Lag 1	0.020	-0.074	0.114	0.338	20
			Lag 2	-0.065	-0.185	0.055	0.143	20
			Lag 3	-0.057	-0.178	0.064	0.179	19
		Lag 2	Lag 0	0.024	-0.080	0.128	0.324	26
			Lag 1	0.039	-0.050	0.129	0.196	26
			Lag 2	-0.049	-0.151	0.054	0.176	26
			Lag 3	0.031	-0.060	0.122	0.251	24
		Lag 3	Lag 0	0.048	-0.059	0.154	0.190	32
			Lag 1	0.017	-0.083	0.117	0.369	25
			Lag 2	-0.104	-0.235	0.028	0.061	25
			Lag 3	0.039	-0.054	0.132	0.204	23
	Cerebrovascular	Lag 0	Lag 0	-0.063	-0.173	0.047	0.130	37
			Lag 1	-0.042	-0.131	0.047	0.179	40
			Lag 2	0.013	-0.090	0.116	0.402	41

			Lag 3	0.032	-0.045	0.109	0.207	41
		Lag 1	Lag 0	-0.004	-0.102	0.094	0.467	43
			Lag 1	-0.046	-0.132	0.039	0.144	40
			Lag 2	-0.024	-0.117	0.069	0.307	41
			Lag 3	0.010	-0.070	0.090	0.400	42
		Lag 2	Lag 0	0.027	-0.070	0.124	0.291	40
			Lag 1	-0.023	-0.090	0.044	0.249	43
			Lag 2	-0.058	-0.159	0.043	0.130	43
			Lag 3	0.026	-0.047	0.099	0.242	44
		Lag 3	Lag 0	0.024	-0.072	0.120	0.312	45
			Lag 1	-0.097	-0.210	0.015	**0.045**	33
			Lag 2	-0.006	-0.070	0.057	0.422	34
			Lag 3	0.035	-0.032	0.102	0.151	34
	Renal	Lag 0	Lag 0	0.077	-0.044	0.199	0.107	20
			Lag 1	-0.031	-0.175	0.113	0.335	14
			Lag 2	0.018	-0.153	0.189	0.418	13
			Lag 3	0.109	-0.023	0.241	0.052	14
		Lag 1	Lag 0	-0.060	-0.253	0.133	0.271	12
			Lag 1	-0.010	-0.159	0.138	0.446	13
			Lag 2	-0.071	-0.270	0.129	0.245	13
			Lag 3	0.060	-0.073	0.194	0.188	14
		Lag 2	Lag 0	0.018	-0.135	0.172	0.408	13
			Lag 1	-0.020	-0.157	0.117	0.387	17
			Lag 2	-0.011	-0.160	0.138	0.442	17
			Lag 3	0.057	-0.049	0.162	0.146	18
		Lag 3	Lag 0	0.026	-0.121	0.174	0.363	17
			Lag 1	0.026	-0.122	0.173	0.367	16
			Lag 2	0.101	0.006	0.195	**0.019**	17
			Lag 3	0.061	-0.040	0.162	0.119	16
	Respiratory	Lag 0	Lag 0	0.005	-0.115	0.126	0.465	18
			Lag 1	-0.030	-0.201	0.141	0.366	12
			Lag 2	0.027	-0.125	0.179	0.365	12
			Lag 3	-0.051	-0.212	0.109	0.266	11
		Lag 1	Lag 0	0.051	-0.056	0.159	0.174	17
			Lag 1	-0.018	-0.171	0.135	0.409	17
			Lag 2	0.006	-0.140	0.152	0.468	17
			Lag 3	0.007	-0.135	0.150	0.459	16
		Lag 2	Lag 0	0.100	0.006	0.195	**0.019**	24
			Lag 1	-0.015	-0.109	0.078	0.373	20
			Lag 2	0.011	-0.135	0.157	0.440	21
			Lag 3	0.052	-0.049	0.154	0.157	19
		Lag 3	Lag 0	0.077	-0.049	0.203	0.115	27
			Lag 1	-0.116	-0.294	0.062	0.100	13
			Lag 2	-0.181	-0.414	0.052	0.064	14
			Lag 3	0.088	-0.020	0.195	0.054	13
Asian	All-natural	Lag 0	Lag 0	0.005	0.000	0.010	**0.020**	5393
			Lag 1	0.007	0.002	0.012	**0.006**	4847

		Lag 2	-0.006	-0.015	0.002	0.065	4760
		Lag 3	-0.001	-0.009	0.006	0.378	4698
	Lag 1	Lag 0	0.008	0.004	0.013	**0.000**	5083
		Lag 1	0.009	0.004	0.013	**0.000**	5355
		Lag 2	0.002	-0.004	0.007	0.247	5348
		Lag 3	0.004	-0.004	0.012	0.151	5344
	Lag 2	Lag 0	0.011	0.006	0.016	**0.000**	4500
		Lag 1	0.006	0.002	0.010	**0.003**	5123
		Lag 2	0.003	-0.002	0.008	0.112	5147
		Lag 3	-0.001	-0.007	0.004	0.338	5187
	Lag 3	Lag 0	0.011	0.005	0.017	**0.000**	4487
		Lag 1	0.004	-0.001	0.009	0.056	4626
		Lag 2	0.002	-0.002	0.007	0.133	4690
		Lag 3	0.000	-0.005	0.005	0.481	4750
Cardiovascular	Lag 0	Lag 0	-	-	-	-	< 10
		Lag 1	-	-	-	-	< 10
		Lag 2	-	-	-	-	< 10
		Lag 3	-	-	-	-	< 10
	Lag 1	Lag 0	-	-	-	-	< 10
		Lag 1	-	-	-	-	< 10
		Lag 2	-	-	-	-	< 10
		Lag 3	-	-	-	-	< 10
	Lag 2	Lag 0	-	-	-	-	< 10
		Lag 1	-	-	-	-	< 10
		Lag 2	-	-	-	-	< 10
		Lag 3	-	-	-	-	< 10
	Lag 3	Lag 0	-	-	-	-	< 10
		Lag 1	-	-	-	-	< 10
		Lag 2	-	-	-	-	< 10
		Lag 3	-	-	-	-	< 10
Cerebrovascular	Lag 0	Lag 0	-0.028	-0.113	0.056	0.256	17
		Lag 1	-0.086	-0.206	0.033	0.079	19
		Lag 2	-0.117	-0.292	0.058	0.095	18
		Lag 3	-0.068	-0.222	0.085	0.192	20
	Lag 1	Lag 0	-0.045	-0.137	0.047	0.169	19
		Lag 1	-0.102	-0.211	0.008	**0.035**	26
		Lag 2	-0.024	-0.120	0.072	0.312	26
		Lag 3	-0.087	-0.242	0.068	0.135	28
	Lag 2	Lag 0	-0.045	-0.150	0.060	0.199	15
		Lag 1	-0.033	-0.089	0.022	0.120	29
		Lag 2	0.014	-0.051	0.078	0.341	27
		Lag 3	-0.008	-0.084	0.069	0.423	28
	Lag 3	Lag 0	-0.013	-0.114	0.088	0.400	20
		Lag 1	-0.002	-0.058	0.054	0.473	24
		Lag 2	0.055	0.005	0.105	**0.016**	24
		Lag 3	0.024	-0.038	0.087	0.223	24
Renal	Lag 0	Lag 0	-	-	-	-	< 10

			Lag 1	-	-	-	-	< 10
			Lag 2	-	-	-	-	< 10
			Lag 3	-	-	-	-	< 10
		Lag 1	Lag 0	0.013	-0.278	0.304	0.465	10
			Lag 1	-	-	-	-	< 10
			Lag 2	-	-	-	-	< 10
			Lag 3	-	-	-	-	< 10
		Lag 2	Lag 0	-	-	-	-	< 10
			Lag 1	-	-	-	-	< 10
			Lag 2	-	-	-	-	< 10
			Lag 3	-	-	-	-	< 10
		Lag 3	Lag 0	-	-	-	-	< 10
			Lag 1	-	-	-	-	< 10
			Lag 2	-	-	-	-	< 10
			Lag 3	-	-	-	-	< 10
	Respiratory	Lag 0	Lag 0	-	-	-	-	< 10
			Lag 1	-	-	-	-	< 10
			Lag 2	-	-	-	-	< 10
			Lag 3	-	-	-	-	< 10
		Lag 1	Lag 0	-	-	-	-	< 10
			Lag 1	-	-	-	-	< 10
			Lag 2	-	-	-	-	< 10
			Lag 3	-	-	-	-	< 10
		Lag 2	Lag 0	-	-	-	-	< 10
			Lag 1	-	-	-	-	< 10
			Lag 2	-	-	-	-	< 10
			Lag 3	-	-	-	-	< 10
		Lag 3	Lag 0	-	-	-	-	< 10
			Lag 1	-	-	-	-	< 10
			Lag 2	-	-	-	-	< 10
			Lag 3	-	-	-	-	< 10
Other Grouped Races	All-natural	Lag 0	Lag 0	0.004	-0.005	0.014	0.179	2542
			Lag 1	0.005	-0.006	0.016	0.184	2421
			Lag 2	0.009	-0.004	0.023	0.083	2395
			Lag 3	0.005	-0.007	0.017	0.210	2374
		Lag 1	Lag 0	0.007	-0.002	0.016	0.069	2522
			Lag 1	-0.004	-0.013	0.006	0.230	2602
			Lag 2	0.007	-0.003	0.018	0.080	2605
			Lag 3	0.002	-0.011	0.015	0.389	2598
		Lag 2	Lag 0	0.005	-0.006	0.015	0.191	2251
			Lag 1	-0.009	-0.018	0.001	**0.033**	2619
			Lag 2	0.002	-0.008	0.011	0.352	2642
			Lag 3	-0.003	-0.014	0.007	0.273	2654
		Lag 3	Lag 0	-0.003	-0.015	0.010	0.335	2271
			Lag 1	0.002	-0.008	0.012	0.344	2392
			Lag 2	0.001	-0.007	0.010	0.371	2426
			Lag 3	-0.009	-0.019	0.001	**0.039**	2461

Cardiovascular	Lag 0	Lag 0	-	-	-	-	< 10
		Lag 1	-	-	-	-	< 10
		Lag 2	-	-	-	-	< 10
		Lag 3	-	-	-	-	< 10
	Lag 1	Lag 0	-	-	-	-	< 10
		Lag 1	-	-	-	-	< 10
		Lag 2	-	-	-	-	< 10
		Lag 3	-	-	-	-	< 10
	Lag 2	Lag 0	-	-	-	-	< 10
		Lag 1	-	-	-	-	< 10
		Lag 2	-	-	-	-	< 10
		Lag 3	-	-	-	-	< 10
	Lag 3	Lag 0	-	-	-	-	< 10
		Lag 1	-	-	-	-	< 10
		Lag 2	-	-	-	-	< 10
		Lag 3	-	-	-	-	< 10
Cerebrovascular	Lag 0	Lag 0	0.179	0.032	0.326	**0.009**	11
		Lag 1	-	-	-	-	< 10
		Lag 2	-	-	-	-	< 10
		Lag 3	-	-	-	-	< 10
	Lag 1	Lag 0	-	-	-	-	< 10
		Lag 1	-	-	-	-	< 10
		Lag 2	-	-	-	-	< 10
		Lag 3	-	-	-	-	< 10
	Lag 2	Lag 0	-	-	-	-	< 10
		Lag 1	0.047	-0.090	0.183	0.252	11
		Lag 2	0.026	-0.247	0.300	0.425	10
		Lag 3	0.114	-0.085	0.314	0.131	11
	Lag 3	Lag 0	-	-	-	-	< 10
		Lag 1	0.036	-0.169	0.240	0.366	10
		Lag 2	0.066	-0.184	0.316	0.303	10
		Lag 3	0.154	-0.058	0.367	0.077	10
Renal	Lag 0	Lag 0	-	-	-	-	< 10
		Lag 1	-	-	-	-	< 10
		Lag 2	-	-	-	-	< 10
		Lag 3	-	-	-	-	< 10
	Lag 1	Lag 0	-	-	-	-	< 10
		Lag 1	-	-	-	-	< 10
		Lag 2	-	-	-	-	< 10
		Lag 3	-	-	-	-	< 10
	Lag 2	Lag 0	-	-	-	-	< 10
		Lag 1	-	-	-	-	< 10
		Lag 2	-	-	-	-	< 10
		Lag 3	-	-	-	-	< 10
	Lag 3	Lag 0	-	-	-	-	< 10
		Lag 1	-	-	-	-	< 10
		Lag 2	-	-	-	-	< 10

			Lag 3	-	-	-	-	< 10
	Respiratory	Lag 0	Lag 0	-	-	-	-	< 10
			Lag 1	-	-	-	-	< 10
			Lag 2	-	-	-	-	< 10
			Lag 3	-	-	-	-	< 10
		Lag 1	Lag 0	-	-	-	-	< 10
			Lag 1	-	-	-	-	< 10
			Lag 2	-	-	-	-	< 10
			Lag 3	-	-	-	-	< 10
		Lag 2	Lag 0	-	-	-	-	< 10
			Lag 1	-	-	-	-	< 10
			Lag 2	-	-	-	-	< 10
			Lag 3	-	-	-	-	< 10
		Lag 3	Lag 0	-	-	-	-	< 10
			Lag 1	-	-	-	-	< 10
			Lag 2	-	-	-	-	< 10
			Lag 3	-	-	-	-	< 10
Higher Education (< 50% high school education or less)	All-natural	Lag 0	Lag 0	0.002	0.000	0.004	**0.020**	39608
			Lag 1	0.001	-0.001	0.004	0.155	35830
			Lag 2	-0.004	-0.008	-0.001	**0.005**	35466
			Lag 3	0.003	0.000	0.006	**0.036**	35132
		Lag 1	Lag 0	0.003	0.001	0.005	**0.003**	38335
			Lag 1	-0.001	-0.003	0.001	0.133	38928
			Lag 2	-0.003	-0.005	0.000	**0.011**	39031
			Lag 3	0.001	-0.002	0.004	0.196	39035
		Lag 2	Lag 0	0.004	0.001	0.006	**0.001**	34605
			Lag 1	-0.001	-0.003	0.001	0.135	38473
			Lag 2	-0.004	-0.006	-0.002	**0.000**	38704
			Lag 3	-0.002	-0.004	0.000	**0.043**	38828
		Lag 3	Lag 0	0.005	0.002	0.007	**0.000**	35172
			Lag 1	0.001	-0.001	0.003	0.201	35858
			Lag 2	-0.003	-0.005	-0.001	**0.000**	36299
			Lag 3	-0.001	-0.004	0.001	0.091	36669
	Cardiovascular	Lag 0	Lag 0	0.014	-0.029	0.058	0.261	74
			Lag 1	0.008	-0.037	0.053	0.364	79
			Lag 2	0.006	-0.067	0.080	0.434	75
			Lag 3	-0.024	-0.098	0.049	0.258	74
		Lag 1	Lag 0	0.024	-0.020	0.068	0.142	67
			Lag 1	0.002	-0.040	0.044	0.460	76
			Lag 2	0.033	-0.018	0.084	0.100	74
			Lag 3	-0.014	-0.086	0.057	0.347	76
		Lag 2	Lag 0	0.037	-0.011	0.084	0.066	76
			Lag 1	-0.010	-0.046	0.026	0.290	83
			Lag 2	0.002	-0.043	0.048	0.458	81
			Lag 3	-0.010	-0.057	0.037	0.334	79
		Lag 3	Lag 0	0.043	-0.012	0.098	0.064	92
			Lag 1	0.000	-0.038	0.037	0.491	78

		Lag 2	0.001	-0.040	0.042	0.482	75
		Lag 3	0.006	-0.035	0.047	0.393	73
Cerebrovascular	Lag 0	Lag 0	0.009	-0.027	0.045	0.310	136
		Lag 1	-0.007	-0.046	0.031	0.354	120
		Lag 2	0.023	-0.033	0.080	0.208	121
		Lag 3	0.041	-0.010	0.092	0.056	122
	Lag 1	Lag 0	0.018	-0.018	0.053	0.164	143
		Lag 1	0.006	-0.025	0.037	0.346	152
		Lag 2	-0.001	-0.045	0.043	0.483	155
		Lag 3	0.004	-0.053	0.061	0.444	156
	Lag 2	Lag 0	0.026	-0.015	0.067	0.104	124
		Lag 1	0.009	-0.018	0.036	0.253	139
		Lag 2	-0.005	-0.041	0.030	0.386	140
		Lag 3	0.009	-0.029	0.048	0.321	144
	Lag 3	Lag 0	0.030	-0.015	0.076	0.096	124
		Lag 1	0.006	-0.027	0.038	0.367	115
		Lag 2	0.006	-0.022	0.034	0.330	116
		Lag 3	0.020	-0.011	0.052	0.099	115
Renal	Lag 0	Lag 0	0.035	-0.023	0.092	0.118	49
		Lag 1	0.021	-0.069	0.112	0.323	44
		Lag 2	0.030	-0.071	0.130	0.280	43
		Lag 3	0.057	-0.038	0.152	0.120	44
	Lag 1	Lag 0	0.036	-0.017	0.088	0.090	46
		Lag 1	0.032	-0.044	0.109	0.204	49
		Lag 2	-0.052	-0.158	0.054	0.169	50
		Lag 3	0.049	-0.037	0.136	0.131	52
	Lag 2	Lag 0	0.006	-0.063	0.075	0.433	31
		Lag 1	0.030	-0.054	0.114	0.244	38
		Lag 2	-0.018	-0.100	0.064	0.331	40
		Lag 3	0.021	-0.047	0.089	0.271	42
	Lag 3	Lag 0	-0.046	-0.143	0.051	0.175	34
		Lag 1	-0.019	-0.129	0.091	0.365	31
		Lag 2	-0.016	-0.086	0.053	0.321	34
		Lag 3	-0.001	-0.060	0.058	0.491	35
Respiratory	Lag 0	Lag 0	0.037	-0.029	0.103	0.138	60
		Lag 1	0.044	-0.021	0.110	0.092	50
		Lag 2	-0.013	-0.111	0.085	0.396	50
		Lag 3	0.120	0.045	0.194	**0.001**	49
	Lag 1	Lag 0	0.009	-0.064	0.082	0.408	54
		Lag 1	0.042	-0.017	0.102	0.082	49
		Lag 2	0.000	-0.067	0.068	0.496	49
		Lag 3	0.104	0.026	0.182	**0.005**	48
	Lag 2	Lag 0	0.048	-0.017	0.113	0.074	58
		Lag 1	0.061	0.011	0.111	**0.009**	58
		Lag 2	-0.006	-0.073	0.061	0.433	61
		Lag 3	0.077	0.007	0.146	**0.016**	59
	Lag 3	Lag 0	0.022	-0.055	0.099	0.289	67

			Lag 1	0.026	-0.044	0.096	0.236	43
			Lag 2	-0.003	-0.070	0.064	0.461	49
			Lag 3	0.043	-0.019	0.104	0.089	49
Lower Education (> 50% high school education or less)	All-natural	Lag 0	Lag 0	0.000	-0.003	0.004	0.401	20355
			Lag 1	-0.002	-0.006	0.002	0.154	19057
			Lag 2	-0.002	-0.007	0.002	0.136	18846
			Lag 3	-0.003	-0.007	0.001	0.091	18633
		Lag 1	Lag 0	0.003	0.000	0.007	**0.019**	20223
			Lag 1	-0.002	-0.005	0.002	0.204	19850
			Lag 2	-0.003	-0.007	0.000	**0.035**	19900
			Lag 3	-0.001	-0.005	0.003	0.307	19914
		Lag 2	Lag 0	0.003	0.000	0.006	**0.029**	18139
			Lag 1	-0.003	-0.006	0.000	**0.040**	19791
			Lag 2	-0.007	-0.011	-0.003	**0.000**	19872
			Lag 3	-0.002	-0.006	0.002	0.141	19926
		Lag 3	Lag 0	0.004	0.000	0.008	**0.018**	18980
			Lag 1	0.003	0.000	0.006	**0.042**	18189
			Lag 2	-0.006	-0.010	-0.003	**0.000**	18337
			Lag 3	0.000	-0.004	0.003	0.442	18468
	Cardiovascular	Lag 0	Lag 0	0.016	-0.068	0.099	0.357	37
			Lag 1	0.035	-0.046	0.116	0.199	36
			Lag 2	0.017	-0.081	0.115	0.367	35
			Lag 3	-0.057	-0.166	0.053	0.156	33
		Lag 1	Lag 0	0.019	-0.054	0.092	0.307	44
			Lag 1	0.030	-0.047	0.108	0.223	39
			Lag 2	0.016	-0.062	0.094	0.343	38
			Lag 3	0.003	-0.084	0.090	0.472	35
		Lag 2	Lag 0	0.079	0.007	0.151	**0.016**	37
			Lag 1	-0.023	-0.112	0.066	0.304	42
			Lag 2	-0.037	-0.132	0.059	0.226	40
			Lag 3	-0.031	-0.126	0.064	0.261	38
		Lag 3	Lag 0	0.090	0.004	0.176	**0.020**	40
			Lag 1	-0.008	-0.074	0.059	0.410	34
			Lag 2	-0.132	-0.261	-0.002	**0.023**	32
			Lag 3	-0.030	-0.133	0.073	0.281	31
	Cerebrovascular	Lag 0	Lag 0	-0.046	-0.133	0.041	0.149	47
			Lag 1	0.019	-0.047	0.085	0.287	59
			Lag 2	-0.003	-0.084	0.078	0.472	60
			Lag 3	-0.009	-0.101	0.082	0.420	62
		Lag 1	Lag 0	-0.019	-0.095	0.057	0.310	51
			Lag 1	0.004	-0.070	0.079	0.456	57
			Lag 2	0.010	-0.063	0.083	0.394	60
			Lag 3	0.017	-0.056	0.090	0.321	62
		Lag 2	Lag 0	-0.053	-0.147	0.042	0.138	36
			Lag 1	0.011	-0.052	0.073	0.370	65
			Lag 2	-0.014	-0.083	0.055	0.344	67
			Lag 3	0.033	-0.030	0.097	0.153	66

		Lag 3	Lag 0	0.023	-0.054	0.100	0.282	49
			Lag 1	0.029	-0.022	0.080	0.135	63
			Lag 2	0.040	-0.024	0.104	0.112	65
			Lag 3	0.019	-0.048	0.086	0.290	65
	Renal	Lag 0	Lag 0	0.010	-0.096	0.115	0.429	22
			Lag 1	-0.069	-0.192	0.055	0.138	20
			Lag 2	0.045	-0.058	0.149	0.195	19
			Lag 3	0.057	-0.120	0.235	0.264	19
		Lag 1	Lag 0	0.025	-0.082	0.132	0.323	20
			Lag 1	-0.113	-0.262	0.035	0.067	18
			Lag 2	0.009	-0.118	0.136	0.447	20
			Lag 3	0.099	-0.036	0.233	0.076	20
		Lag 2	Lag 0	-0.028	-0.165	0.109	0.345	19
			Lag 1	-0.078	-0.218	0.063	0.139	20
			Lag 2	0.056	-0.055	0.167	0.160	21
			Lag 3	0.134	0.030	0.238	**0.006**	23
		Lag 3	Lag 0	-0.064	-0.244	0.115	0.242	22
			Lag 1	-0.040	-0.198	0.117	0.308	26
			Lag 2	0.060	-0.041	0.162	0.121	27
			Lag 3	0.069	-0.026	0.165	0.078	25
	Respiratory	Lag 0	Lag 0	-0.071	-0.215	0.073	0.168	25
			Lag 1	0.006	-0.106	0.119	0.455	25
			Lag 2	0.020	-0.078	0.118	0.345	26
			Lag 3	0.030	-0.062	0.122	0.263	25
		Lag 1	Lag 0	-0.036	-0.162	0.089	0.286	24
			Lag 1	0.027	-0.067	0.120	0.289	31
			Lag 2	-0.028	-0.132	0.076	0.300	32
			Lag 3	0.047	-0.048	0.143	0.167	31
		Lag 2	Lag 0	0.065	-0.012	0.142	**0.048**	38
			Lag 1	0.002	-0.065	0.069	0.476	32
			Lag 2	-0.001	-0.093	0.090	0.490	34
			Lag 3	-0.020	-0.128	0.088	0.358	32
		Lag 3	Lag 0	-0.032	-0.147	0.082	0.290	32
			Lag 1	0.015	-0.023	0.054	0.215	21
			Lag 2	-0.053	-0.166	0.059	0.176	23
			Lag 3	0.004	-0.092	0.100	0.465	21
Lesser poverty (<25% households living under poverty)	All-natural	Lag 0	Lag 0	0.002	0.000	0.004	**0.039**	47779
			Lag 1	0.001	-0.001	0.003	0.206	42041
			Lag 2	-0.005	-0.008	-0.002	**0.001**	41560
			Lag 3	0.002	0.000	0.005	0.052	41129
		Lag 1	Lag 0	0.003	0.001	0.005	**0.002**	46248
			Lag 1	-0.001	-0.003	0.001	0.162	46782
			Lag 2	-0.003	-0.005	0.000	**0.013**	46844
			Lag 3	0.001	-0.002	0.004	0.314	46798
		Lag 2	Lag 0	0.004	0.002	0.006	**0.000**	41676
			Lag 1	-0.002	-0.003	0.000	0.050	46270
			Lag 2	-0.005	-0.007	-0.002	**0.000**	46530

		Lag 3	-0.003	-0.005	-0.001	**0.005**	46651
	Lag 3	Lag 0	0.005	0.002	0.007	**0.000**	42465
		Lag 1	0.002	0.000	0.004	**0.046**	42950
		Lag 2	-0.004	-0.006	-0.002	**0.000**	43445
		Lag 3	-0.001	-0.003	0.001	0.077	43860
Cardiovascular	Lag 0	Lag 0	0.001	-0.044	0.045	0.487	81
		Lag 1	0.015	-0.026	0.056	0.238	91
		Lag 2	-0.001	-0.070	0.067	0.483	86
		Lag 3	-0.033	-0.101	0.035	0.172	84
	Lag 1	Lag 0	0.018	-0.025	0.061	0.209	84
		Lag 1	0.005	-0.035	0.045	0.405	90
		Lag 2	0.025	-0.023	0.073	0.158	89
		Lag 3	-0.023	-0.095	0.048	0.263	90
	Lag 2	Lag 0	0.047	0.002	0.092	**0.019**	86
		Lag 1	-0.016	-0.055	0.022	0.201	102
		Lag 2	-0.015	-0.063	0.033	0.275	99
		Lag 3	-0.030	-0.084	0.025	0.143	95
	Lag 3	Lag 0	0.057	0.005	0.110	**0.016**	104
		Lag 1	-0.001	-0.038	0.036	0.479	91
		Lag 2	-0.020	-0.065	0.026	0.202	85
		Lag 3	-0.010	-0.055	0.035	0.331	82
Cerebrovascular	Lag 0	Lag 0	0.010	-0.025	0.046	0.290	154
		Lag 1	0.007	-0.028	0.041	0.349	149
		Lag 2	0.019	-0.035	0.072	0.247	150
		Lag 3	0.034	-0.015	0.084	0.086	153
	Lag 1	Lag 0	0.023	-0.011	0.057	0.096	166
		Lag 1	0.008	-0.022	0.039	0.299	176
		Lag 2	0.004	-0.038	0.045	0.433	180
		Lag 3	-0.008	-0.063	0.048	0.395	183
	Lag 2	Lag 0	0.015	-0.025	0.056	0.232	136
		Lag 1	0.009	-0.018	0.035	0.259	166
		Lag 2	-0.003	-0.037	0.031	0.430	169
		Lag 3	0.020	-0.016	0.055	0.140	175
	Lag 3	Lag 0	0.034	-0.009	0.077	0.061	148
		Lag 1	0.008	-0.023	0.040	0.300	147
		Lag 2	0.015	-0.012	0.041	0.137	149
		Lag 3	0.027	-0.002	0.057	**0.034**	151
Renal	Lag 0	Lag 0	0.006	-0.059	0.071	0.425	54
		Lag 1	-0.019	-0.110	0.073	0.344	45
		Lag 2	0.002	-0.097	0.101	0.486	43
		Lag 3	0.052	-0.047	0.150	0.152	43
	Lag 1	Lag 0	0.029	-0.030	0.087	0.168	50
		Lag 1	-0.005	-0.080	0.070	0.446	51
		Lag 2	-0.090	-0.212	0.032	0.073	52
		Lag 3	0.062	-0.023	0.147	0.075	53
	Lag 2	Lag 0	0.000	-0.075	0.076	0.496	41
		Lag 1	0.015	-0.060	0.090	0.345	47

			Lag 2	-0.012	-0.093	0.070	0.390	48
			Lag 3	0.032	-0.033	0.097	0.167	50
		Lag 3	Lag 0	-0.018	-0.106	0.069	0.339	49
			Lag 1	-0.016	-0.112	0.080	0.371	45
			Lag 2	0.017	-0.042	0.076	0.287	49
			Lag 3	0.001	-0.055	0.058	0.481	48
	Respiratory	Lag 0	Lag 0	0.023	-0.048	0.095	0.260	65
			Lag 1	0.032	-0.030	0.094	0.154	59
			Lag 2	-0.004	-0.083	0.076	0.465	58
			Lag 3	0.095	0.025	0.165	**0.004**	57
		Lag 1	Lag 0	0.026	-0.046	0.097	0.241	63
			Lag 1	0.023	-0.036	0.082	0.218	62
			Lag 2	-0.003	-0.067	0.061	0.465	60
			Lag 3	0.072	-0.001	0.145	**0.027**	59
		Lag 2	Lag 0	0.067	0.009	0.126	**0.012**	75
			Lag 1	0.048	0.003	0.094	**0.019**	72
			Lag 2	-0.002	-0.063	0.058	0.469	74
			Lag 3	0.047	-0.023	0.118	0.095	71
		Lag 3	Lag 0	0.013	-0.061	0.087	0.362	79
			Lag 1	0.019	-0.017	0.055	0.152	50
			Lag 2	-0.008	-0.074	0.058	0.401	56
			Lag 3	0.030	-0.030	0.090	0.164	55
Higher poverty (>25% households living under poverty)	All-natural	Lag 0	Lag 0	0.001	-0.003	0.006	0.242	12242
			Lag 1	-0.002	-0.006	0.002	0.194	12892
			Lag 2	-0.001	-0.006	0.004	0.378	12774
			Lag 3	-0.003	-0.007	0.002	0.101	12649
		Lag 1	Lag 0	0.004	0.000	0.008	**0.022**	12365
			Lag 1	-0.002	-0.007	0.002	0.140	12039
			Lag 2	-0.004	-0.008	0.000	**0.027**	12118
			Lag 3	0.000	-0.005	0.005	0.475	12171
		Lag 2	Lag 0	0.003	-0.001	0.007	0.051	11139
			Lag 1	-0.002	-0.005	0.002	0.192	12052
			Lag 2	-0.007	-0.011	-0.003	**0.001**	12086
			Lag 3	0.001	-0.003	0.005	0.362	12126
		Lag 3	Lag 0	0.005	0.000	0.009	**0.022**	11756
			Lag 1	0.001	-0.003	0.005	0.342	11158
			Lag 2	-0.005	-0.009	-0.002	**0.002**	11241
			Lag 3	0.000	-0.004	0.004	0.445	11306
	Cardiovascular	Lag 0	Lag 0	0.072	-0.011	0.156	**0.045**	30
			Lag 1	0.005	-0.112	0.122	0.468	24
			Lag 2	0.049	-0.066	0.165	0.202	24
			Lag 3	-0.045	-0.181	0.092	0.262	23
		Lag 1	Lag 0	0.045	-0.032	0.122	0.126	27
			Lag 1	0.024	-0.071	0.118	0.311	25
			Lag 2	0.044	-0.047	0.135	0.173	23
			Lag 3	0.020	-0.067	0.107	0.328	21
		Lag 2	Lag 0	0.058	-0.025	0.141	0.084	27

		Lag 1	0.002	-0.070	0.074	0.480	24
		Lag 2	0.023	-0.056	0.102	0.285	23
		Lag 3	0.018	-0.055	0.090	0.315	23
	Lag 3	Lag 0	0.050	-0.056	0.155	0.178	28
		Lag 1	-0.015	-0.087	0.057	0.342	21
		Lag 2	-0.023	-0.111	0.065	0.302	22
		Lag 3	0.032	-0.048	0.112	0.219	22
Cerebrovascular	Lag 0	Lag 0	-0.058	-0.154	0.039	0.121	29
		Lag 1	-0.055	-0.152	0.043	0.135	30
		Lag 2	0.001	-0.091	0.094	0.488	31
		Lag 3	-0.003	-0.105	0.099	0.477	31
	Lag 1	Lag 0	-0.061	-0.163	0.041	0.120	28
		Lag 1	-0.006	-0.086	0.074	0.441	33
		Lag 2	-0.006	-0.092	0.080	0.443	35
		Lag 3	0.042	-0.036	0.119	0.146	35
	Lag 2	Lag 0	-0.013	-0.105	0.079	0.392	24
		Lag 1	0.019	-0.046	0.083	0.284	38
		Lag 2	-0.022	-0.101	0.057	0.293	38
		Lag 3	-0.004	-0.087	0.078	0.458	35
	Lag 3	Lag 0	0.005	-0.094	0.104	0.461	25
		Lag 1	0.027	-0.030	0.083	0.177	31
		Lag 2	-0.025	-0.109	0.058	0.276	33
		Lag 3	-0.041	-0.134	0.053	0.198	29
Renal	Lag 0	Lag 0	0.052	-0.030	0.135	0.106	17
		Lag 1	-0.029	-0.145	0.088	0.316	19
		Lag 2	0.093	-0.019	0.205	0.051	19
		Lag 3	0.073	-0.087	0.233	0.186	20
	Lag 1	Lag 0	0.038	-0.042	0.118	0.174	16
		Lag 1	-0.060	-0.208	0.087	0.212	16
		Lag 2	0.049	-0.064	0.162	0.197	18
		Lag 3	0.060	-0.081	0.202	0.202	19
	Lag 2	Lag 0	-	-	-	-	< 10
		Lag 1	-0.138	-0.384	0.107	0.134	11
		Lag 2	0.036	-0.073	0.144	0.260	13
		Lag 3	0.110	-0.001	0.220	**0.026**	15
	Lag 3	Lag 0	-	-	-	-	< 10
		Lag 1	-0.105	-0.380	0.171	0.228	12
		Lag 2	-0.066	-0.222	0.090	0.203	12
		Lag 3	0.080	-0.030	0.190	0.078	12
Respiratory	Lag 0	Lag 0	-0.024	-0.146	0.097	0.348	20
		Lag 1	0.059	-0.077	0.195	0.198	17
		Lag 2	0.013	-0.127	0.153	0.426	18
		Lag 3	0.053	-0.048	0.153	0.151	17
	Lag 1	Lag 0	-0.095	-0.248	0.058	0.113	15
		Lag 1	0.100	0.000	0.200	**0.025**	19
		Lag 2	-0.034	-0.160	0.092	0.297	21
		Lag 3	0.097	-0.009	0.204	**0.037**	20

		Lag 2	Lag 0	0.028	-0.065	0.120	0.279	21
			Lag 1	0.031	-0.043	0.105	0.208	19
			Lag 2	-0.012	-0.130	0.107	0.422	21
			Lag 3	0.037	-0.061	0.135	0.228	20
		Lag 3	Lag 0	-0.026	-0.154	0.102	0.345	20
			Lag 1	-0.004	-0.100	0.091	0.467	15
			Lag 2	-0.050	-0.174	0.073	0.212	16
			Lag 3	0.031	-0.074	0.137	0.281	15
Majority Rural (>50%)	All-natural	Lag 0	Lag 0	0.002	-0.012	0.017	0.371	1672
			Lag 1	-0.039	-0.064	-0.015	**0.001**	1332
			Lag 2	-0.028	-0.052	-0.005	**0.010**	1171
			Lag 3	-0.017	-0.048	0.015	0.149	1065
		Lag 1	Lag 0	0.005	-0.008	0.019	0.222	1747
			Lag 1	-0.025	-0.042	-0.009	**0.001**	1522
			Lag 2	-0.028	-0.050	-0.005	**0.008**	1436
			Lag 3	-0.050	-0.079	-0.021	**0.000**	1322
		Lag 2	Lag 0	0.001	-0.009	0.011	0.446	1659
			Lag 1	-0.020	-0.035	-0.006	**0.003**	1709
			Lag 2	-0.023	-0.040	-0.006	**0.004**	1635
			Lag 3	-0.043	-0.069	-0.017	**0.001**	1546
		Lag 3	Lag 0	0.006	-0.004	0.015	0.127	1756
			Lag 1	0.002	-0.007	0.012	0.321	1701
			Lag 2	-0.007	-0.021	0.007	0.178	1690
			Lag 3	-0.011	-0.027	0.005	0.095	1627
	Cardiovascular	Lag 0	Lag 0	-	-	-	-	< 10
			Lag 1	-	-	-	-	< 10
			Lag 2	-	-	-	-	< 10
			Lag 3	-	-	-	-	< 10
		Lag 1	Lag 0	-	-	-	-	< 10
			Lag 1	-	-	-	-	< 10
			Lag 2	-	-	-	-	< 10
			Lag 3	-	-	-	-	< 10
		Lag 2	Lag 0	-	-	-	-	< 10
			Lag 1	-	-	-	-	< 10
			Lag 2	-	-	-	-	< 10
			Lag 3	-	-	-	-	< 10
		Lag 3	Lag 0	-	-	-	-	< 10
			Lag 1	-	-	-	-	< 10
			Lag 2	-	-	-	-	< 10
			Lag 3	-	-	-	-	< 10
	Cerebrovascular	Lag 0	Lag 0	-	-	-	-	< 10
			Lag 1	-	-	-	-	< 10
			Lag 2	-	-	-	-	< 10
			Lag 3	-	-	-	-	< 10
		Lag 1	Lag 0	-	-	-	-	< 10
			Lag 1	-	-	-	-	< 10
			Lag 2	-	-	-	-	< 10

			Lag 3	-	-	-	-	< 10
		Lag 2	Lag 0	-	-	-	-	< 10
			Lag 1	-	-	-	-	< 10
			Lag 2	-	-	-	-	< 10
			Lag 3	-	-	-	-	< 10
		Lag 3	Lag 0	-	-	-	-	< 10
			Lag 1	-	-	-	-	< 10
			Lag 2	-	-	-	-	< 10
			Lag 3	-	-	-	-	< 10
	Renal	Lag 0	Lag 0	-	-	-	-	< 10
			Lag 1	-	-	-	-	< 10
			Lag 2	-	-	-	-	< 10
			Lag 3	-	-	-	-	< 10
		Lag 1	Lag 0	-	-	-	-	< 10
			Lag 1	-	-	-	-	< 10
			Lag 2	-	-	-	-	< 10
			Lag 3	-	-	-	-	< 10
		Lag 2	Lag 0	-	-	-	-	< 10
			Lag 1	-	-	-	-	< 10
			Lag 2	-	-	-	-	< 10
			Lag 3	-	-	-	-	< 10
		Lag 3	Lag 0	-	-	-	-	< 10
			Lag 1	-	-	-	-	< 10
			Lag 2	-	-	-	-	< 10
			Lag 3	-	-	-	-	< 10
	Respiratory	Lag 0	Lag 0	-	-	-	-	< 10
			Lag 1	-	-	-	-	< 10
			Lag 2	-	-	-	-	< 10
			Lag 3	-	-	-	-	< 10
		Lag 1	Lag 0	-	-	-	-	< 10
			Lag 1	-	-	-	-	< 10
			Lag 2	-	-	-	-	< 10
			Lag 3	-	-	-	-	< 10
		Lag 2	Lag 0	-	-	-	-	< 10
			Lag 1	-	-	-	-	< 10
			Lag 2	-	-	-	-	< 10
			Lag 3	-	-	-	-	< 10
		Lag 3	Lag 0	-	-	-	-	< 10
			Lag 1	-	-	-	-	< 10
			Lag 2	-	-	-	-	< 10
			Lag 3	-	-	-	-	< 10
Majority Urban	All-natural	Lag 0	Lag 0	0.002	0.000	0.004	**0.033**	58356
			Lag 1	0.001	-0.001	0.003	0.272	53603
			Lag 2	-0.003	-0.006	-0.001	**0.007**	53164
			Lag 3	0.001	-0.001	0.003	0.213	52713
		Lag 1	Lag 0	0.003	0.001	0.005	**0.000**	56873
			Lag 1	-0.001	-0.003	0.001	0.153	57302

		Lag 2	-0.003	-0.005	-0.001	**0.004**	57528
		Lag 3	0.001	-0.002	0.003	0.231	57647
	Lag 2	Lag 0	0.004	0.002	0.006	**0.000**	51162
		Lag 1	-0.001	-0.003	0.000	0.062	56619
		Lag 2	-0.005	-0.007	-0.003	**0.000**	56983
		Lag 3	-0.002	-0.004	0.000	**0.038**	57233
	Lag 3	Lag 0	0.004	0.002	0.007	**0.000**	52470
		Lag 1	0.002	0.000	0.003	0.052	52411
		Lag 2	-0.004	-0.006	-0.002	**0.000**	52999
		Lag 3	-0.001	-0.003	0.001	0.143	53541
Cardiovascular	Lag 0	Lag 0	0.016	-0.023	0.054	0.215	109
		Lag 1	0.017	-0.022	0.055	0.194	115
		Lag 2	0.016	-0.043	0.074	0.299	110
		Lag 3	-0.030	-0.091	0.030	0.161	107
	Lag 1	Lag 0	0.024	-0.014	0.061	0.108	109
		Lag 1	0.011	-0.025	0.047	0.276	113
		Lag 2	0.029	-0.014	0.071	0.091	111
		Lag 3	-0.006	-0.061	0.049	0.420	110
	Lag 2	Lag 0	0.050	0.010	0.090	**0.007**	111
		Lag 1	-0.012	-0.045	0.022	0.245	121
		Lag 2	-0.008	-0.049	0.034	0.355	117
		Lag 3	-0.014	-0.056	0.028	0.261	113
	Lag 3	Lag 0	0.058	0.011	0.105	**0.008**	128
		Lag 1	-0.006	-0.042	0.029	0.359	107
		Lag 2	-0.018	-0.059	0.022	0.187	105
		Lag 3	-0.006	-0.047	0.034	0.378	102
Cerebrovascular	Lag 0	Lag 0	-0.002	-0.035	0.032	0.463	176
		Lag 1	-0.002	-0.036	0.031	0.446	174
		Lag 2	0.016	-0.030	0.063	0.243	176
		Lag 3	0.027	-0.018	0.071	0.122	180
	Lag 1	Lag 0	0.010	-0.023	0.042	0.276	187
		Lag 1	0.005	-0.024	0.034	0.370	202
		Lag 2	0.005	-0.032	0.042	0.392	209
		Lag 3	0.012	-0.033	0.057	0.302	214
	Lag 2	Lag 0	0.011	-0.026	0.048	0.283	154
		Lag 1	0.011	-0.014	0.035	0.200	199
		Lag 2	-0.006	-0.038	0.026	0.352	199
		Lag 3	0.017	-0.016	0.050	0.153	204
	Lag 3	Lag 0	0.028	-0.012	0.068	0.084	166
		Lag 1	0.010	-0.017	0.038	0.228	173
		Lag 2	0.009	-0.016	0.034	0.237	174
		Lag 3	0.020	-0.008	0.048	0.081	175
Renal	Lag 0	Lag 0	0.029	-0.022	0.080	0.136	67
		Lag 1	-0.022	-0.094	0.050	0.278	62
		Lag 2	0.027	-0.053	0.107	0.251	61
		Lag 3	0.066	-0.018	0.150	0.063	62
	Lag 1	Lag 0	0.034	-0.014	0.081	0.081	64

			Lag 1	-0.012	-0.078	0.054	0.363	66
			Lag 2	-0.020	-0.099	0.060	0.315	70
			Lag 3	0.069	-0.004	0.143	**0.031**	72
		Lag 2	Lag 0	-0.004	-0.066	0.059	0.455	49
			Lag 1	-0.006	-0.074	0.062	0.427	57
			Lag 2	0.007	-0.058	0.072	0.422	61
			Lag 3	0.052	-0.003	0.108	**0.032**	64
		Lag 3	Lag 0	-0.053	-0.139	0.034	0.116	54
			Lag 1	-0.025	-0.115	0.064	0.288	56
			Lag 2	0.007	-0.048	0.062	0.400	60
			Lag 3	0.017	-0.032	0.066	0.251	59
	Respiratory	Lag 0	Lag 0	0.013	-0.048	0.074	0.343	83
			Lag 1	0.034	-0.023	0.091	0.123	73
			Lag 2	0.014	-0.054	0.083	0.341	75
			Lag 3	0.078	0.020	0.135	**0.004**	73
		Lag 1	Lag 0	-0.004	-0.067	0.060	0.454	77
			Lag 1	0.041	-0.009	0.092	0.055	78
			Lag 2	-0.001	-0.057	0.055	0.487	79
			Lag 3	0.080	0.019	0.140	**0.005**	78
		Lag 2	Lag 0	0.056	0.007	0.106	**0.013**	96
			Lag 1	0.039	0.002	0.076	**0.019**	89
			Lag 2	0.002	-0.051	0.056	0.464	94
			Lag 3	0.041	-0.017	0.098	0.082	91
		Lag 3	Lag 0	0.007	-0.056	0.071	0.412	99
			Lag 1	-0.017	-0.076	0.042	0.289	60
			Lag 2	-0.013	-0.071	0.045	0.332	69
			Lag 3	0.032	-0.020	0.084	0.112	69

Table S4 Stratified results for multiplicative odds ratio with upper and lower limits (UL and LL, respectively) for 95% confidence intervals (CIs) and p-values (bolded indicates significant $p < 0.05$) for the joint effects of wildfire smoke and extreme heat. Subgroups of individual and community level factors were tested in stratified models. Separate models were run for combinations of lag 0-3 and each outcome of interest: all-natural cause, cardiovascular, cerebrovascular, renal, and respiratory morbidity. Results with fewer than 10 exposed cases are suppressed.

Subgroup	OOI	Extreme Heat	Wildfire Smoke	Estimate	CI LL	CI UL	P-value	Exposed Case Count
Male	All-natural	Lag 0	Lag 0	1.003	1.000	1.006	0.056	26016
			Lag 1	1.000	0.997	1.003	0.882	23718
			Lag 2	0.999	0.995	1.003	0.633	23468
			Lag 3	1.002	0.998	1.006	0.263	23296
		Lag 1	Lag 0	1.005	1.002	1.007	**0.000**	25713
			Lag 1	0.998	0.996	1.001	0.243	25405
			Lag 2	0.999	0.996	1.002	0.395	25439
			Lag 3	1.002	0.998	1.006	0.248	25484
		Lag 2	Lag 0	1.006	1.003	1.009	**0.000**	23047
			Lag 1	0.998	0.996	1.001	0.175	25442
			Lag 2	0.997	0.994	1.000	**0.027**	25563
			Lag 3	0.999	0.996	1.002	0.465	25702
		Lag 3	Lag 0	1.007	1.004	1.011	**0.000**	23314
			Lag 1	1.002	0.999	1.005	0.188	23420
			Lag 2	0.996	0.994	0.999	**0.003**	23683
			Lag 3	1.000	0.997	1.002	0.758	23935
	Cardiovascular	Lag 0	Lag 0	0.995	0.945	1.048	0.864	55
			Lag 1	1.031	0.982	1.083	0.215	63
			Lag 2	1.006	0.927	1.092	0.886	59
			Lag 3	0.989	0.925	1.057	0.739	56
		Lag 1	Lag 0	1.005	0.951	1.061	0.868	57
			Lag 1	1.025	0.978	1.074	0.300	62
			Lag 2	1.041	0.981	1.106	0.183	59
			Lag 3	0.972	0.903	1.045	0.439	58
		Lag 2	Lag 0	1.017	0.954	1.084	0.615	58
			Lag 1	1.014	0.973	1.056	0.519	71
			Lag 2	1.002	0.948	1.059	0.948	67
			Lag 3	0.994	0.943	1.047	0.822	63
		Lag 3	Lag 0	1.016	0.944	1.092	0.675	69
			Lag 1	1.018	0.977	1.060	0.394	60
			Lag 2	0.974	0.922	1.028	0.334	54
			Lag 3	1.020	0.973	1.070	0.403	51
	Cerebrovascular	Lag 0	Lag 0	0.987	0.940	1.036	0.607	94

			Lag 1	0.981	0.928	1.037	0.494	92
			Lag 2	1.049	0.984	1.118	0.140	91
			Lag 3	1.011	0.946	1.080	0.744	93
		Lag 1	Lag 0	1.001	0.955	1.049	0.965	99
			Lag 1	0.993	0.948	1.040	0.758	99
			Lag 2	0.985	0.933	1.041	0.595	99
			Lag 3	0.984	0.921	1.052	0.636	101
		Lag 2	Lag 0	0.963	0.901	1.028	0.259	75
			Lag 1	1.004	0.968	1.042	0.815	106
			Lag 2	0.995	0.957	1.035	0.807	105
			Lag 3	0.991	0.945	1.038	0.698	108
		Lag 3	Lag 0	0.998	0.943	1.056	0.941	87
			Lag 1	1.012	0.975	1.050	0.537	102
			Lag 2	1.019	0.989	1.050	0.210	103
			Lag 3	1.006	0.968	1.046	0.748	104
	Renal	Lag 0	Lag 0	1.031	0.957	1.111	0.415	32
			Lag 1	0.981	0.888	1.084	0.707	36
			Lag 2	1.064	0.963	1.175	0.222	34
			Lag 3	1.000	0.861	1.161	0.997	34
		Lag 1	Lag 0	1.037	0.960	1.121	0.357	28
			Lag 1	0.976	0.886	1.074	0.617	36
			Lag 2	0.955	0.846	1.079	0.461	36
			Lag 3	1.084	0.967	1.215	0.164	36
		Lag 2	Lag 0	0.989	0.901	1.084	0.810	17
			Lag 1	0.961	0.867	1.065	0.448	26
			Lag 2	0.979	0.890	1.077	0.666	27
			Lag 3	1.028	0.942	1.122	0.538	27
		Lag 3	Lag 0	0.890	0.775	1.023	0.102	23
			Lag 1	0.964	0.843	1.101	0.586	28
			Lag 2	1.014	0.919	1.120	0.775	29
			Lag 3	1.028	0.944	1.119	0.531	28
	Respiratory	Lag 0	Lag 0	1.047	0.951	1.152	0.348	39
			Lag 1	1.096	1.010	1.190	**0.028**	40
			Lag 2	1.064	0.969	1.168	0.192	40
			Lag 3	1.193	1.079	1.320	**0.001**	39
		Lag 1	Lag 0	0.958	0.855	1.074	0.461	29
			Lag 1	1.064	0.987	1.146	0.105	43
			Lag 2	1.007	0.939	1.080	0.846	43
			Lag 3	1.143	1.034	1.264	**0.009**	42
		Lag 2	Lag 0	1.084	0.999	1.176	0.054	42
			Lag 1	1.034	0.974	1.098	0.274	43
			Lag 2	1.002	0.933	1.076	0.957	46

			Lag 3	1.057	0.962	1.160	0.248	44
		Lag 3	Lag 0	1.037	0.946	1.137	0.437	43
			Lag 1	1.018	0.980	1.058	0.361	30
			Lag 2	0.968	0.881	1.063	0.495	33
			Lag 3	1.029	0.927	1.142	0.594	31
Female	All-natural	Lag 0	Lag 0	1.001	0.999	1.004	0.406	34010
			Lag 1	1.001	0.998	1.004	0.576	31215
			Lag 2	0.994	0.991	0.998	**0.001**	30865
			Lag 3	1.000	0.997	1.003	0.976	30480
		Lag 1	Lag 0	1.002	0.999	1.004	0.196	32905
			Lag 1	0.999	0.997	1.001	0.412	33417
			Lag 2	0.996	0.993	0.998	**0.002**	33523
			Lag 3	0.999	0.996	1.003	0.637	33483
		Lag 2	Lag 0	1.002	0.999	1.004	0.176	29774
			Lag 1	0.999	0.996	1.001	0.199	32884
			Lag 2	0.994	0.991	0.996	**0.000**	33052
			Lag 3	0.997	0.995	1.000	**0.042**	33075
		Lag 3	Lag 0	1.002	0.999	1.005	0.107	30912
			Lag 1	1.001	0.999	1.004	0.277	30692
			Lag 2	0.996	0.994	0.998	**0.000**	31006
			Lag 3	0.998	0.996	1.001	0.193	31233
	Cardiovascular	Lag 0	Lag 0	1.044	0.982	1.111	0.170	56
			Lag 1	0.986	0.917	1.061	0.712	52
			Lag 2	1.019	0.935	1.111	0.666	51
			Lag 3	0.911	0.809	1.025	0.121	51
		Lag 1	Lag 0	1.046	0.989	1.107	0.112	54
			Lag 1	0.987	0.926	1.052	0.678	53
			Lag 2	1.018	0.954	1.087	0.582	53
			Lag 3	1.031	0.947	1.122	0.486	53
		Lag 2	Lag 0	1.084	1.022	1.149	**0.007**	55
			Lag 1	0.955	0.894	1.020	0.171	55
			Lag 2	0.989	0.931	1.051	0.728	55
			Lag 3	0.978	0.911	1.050	0.533	55
		Lag 3	Lag 0	1.109	1.027	1.197	**0.008**	63
			Lag 1	0.970	0.909	1.034	0.351	52
			Lag 2	0.991	0.933	1.053	0.779	53
			Lag 3	0.968	0.901	1.041	0.386	53
	Cerebrovascular	Lag 0	Lag 0	1.011	0.965	1.059	0.639	89
			Lag 1	1.010	0.969	1.053	0.633	87
			Lag 2	0.980	0.913	1.052	0.576	90
			Lag 3	1.046	0.982	1.115	0.159	91
		Lag 1	Lag 0	1.019	0.974	1.067	0.414	95

			Lag 1	1.016	0.979	1.054	0.412	110
			Lag 2	1.020	0.967	1.075	0.469	116
			Lag 3	1.035	0.972	1.102	0.284	117
		Lag 2	Lag 0	1.041	0.993	1.091	0.094	85
			Lag 1	1.015	0.981	1.050	0.394	98
			Lag 2	0.989	0.938	1.042	0.673	102
			Lag 3	1.047	0.997	1.101	0.067	102
		Lag 3	Lag 0	1.071	1.009	1.137	**0.025**	86
			Lag 1	1.011	0.970	1.054	0.602	76
			Lag 2	0.985	0.937	1.036	0.564	79
			Lag 3	1.038	0.994	1.085	0.094	76
	Renal	Lag 0	Lag 0	1.029	0.955	1.108	0.456	39
			Lag 1	0.983	0.891	1.085	0.740	28
			Lag 2	1.001	0.888	1.128	0.988	28
			Lag 3	1.103	0.987	1.234	0.085	29
		Lag 1	Lag 0	1.035	0.970	1.104	0.298	38
			Lag 1	0.993	0.906	1.088	0.877	31
			Lag 2	0.981	0.884	1.089	0.719	34
			Lag 3	1.051	0.945	1.169	0.361	36
		Lag 2	Lag 0	1.014	0.925	1.112	0.761	33
			Lag 1	1.019	0.925	1.123	0.705	32
			Lag 2	1.024	0.936	1.120	0.611	34
			Lag 3	1.073	0.992	1.161	0.080	38
		Lag 3	Lag 0	1.000	0.902	1.108	0.999	33
			Lag 1	0.980	0.872	1.102	0.735	29
			Lag 2	1.003	0.936	1.075	0.934	32
			Lag 3	1.014	0.952	1.081	0.664	32
	Respiratory	Lag 0	Lag 0	1.009	0.926	1.099	0.838	46
			Lag 1	0.981	0.894	1.076	0.683	36
			Lag 2	0.938	0.843	1.042	0.232	36
			Lag 3	1.024	0.941	1.114	0.586	35
		Lag 1	Lag 0	1.039	0.960	1.123	0.343	49
			Lag 1	1.022	0.949	1.100	0.571	38
			Lag 2	0.967	0.881	1.060	0.473	38
			Lag 3	1.049	0.961	1.145	0.283	37
		Lag 2	Lag 0	1.069	0.994	1.149	0.071	54
			Lag 1	1.046	0.994	1.101	0.083	48
			Lag 2	0.989	0.911	1.074	0.792	49
			Lag 3	1.037	0.959	1.121	0.360	47
		Lag 3	Lag 0	0.995	0.906	1.092	0.913	56
			Lag 1	1.008	0.944	1.077	0.803	35
			Lag 2	0.988	0.919	1.062	0.744	39

			Lag 3	1.036	0.972	1.104	0.273	39
Age 18-49 year	All-natural	Lag 0	Lag 0	1.001	0.998	1.004	0.468	21442
			Lag 1	0.998	0.994	1.002	0.263	19677
			Lag 2	0.992	0.988	0.997	**0.000**	19427
			Lag 3	1.000	0.995	1.004	0.855	19164
		Lag 1	Lag 0	1.001	0.998	1.004	0.349	20621
			Lag 1	0.996	0.993	0.999	**0.008**	20970
			Lag 2	0.992	0.988	0.995	**0.000**	21004
			Lag 3	1.000	0.996	1.004	0.947	20948
		Lag 2	Lag 0	1.002	0.999	1.005	0.207	18757
			Lag 1	0.998	0.995	1.001	0.138	20318
			Lag 2	0.991	0.988	0.994	**0.000**	20397
			Lag 3	1.000	0.997	1.004	0.829	20392
		Lag 3	Lag 0	1.002	0.998	1.006	0.292	19793
			Lag 1	1.003	1.000	1.006	0.091	19032
			Lag 2	0.993	0.990	0.996	**0.000**	19163
			Lag 3	1.000	0.997	1.003	0.975	19285
	Cardiovascular	Lag 0	Lag 0	0.945	0.817	1.092	0.440	14
			Lag 1	0.858	0.684	1.076	0.186	11
			Lag 2	0.915	0.754	1.110	0.369	10
			Lag 3	0.879	0.714	1.084	0.228	10
		Lag 1	Lag 0	0.970	0.868	1.083	0.589	17
			Lag 1	0.968	0.870	1.077	0.549	14
			Lag 2	0.921	0.787	1.077	0.304	13
			Lag 3	0.885	0.737	1.063	0.192	14
		Lag 2	Lag 0	0.984	0.882	1.098	0.773	18
			Lag 1	0.964	0.883	1.053	0.416	16
			Lag 2	0.934	0.800	1.090	0.386	17
			Lag 3	0.860	0.705	1.049	0.137	17
		Lag 3	Lag 0	0.975	0.838	1.134	0.740	16
			Lag 1	0.950	0.854	1.057	0.348	13
			Lag 2	0.921	0.796	1.067	0.274	13
			Lag 3	0.949	0.834	1.080	0.429	14
	Cerebrovascular	Lag 0	Lag 0	1.045	0.953	1.145	0.351	16
			Lag 1	1.038	0.928	1.161	0.511	16
			Lag 2	1.123	0.993	1.270	0.064	16
			Lag 3	1.064	0.940	1.203	0.328	16
		Lag 1	Lag 0	1.057	0.960	1.163	0.259	18
			Lag 1	1.048	0.947	1.159	0.365	21
			Lag 2	1.005	0.896	1.128	0.928	22
			Lag 3	0.939	0.791	1.114	0.470	22
		Lag 2	Lag 0	1.073	0.950	1.212	0.256	16

			Lag 1	1.019	0.924	1.125	0.703	22
			Lag 2	0.972	0.867	1.089	0.620	24
			Lag 3	1.001	0.883	1.134	0.989	22
		Lag 3	Lag 0	0.913	0.730	1.142	0.427	10
			Lag 1	0.943	0.829	1.073	0.373	13
			Lag 2	1.011	0.892	1.146	0.862	14
			Lag 3	0.973	0.853	1.111	0.687	11
	Renal	Lag 0	Lag 0	-	-	-	-	< 10
			Lag 1	-	-	-	-	< 10
			Lag 2	-	-	-	-	< 10
			Lag 3	-	-	-	-	< 10
		Lag 1	Lag 0	-	-	-	-	< 10
			Lag 1	-	-	-	-	< 10
			Lag 2	-	-	-	-	< 10
			Lag 3	-	-	-	-	< 10
		Lag 2	Lag 0	-	-	-	-	< 10
			Lag 1	-	-	-	-	< 10
			Lag 2	-	-	-	-	< 10
			Lag 3	-	-	-	-	< 10
		Lag 3	Lag 0	0.962	0.825	1.122	0.619	10
			Lag 1	-	-	-	-	< 10
			Lag 2	-	-	-	-	< 10
			Lag 3	-	-	-	-	< 10
	Respiratory	Lag 0	Lag 0	1.112	0.952	1.297	0.180	18
			Lag 1	1.013	0.861	1.193	0.872	12
			Lag 2	1.033	0.816	1.307	0.790	12
			Lag 3	1.125	0.955	1.325	0.158	11
		Lag 1	Lag 0	1.053	0.883	1.255	0.565	18
			Lag 1	1.072	0.926	1.240	0.352	16
			Lag 2	1.036	0.888	1.207	0.655	16
			Lag 3	1.149	0.978	1.349	0.091	16
		Lag 2	Lag 0	1.171	1.027	1.335	**0.018**	24
			Lag 1	1.001	0.896	1.117	0.992	15
			Lag 2	1.020	0.868	1.200	0.807	16
			Lag 3	0.983	0.845	1.143	0.820	16
		Lag 3	Lag 0	0.961	0.821	1.126	0.625	20
			Lag 1	-	-	-	-	< 10
			Lag 2	0.988	0.872	1.120	0.853	10
			Lag 3	0.976	0.836	1.139	0.760	10
Age 50-64 year	All-natural	Lag 0	Lag 0	1.001	0.997	1.005	0.636	14664
			Lag 1	0.998	0.994	1.003	0.439	13520
			Lag 2	0.999	0.994	1.005	0.844	13405

		Lag 3	1.002	0.997	1.007	0.411	13277
	Lag 1	Lag 0	1.002	0.999	1.006	0.183	14349
		Lag 1	0.997	0.993	1.001	0.129	14309
		Lag 2	1.000	0.995	1.004	0.825	14351
		Lag 3	1.002	0.997	1.007	0.432	14369
	Lag 2	Lag 0	1.001	0.997	1.005	0.626	12914
		Lag 1	0.994	0.991	0.997	**0.001**	14183
		Lag 2	0.994	0.990	0.997	**0.001**	14260
		Lag 3	0.998	0.994	1.002	0.349	14293
	Lag 3	Lag 0	1.002	0.997	1.007	0.386	13140
		Lag 1	0.998	0.994	1.002	0.353	13361
		Lag 2	0.996	0.993	1.000	**0.026**	13545
		Lag 3	0.999	0.995	1.002	0.490	13639
Cardiovascular	Lag 0	Lag 0	0.988	0.906	1.078	0.792	23
		Lag 1	1.033	0.942	1.132	0.489	36
		Lag 2	1.025	0.917	1.145	0.666	34
		Lag 3	0.961	0.860	1.074	0.485	33
	Lag 1	Lag 0	0.959	0.871	1.055	0.389	26
		Lag 1	1.021	0.933	1.116	0.655	29
		Lag 2	1.039	0.957	1.128	0.360	29
		Lag 3	0.964	0.871	1.066	0.475	29
	Lag 2	Lag 0	1.023	0.931	1.126	0.632	24
		Lag 1	0.969	0.893	1.051	0.445	32
		Lag 2	0.997	0.921	1.078	0.931	31
		Lag 3	0.967	0.889	1.052	0.438	31
	Lag 3	Lag 0	1.065	0.954	1.189	0.260	37
		Lag 1	0.956	0.866	1.054	0.364	28
		Lag 2	0.980	0.913	1.053	0.586	28
		Lag 3	0.985	0.911	1.065	0.702	29
Cerebrovascular	Lag 0	Lag 0	0.965	0.886	1.050	0.409	42
		Lag 1	0.999	0.927	1.077	0.980	47
		Lag 2	1.025	0.934	1.125	0.605	48
		Lag 3	1.069	0.987	1.157	0.104	47
	Lag 1	Lag 0	0.984	0.909	1.064	0.680	49
		Lag 1	0.963	0.893	1.039	0.336	45
		Lag 2	0.974	0.900	1.055	0.517	45
		Lag 3	1.071	0.992	1.157	0.080	47
	Lag 2	Lag 0	1.020	0.946	1.099	0.611	41
		Lag 1	0.990	0.939	1.044	0.718	52
		Lag 2	1.017	0.961	1.076	0.559	51
		Lag 3	1.045	0.989	1.105	0.117	53
	Lag 3	Lag 0	1.066	0.990	1.149	0.091	55

			Lag 1	0.986	0.928	1.047	0.644	52
			Lag 2	1.010	0.967	1.056	0.644	53
			Lag 3	1.031	0.980	1.083	0.236	54
	Renal	Lag 0	Lag 0	1.045	0.938	1.164	0.429	16
			Lag 1	0.957	0.830	1.103	0.543	16
			Lag 2	1.126	0.946	1.339	0.181	15
			Lag 3	1.184	0.967	1.449	0.102	15
		Lag 1	Lag 0	1.034	0.945	1.132	0.468	11
			Lag 1	1.056	0.953	1.169	0.297	19
			Lag 2	1.137	0.959	1.348	0.139	20
			Lag 3	1.060	0.893	1.259	0.504	21
		Lag 2	Lag 0	-	-	-	-	< 10
			Lag 1	1.070	0.966	1.187	0.196	20
			Lag 2	0.977	0.835	1.143	0.774	21
			Lag 3	1.028	0.869	1.217	0.746	22
		Lag 3	Lag 0	-	-	-	-	< 10
			Lag 1	1.041	0.891	1.216	0.611	15
			Lag 2	0.947	0.809	1.108	0.498	17
			Lag 3	1.010	0.867	1.177	0.897	17
	Respiratory	Lag 0	Lag 0	1.002	0.912	1.101	0.969	30
			Lag 1	1.126	1.011	1.255	**0.031**	31
			Lag 2	0.982	0.886	1.090	0.739	30
			Lag 3	1.132	1.006	1.274	**0.040**	30
		Lag 1	Lag 0	1.006	0.919	1.102	0.896	28
			Lag 1	1.055	0.955	1.165	0.292	26
			Lag 2	0.952	0.860	1.053	0.341	26
			Lag 3	1.009	0.891	1.142	0.888	26
		Lag 2	Lag 0	1.076	0.991	1.167	0.079	35
			Lag 1	1.047	0.964	1.138	0.272	35
			Lag 2	0.989	0.900	1.086	0.811	36
			Lag 3	1.057	0.954	1.172	0.292	37
		Lag 3	Lag 0	1.025	0.924	1.137	0.638	34
			Lag 1	1.002	0.907	1.107	0.971	23
			Lag 2	0.933	0.823	1.058	0.282	24
			Lag 3	0.988	0.895	1.090	0.811	25
Age 65+ year	All-natural	Lag 0	Lag 0	1.003	1.000	1.006	0.054	23923
			Lag 1	1.004	1.000	1.007	**0.032**	21739
			Lag 2	0.998	0.994	1.002	0.421	21503
			Lag 3	1.001	0.998	1.005	0.465	21337
		Lag 1	Lag 0	1.005	1.002	1.007	**0.000**	23651
			Lag 1	1.002	0.999	1.005	0.150	23546
			Lag 2	1.000	0.997	1.003	0.948	23609

		Lag 3	1.000	0.996	1.004	0.993	23652
	Lag 2	Lag 0	1.006	1.003	1.009	**0.000**	21151
		Lag 1	1.001	0.999	1.004	0.323	23829
		Lag 2	0.999	0.996	1.002	0.570	23961
		Lag 3	0.996	0.993	0.999	**0.009**	24095
	Lag 3	Lag 0	1.008	1.005	1.011	**0.000**	21294
		Lag 1	1.002	1.000	1.005	0.078	21721
		Lag 2	0.998	0.996	1.001	0.166	21982
		Lag 3	0.998	0.995	1.001	0.205	22245
Cardiovascular	Lag 0	Lag 0	1.036	0.987	1.088	0.153	74
		Lag 1	1.025	0.978	1.074	0.301	68
		Lag 2	1.026	0.950	1.108	0.519	66
		Lag 3	0.986	0.916	1.061	0.705	64
	Lag 1	Lag 0	1.060	1.009	1.114	**0.020**	68
		Lag 1	1.010	0.966	1.057	0.646	72
		Lag 2	1.043	0.987	1.103	0.133	70
		Lag 3	1.038	0.965	1.117	0.314	68
	Lag 2	Lag 0	1.080	1.022	1.141	**0.006**	71
		Lag 1	0.999	0.960	1.039	0.952	78
		Lag 2	1.003	0.954	1.054	0.906	74
		Lag 3	1.011	0.960	1.064	0.689	70
	Lag 3	Lag 0	1.072	1.008	1.140	**0.028**	79
		Lag 1	1.014	0.976	1.053	0.480	71
		Lag 2	0.993	0.942	1.047	0.793	66
		Lag 3	1.016	0.967	1.067	0.535	61
Cerebrovascular	Lag 0	Lag 0	1.001	0.962	1.042	0.952	125
		Lag 1	0.995	0.957	1.035	0.814	116
		Lag 2	0.989	0.930	1.052	0.727	117
		Lag 3	1.003	0.942	1.068	0.921	121
	Lag 1	Lag 0	1.012	0.973	1.052	0.556	127
		Lag 1	1.012	0.980	1.046	0.458	143
		Lag 2	1.012	0.967	1.059	0.604	148
		Lag 3	0.989	0.931	1.050	0.718	149
	Lag 2	Lag 0	0.998	0.952	1.046	0.930	103
		Lag 1	1.016	0.986	1.046	0.295	130
		Lag 2	0.986	0.947	1.026	0.481	132
		Lag 3	1.002	0.957	1.048	0.944	135
	Lag 3	Lag 0	1.023	0.972	1.077	0.380	108
		Lag 1	1.030	0.996	1.064	0.084	113
		Lag 2	1.011	0.979	1.044	0.512	115
		Lag 3	1.020	0.983	1.058	0.287	115
Renal	Lag 0	Lag 0	1.033	0.970	1.100	0.317	48

			Lag 1	1.048	0.958	1.147	0.304	42
			Lag 2	1.028	0.940	1.123	0.547	41
			Lag 3	1.001	0.885	1.132	0.991	42
		Lag 1	Lag 0	1.072	1.004	1.144	**0.036**	51
			Lag 1	0.982	0.895	1.078	0.708	43
			Lag 2	0.945	0.856	1.043	0.260	45
			Lag 3	1.052	0.946	1.169	0.347	46
		Lag 2	Lag 0	1.055	0.981	1.135	0.148	34
			Lag 1	0.984	0.880	1.101	0.779	31
			Lag 2	1.004	0.928	1.086	0.920	33
			Lag 3	1.049	0.971	1.132	0.223	36
		Lag 3	Lag 0	0.976	0.876	1.088	0.665	37
			Lag 1	0.980	0.872	1.103	0.742	34
			Lag 2	1.019	0.953	1.090	0.583	35
			Lag 3	1.011	0.953	1.073	0.715	34
	Respiratory	Lag 0	Lag 0	0.978	0.885	1.081	0.663	37
			Lag 1	1.002	0.922	1.089	0.958	33
			Lag 2	0.999	0.903	1.106	0.986	34
			Lag 3	1.062	0.977	1.153	0.157	33
		Lag 1	Lag 0	0.960	0.866	1.064	0.435	32
			Lag 1	1.029	0.962	1.102	0.406	39
			Lag 2	0.999	0.924	1.080	0.983	39
			Lag 3	1.115	1.020	1.218	**0.017**	37
		Lag 2	Lag 0	0.993	0.907	1.088	0.880	37
			Lag 1	1.050	0.998	1.104	0.058	41
			Lag 2	0.992	0.921	1.068	0.830	43
			Lag 3	1.059	0.971	1.156	0.194	38
		Lag 3	Lag 0	1.005	0.911	1.108	0.920	45
			Lag 1	1.044	0.977	1.116	0.205	33
			Lag 2	1.001	0.929	1.078	0.989	38
			Lag 3	1.073	0.998	1.154	0.058	35
English, preferred language	All-natural	Lag 0	Lag 0	1.002	1.000	1.004	0.127	51173
			Lag 1	0.999	0.997	1.002	0.517	46427
			Lag 2	0.995	0.992	0.998	**0.001**	45897
			Lag 3	1.001	0.999	1.004	0.375	45400
		Lag 1	Lag 0	1.003	1.001	1.005	**0.002**	50170
			Lag 1	0.997	0.995	0.999	**0.010**	50102
			Lag 2	0.996	0.994	0.998	**0.000**	50221
			Lag 3	1.000	0.998	1.003	0.831	50217
		Lag 2	Lag 0	1.004	1.002	1.006	**0.000**	45298
			Lag 1	0.998	0.996	0.999	**0.009**	49702
			Lag 2	0.994	0.992	0.996	**0.000**	49944

		Lag 3	0.998	0.995	1.000	**0.028**	50088
	Lag 3	Lag 0	1.004	1.002	1.006	**0.001**	46350
		Lag 1	1.002	1.000	1.004	0.122	46285
		Lag 2	0.995	0.994	0.997	**0.000**	46775
		Lag 3	0.999	0.997	1.001	0.575	47171
Cardiovascular	Lag 0	Lag 0	1.017	0.977	1.059	0.400	97
		Lag 1	1.009	0.968	1.052	0.677	100
		Lag 2	1.014	0.950	1.082	0.676	96
		Lag 3	0.962	0.900	1.027	0.242	94
	Lag 1	Lag 0	1.021	0.979	1.065	0.325	94
		Lag 1	1.000	0.960	1.042	0.991	99
		Lag 2	1.024	0.974	1.077	0.360	96
		Lag 3	0.999	0.940	1.062	0.967	96
	Lag 2	Lag 0	1.052	1.005	1.102	**0.029**	97
		Lag 1	0.984	0.948	1.022	0.405	111
		Lag 2	0.985	0.941	1.032	0.529	107
		Lag 3	0.981	0.936	1.028	0.416	104
	Lag 3	Lag 0	1.056	0.998	1.118	0.058	114
		Lag 1	0.992	0.956	1.029	0.663	95
		Lag 2	0.973	0.930	1.018	0.232	90
		Lag 3	0.993	0.951	1.036	0.734	88
Cerebrovascular	Lag 0	Lag 0	0.996	0.958	1.035	0.829	143
		Lag 1	1.012	0.977	1.048	0.500	146
		Lag 2	1.015	0.962	1.070	0.593	147
		Lag 3	1.010	0.954	1.069	0.732	149
	Lag 1	Lag 0	1.017	0.979	1.057	0.378	155
		Lag 1	1.018	0.987	1.051	0.248	172
		Lag 2	1.013	0.972	1.055	0.536	177
		Lag 3	0.998	0.946	1.054	0.950	178
	Lag 2	Lag 0	1.011	0.969	1.055	0.615	128
		Lag 1	1.022	0.995	1.050	0.115	167
		Lag 2	1.007	0.974	1.041	0.685	170
		Lag 3	1.010	0.971	1.050	0.628	171
	Lag 3	Lag 0	1.019	0.971	1.069	0.448	136
		Lag 1	1.021	0.991	1.053	0.166	150
		Lag 2	1.002	0.971	1.034	0.898	152
		Lag 3	1.010	0.977	1.045	0.554	149
Renal	Lag 0	Lag 0	1.027	0.974	1.083	0.317	61
		Lag 1	0.996	0.929	1.069	0.919	56
		Lag 2	1.050	0.969	1.139	0.232	54
		Lag 3	1.079	0.980	1.189	0.123	55
	Lag 1	Lag 0	1.041	0.990	1.094	0.116	55

			Lag 1	0.973	0.905	1.045	0.450	55
			Lag 2	0.969	0.890	1.055	0.470	58
			Lag 3	1.074	0.985	1.172	0.107	60
		Lag 2	Lag 0	0.991	0.930	1.056	0.787	41
			Lag 1	0.978	0.908	1.053	0.554	48
			Lag 2	1.019	0.952	1.091	0.581	50
			Lag 3	1.076	1.009	1.147	**0.025**	54
		Lag 3	Lag 0	0.937	0.858	1.023	0.144	48
			Lag 1	0.979	0.894	1.072	0.651	47
			Lag 2	0.995	0.936	1.059	0.886	50
			Lag 3	1.032	0.979	1.087	0.242	49
	Respiratory	Lag 0	Lag 0	1.014	0.948	1.085	0.680	75
			Lag 1	1.052	0.991	1.118	0.098	68
			Lag 2	0.999	0.928	1.076	0.984	68
			Lag 3	1.143	1.063	1.228	**0.000**	66
		Lag 1	Lag 0	0.968	0.898	1.044	0.399	66
			Lag 1	1.038	0.981	1.098	0.200	71
			Lag 2	0.992	0.932	1.055	0.792	71
			Lag 3	1.095	1.020	1.175	**0.012**	69
		Lag 2	Lag 0	1.054	0.994	1.118	0.077	82
			Lag 1	1.047	1.003	1.093	**0.038**	80
			Lag 2	1.004	0.948	1.062	0.899	84
			Lag 3	1.040	0.976	1.108	0.224	80
		Lag 3	Lag 0	0.999	0.932	1.070	0.970	82
			Lag 1	1.022	0.984	1.061	0.260	60
			Lag 2	0.978	0.921	1.039	0.475	67
			Lag 3	1.024	0.968	1.083	0.413	65
Spanish, preferred language	All-natural	Lag 0	Lag 0	0.999	0.993	1.005	0.806	6227
			Lag 1	0.999	0.993	1.006	0.824	6083
			Lag 2	0.999	0.992	1.006	0.752	6037
			Lag 3	0.997	0.991	1.004	0.455	6006
		Lag 1	Lag 0	0.999	0.993	1.005	0.689	5960
			Lag 1	0.997	0.991	1.003	0.380	6011
			Lag 2	0.997	0.991	1.003	0.341	6027
			Lag 3	1.000	0.994	1.007	0.911	6040
		Lag 2	Lag 0	0.995	0.989	1.002	0.172	5296
			Lag 1	0.998	0.992	1.004	0.485	5983
			Lag 2	0.995	0.989	1.001	0.093	6018
			Lag 3	1.000	0.994	1.006	0.948	6030
		Lag 3	Lag 0	1.008	1.000	1.015	**0.044**	5701
			Lag 1	1.002	0.995	1.008	0.594	5518
			Lag 2	0.995	0.989	1.001	0.077	5559

			Lag 3	0.995	0.989	1.001	0.121	5605
	Cardiovascular	Lag 0	Lag 0	1.012	0.832	1.232	0.903	10
			Lag 1	-	-	-	-	< 10
			Lag 2	-	-	-	-	< 10
			Lag 3	-	-	-	-	< 10
		Lag 1	Lag 0	0.982	0.820	1.177	0.847	13
			Lag 1	1.046	0.908	1.204	0.535	10
			Lag 2	1.011	0.894	1.142	0.864	10
			Lag 3	-	-	-	-	< 10
		Lag 2	Lag 0	0.959	0.783	1.175	0.688	11
			Lag 1	-	-	-	-	< 10
			Lag 2	-	-	-	-	< 10
			Lag 3	-	-	-	-	< 10
		Lag 3	Lag 0	1.044	0.843	1.294	0.691	14
			Lag 1	-	-	-	-	< 10
			Lag 2	-	-	-	-	< 10
			Lag 3	-	-	-	-	< 10
	Cerebrovascular	Lag 0	Lag 0	0.950	0.840	1.075	0.419	21
			Lag 1	0.935	0.825	1.060	0.294	24
			Lag 2	1.017	0.894	1.158	0.796	25
			Lag 3	1.084	0.991	1.186	0.076	25
		Lag 1	Lag 0	1.028	0.930	1.136	0.588	22
			Lag 1	0.969	0.864	1.086	0.588	22
			Lag 2	0.962	0.843	1.098	0.564	23
			Lag 3	1.086	0.990	1.192	0.081	24
		Lag 2	Lag 0	1.034	0.910	1.175	0.607	22
			Lag 1	0.959	0.866	1.062	0.425	24
			Lag 2	0.932	0.824	1.054	0.262	25
			Lag 3	1.070	0.983	1.165	0.119	26
		Lag 3	Lag 0	1.120	0.977	1.285	0.103	25
			Lag 1	0.957	0.857	1.070	0.442	18
			Lag 2	0.995	0.931	1.063	0.876	20
			Lag 3	1.042	0.963	1.129	0.307	20
	Renal	Lag 0	Lag 0	-	-	-	-	< 10
			Lag 1	-	-	-	-	< 10
			Lag 2	-	-	-	-	< 10
			Lag 3	-	-	-	-	< 10
		Lag 1	Lag 0	-	-	-	-	< 10
			Lag 1	-	-	-	-	< 10
			Lag 2	-	-	-	-	< 10
			Lag 3	-	-	-	-	< 10
		Lag 2	Lag 0	-	-	-	-	< 10

			Lag 1	-	-	-	-		< 10
			Lag 2	-	-	-	-		< 10
			Lag 3	-	-	-	-		< 10
		Lag 3	Lag 0	-	-	-	-		< 10
			Lag 1	-	-	-	-		< 10
			Lag 2	-	-	-	-		< 10
			Lag 3	-	-	-	-		< 10
	Respiratory	Lag 0	Lag 0	-	-	-	-		< 10
			Lag 1	-	-	-	-		< 10
			Lag 2	-	-	-	-		< 10
			Lag 3	-	-	-	-		< 10
		Lag 1	Lag 0	-	-	-	-		< 10
			Lag 1	-	-	-	-		< 10
			Lag 2	-	-	-	-		< 10
			Lag 3	-	-	-	-		< 10
		Lag 2	Lag 0	-	-	-	-		< 10
			Lag 1	-	-	-	-		< 10
			Lag 2	-	-	-	-		< 10
			Lag 3	-	-	-	-		< 10
		Lag 3	Lag 0		1.097	0.879	1.368	0.413	11
			Lag 1	-	-	-	-		< 10
			Lag 2	-	-	-	-		< 10
			Lag 3	-	-	-	-		< 10
Other, preferred language	All-natural	Lag 0	Lag 0		1.007	1.000	1.014	**0.038**	2629
			Lag 1		1.014	1.007	1.022	**0.000**	2426
			Lag 2		1.009	0.997	1.020	0.135	2401
			Lag 3		1.004	0.995	1.014	0.373	2372
		Lag 1	Lag 0		1.009	1.003	1.015	**0.004**	2491
			Lag 1		1.016	1.009	1.023	**0.000**	2712
			Lag 2		1.010	1.003	1.018	**0.008**	2716
			Lag 3		1.005	0.993	1.016	0.419	2712
		Lag 2	Lag 0		1.011	1.004	1.019	**0.002**	2228
			Lag 1		1.008	1.002	1.014	**0.009**	2645
			Lag 2		1.009	1.002	1.016	**0.009**	2656
			Lag 3		0.999	0.992	1.007	0.836	2662
		Lag 3	Lag 0		1.010	1.002	1.019	**0.016**	2176
			Lag 1		1.002	0.995	1.008	0.663	2311
			Lag 2		1.003	0.997	1.008	0.394	2356
			Lag 3		0.997	0.990	1.004	0.430	2393
	Cardiovascular	Lag 0	Lag 0	-	-	-	-		< 10
			Lag 1	-	-	-	-		< 10
			Lag 2	-	-	-	-		< 10

			Lag 3	-	-	-	-		< 10
		Lag 1	Lag 0	-	-	-	-		< 10
			Lag 1	-	-	-	-		< 10
			Lag 2	-	-	-	-		< 10
			Lag 3	-	-	-	-		< 10
		Lag 2	Lag 0	-	-	-	-		< 10
			Lag 1	-	-	-	-		< 10
			Lag 2	-	-	-	-		< 10
			Lag 3	-	-	-	-		< 10
		Lag 3	Lag 0	-	-	-	-		< 10
			Lag 1	-	-	-	-		< 10
			Lag 2	-	-	-	-		< 10
			Lag 3	-	-	-	-		< 10
	Cerebrovascular	Lag 0	Lag 0		1.047	0.967	1.134	0.261	19
			Lag 1	-	-	-	-		< 10
			Lag 2	-	-	-	-		< 10
			Lag 3		0.986	0.826	1.177	0.875	10
		Lag 1	Lag 0		0.961	0.863	1.072	0.478	17
			Lag 1		0.927	0.824	1.044	0.212	15
			Lag 2		0.941	0.809	1.095	0.430	15
			Lag 3		0.865	0.686	1.092	0.222	16
		Lag 2	Lag 0		0.986	0.887	1.096	0.792	10
			Lag 1		0.943	0.845	1.052	0.292	13
			Lag 2		0.886	0.746	1.052	0.166	12
			Lag 3		0.981	0.868	1.109	0.755	13
		Lag 3	Lag 0		1.032	0.941	1.132	0.499	12
			Lag 1		0.974	0.879	1.078	0.607	10
			Lag 2		1.050	0.987	1.118	0.122	10
			Lag 3		1.060	0.979	1.147	0.149	11
	Renal	Lag 0	Lag 0	-	-	-	-		< 10
			Lag 1	-	-	-	-		< 10
			Lag 2	-	-	-	-		< 10
			Lag 3	-	-	-	-		< 10
		Lag 1	Lag 0	-	-	-	-		< 10
			Lag 1	-	-	-	-		< 10
			Lag 2	-	-	-	-		< 10
			Lag 3	-	-	-	-		< 10
		Lag 2	Lag 0	-	-	-	-		< 10
			Lag 1	-	-	-	-		< 10
			Lag 2	-	-	-	-		< 10
			Lag 3	-	-	-	-		< 10
		Lag 3	Lag 0	-	-	-	-		< 10

			Lag 1	-	-	-	-	< 10
			Lag 2	-	-	-	-	< 10
			Lag 3	-	-	-	-	< 10
	Respiratory	Lag 0	Lag 0	-	-	-	-	< 10
			Lag 1	-	-	-	-	< 10
			Lag 2	-	-	-	-	< 10
			Lag 3	-	-	-	-	< 10
		Lag 1	Lag 0	-	-	-	-	< 10
			Lag 1	-	-	-	-	< 10
			Lag 2	-	-	-	-	< 10
			Lag 3	-	-	-	-	< 10
		Lag 2	Lag 0	-	-	-	-	< 10
			Lag 1	-	-	-	-	< 10
			Lag 2	-	-	-	-	< 10
			Lag 3	-	-	-	-	< 10
		Lag 3	Lag 0	-	-	-	-	< 10
			Lag 1	-	-	-	-	< 10
			Lag 2	-	-	-	-	< 10
			Lag 3	-	-	-	-	< 10
White	All-natural	Lag 0	Lag 0	1.002	0.999	1.004	0.280	29020
			Lag 1	1.000	0.997	1.003	0.894	25632
			Lag 2	0.994	0.990	0.998	**0.004**	25284
			Lag 3	1.002	0.998	1.006	0.433	24938
		Lag 1	Lag 0	1.002	1.000	1.005	0.080	28772
			Lag 1	0.996	0.993	0.999	**0.003**	28242
			Lag 2	0.995	0.992	0.998	**0.002**	28284
			Lag 3	0.999	0.995	1.003	0.569	28256
		Lag 2	Lag 0	1.002	0.999	1.005	0.136	26330
			Lag 1	0.996	0.994	0.999	**0.003**	28421
			Lag 2	0.993	0.990	0.996	**0.000**	28582
			Lag 3	0.996	0.993	0.999	**0.018**	28650
		Lag 3	Lag 0	1.004	1.001	1.007	**0.011**	26605
			Lag 1	1.001	0.998	1.003	0.511	27009
			Lag 2	0.995	0.992	0.997	**0.000**	27290
			Lag 3	0.999	0.997	1.002	0.613	27472
	Cardiovascular	Lag 0	Lag 0	1.004	0.944	1.067	0.909	62
			Lag 1	1.000	0.937	1.067	0.999	70
			Lag 2	1.051	0.965	1.145	0.250	66
			Lag 3	1.005	0.935	1.081	0.892	65
		Lag 1	Lag 0	0.998	0.940	1.061	0.960	61
			Lag 1	0.947	0.879	1.020	0.148	66
			Lag 2	1.015	0.943	1.094	0.686	64

		Lag 3	1.004	0.929	1.086	0.911	63
	Lag 2	Lag 0	1.040	0.975	1.110	0.236	67
		Lag 1	0.985	0.943	1.029	0.502	78
		Lag 2	0.989	0.934	1.049	0.721	75
		Lag 3	0.969	0.913	1.029	0.301	73
	Lag 3	Lag 0	1.083	1.008	1.164	**0.030**	80
		Lag 1	0.994	0.955	1.036	0.788	67
		Lag 2	1.000	0.945	1.059	0.993	63
		Lag 3	1.001	0.953	1.051	0.968	61
Cerebrovascular	Lag 0	Lag 0	1.010	0.966	1.056	0.670	99
		Lag 1	1.029	0.982	1.079	0.228	88
		Lag 2	1.031	0.964	1.103	0.373	91
		Lag 3	0.994	0.919	1.076	0.886	91
	Lag 1	Lag 0	1.026	0.982	1.073	0.253	106
		Lag 1	1.051	1.009	1.094	**0.017**	110
		Lag 2	1.029	0.977	1.083	0.282	116
		Lag 3	0.996	0.923	1.075	0.920	115
	Lag 2	Lag 0	1.007	0.954	1.063	0.803	78
		Lag 1	1.035	0.995	1.077	0.089	96
		Lag 2	1.000	0.957	1.046	0.985	102
		Lag 3	1.000	0.944	1.059	0.997	102
	Lag 3	Lag 0	1.024	0.966	1.085	0.421	78
		Lag 1	1.043	1.000	1.087	**0.048**	89
		Lag 2	0.992	0.949	1.037	0.714	93
		Lag 3	1.001	0.957	1.047	0.976	91
Renal	Lag 0	Lag 0	1.005	0.939	1.075	0.886	32
		Lag 1	0.910	0.803	1.031	0.138	30
		Lag 2	1.048	0.940	1.169	0.400	30
		Lag 3	1.080	0.936	1.246	0.292	30
	Lag 1	Lag 0	1.028	0.969	1.090	0.364	31
		Lag 1	0.966	0.884	1.054	0.434	36
		Lag 2	0.976	0.876	1.088	0.666	40
		Lag 3	1.121	0.993	1.265	0.064	41
	Lag 2	Lag 0	0.998	0.929	1.073	0.963	24
		Lag 1	0.980	0.894	1.074	0.670	31
		Lag 2	1.001	0.919	1.090	0.983	34
		Lag 3	1.114	1.018	1.219	**0.019**	36
	Lag 3	Lag 0	0.877	0.758	1.014	0.076	25
		Lag 1	0.930	0.818	1.058	0.272	28
		Lag 2	0.948	0.860	1.045	0.279	30
		Lag 3	1.020	0.958	1.086	0.533	29
Respiratory	Lag 0	Lag 0	1.001	0.910	1.101	0.985	42

			Lag 1	1.019	0.943	1.101	0.633	38
			Lag 2	1.006	0.912	1.111	0.900	37
			Lag 3	1.169	1.062	1.287	**0.001**	36
		Lag 1	Lag 0	0.942	0.847	1.047	0.268	37
			Lag 1	1.015	0.948	1.086	0.669	40
			Lag 2	1.012	0.938	1.093	0.755	39
			Lag 3	1.135	1.028	1.254	**0.013**	38
		Lag 2	Lag 0	1.033	0.960	1.111	0.389	46
			Lag 1	1.054	0.998	1.113	0.060	47
			Lag 2	1.014	0.943	1.089	0.715	48
			Lag 3	1.045	0.945	1.156	0.390	45
		Lag 3	Lag 0	0.980	0.897	1.070	0.653	48
			Lag 1	1.023	0.976	1.072	0.341	34
			Lag 2	1.002	0.935	1.074	0.951	38
			Lag 3	1.025	0.949	1.108	0.526	35
Black	All-natural	Lag 0	Lag 0	0.997	0.992	1.002	0.246	5850
			Lag 1	0.995	0.989	1.000	0.073	5603
			Lag 2	0.998	0.991	1.005	0.643	5574
			Lag 3	1.000	0.993	1.007	0.978	5550
		Lag 1	Lag 0	1.001	0.996	1.006	0.612	5580
			Lag 1	1.000	0.995	1.005	0.927	5929
			Lag 2	0.998	0.992	1.003	0.384	5969
			Lag 3	0.999	0.992	1.006	0.777	5990
		Lag 2	Lag 0	1.006	1.001	1.012	**0.026**	4870
			Lag 1	1.000	0.995	1.004	0.937	5585
			Lag 2	0.996	0.991	1.001	0.098	5605
			Lag 3	0.995	0.990	1.000	0.053	5623
		Lag 3	Lag 0	1.005	0.999	1.012	0.122	5043
			Lag 1	1.003	0.998	1.008	0.262	4930
			Lag 2	0.996	0.992	1.001	0.110	4976
			Lag 3	0.997	0.992	1.002	0.277	5022
	Cardiovascular	Lag 0	Lag 0	1.041	0.964	1.124	0.311	14
			Lag 1	0.944	0.840	1.060	0.329	14
			Lag 2	1.032	0.900	1.183	0.654	14
			Lag 3	0.756	0.576	0.994	**0.045**	14
		Lag 1	Lag 0	1.042	0.962	1.128	0.317	10
			Lag 1	0.997	0.904	1.099	0.948	14
			Lag 2	1.098	1.004	1.202	**0.042**	14
			Lag 3	1.018	0.889	1.165	0.797	15
		Lag 2	Lag 0	-	-	-	-	< 10
			Lag 1	-	-	-	-	< 10
			Lag 2	-	-	-	-	< 10

			Lag 3	-	-	-	-	< 10
		Lag 3	Lag 0	-	-	-	-	< 10
			Lag 1	-	-	-	-	< 10
			Lag 2	-	-	-	-	< 10
			Lag 3	-	-	-	-	< 10
	Cerebrovascular	Lag 0	Lag 0	1.004	0.903	1.117	0.939	19
			Lag 1	1.014	0.929	1.107	0.748	23
			Lag 2	1.052	0.931	1.189	0.419	23
			Lag 3	1.173	1.036	1.329	**0.012**	23
		Lag 1	Lag 0	0.997	0.895	1.110	0.951	20
			Lag 1	1.029	0.947	1.119	0.498	25
			Lag 2	0.988	0.873	1.117	0.845	25
			Lag 3	1.115	0.990	1.254	0.072	25
		Lag 2	Lag 0	1.040	0.933	1.159	0.479	21
			Lag 1	1.044	0.977	1.115	0.201	25
			Lag 2	0.999	0.919	1.087	0.989	25
			Lag 3	1.049	0.975	1.130	0.200	25
		Lag 3	Lag 0	1.188	1.035	1.365	**0.015**	23
			Lag 1	0.997	0.927	1.072	0.942	22
			Lag 2	1.009	0.945	1.076	0.797	21
			Lag 3	1.023	0.955	1.096	0.514	21
	Renal	Lag 0	Lag 0	-	-	-	-	< 10
			Lag 1	-	-	-	-	< 10
			Lag 2	-	-	-	-	< 10
			Lag 3	-	-	-	-	< 10
		Lag 1	Lag 0	-	-	-	-	< 10
			Lag 1	-	-	-	-	< 10
			Lag 2	-	-	-	-	< 10
			Lag 3	-	-	-	-	< 10
		Lag 2	Lag 0	-	-	-	-	< 10
			Lag 1	-	-	-	-	< 10
			Lag 2	-	-	-	-	< 10
			Lag 3	-	-	-	-	< 10
		Lag 3	Lag 0	-	-	-	-	< 10
			Lag 1	-	-	-	-	< 10
			Lag 2	-	-	-	-	< 10
			Lag 3	-	-	-	-	< 10
	Respiratory	Lag 0	Lag 0	1.088	0.961	1.232	0.183	19
			Lag 1	1.190	1.025	1.381	**0.023**	16
			Lag 2	1.072	0.926	1.241	0.355	17
			Lag 3	1.240	1.059	1.452	**0.007**	17
		Lag 1	Lag 0	1.060	0.934	1.203	0.364	15

			Lag 1	1.183	1.014	1.381	**0.033**	16
			Lag 2	0.985	0.856	1.132	0.827	17
			Lag 3	1.146	0.996	1.319	0.057	17
		Lag 2	Lag 0	1.128	0.976	1.305	0.103	14
			Lag 1	1.068	0.959	1.190	0.231	16
			Lag 2	1.060	0.940	1.196	0.341	17
			Lag 3	1.105	0.965	1.265	0.150	18
		Lag 3	Lag 0	1.085	0.876	1.344	0.456	10
			Lag 1	-	-	-	-	< 10
			Lag 2	-	-	-	-	< 10
			Lag 3	-	-	-	-	< 10
Hispanic	All-natural	Lag 0	Lag 0	1.003	0.999	1.007	0.139	17224
			Lag 1	1.000	0.996	1.004	0.934	16433
			Lag 2	0.998	0.993	1.002	0.362	16322
			Lag 3	1.001	0.996	1.005	0.795	16218
		Lag 1	Lag 0	1.002	0.998	1.005	0.306	16664
			Lag 1	0.998	0.994	1.002	0.248	16697
			Lag 2	0.996	0.992	1.000	**0.042**	16758
			Lag 3	1.001	0.997	1.006	0.541	16781
		Lag 2	Lag 0	1.001	0.997	1.005	0.474	14871
			Lag 1	0.998	0.994	1.001	0.241	16582
			Lag 2	0.993	0.989	0.997	**0.000**	16642
			Lag 3	1.001	0.997	1.005	0.607	16666
		Lag 3	Lag 0	1.003	0.998	1.007	0.220	15821
			Lag 1	1.001	0.997	1.005	0.665	15157
			Lag 2	0.993	0.990	0.997	**0.000**	15308
			Lag 3	0.999	0.995	1.003	0.557	15464
	Cardiovascular	Lag 0	Lag 0	1.020	0.906	1.148	0.749	23
			Lag 1	1.034	0.931	1.148	0.532	18
			Lag 2	0.901	0.783	1.038	0.149	17
			Lag 3	0.957	0.849	1.079	0.473	15
		Lag 1	Lag 0	1.011	0.899	1.138	0.852	27
			Lag 1	1.020	0.927	1.124	0.681	20
			Lag 2	0.939	0.839	1.051	0.271	20
			Lag 3	0.946	0.844	1.061	0.344	19
		Lag 2	Lag 0	1.025	0.921	1.140	0.653	26
			Lag 1	1.041	0.948	1.143	0.402	26
			Lag 2	0.954	0.865	1.052	0.341	26
			Lag 3	1.032	0.940	1.134	0.508	24
		Lag 3	Lag 0	1.050	0.939	1.175	0.391	32
			Lag 1	1.018	0.918	1.127	0.739	25
			Lag 2	0.906	0.804	1.021	0.104	25

		Lag 3	1.041	0.945	1.147	0.417	23
Cerebrovascular	Lag 0	Lag 0	0.940	0.848	1.043	0.245	37
		Lag 1	0.960	0.881	1.046	0.347	40
		Lag 2	1.013	0.913	1.125	0.805	41
		Lag 3	1.033	0.954	1.119	0.421	41
	Lag 1	Lag 0	0.996	0.903	1.098	0.933	43
		Lag 1	0.955	0.880	1.037	0.276	40
		Lag 2	0.977	0.892	1.070	0.610	41
		Lag 3	1.011	0.932	1.096	0.799	42
	Lag 2	Lag 0	1.028	0.931	1.136	0.587	40
		Lag 1	0.978	0.916	1.044	0.495	43
		Lag 2	0.945	0.859	1.040	0.247	43
		Lag 3	1.027	0.953	1.106	0.490	44
	Lag 3	Lag 0	1.025	0.929	1.130	0.628	45
		Lag 1	0.911	0.822	1.010	0.076	33
		Lag 2	0.994	0.933	1.058	0.843	34
		Lag 3	1.036	0.967	1.110	0.311	34
Renal	Lag 0	Lag 0	1.093	0.959	1.246	0.182	20
		Lag 1	0.972	0.845	1.118	0.689	14
		Lag 2	1.018	0.856	1.212	0.837	13
		Lag 3	1.125	0.970	1.304	0.120	14
	Lag 1	Lag 0	0.946	0.790	1.132	0.543	12
		Lag 1	0.990	0.855	1.147	0.895	13
		Lag 2	0.934	0.775	1.126	0.475	13
		Lag 3	1.064	0.924	1.226	0.388	14
	Lag 2	Lag 0	1.028	0.880	1.202	0.725	13
		Lag 1	0.983	0.859	1.125	0.802	17
		Lag 2	0.989	0.853	1.146	0.884	17
		Lag 3	1.062	0.950	1.188	0.290	18
	Lag 3	Lag 0	1.033	0.889	1.200	0.674	17
		Lag 1	1.028	0.883	1.196	0.723	16
		Lag 2	1.112	1.001	1.236	**0.048**	17
		Lag 3	1.066	0.957	1.187	0.244	16
Respiratory	Lag 0	Lag 0	1.008	0.893	1.138	0.897	18
		Lag 1	0.972	0.823	1.148	0.735	12
		Lag 2	1.027	0.877	1.201	0.742	12
		Lag 3	0.955	0.820	1.113	0.557	11
	Lag 1	Lag 0	1.056	0.943	1.183	0.346	17
		Lag 1	0.983	0.845	1.143	0.823	17
		Lag 2	1.005	0.867	1.166	0.942	17
		Lag 3	1.010	0.876	1.166	0.887	16
	Lag 2	Lag 0	1.113	1.002	1.236	**0.046**	24

			Lag 1	0.985	0.898	1.080	0.751	20
			Lag 2	1.011	0.872	1.172	0.884	21
			Lag 3	1.057	0.950	1.177	0.307	19
		Lag 3	Lag 0	1.081	0.945	1.237	0.256	27
			Lag 1	0.896	0.764	1.050	0.176	13
			Lag 2	0.847	0.695	1.032	0.099	14
			Lag 3	1.092	0.973	1.225	0.133	13
Asian	All-natural	Lag 0	Lag 0	1.005	1.000	1.010	**0.041**	5393
			Lag 1	1.007	1.002	1.012	**0.011**	4847
			Lag 2	0.994	0.985	1.002	0.129	4760
			Lag 3	0.999	0.991	1.006	0.761	4698
		Lag 1	Lag 0	1.008	1.004	1.013	**0.000**	5083
			Lag 1	1.009	1.004	1.014	**0.000**	5355
			Lag 2	1.002	0.996	1.008	0.492	5348
			Lag 3	1.004	0.996	1.012	0.301	5344
		Lag 2	Lag 0	1.011	1.006	1.016	**0.000**	4500
			Lag 1	1.006	1.002	1.010	**0.005**	5123
			Lag 2	1.003	0.998	1.008	0.222	5147
			Lag 3	0.999	0.993	1.004	0.679	5187
		Lag 3	Lag 0	1.011	1.005	1.017	**0.000**	4487
			Lag 1	1.004	0.999	1.009	0.113	4626
			Lag 2	1.002	0.998	1.007	0.265	4690
			Lag 3	1.000	0.995	1.005	0.966	4750
	Cardiovascular	Lag 0	Lag 0	-	-	-	-	< 10
			Lag 1	-	-	-	-	< 10
			Lag 2	-	-	-	-	< 10
			Lag 3	-	-	-	-	< 10
		Lag 1	Lag 0	-	-	-	-	< 10
			Lag 1	-	-	-	-	< 10
			Lag 2	-	-	-	-	< 10
			Lag 3	-	-	-	-	< 10
		Lag 2	Lag 0	-	-	-	-	< 10
			Lag 1	-	-	-	-	< 10
			Lag 2	-	-	-	-	< 10
			Lag 3	-	-	-	-	< 10
		Lag 3	Lag 0	-	-	-	-	< 10
			Lag 1	-	-	-	-	< 10
			Lag 2	-	-	-	-	< 10
			Lag 3	-	-	-	-	< 10
	Cerebrovascular	Lag 0	Lag 0	0.973	0.896	1.057	0.518	17
			Lag 1	0.921	0.825	1.029	0.145	19
			Lag 2	0.895	0.765	1.047	0.166	18

			Lag 3	0.936	0.811	1.080	0.364	20
		Lag 1	Lag 0	0.957	0.876	1.045	0.329	19
			Lag 1	0.907	0.821	1.003	0.057	26
			Lag 2	0.977	0.889	1.073	0.620	26
			Lag 3	0.920	0.798	1.061	0.251	28
		Lag 2	Lag 0	0.956	0.864	1.057	0.378	15
			Lag 1	0.966	0.915	1.020	0.218	29
			Lag 2	1.014	0.949	1.082	0.688	27
			Lag 3	0.993	0.920	1.071	0.856	28
		Lag 3	Lag 0	0.986	0.892	1.090	0.788	20
			Lag 1	0.997	0.942	1.055	0.923	24
			Lag 2	1.058	1.003	1.115	**0.037**	24
			Lag 3	1.025	0.962	1.093	0.443	24
	Renal	Lag 0	Lag 0	-	-	-	-	< 10
			Lag 1	-	-	-	-	< 10
			Lag 2	-	-	-	-	< 10
			Lag 3	-	-	-	-	< 10
		Lag 1	Lag 0	1.024	0.763	1.374	0.875	10
			Lag 1	-	-	-	-	< 10
			Lag 2	-	-	-	-	< 10
			Lag 3	-	-	-	-	< 10
		Lag 2	Lag 0	-	-	-	-	< 10
			Lag 1	-	-	-	-	< 10
			Lag 2	-	-	-	-	< 10
			Lag 3	-	-	-	-	< 10
		Lag 3	Lag 0	-	-	-	-	< 10
			Lag 1	-	-	-	-	< 10
			Lag 2	-	-	-	-	< 10
			Lag 3	-	-	-	-	< 10
	Respiratory	Lag 0	Lag 0	-	-	-	-	< 10
			Lag 1	-	-	-	-	< 10
			Lag 2	-	-	-	-	< 10
			Lag 3	-	-	-	-	< 10
		Lag 1	Lag 0	-	-	-	-	< 10
			Lag 1	-	-	-	-	< 10
			Lag 2	-	-	-	-	< 10
			Lag 3	-	-	-	-	< 10
		Lag 2	Lag 0	-	-	-	-	< 10
			Lag 1	-	-	-	-	< 10
			Lag 2	-	-	-	-	< 10
			Lag 3	-	-	-	-	< 10
		Lag 3	Lag 0	-	-	-	-	< 10

			Lag 1	-	-	-	-		< 10
			Lag 2	-	-	-	-		< 10
			Lag 3	-	-	-	-		< 10
Other Grouped Races	All-natural	Lag 0	Lag 0		1.004	0.995	1.014	0.363	2542
			Lag 1		1.005	0.994	1.016	0.371	2421
			Lag 2		1.009	0.996	1.023	0.169	2395
			Lag 3		1.005	0.993	1.017	0.418	2374
		Lag 1	Lag 0		1.007	0.998	1.016	0.140	2522
			Lag 1		0.996	0.987	1.006	0.458	2602
			Lag 2		1.007	0.997	1.018	0.162	2605
			Lag 3		1.002	0.989	1.015	0.778	2598
		Lag 2	Lag 0		1.005	0.994	1.015	0.387	2251
			Lag 1		0.991	0.982	1.001	0.065	2619
			Lag 2		1.002	0.992	1.011	0.705	2642
			Lag 3		0.997	0.986	1.008	0.548	2654
		Lag 3	Lag 0		0.997	0.985	1.010	0.666	2271
			Lag 1		1.002	0.992	1.012	0.689	2392
			Lag 2		1.001	0.993	1.010	0.742	2426
			Lag 3		0.991	0.981	1.001	0.076	2461
	Cardiovascular	Lag 0	Lag 0	-	-	-	-		< 10
			Lag 1	-	-	-	-		< 10
			Lag 2	-	-	-	-		< 10
			Lag 3	-	-	-	-		< 10
		Lag 1	Lag 0	-	-	-	-		< 10
			Lag 1	-	-	-	-		< 10
			Lag 2	-	-	-	-		< 10
			Lag 3	-	-	-	-		< 10
		Lag 2	Lag 0	-	-	-	-		< 10
			Lag 1	-	-	-	-		< 10
			Lag 2	-	-	-	-		< 10
			Lag 3	-	-	-	-		< 10
		Lag 3	Lag 0	-	-	-	-		< 10
			Lag 1	-	-	-	-		< 10
			Lag 2	-	-	-	-		< 10
			Lag 3	-	-	-	-		< 10
	Cerebrovascular	Lag 0	Lag 0		1.217	1.019	1.453	**0.031**	11
			Lag 1	-	-	-	-		< 10
			Lag 2	-	-	-	-		< 10
			Lag 3	-	-	-	-		< 10
		Lag 1	Lag 0	-	-	-	-		< 10
			Lag 1	-	-	-	-		< 10
			Lag 2	-	-	-	-		< 10

			Lag 3	-	-	-	-	< 10
		Lag 2	Lag 0	-	-	-	-	< 10
			Lag 1	1.047	0.909	1.207	0.521	11
			Lag 2	1.026	0.776	1.356	0.859	10
			Lag 3	1.123	0.902	1.398	0.298	11
		Lag 3	Lag 0	-	-	-	-	< 10
			Lag 1	1.037	0.839	1.281	0.737	10
			Lag 2	1.070	0.820	1.395	0.619	10
			Lag 3	1.180	0.922	1.509	0.188	10
	Renal	Lag 0	Lag 0	-	-	-	-	< 10
			Lag 1	-	-	-	-	< 10
			Lag 2	-	-	-	-	< 10
			Lag 3	-	-	-	-	< 10
		Lag 1	Lag 0	-	-	-	-	< 10
			Lag 1	-	-	-	-	< 10
			Lag 2	-	-	-	-	< 10
			Lag 3	-	-	-	-	< 10
		Lag 2	Lag 0	-	-	-	-	< 10
			Lag 1	-	-	-	-	< 10
			Lag 2	-	-	-	-	< 10
			Lag 3	-	-	-	-	< 10
		Lag 3	Lag 0	-	-	-	-	< 10
			Lag 1	-	-	-	-	< 10
			Lag 2	-	-	-	-	< 10
			Lag 3	-	-	-	-	< 10
	Respiratory	Lag 0	Lag 0	-	-	-	-	< 10
			Lag 1	-	-	-	-	< 10
			Lag 2	-	-	-	-	< 10
			Lag 3	-	-	-	-	< 10
		Lag 1	Lag 0	-	-	-	-	< 10
			Lag 1	-	-	-	-	< 10
			Lag 2	-	-	-	-	< 10
			Lag 3	-	-	-	-	< 10
		Lag 2	Lag 0	-	-	-	-	< 10
			Lag 1	-	-	-	-	< 10
			Lag 2	-	-	-	-	< 10
			Lag 3	-	-	-	-	< 10
		Lag 3	Lag 0	-	-	-	-	< 10
			Lag 1	-	-	-	-	< 10
			Lag 2	-	-	-	-	< 10
			Lag 3	-	-	-	-	< 10
Higher	All-natural	Lag 0	Lag 0	1.002	1.000	1.004	**0.040**	39608

Education (< 50% high school education or less)			Lag 1	1.001	0.999	1.004	0.309	35830
			Lag 2	0.996	0.992	0.999	**0.009**	35466
			Lag 3	1.003	1.000	1.006	0.069	35132
		Lag 1	Lag 0	1.003	1.001	1.005	**0.005**	38335
			Lag 1	0.999	0.997	1.001	0.268	38928
			Lag 2	0.997	0.995	1.000	**0.024**	39031
			Lag 3	1.001	0.998	1.005	0.384	39035
		Lag 2	Lag 0	1.004	1.001	1.006	**0.002**	34605
			Lag 1	0.999	0.997	1.001	0.272	38473
			Lag 2	0.996	0.994	0.998	**0.000**	38704
			Lag 3	0.998	0.996	1.000	0.088	38828
		Lag 3	Lag 0	1.005	1.002	1.007	**0.000**	35172
			Lag 1	1.001	0.999	1.003	0.402	35858
			Lag 2	0.997	0.995	0.999	**0.001**	36299
			Lag 3	0.999	0.996	1.001	0.187	36669
	Cardiovascular	Lag 0	Lag 0	1.014	0.971	1.060	0.525	74
			Lag 1	1.008	0.963	1.055	0.729	79
			Lag 2	1.006	0.934	1.084	0.868	75
			Lag 3	0.976	0.909	1.049	0.511	74
		Lag 1	Lag 0	1.024	0.980	1.071	0.290	67
			Lag 1	1.002	0.960	1.045	0.926	76
			Lag 2	1.034	0.981	1.090	0.207	74
			Lag 3	0.986	0.919	1.058	0.693	76
		Lag 2	Lag 0	1.038	0.988	1.091	0.139	76
			Lag 1	0.990	0.955	1.026	0.572	83
			Lag 2	1.002	0.958	1.049	0.915	81
			Lag 3	0.990	0.945	1.037	0.670	79
		Lag 3	Lag 0	1.045	0.986	1.107	0.137	92
			Lag 1	1.000	0.963	1.038	0.981	78
			Lag 2	1.001	0.961	1.043	0.963	75
			Lag 3	1.006	0.965	1.048	0.786	73
	Cerebrovascular	Lag 0	Lag 0	1.009	0.973	1.046	0.627	136
			Lag 1	0.993	0.955	1.032	0.704	120
			Lag 2	1.024	0.967	1.085	0.421	121
			Lag 3	1.043	0.989	1.100	0.119	122
		Lag 1	Lag 0	1.018	0.982	1.055	0.337	143
			Lag 1	1.006	0.975	1.038	0.696	152
			Lag 2	0.999	0.956	1.044	0.971	155
			Lag 3	1.004	0.948	1.063	0.883	156
		Lag 2	Lag 0	1.027	0.985	1.070	0.219	124
			Lag 1	1.009	0.982	1.037	0.513	139
			Lag 2	0.995	0.960	1.031	0.779	140

			Lag 3	1.010	0.971	1.050	0.633	144
		Lag 3	Lag 0	1.031	0.984	1.081	0.200	124
			Lag 1	1.006	0.974	1.039	0.736	115
			Lag 2	1.006	0.979	1.035	0.658	116
			Lag 3	1.021	0.989	1.054	0.201	115
	Renal	Lag 0	Lag 0	1.036	0.975	1.100	0.251	49
			Lag 1	1.024	0.933	1.123	0.619	44
			Lag 2	1.030	0.929	1.143	0.572	43
			Lag 3	1.060	0.958	1.173	0.256	44
		Lag 1	Lag 0	1.037	0.982	1.096	0.194	46
			Lag 1	1.036	0.957	1.122	0.383	49
			Lag 2	0.950	0.859	1.051	0.322	50
			Lag 3	1.052	0.960	1.152	0.278	52
		Lag 2	Lag 0	1.006	0.938	1.078	0.874	31
			Lag 1	1.032	0.946	1.126	0.474	38
			Lag 2	0.982	0.905	1.064	0.655	40
			Lag 3	1.021	0.953	1.095	0.550	42
		Lag 3	Lag 0	0.956	0.871	1.049	0.338	34
			Lag 1	0.982	0.882	1.094	0.741	31
			Lag 2	0.984	0.918	1.054	0.637	34
			Lag 3	0.999	0.942	1.060	0.980	35
	Respiratory	Lag 0	Lag 0	1.038	0.969	1.111	0.287	60
			Lag 1	1.046	0.977	1.120	0.193	50
			Lag 2	0.987	0.896	1.087	0.791	50
			Lag 3	1.136	1.044	1.236	**0.003**	49
		Lag 1	Lag 0	1.009	0.937	1.086	0.812	54
			Lag 1	1.044	0.981	1.111	0.173	49
			Lag 2	1.000	0.935	1.070	0.994	49
			Lag 3	1.116	1.023	1.217	**0.014**	48
		Lag 2	Lag 0	1.051	0.982	1.125	0.154	58
			Lag 1	1.065	1.009	1.123	**0.022**	58
			Lag 2	0.994	0.930	1.063	0.860	61
			Lag 3	1.083	1.004	1.168	**0.039**	59
		Lag 3	Lag 0	1.023	0.945	1.108	0.571	67
			Lag 1	1.026	0.955	1.103	0.480	43
			Lag 2	0.996	0.932	1.065	0.913	49
			Lag 3	1.045	0.979	1.114	0.187	49
Lower Education (> 50% high school education or less)	All-natural	Lag 0	Lag 0	1.000	0.997	1.004	0.808	20355
			Lag 1	0.998	0.994	1.002	0.308	19057
			Lag 2	0.998	0.993	1.002	0.275	18846
			Lag 3	0.997	0.993	1.001	0.184	18633
		Lag 1	Lag 0	1.003	1.000	1.007	**0.039**	20223

or less)			Lag 1	0.998	0.995	1.002	0.408	19850
			Lag 2	0.997	0.993	1.000	0.070	19900
			Lag 3	0.999	0.995	1.003	0.618	19914
		Lag 2	Lag 0	1.003	1.000	1.006	0.059	18139
			Lag 1	0.997	0.994	1.000	0.080	19791
			Lag 2	0.993	0.990	0.997	**0.000**	19872
			Lag 3	0.998	0.994	1.002	0.283	19926
		Lag 3	Lag 0	1.004	1.000	1.008	**0.037**	18980
			Lag 1	1.003	1.000	1.006	0.084	18189
			Lag 2	0.994	0.990	0.997	**0.000**	18337
			Lag 3	1.000	0.996	1.003	0.888	18468
	Cardiovascular	Lag 0	Lag 0	1.015	0.932	1.105	0.736	37
			Lag 1	1.035	0.951	1.126	0.426	36
			Lag 2	1.017	0.920	1.124	0.742	35
			Lag 3	0.946	0.853	1.050	0.299	33
		Lag 1	Lag 0	1.018	0.944	1.098	0.638	44
			Lag 1	1.030	0.950	1.116	0.474	39
			Lag 2	1.016	0.938	1.100	0.694	38
			Lag 3	1.003	0.919	1.095	0.943	35
		Lag 2	Lag 0	1.085	1.004	1.174	**0.040**	37
			Lag 1	0.977	0.895	1.066	0.594	42
			Lag 2	0.964	0.880	1.057	0.441	40
			Lag 3	0.970	0.884	1.064	0.516	38
		Lag 3	Lag 0	1.098	0.999	1.207	0.053	40
			Lag 1	0.991	0.927	1.059	0.793	34
			Lag 2	0.883	0.787	0.990	**0.033**	32
			Lag 3	0.970	0.878	1.073	0.558	31
	Cerebrovascular	Lag 0	Lag 0	0.956	0.880	1.039	0.288	47
			Lag 1	1.019	0.953	1.090	0.578	59
			Lag 2	0.997	0.920	1.081	0.944	60
			Lag 3	0.991	0.905	1.084	0.840	62
		Lag 1	Lag 0	0.981	0.910	1.057	0.615	51
			Lag 1	1.004	0.932	1.082	0.912	57
			Lag 2	1.010	0.938	1.087	0.790	60
			Lag 3	1.018	0.945	1.096	0.644	62
		Lag 2	Lag 0	0.950	0.868	1.040	0.265	36
			Lag 1	1.011	0.949	1.076	0.741	65
			Lag 2	0.986	0.921	1.055	0.686	67
			Lag 3	1.034	0.969	1.105	0.314	66
		Lag 3	Lag 0	1.023	0.945	1.107	0.570	49
			Lag 1	1.030	0.977	1.086	0.276	63
			Lag 2	1.041	0.974	1.113	0.235	65

			Lag 3	1.019	0.952	1.091	0.583	65
	Renal	Lag 0	Lag 0	1.011	0.909	1.125	0.837	22
			Lag 1	0.935	0.833	1.051	0.260	20
			Lag 2	1.047	0.939	1.168	0.407	19
			Lag 3	1.061	0.879	1.280	0.538	19
		Lag 1	Lag 0	1.029	0.922	1.149	0.607	20
			Lag 1	0.898	0.785	1.026	0.113	18
			Lag 2	1.007	0.886	1.146	0.911	20
			Lag 3	1.110	0.956	1.289	0.169	20
		Lag 2	Lag 0	0.975	0.853	1.113	0.705	19
			Lag 1	0.928	0.814	1.057	0.260	20
			Lag 2	1.059	0.941	1.192	0.340	21
			Lag 3	1.155	1.026	1.301	**0.018**	23
		Lag 3	Lag 0	0.941	0.795	1.113	0.476	22
			Lag 1	0.961	0.826	1.118	0.609	26
			Lag 2	1.064	0.955	1.186	0.260	27
			Lag 3	1.074	0.970	1.190	0.168	25
	Respiratory	Lag 0	Lag 0	0.934	0.816	1.069	0.322	25
			Lag 1	1.007	0.899	1.128	0.908	25
			Lag 2	1.021	0.924	1.127	0.689	26
			Lag 3	1.031	0.938	1.134	0.524	25
		Lag 1	Lag 0	0.965	0.855	1.090	0.566	24
			Lag 1	1.027	0.933	1.131	0.583	31
			Lag 2	0.973	0.879	1.077	0.595	32
			Lag 3	1.050	0.949	1.160	0.345	31
		Lag 2	Lag 0	1.070	0.986	1.162	0.105	38
			Lag 1	1.002	0.938	1.071	0.949	32
			Lag 2	0.999	0.912	1.094	0.983	34
			Lag 3	0.981	0.882	1.090	0.720	32
		Lag 3	Lag 0	0.969	0.867	1.082	0.574	32
			Lag 1	1.016	0.977	1.056	0.434	21
			Lag 2	0.949	0.853	1.056	0.340	23
			Lag 3	1.004	0.912	1.106	0.930	21
Lesser poverty (<25% households living under poverty)	All-natural	Lag 0	Lag 0	1.002	1.000	1.004	0.078	47779
			Lag 1	1.001	0.999	1.003	0.411	42041
			Lag 2	0.995	0.992	0.998	**0.002**	41560
			Lag 3	1.002	1.000	1.005	0.101	41129
		Lag 1	Lag 0	1.003	1.001	1.005	**0.004**	46248
			Lag 1	0.999	0.997	1.001	0.326	46782
			Lag 2	0.997	0.995	1.000	**0.027**	46844
			Lag 3	1.001	0.998	1.004	0.616	46798
		Lag 2	Lag 0	1.004	1.002	1.006	**0.001**	41676

			Lag 1	0.998	0.997	1.000	0.101	46270
			Lag 2	0.995	0.993	0.998	**0.000**	46530
			Lag 3	0.997	0.995	0.999	**0.011**	46651
		Lag 3	Lag 0	1.005	1.002	1.007	**0.000**	42465
			Lag 1	1.002	1.000	1.004	0.091	42950
			Lag 2	0.996	0.994	0.998	**0.000**	43445
			Lag 3	0.999	0.997	1.001	0.159	43860
	Cardiovascular	Lag 0	Lag 0	1.001	0.957	1.046	0.978	81
			Lag 1	1.015	0.973	1.058	0.484	91
			Lag 2	0.999	0.933	1.069	0.969	86
			Lag 3	0.968	0.907	1.034	0.339	84
		Lag 1	Lag 0	1.018	0.974	1.064	0.426	84
			Lag 1	1.005	0.965	1.046	0.820	90
			Lag 2	1.025	0.976	1.077	0.319	89
			Lag 3	0.978	0.911	1.048	0.525	90
		Lag 2	Lag 0	1.050	1.001	1.101	**0.044**	86
			Lag 1	0.984	0.947	1.022	0.392	102
			Lag 2	0.986	0.940	1.034	0.551	99
			Lag 3	0.971	0.921	1.024	0.281	95
		Lag 3	Lag 0	1.061	1.003	1.121	**0.038**	104
			Lag 1	0.999	0.963	1.036	0.951	91
			Lag 2	0.981	0.938	1.026	0.402	85
			Lag 3	0.990	0.947	1.035	0.664	82
	Cerebrovascular	Lag 0	Lag 0	1.010	0.974	1.047	0.583	154
			Lag 1	1.007	0.973	1.042	0.700	149
			Lag 2	1.019	0.965	1.076	0.498	150
			Lag 3	1.035	0.984	1.090	0.180	153
		Lag 1	Lag 0	1.023	0.988	1.060	0.199	166
			Lag 1	1.008	0.978	1.040	0.602	176
			Lag 2	1.004	0.963	1.046	0.864	180
			Lag 3	0.993	0.939	1.049	0.793	183
		Lag 2	Lag 0	1.015	0.974	1.058	0.475	136
			Lag 1	1.009	0.982	1.036	0.524	166
			Lag 2	0.997	0.963	1.032	0.863	169
			Lag 3	1.020	0.984	1.058	0.279	175
		Lag 3	Lag 0	1.035	0.990	1.082	0.131	148
			Lag 1	1.008	0.977	1.041	0.603	147
			Lag 2	1.015	0.988	1.042	0.276	149
			Lag 3	1.028	0.998	1.060	0.071	151
	Renal	Lag 0	Lag 0	1.007	0.944	1.076	0.824	54
			Lag 1	0.982	0.898	1.075	0.699	45
			Lag 2	1.001	0.906	1.106	0.980	43

			Lag 3	1.054	0.950	1.170	0.318	43
		Lag 1	Lag 0	1.031	0.971	1.096	0.319	50
			Lag 1	0.996	0.924	1.073	0.916	51
			Lag 2	0.917	0.820	1.025	0.127	52
			Lag 3	1.066	0.974	1.168	0.165	53
		Lag 2	Lag 0	1.001	0.928	1.080	0.970	41
			Lag 1	1.016	0.942	1.097	0.676	47
			Lag 2	0.988	0.911	1.071	0.769	48
			Lag 3	1.033	0.966	1.105	0.343	50
		Lag 3	Lag 0	0.982	0.901	1.070	0.683	49
			Lag 1	0.984	0.896	1.082	0.746	45
			Lag 2	1.017	0.958	1.080	0.582	49
			Lag 3	1.001	0.946	1.060	0.963	48
	Respiratory	Lag 0	Lag 0	1.024	0.952	1.101	0.529	65
			Lag 1	1.033	0.969	1.102	0.314	59
			Lag 2	0.997	0.921	1.079	0.934	58
			Lag 3	1.105	1.023	1.194	**0.011**	57
		Lag 1	Lag 0	1.026	0.954	1.104	0.487	63
			Lag 1	1.024	0.964	1.088	0.443	62
			Lag 2	0.997	0.935	1.063	0.929	60
			Lag 3	1.077	0.996	1.166	0.064	59
		Lag 2	Lag 0	1.072	1.007	1.142	**0.030**	75
			Lag 1	1.051	1.001	1.103	**0.044**	72
			Lag 2	0.998	0.939	1.060	0.937	74
			Lag 3	1.050	0.975	1.130	0.201	71
		Lag 3	Lag 0	1.014	0.940	1.093	0.722	79
			Lag 1	1.019	0.982	1.058	0.310	50
			Lag 2	0.991	0.929	1.059	0.798	56
			Lag 3	1.031	0.969	1.096	0.338	55
Higher poverty (>25% households living under poverty)	All-natural	Lag 0	Lag 0	1.001	0.997	1.006	0.487	12242
			Lag 1	0.998	0.994	1.002	0.388	12892
			Lag 2	0.999	0.994	1.004	0.757	12774
			Lag 3	0.997	0.993	1.002	0.201	12649
		Lag 1	Lag 0	1.004	1.000	1.008	**0.045**	12365
			Lag 1	0.998	0.994	1.002	0.280	12039
			Lag 2	0.996	0.992	1.000	0.053	12118
			Lag 3	1.000	0.995	1.005	0.951	12171
		Lag 2	Lag 0	1.003	0.999	1.007	0.102	11139
			Lag 1	0.998	0.995	1.002	0.385	12052
			Lag 2	0.993	0.989	0.997	**0.001**	12086
			Lag 3	1.001	0.997	1.005	0.723	12126
		Lag 3	Lag 0	1.005	1.000	1.009	**0.045**	11756

		Lag 1	1.001	0.997	1.005	0.684	11158
		Lag 2	0.995	0.991	0.998	**0.004**	11241
		Lag 3	1.000	0.996	1.004	0.889	11306
Cardiovascular	Lag 0	Lag 0	1.078	0.985	1.178	0.101	30
		Lag 1	1.006	0.894	1.132	0.922	24
		Lag 2	1.052	0.932	1.189	0.411	24
		Lag 3	0.957	0.840	1.091	0.515	23
	Lag 1	Lag 0	1.047	0.966	1.134	0.263	27
		Lag 1	1.025	0.930	1.130	0.620	25
		Lag 2	1.046	0.951	1.151	0.355	23
		Lag 3	1.020	0.934	1.115	0.659	21
	Lag 2	Lag 0	1.061	0.973	1.158	0.181	27
		Lag 1	1.003	0.933	1.078	0.938	24
		Lag 2	1.024	0.944	1.111	0.566	23
		Lag 3	1.018	0.946	1.096	0.631	23
	Lag 3	Lag 0	1.052	0.942	1.175	0.369	28
		Lag 1	0.986	0.918	1.059	0.695	21
		Lag 2	0.978	0.897	1.065	0.605	22
		Lag 3	1.033	0.951	1.122	0.443	22
Cerebrovascular	Lag 0	Lag 0	0.945	0.863	1.036	0.230	29
		Lag 1	0.948	0.865	1.040	0.261	30
		Lag 2	1.002	0.913	1.099	0.971	31
		Lag 3	0.997	0.900	1.104	0.950	31
	Lag 1	Lag 0	0.942	0.856	1.038	0.227	28
		Lag 1	0.994	0.918	1.077	0.889	33
		Lag 2	0.994	0.912	1.083	0.889	35
		Lag 3	1.043	0.962	1.131	0.304	35
	Lag 2	Lag 0	0.987	0.902	1.081	0.783	24
		Lag 1	1.019	0.954	1.088	0.573	38
		Lag 2	0.978	0.905	1.057	0.580	38
		Lag 3	0.996	0.917	1.081	0.916	35
	Lag 3	Lag 0	1.005	0.910	1.110	0.922	25
		Lag 1	1.027	0.970	1.089	0.361	31
		Lag 2	0.975	0.899	1.058	0.546	33
		Lag 3	0.961	0.878	1.051	0.386	29
Renal	Lag 0	Lag 0	1.055	0.966	1.152	0.237	17
		Lag 1	0.972	0.866	1.091	0.628	19
		Lag 2	1.102	0.973	1.248	0.125	19
		Lag 3	1.079	0.908	1.283	0.386	20
	Lag 1	Lag 0	1.039	0.955	1.130	0.377	16
		Lag 1	0.942	0.819	1.085	0.410	16
		Lag 2	1.051	0.932	1.185	0.419	18

			Lag 3	1.065	0.916	1.238	0.413	19
		Lag 2	Lag 0	-	-	-	-	< 10
			Lag 1	0.878	0.708	1.090	0.238	11
			Lag 2	1.037	0.926	1.161	0.529	13
			Lag 3	1.123	0.993	1.270	0.065	15
		Lag 3	Lag 0	-	-	-	-	< 10
			Lag 1	0.905	0.705	1.162	0.434	12
			Lag 2	0.937	0.808	1.086	0.385	12
			Lag 3	1.087	0.965	1.226	0.170	12
	Respiratory	Lag 0	Lag 0	0.976	0.867	1.099	0.692	20
			Lag 1	1.063	0.920	1.227	0.409	17
			Lag 2	1.013	0.879	1.168	0.854	18
			Lag 3	1.056	0.950	1.174	0.315	17
		Lag 1	Lag 0	0.913	0.794	1.051	0.205	15
			Lag 1	1.111	0.995	1.241	0.061	19
			Lag 2	0.966	0.855	1.092	0.585	21
			Lag 3	1.108	0.985	1.247	0.089	20
		Lag 2	Lag 0	1.028	0.935	1.131	0.563	21
			Lag 1	1.032	0.956	1.113	0.420	19
			Lag 2	0.988	0.879	1.111	0.843	21
			Lag 3	1.039	0.938	1.150	0.464	20
		Lag 3	Lag 0	0.974	0.860	1.104	0.684	20
			Lag 1	0.997	0.906	1.097	0.949	15
			Lag 2	0.951	0.846	1.071	0.409	16
			Lag 3	1.032	0.926	1.151	0.566	15
Majority Rural (>50%)	All-natural	Lag 0	Lag 0	1.002	0.988	1.017	0.744	1672
			Lag 1	0.962	0.939	0.985	**0.001**	1332
			Lag 2	0.972	0.950	0.995	**0.018**	1171
			Lag 3	0.984	0.954	1.014	0.294	1065
		Lag 1	Lag 0	1.005	0.992	1.019	0.448	1747
			Lag 1	0.975	0.960	0.991	**0.002**	1522
			Lag 2	0.973	0.952	0.995	**0.015**	1436
			Lag 3	0.952	0.926	0.979	**0.001**	1322
		Lag 2	Lag 0	1.001	0.991	1.011	0.890	1659
			Lag 1	0.980	0.966	0.994	**0.005**	1709
			Lag 2	0.978	0.961	0.994	**0.008**	1635
			Lag 3	0.959	0.935	0.983	**0.001**	1546
		Lag 3	Lag 0	1.006	0.996	1.016	0.257	1756
			Lag 1	1.002	0.993	1.012	0.643	1701
			Lag 2	0.993	0.980	1.007	0.355	1690
			Lag 3	0.989	0.974	1.005	0.189	1627
	Cardiovascular	Lag 0	Lag 0	-	-	-	-	< 10

			Lag 1	-	-	-	-	< 10
			Lag 2	-	-	-	-	< 10
			Lag 3	-	-	-	-	< 10
		Lag 1	Lag 0	-	-	-	-	< 10
			Lag 1	-	-	-	-	< 10
			Lag 2	-	-	-	-	< 10
			Lag 3	-	-	-	-	< 10
		Lag 2	Lag 0	-	-	-	-	< 10
			Lag 1	-	-	-	-	< 10
			Lag 2	-	-	-	-	< 10
			Lag 3	-	-	-	-	< 10
		Lag 3	Lag 0	-	-	-	-	< 10
			Lag 1	-	-	-	-	< 10
			Lag 2	-	-	-	-	< 10
			Lag 3	-	-	-	-	< 10
	Cerebrovascular	Lag 0	Lag 0	-	-	-	-	< 10
			Lag 1	-	-	-	-	< 10
			Lag 2	-	-	-	-	< 10
			Lag 3	-	-	-	-	< 10
		Lag 1	Lag 0	-	-	-	-	< 10
			Lag 1	-	-	-	-	< 10
			Lag 2	-	-	-	-	< 10
			Lag 3	-	-	-	-	< 10
		Lag 2	Lag 0	-	-	-	-	< 10
			Lag 1	-	-	-	-	< 10
			Lag 2	-	-	-	-	< 10
			Lag 3	-	-	-	-	< 10
		Lag 3	Lag 0	-	-	-	-	< 10
			Lag 1	-	-	-	-	< 10
			Lag 2	-	-	-	-	< 10
			Lag 3	-	-	-	-	< 10
	Renal	Lag 0	Lag 0	-	-	-	-	< 10
			Lag 1	-	-	-	-	< 10
			Lag 2	-	-	-	-	< 10
			Lag 3	-	-	-	-	< 10
		Lag 1	Lag 0	-	-	-	-	< 10
			Lag 1	-	-	-	-	< 10
			Lag 2	-	-	-	-	< 10
			Lag 3	-	-	-	-	< 10
		Lag 2	Lag 0	-	-	-	-	< 10
			Lag 1	-	-	-	-	< 10
			Lag 2	-	-	-	-	< 10

			Lag 3	-	-	-	-	< 10
		Lag 3	Lag 0	-	-	-	-	< 10
			Lag 1	-	-	-	-	< 10
			Lag 2	-	-	-	-	< 10
			Lag 3	-	-	-	-	< 10
	Respiratory	Lag 0	Lag 0	-	-	-	-	< 10
			Lag 1	-	-	-	-	< 10
			Lag 2	-	-	-	-	< 10
			Lag 3	-	-	-	-	< 10
		Lag 1	Lag 0	-	-	-	-	< 10
			Lag 1	-	-	-	-	< 10
			Lag 2	-	-	-	-	< 10
			Lag 3	-	-	-	-	< 10
		Lag 2	Lag 0	-	-	-	-	< 10
			Lag 1	-	-	-	-	< 10
			Lag 2	-	-	-	-	< 10
			Lag 3	-	-	-	-	< 10
		Lag 3	Lag 0	-	-	-	-	< 10
			Lag 1	-	-	-	-	< 10
			Lag 2	-	-	-	-	< 10
			Lag 3	-	-	-	-	< 10
Majority Urban	All-natural	Lag 0	Lag 0	1.002	1.000	1.004	0.067	58356
			Lag 1	1.001	0.999	1.003	0.541	53603
			Lag 2	0.997	0.994	0.999	**0.013**	53164
			Lag 3	1.001	0.999	1.003	0.416	52713
		Lag 1	Lag 0	1.003	1.001	1.005	**0.001**	56873
			Lag 1	0.999	0.997	1.001	0.308	57302
			Lag 2	0.997	0.995	0.999	**0.008**	57528
			Lag 3	1.001	0.998	1.003	0.451	57647
		Lag 2	Lag 0	1.004	1.002	1.006	**0.000**	51162
			Lag 1	0.999	0.997	1.000	0.125	56619
			Lag 2	0.995	0.993	0.997	**0.000**	56983
			Lag 3	0.998	0.996	1.000	0.078	57233
		Lag 3	Lag 0	1.005	1.002	1.007	**0.000**	52470
			Lag 1	1.002	1.000	1.003	0.103	52411
			Lag 2	0.996	0.994	0.998	**0.000**	52999
			Lag 3	0.999	0.997	1.001	0.293	53541
	Cardiovascular	Lag 0	Lag 0	1.016	0.977	1.056	0.435	109
			Lag 1	1.017	0.978	1.058	0.397	115
			Lag 2	1.016	0.957	1.078	0.601	110
			Lag 3	0.970	0.915	1.029	0.316	107
		Lag 1	Lag 0	1.024	0.985	1.064	0.224	109

			Lag 1	1.011	0.974	1.049	0.567	113
			Lag 2	1.030	0.986	1.076	0.189	111
			Lag 3	0.995	0.941	1.051	0.844	110
		Lag 2	Lag 0	1.053	1.010	1.098	**0.016**	111
			Lag 1	0.988	0.956	1.021	0.477	121
			Lag 2	0.992	0.952	1.034	0.709	117
			Lag 3	0.986	0.946	1.029	0.522	113
		Lag 3	Lag 0	1.061	1.009	1.116	**0.020**	128
			Lag 1	0.993	0.959	1.029	0.709	107
			Lag 2	0.982	0.944	1.022	0.370	105
			Lag 3	0.994	0.955	1.034	0.759	102
	Cerebrovascular	Lag 0	Lag 0	0.998	0.965	1.032	0.921	176
			Lag 1	0.998	0.965	1.031	0.891	174
			Lag 2	1.017	0.970	1.066	0.488	176
			Lag 3	1.027	0.981	1.076	0.249	180
		Lag 1	Lag 0	1.010	0.977	1.044	0.558	187
			Lag 1	1.005	0.976	1.035	0.742	202
			Lag 2	1.005	0.968	1.044	0.783	209
			Lag 3	1.012	0.967	1.059	0.603	214
		Lag 2	Lag 0	1.011	0.974	1.049	0.573	154
			Lag 1	1.011	0.986	1.036	0.405	199
			Lag 2	0.994	0.963	1.026	0.706	199
			Lag 3	1.018	0.984	1.052	0.306	204
		Lag 3	Lag 0	1.029	0.988	1.072	0.174	166
			Lag 1	1.010	0.983	1.039	0.460	173
			Lag 2	1.009	0.984	1.035	0.475	174
			Lag 3	1.021	0.992	1.050	0.164	175
	Renal	Lag 0	Lag 0	1.030	0.977	1.086	0.273	67
			Lag 1	0.979	0.913	1.051	0.565	62
			Lag 2	1.028	0.946	1.116	0.518	61
			Lag 3	1.071	0.978	1.172	0.139	62
		Lag 1	Lag 0	1.036	0.986	1.088	0.164	64
			Lag 1	0.989	0.926	1.056	0.744	66
			Lag 2	0.980	0.906	1.060	0.614	70
			Lag 3	1.075	0.993	1.163	0.072	72
		Lag 2	Lag 0	0.997	0.936	1.061	0.914	49
			Lag 1	0.994	0.929	1.064	0.865	57
			Lag 2	1.006	0.942	1.074	0.854	61
			Lag 3	1.055	0.995	1.119	0.071	64
		Lag 3	Lag 0	0.950	0.875	1.031	0.222	54
			Lag 1	0.975	0.894	1.064	0.576	56
			Lag 2	1.007	0.952	1.065	0.807	60

			Lag 3	1.017	0.967	1.070	0.506	59
	Respiratory	Lag 0	Lag 0	1.013	0.952	1.077	0.690	83
			Lag 1	1.035	0.976	1.098	0.254	73
			Lag 2	1.015	0.946	1.088	0.683	75
			Lag 3	1.084	1.018	1.154	**0.011**	73
		Lag 1	Lag 0	0.996	0.935	1.061	0.908	77
			Lag 1	1.043	0.990	1.099	0.117	78
			Lag 2	0.999	0.944	1.057	0.973	79
			Lag 3	1.087	1.018	1.160	**0.013**	78
		Lag 2	Lag 0	1.060	1.006	1.117	**0.030**	96
			Lag 1	1.041	1.002	1.082	**0.041**	89
			Lag 2	1.002	0.950	1.058	0.929	94
			Lag 3	1.043	0.982	1.107	0.173	91
		Lag 3	Lag 0	1.007	0.945	1.074	0.822	99
			Lag 1	0.984	0.928	1.042	0.575	60
			Lag 2	0.987	0.932	1.045	0.658	69
			Lag 3	1.033	0.979	1.090	0.234	69

Table S5 Stratified results for odds ratio for wildfire smoke effect, without extreme heat (OR01), and extreme heat effect, without wildfire smoke (OR10), with upper and lower limits (UL and LL, respectively) for 95% confidence intervals (CIs) and p-values (bolded indicates significant $p < 0.05$) for the joint effects of wildfire smoke and extreme heat. Separate models were run for combinations of lag 0-3 and each outcome of interest: all-natural cause, cardiovascular, cerebrovascular, renal, and respiratory morbidity.

Mod	OOI	Extreme Heat	Wildfire Smoke	Measure	Estimate	CI LL	CI UL	P-value
Age 18-49 years	All-natural	Lag 0	Lag 0	OR01	1.000	1.000	1.001	0.427
				OR10	1.022	1.017	1.027	**0.000**
			Lag 1	OR01	1.000	1.000	1.001	0.248
				OR10	1.023	1.018	1.028	**0.000**
			Lag 2	OR01	0.999	0.999	1.000	**0.017**
				OR10	1.024	1.019	1.029	**0.000**
			Lag 3	OR01	0.999	0.998	1.000	**0.005**
				OR10	1.022	1.018	1.027	**0.000**
		Lag 1	Lag 0	OR01	1.000	1.000	1.001	0.474
				OR10	1.023	1.018	1.028	**0.000**
			Lag 1	OR01	1.000	1.000	1.001	0.154
				OR10	1.024	1.020	1.029	**0.000**
			Lag 2	OR01	0.999	0.999	1.000	**0.036**
				OR10	1.026	1.021	1.030	**0.000**
			Lag 3	OR01	0.999	0.998	1.000	**0.004**
				OR10	1.023	1.019	1.028	**0.000**
		Lag 2	Lag 0	OR01	1.000	1.000	1.001	0.422
				OR10	1.016	1.012	1.021	**0.000**
			Lag 1	OR01	1.000	1.000	1.001	0.217
				OR10	1.017	1.013	1.022	**0.000**
			Lag 2	OR01	0.999	0.999	1.000	0.065
				OR10	1.019	1.015	1.024	**0.000**
			Lag 3	OR01	0.999	0.998	1.000	**0.003**
				OR10	1.017	1.012	1.021	**0.000**
		Lag 3	Lag 0	OR01	1.000	1.000	1.001	0.375
				OR10	1.016	1.011	1.020	**0.000**
			Lag 1	OR01	1.000	1.000	1.001	0.504
				OR10	1.016	1.011	1.020	**0.000**
			Lag 2	OR01	0.999	0.999	1.000	0.062
				OR10	1.018	1.014	1.023	**0.000**
			Lag 3	OR01	0.999	0.998	1.000	**0.004**
				OR10	1.016	1.012	1.021	**0.000**
	Cardiovascular	Lag 0	Lag 0	OR01	1.010	0.989	1.030	0.355
				OR10	0.986	0.824	1.181	0.880

			Lag 1	OR01	1.018	0.993	1.043	0.167
				OR10	1.002	0.836	1.200	0.986
			Lag 2	OR01	1.005	0.977	1.034	0.717
				OR10	0.989	0.826	1.184	0.903
			Lag 3	OR01	0.994	0.968	1.022	0.686
				OR10	0.994	0.831	1.190	0.951
		Lag 1	Lag 0	OR01	1.009	0.989	1.030	0.380
				OR10	1.166	0.982	1.384	0.080
			Lag 1	OR01	1.016	0.991	1.041	0.225
				OR10	1.166	0.982	1.384	0.080
			Lag 2	OR01	1.006	0.977	1.035	0.686
				OR10	1.179	0.992	1.401	0.061
			Lag 3	OR01	0.995	0.968	1.022	0.712
				OR10	1.183	0.996	1.404	0.055
		Lag 2	Lag 0	OR01	1.009	0.988	1.029	0.407
				OR10	1.174	0.993	1.389	0.060
			Lag 1	OR01	1.017	0.992	1.044	0.190
				OR10	1.182	1.000	1.397	0.051
			Lag 2	OR01	1.006	0.977	1.035	0.697
				OR10	1.186	1.003	1.403	**0.046**
			Lag 3	OR01	0.996	0.970	1.024	0.799
				OR10	1.206	1.019	1.427	**0.029**
		Lag 3	Lag 0	OR01	1.009	0.988	1.029	0.406
				OR10	1.142	0.966	1.349	0.120
			Lag 1	OR01	1.017	0.992	1.043	0.182
				OR10	1.151	0.974	1.360	0.098
			Lag 2	OR01	1.008	0.979	1.038	0.589
				OR10	1.157	0.979	1.367	0.087
			Lag 3	OR01	0.994	0.966	1.022	0.666
				OR10	1.154	0.976	1.364	0.095
	Cerebrovascular	Lag 0	Lag 0	OR01	0.982	0.955	1.009	0.190
				OR10	1.057	0.899	1.242	0.502
			Lag 1	OR01	0.980	0.954	1.006	0.125
				OR10	1.060	0.902	1.246	0.477
			Lag 2	OR01	0.992	0.971	1.014	0.492
				OR10	1.035	0.879	1.218	0.678
			Lag 3	OR01	0.970	0.934	1.008	0.124
				OR10	1.056	0.898	1.241	0.511
		Lag 1	Lag 0	OR01	0.982	0.955	1.009	0.183
				OR10	1.056	0.902	1.236	0.499
			Lag 1	OR01	0.979	0.953	1.005	0.116
				OR10	1.056	0.901	1.237	0.502

			Lag 2	OR01	0.995	0.975	1.016	0.660
				OR10	1.069	0.913	1.253	0.407
			Lag 3	OR01	0.978	0.945	1.013	0.217
				OR10	1.085	0.927	1.270	0.308
		Lag 2	Lag 0	OR01	0.983	0.957	1.009	0.204
				OR10	1.151	0.988	1.341	0.070
			Lag 1	OR01	0.980	0.954	1.006	0.134
				OR10	1.164	0.999	1.357	0.052
			Lag 2	OR01	0.996	0.976	1.017	0.720
				OR10	1.178	1.010	1.373	**0.037**
			Lag 3	OR01	0.974	0.939	1.011	0.168
				OR10	1.172	1.005	1.367	**0.043**
		Lag 3	Lag 0	OR01	0.987	0.963	1.012	0.299
				OR10	1.088	0.930	1.273	0.293
			Lag 1	OR01	0.985	0.960	1.010	0.224
				OR10	1.092	0.933	1.277	0.272
			Lag 2	OR01	0.995	0.974	1.016	0.649
				OR10	1.072	0.916	1.255	0.386
			Lag 3	OR01	0.977	0.943	1.013	0.208
				OR10	1.085	0.927	1.270	0.309
	Renal	Lag 0	Lag 0	OR01	1.000	0.972	1.029	0.987
				OR10	0.937	0.776	1.131	0.498
			Lag 1	OR01	1.017	0.988	1.046	0.256
				OR10	0.958	0.795	1.156	0.656
			Lag 2	OR01	1.002	0.967	1.038	0.906
				OR10	0.929	0.770	1.121	0.441
			Lag 3	OR01	0.970	0.924	1.019	0.224
				OR10	0.902	0.747	1.088	0.281
		Lag 1	Lag 0	OR01	1.003	0.976	1.031	0.840
				OR10	0.958	0.800	1.147	0.640
			Lag 1	OR01	1.016	0.988	1.046	0.267
				OR10	0.957	0.800	1.145	0.631
			Lag 2	OR01	1.005	0.970	1.040	0.790
				OR10	0.952	0.795	1.140	0.593
			Lag 3	OR01	0.968	0.920	1.018	0.208
				OR10	0.904	0.754	1.084	0.277
		Lag 2	Lag 0	OR01	1.004	0.977	1.032	0.782
				OR10	1.152	0.972	1.367	0.103
			Lag 1	OR01	1.017	0.988	1.047	0.259
				OR10	1.139	0.961	1.350	0.132
			Lag 2	OR01	0.999	0.964	1.036	0.978
				OR10	1.107	0.932	1.314	0.247

			Lag 3	OR01	0.959	0.905	1.016	0.156
				OR10	1.091	0.919	1.296	0.319
		Lag 3	Lag 0	OR01	0.999	0.971	1.029	0.972
				OR10	1.058	0.895	1.251	0.510
			Lag 1	OR01	1.015	0.987	1.044	0.304
				OR10	1.072	0.907	1.268	0.415
			Lag 2	OR01	1.001	0.966	1.038	0.954
				OR10	1.050	0.888	1.242	0.569
			Lag 3	OR01	0.970	0.921	1.021	0.247
				OR10	1.042	0.880	1.233	0.633
	Respiratory	Lag 0	Lag 0	OR01	0.978	0.941	1.017	0.274
				OR10	1.004	0.838	1.202	0.965
			Lag 1	OR01	0.985	0.955	1.017	0.357
				OR10	1.030	0.863	1.229	0.746
			Lag 2	OR01	0.998	0.969	1.028	0.905
				OR10	1.027	0.861	1.225	0.769
			Lag 3	OR01	0.995	0.970	1.021	0.720
				OR10	1.005	0.841	1.201	0.954
		Lag 1	Lag 0	OR01	0.982	0.946	1.020	0.343
				OR10	1.036	0.868	1.235	0.698
			Lag 1	OR01	0.983	0.952	1.015	0.303
				OR10	1.030	0.864	1.227	0.745
			Lag 2	OR01	0.997	0.968	1.028	0.862
				OR10	1.037	0.871	1.236	0.682
			Lag 3	OR01	0.995	0.969	1.021	0.689
				OR10	1.013	0.849	1.209	0.884
		Lag 2	Lag 0	OR01	0.970	0.929	1.013	0.165
				OR10	0.990	0.833	1.175	0.905
			Lag 1	OR01	0.986	0.955	1.018	0.380
				OR10	1.032	0.870	1.223	0.720
			Lag 2	OR01	0.998	0.969	1.028	0.893
				OR10	1.026	0.865	1.217	0.767
			Lag 3	OR01	0.998	0.974	1.023	0.882
				OR10	1.034	0.872	1.226	0.701
		Lag 3	Lag 0	OR01	0.987	0.951	1.024	0.473
				OR10	0.999	0.847	1.178	0.987
			Lag 1	OR01	0.992	0.964	1.022	0.612
				OR10	1.019	0.865	1.202	0.819
			Lag 2	OR01	0.999	0.970	1.030	0.964
				OR10	0.992	0.842	1.170	0.926
			Lag 3	OR01	0.998	0.974	1.023	0.892
				OR10	0.995	0.843	1.174	0.952

Age 50-64 years	All-natural	Lag 0	Lag 0	OR01	1.000	0.999	1.000	0.458
				OR10	1.021	1.016	1.027	**0.000**
			Lag 1	OR01	1.000	0.999	1.000	0.317
				OR10	1.022	1.016	1.028	**0.000**
			Lag 2	OR01	0.999	0.998	1.000	**0.040**
				OR10	1.022	1.016	1.028	**0.000**
			Lag 3	OR01	0.999	0.998	1.000	**0.006**
				OR10	1.021	1.015	1.027	**0.000**
		Lag 1	Lag 0	OR01	1.000	0.999	1.000	0.350
				OR10	1.020	1.014	1.026	**0.000**
			Lag 1	OR01	1.000	0.999	1.000	0.383
				OR10	1.021	1.016	1.027	**0.000**
			Lag 2	OR01	0.999	0.998	1.000	**0.037**
				OR10	1.021	1.015	1.026	**0.000**
			Lag 3	OR01	0.999	0.998	1.000	**0.006**
				OR10	1.020	1.014	1.026	**0.000**
		Lag 2	Lag 0	OR01	1.000	0.999	1.000	0.505
				OR10	1.019	1.013	1.025	**0.000**
			Lag 1	OR01	1.000	0.999	1.001	0.653
				OR10	1.021	1.016	1.027	**0.000**
			Lag 2	OR01	0.999	0.999	1.000	0.136
				OR10	1.021	1.016	1.027	**0.000**
			Lag 3	OR01	0.999	0.998	1.000	**0.013**
				OR10	1.020	1.014	1.026	**0.000**
		Lag 3	Lag 0	OR01	1.000	0.999	1.000	0.487
				OR10	1.015	1.009	1.021	**0.000**
			Lag 1	OR01	1.000	0.999	1.000	0.361
				OR10	1.016	1.010	1.022	**0.000**
			Lag 2	OR01	0.999	0.999	1.000	0.101
				OR10	1.017	1.011	1.022	**0.000**
			Lag 3	OR01	0.999	0.998	1.000	**0.012**
				OR10	1.016	1.010	1.022	**0.000**
	Cardiovascular	Lag 0	Lag 0	OR01	0.995	0.978	1.012	0.547
				OR10	0.973	0.866	1.094	0.649
			Lag 1	OR01	0.996	0.980	1.012	0.597
				OR10	0.962	0.856	1.082	0.523
			Lag 2	OR01	0.999	0.984	1.014	0.888
				OR10	0.966	0.859	1.085	0.557
			Lag 3	OR01	0.997	0.981	1.012	0.663
				OR10	0.978	0.870	1.098	0.703
		Lag 1	Lag 0	OR01	0.996	0.979	1.013	0.609
				OR10	1.051	0.937	1.179	0.398

			Lag 1	OR01	0.996	0.980	1.012	0.619
				OR10	1.035	0.922	1.161	0.562
			Lag 2	OR01	0.998	0.983	1.014	0.810
				OR10	1.028	0.915	1.154	0.641
			Lag 3	OR01	0.997	0.981	1.012	0.676
				OR10	1.048	0.934	1.175	0.424
		Lag 2	Lag 0	OR01	0.994	0.976	1.011	0.469
				OR10	1.016	0.909	1.135	0.780
			Lag 1	OR01	0.998	0.982	1.014	0.802
				OR10	1.029	0.921	1.149	0.615
			Lag 2	OR01	0.999	0.984	1.015	0.943
				OR10	1.022	0.914	1.142	0.708
			Lag 3	OR01	0.997	0.982	1.013	0.718
				OR10	1.029	0.921	1.149	0.618
		Lag 3	Lag 0	OR01	0.993	0.976	1.011	0.428
				OR10	0.968	0.865	1.083	0.566
			Lag 1	OR01	0.998	0.983	1.014	0.816
				OR10	0.989	0.884	1.106	0.845
			Lag 2	OR01	1.000	0.985	1.016	0.959
				OR10	0.985	0.881	1.101	0.791
			Lag 3	OR01	0.996	0.981	1.012	0.656
				OR10	0.984	0.880	1.100	0.772
	Cerebrovascular	Lag 0	Lag 0	OR01	1.010	0.998	1.022	0.101
				OR10	0.958	0.868	1.058	0.401
			Lag 1	OR01	1.007	0.996	1.019	0.217
				OR10	0.950	0.860	1.050	0.318
			Lag 2	OR01	0.996	0.983	1.009	0.588
				OR10	0.946	0.856	1.044	0.269
			Lag 3	OR01	0.993	0.980	1.007	0.330
				OR10	0.937	0.849	1.035	0.201
		Lag 1	Lag 0	OR01	1.010	0.998	1.022	0.115
				OR10	1.039	0.943	1.145	0.434
			Lag 1	OR01	1.008	0.997	1.020	0.168
				OR10	1.046	0.949	1.153	0.366
			Lag 2	OR01	0.998	0.985	1.011	0.710
				OR10	1.043	0.946	1.149	0.398
			Lag 3	OR01	0.993	0.979	1.007	0.320
				OR10	1.019	0.924	1.123	0.708
		Lag 2	Lag 0	OR01	1.009	0.997	1.021	0.150
				OR10	1.046	0.952	1.150	0.348
			Lag 1	OR01	1.008	0.996	1.019	0.199
				OR10	1.054	0.959	1.158	0.279

			Lag 2	OR01	0.996	0.983	1.009	0.546
				OR10	1.046	0.951	1.150	0.353
			Lag 3	OR01	0.992	0.979	1.007	0.290
				OR10	1.037	0.943	1.140	0.450
		Lag 3	Lag 0	OR01	1.008	0.996	1.020	0.206
				OR10	1.028	0.935	1.131	0.565
			Lag 1	OR01	1.008	0.996	1.019	0.188
				OR10	1.047	0.953	1.152	0.338
			Lag 2	OR01	0.996	0.982	1.010	0.545
				OR10	1.040	0.946	1.143	0.418
			Lag 3	OR01	0.993	0.979	1.007	0.313
				OR10	1.033	0.939	1.136	0.509
	Renal	Lag 0	Lag 0	OR01	0.986	0.948	1.025	0.476
				OR10	1.060	0.905	1.243	0.469
			Lag 1	OR01	0.974	0.934	1.016	0.226
				OR10	1.087	0.928	1.275	0.301
			Lag 2	OR01	0.987	0.954	1.021	0.435
				OR10	1.049	0.895	1.231	0.553
			Lag 3	OR01	0.982	0.942	1.023	0.389
				OR10	1.049	0.896	1.229	0.550
		Lag 1	Lag 0	OR01	0.984	0.945	1.025	0.440
				OR10	1.175	1.006	1.373	**0.041**
			Lag 1	OR01	0.959	0.914	1.006	0.085
				OR10	1.175	1.005	1.374	**0.043**
			Lag 2	OR01	0.985	0.952	1.020	0.407
				OR10	1.156	0.987	1.352	0.072
			Lag 3	OR01	0.984	0.945	1.025	0.446
				OR10	1.173	1.004	1.371	**0.044**
		Lag 2	Lag 0	OR01	0.995	0.960	1.031	0.777
				OR10	1.074	0.919	1.256	0.368
			Lag 1	OR01	0.959	0.915	1.006	0.084
				OR10	1.052	0.900	1.231	0.523
			Lag 2	OR01	0.991	0.960	1.022	0.563
				OR10	1.069	0.913	1.251	0.406
			Lag 3	OR01	0.986	0.946	1.027	0.487
				OR10	1.059	0.905	1.240	0.475
		Lag 3	Lag 0	OR01	0.998	0.963	1.034	0.897
				OR10	1.119	0.957	1.309	0.158
			Lag 1	OR01	0.968	0.927	1.010	0.136
				OR10	1.091	0.932	1.277	0.276
			Lag 2	OR01	0.992	0.962	1.023	0.628
				OR10	1.110	0.949	1.299	0.193

			Lag 3	OR01	0.986	0.946	1.027	0.499
				OR10	1.098	0.937	1.286	0.248
	Respiratory	Lag 0	Lag 0	OR01	1.005	0.984	1.027	0.621
				OR10	1.080	0.950	1.228	0.242
			Lag 1	OR01	1.006	0.989	1.023	0.495
				OR10	1.048	0.921	1.194	0.474
			Lag 2	OR01	1.027	1.007	1.049	**0.010**
				OR10	1.083	0.953	1.232	0.222
			Lag 3	OR01	1.018	0.999	1.037	0.059
				OR10	1.055	0.927	1.200	0.419
		Lag 1	Lag 0	OR01	1.005	0.984	1.027	0.632
				OR10	1.042	0.916	1.185	0.534
			Lag 1	OR01	1.007	0.990	1.025	0.402
				OR10	1.030	0.904	1.172	0.660
			Lag 2	OR01	1.029	1.008	1.051	**0.007**
				OR10	1.054	0.926	1.199	0.426
			Lag 3	OR01	1.020	1.002	1.039	**0.032**
				OR10	1.042	0.916	1.186	0.532
		Lag 2	Lag 0	OR01	1.001	0.978	1.024	0.947
				OR10	1.043	0.917	1.187	0.520
			Lag 1	OR01	1.007	0.990	1.024	0.442
				OR10	1.051	0.924	1.196	0.449
			Lag 2	OR01	1.027	1.006	1.049	**0.011**
				OR10	1.068	0.939	1.215	0.318
			Lag 3	OR01	1.019	1.000	1.038	**0.048**
				OR10	1.052	0.924	1.197	0.445
		Lag 3	Lag 0	OR01	1.005	0.984	1.026	0.671
				OR10	1.024	0.903	1.160	0.714
			Lag 1	OR01	1.009	0.992	1.026	0.315
				OR10	1.029	0.908	1.167	0.650
			Lag 2	OR01	1.029	1.008	1.050	**0.006**
				OR10	1.043	0.921	1.182	0.509
			Lag 3	OR01	1.021	1.002	1.040	**0.028**
				OR10	1.033	0.911	1.170	0.617
Age ≥ 65 years	All-natural	Lag 0	Lag 0	OR01	1.000	0.999	1.001	0.875
				OR10	1.027	1.022	1.031	**0.000**
			Lag 1	OR01	1.000	0.999	1.000	0.093
				OR10	1.027	1.022	1.031	**0.000**
			Lag 2	OR01	0.999	0.999	1.000	**0.017**
				OR10	1.028	1.023	1.032	**0.000**
			Lag 3	OR01	0.999	0.999	1.000	**0.004**
				OR10	1.027	1.023	1.032	**0.000**

		Lag 1	Lag 0	OR01	1.000	0.999	1.000	0.820
				OR10	1.025	1.020	1.029	**0.000**
			Lag 1	OR01	1.000	0.999	1.000	0.096
				OR10	1.026	1.021	1.030	**0.000**
			Lag 2	OR01	0.999	0.999	1.000	**0.010**
				OR10	1.026	1.022	1.031	**0.000**
			Lag 3	OR01	0.999	0.999	1.000	**0.005**
				OR10	1.026	1.022	1.031	**0.000**
		Lag 2	Lag 0	OR01	1.000	0.999	1.001	0.888
				OR10	1.019	1.015	1.024	**0.000**
			Lag 1	OR01	1.000	0.999	1.000	0.103
				OR10	1.021	1.016	1.025	**0.000**
			Lag 2	OR01	0.999	0.999	1.000	**0.013**
				OR10	1.021	1.017	1.026	**0.000**
			Lag 3	OR01	0.999	0.999	1.000	**0.014**
				OR10	1.022	1.018	1.027	**0.000**
		Lag 3	Lag 0	OR01	1.000	0.999	1.001	0.883
				OR10	1.011	1.007	1.015	**0.000**
			Lag 1	OR01	1.000	0.999	1.000	0.101
				OR10	1.012	1.008	1.016	**0.000**
			Lag 2	OR01	0.999	0.999	1.000	**0.022**
				OR10	1.013	1.009	1.018	**0.000**
			Lag 3	OR01	0.999	0.999	1.000	**0.008**
				OR10	1.013	1.009	1.018	**0.000**
	Cardiovascular	Lag 0	Lag 0	OR01	1.003	0.992	1.014	0.641
				OR10	1.058	0.981	1.141	0.145
			Lag 1	OR01	1.008	0.998	1.018	0.123
				OR10	1.062	0.985	1.145	0.119
			Lag 2	OR01	1.000	0.989	1.010	0.936
				OR10	1.064	0.986	1.148	0.108
			Lag 3	OR01	1.001	0.990	1.011	0.912
				OR10	1.072	0.994	1.156	0.072
		Lag 1	Lag 0	OR01	1.002	0.991	1.013	0.771
				OR10	1.041	0.967	1.122	0.286
			Lag 1	OR01	1.009	0.998	1.019	0.102
				OR10	1.053	0.977	1.134	0.176
			Lag 2	OR01	0.999	0.988	1.009	0.788
				OR10	1.047	0.971	1.128	0.231
			Lag 3	OR01	1.000	0.989	1.011	0.934
				OR10	1.050	0.975	1.131	0.200
		Lag 2	Lag 0	OR01	1.001	0.990	1.013	0.796
				OR10	1.012	0.940	1.091	0.745

			Lag 1	OR01	1.009	0.999	1.020	0.081
				OR10	1.029	0.956	1.108	0.448
			Lag 2	OR01	1.000	0.989	1.010	0.965
				OR10	1.029	0.955	1.108	0.454
			Lag 3	OR01	1.000	0.989	1.011	0.968
				OR10	1.027	0.954	1.106	0.479
		Lag 3	Lag 0	OR01	1.002	0.991	1.013	0.692
				OR10	1.020	0.947	1.099	0.606
			Lag 1	OR01	1.008	0.998	1.019	0.122
				OR10	1.030	0.957	1.109	0.432
			Lag 2	OR01	1.000	0.990	1.011	0.976
				OR10	1.036	0.962	1.116	0.344
			Lag 3	OR01	0.999	0.989	1.011	0.928
				OR10	1.030	0.957	1.110	0.430
	Cerebrovascular	Lag 0	Lag 0	OR01	1.003	0.996	1.011	0.366
				OR10	1.046	0.984	1.113	0.149
			Lag 1	OR01	1.001	0.993	1.009	0.831
				OR10	1.048	0.986	1.114	0.134
			Lag 2	OR01	1.000	0.992	1.007	0.953
				OR10	1.049	0.986	1.115	0.129
			Lag 3	OR01	0.998	0.990	1.006	0.581
				OR10	1.046	0.984	1.112	0.152
		Lag 1	Lag 0	OR01	1.003	0.996	1.010	0.416
				OR10	1.024	0.963	1.089	0.443
			Lag 1	OR01	1.000	0.992	1.008	0.997
				OR10	1.024	0.963	1.089	0.457
			Lag 2	OR01	0.999	0.991	1.007	0.849
				OR10	1.025	0.963	1.090	0.442
			Lag 3	OR01	0.998	0.990	1.006	0.615
				OR10	1.030	0.968	1.095	0.353
		Lag 2	Lag 0	OR01	1.003	0.996	1.011	0.345
				OR10	1.019	0.959	1.083	0.547
			Lag 1	OR01	1.000	0.992	1.008	0.918
				OR10	1.013	0.953	1.077	0.681
			Lag 2	OR01	1.000	0.992	1.008	0.982
				OR10	1.023	0.962	1.087	0.475
			Lag 3	OR01	0.998	0.990	1.006	0.573
				OR10	1.018	0.957	1.082	0.572
		Lag 3	Lag 0	OR01	1.003	0.996	1.010	0.419
				OR10	0.999	0.940	1.061	0.966
			Lag 1	OR01	0.999	0.991	1.007	0.836
				OR10	0.995	0.936	1.057	0.866

			Lag 2	OR01	0.999	0.991	1.007	0.795
				OR10	1.000	0.941	1.062	0.994
			Lag 3	OR01	0.997	0.989	1.005	0.448
				OR10	0.997	0.938	1.060	0.929
	Renal	Lag 0	Lag 0	OR01	0.989	0.963	1.017	0.441
				OR10	1.156	1.043	1.281	**0.006**
			Lag 1	OR01	0.985	0.961	1.009	0.223
				OR10	1.154	1.040	1.279	**0.007**
			Lag 2	OR01	1.015	0.999	1.031	0.071
				OR10	1.158	1.045	1.284	**0.005**
			Lag 3	OR01	1.007	0.991	1.024	0.387
				OR10	1.165	1.052	1.291	**0.003**
		Lag 1	Lag 0	OR01	0.984	0.956	1.012	0.260
				OR10	1.148	1.038	1.269	**0.007**
			Lag 1	OR01	0.989	0.966	1.011	0.326
				OR10	1.173	1.061	1.296	**0.002**
			Lag 2	OR01	1.017	1.001	1.033	**0.038**
				OR10	1.179	1.066	1.303	**0.001**
			Lag 3	OR01	1.006	0.989	1.023	0.485
				OR10	1.157	1.047	1.280	**0.004**
		Lag 2	Lag 0	OR01	0.990	0.964	1.017	0.455
				OR10	1.049	0.948	1.161	0.354
			Lag 1	OR01	0.989	0.967	1.011	0.330
				OR10	1.063	0.960	1.176	0.240
			Lag 2	OR01	1.015	0.999	1.031	0.064
				OR10	1.057	0.954	1.170	0.288
			Lag 3	OR01	1.005	0.988	1.022	0.593
				OR10	1.048	0.947	1.160	0.367
		Lag 3	Lag 0	OR01	0.997	0.973	1.022	0.818
				OR10	1.020	0.922	1.129	0.699
			Lag 1	OR01	0.989	0.967	1.011	0.334
				OR10	1.019	0.921	1.127	0.720
			Lag 2	OR01	1.014	0.998	1.031	0.081
				OR10	1.009	0.912	1.117	0.858
			Lag 3	OR01	1.006	0.989	1.024	0.494
				OR10	1.012	0.914	1.119	0.824
	Respiratory	Lag 0	Lag 0	OR01	0.994	0.980	1.009	0.458
				OR10	0.904	0.818	0.998	**0.046**
			Lag 1	OR01	1.002	0.991	1.013	0.764
				OR10	0.899	0.814	0.993	**0.036**
			Lag 2	OR01	0.997	0.983	1.011	0.654
				OR10	0.900	0.814	0.994	**0.037**

			Lag 3	OR01	0.992	0.977	1.008	0.331
				OR10	0.889	0.805	0.981	**0.020**
		Lag 1	Lag 0	OR01	0.995	0.980	1.010	0.487
				OR10	0.994	0.904	1.094	0.909
			Lag 1	OR01	1.001	0.990	1.012	0.847
				OR10	0.980	0.890	1.078	0.677
			Lag 2	OR01	0.997	0.983	1.011	0.668
				OR10	0.987	0.897	1.086	0.791
			Lag 3	OR01	0.991	0.976	1.007	0.281
				OR10	0.965	0.877	1.063	0.471
		Lag 2	Lag 0	OR01	0.994	0.979	1.009	0.435
				OR10	0.981	0.891	1.080	0.699
			Lag 1	OR01	0.999	0.988	1.011	0.909
				OR10	0.966	0.878	1.063	0.478
			Lag 2	OR01	0.997	0.983	1.012	0.698
				OR10	0.982	0.892	1.081	0.711
			Lag 3	OR01	0.993	0.977	1.008	0.349
				OR10	0.968	0.878	1.066	0.506
		Lag 3	Lag 0	OR01	0.994	0.979	1.009	0.415
				OR10	1.064	0.970	1.168	0.187
			Lag 1	OR01	0.998	0.986	1.010	0.787
				OR10	1.055	0.962	1.157	0.259
			Lag 2	OR01	0.997	0.982	1.011	0.661
				OR10	1.065	0.971	1.169	0.180
			Lag 3	OR01	0.991	0.976	1.007	0.280
				OR10	1.048	0.954	1.150	0.329
Asian	All-natural	Lag 0	Lag 0	OR01	1.000	0.999	1.001	0.606
				OR10	1.029	1.019	1.038	**0.000**
			Lag 1	OR01	1.000	0.999	1.001	0.840
				OR10	1.028	1.019	1.038	**0.000**
			Lag 2	OR01	1.000	0.999	1.001	0.359
				OR10	1.032	1.022	1.042	**0.000**
			Lag 3	OR01	0.999	0.998	1.000	0.111
				OR10	1.031	1.021	1.041	**0.000**
		Lag 1	Lag 0	OR01	1.000	0.999	1.001	0.910
				OR10	1.027	1.017	1.036	**0.000**
			Lag 1	OR01	1.000	0.999	1.001	0.635
				OR10	1.027	1.017	1.036	**0.000**
			Lag 2	OR01	0.999	0.998	1.000	0.206
				OR10	1.029	1.020	1.039	**0.000**
			Lag 3	OR01	0.999	0.998	1.000	0.078
				OR10	1.029	1.019	1.038	**0.000**

		Lag 2	Lag 0	OR01	1.000	0.999	1.001	0.787
				OR10	1.017	1.008	1.027	**0.000**
			Lag 1	OR01	1.000	0.999	1.001	0.651
				OR10	1.018	1.009	1.027	**0.000**
			Lag 2	OR01	0.999	0.998	1.000	0.156
				OR10	1.019	1.010	1.029	**0.000**
			Lag 3	OR01	0.999	0.998	1.000	0.104
				OR10	1.021	1.011	1.030	**0.000**
		Lag 3	Lag 0	OR01	1.000	0.999	1.001	0.679
				OR10	1.014	1.004	1.023	**0.004**
			Lag 1	OR01	1.000	0.999	1.001	0.934
				OR10	1.015	1.006	1.024	**0.001**
			Lag 2	OR01	0.999	0.998	1.000	0.149
				OR10	1.015	1.006	1.025	**0.001**
			Lag 3	OR01	0.999	0.998	1.000	0.088
				OR10	1.016	1.007	1.026	**0.001**
	Cardiovascular	Lag 0	Lag 0	OR01	1.002	0.981	1.024	0.861
				OR10	0.917	0.744	1.131	0.420
			Lag 1	OR01	0.998	0.966	1.032	0.929
				OR10	0.891	0.723	1.097	0.276
			Lag 2	OR01	0.976	0.946	1.007	0.130
				OR10	0.908	0.737	1.118	0.363
			Lag 3	OR01	0.975	0.943	1.009	0.152
				OR10	0.931	0.757	1.145	0.499
		Lag 1	Lag 0	OR01	0.999	0.976	1.022	0.919
				OR10	1.076	0.876	1.322	0.487
			Lag 1	OR01	0.991	0.954	1.028	0.621
				OR10	1.066	0.867	1.312	0.543
			Lag 2	OR01	0.969	0.933	1.006	0.101
				OR10	1.067	0.868	1.311	0.540
			Lag 3	OR01	0.971	0.936	1.008	0.127
				OR10	1.084	0.882	1.332	0.445
		Lag 2	Lag 0	OR01	0.998	0.976	1.022	0.895
				OR10	0.954	0.780	1.167	0.645
			Lag 1	OR01	0.997	0.963	1.031	0.844
				OR10	0.959	0.784	1.172	0.680
			Lag 2	OR01	0.971	0.937	1.006	0.108
				OR10	0.952	0.778	1.165	0.632
			Lag 3	OR01	0.971	0.935	1.008	0.124
				OR10	0.961	0.786	1.176	0.700
		Lag 3	Lag 0	OR01	0.999	0.976	1.022	0.908
				OR10	0.875	0.712	1.076	0.207

			Lag 1	OR01	0.990	0.956	1.026	0.585
				OR10	0.848	0.688	1.046	0.123
			Lag 2	OR01	0.973	0.938	1.008	0.129
				OR10	0.885	0.720	1.087	0.244
			Lag 3	OR01	0.972	0.937	1.009	0.134
				OR10	0.889	0.722	1.094	0.267
	Cerebrovascular	Lag 0	Lag 0	OR01	1.010	1.000	1.021	0.052
				OR10	0.940	0.810	1.091	0.414
			Lag 1	OR01	1.013	1.001	1.026	**0.040**
				OR10	0.956	0.824	1.110	0.556
			Lag 2	OR01	1.003	0.991	1.015	0.607
				OR10	0.953	0.821	1.105	0.521
			Lag 3	OR01	0.995	0.980	1.009	0.454
				OR10	0.941	0.812	1.091	0.422
		Lag 1	Lag 0	OR01	1.011	1.000	1.021	**0.045**
				OR10	1.004	0.867	1.162	0.955
			Lag 1	OR01	1.015	1.002	1.028	**0.027**
				OR10	1.029	0.888	1.192	0.707
			Lag 2	OR01	1.003	0.991	1.015	0.646
				OR10	0.998	0.861	1.156	0.974
			Lag 3	OR01	0.995	0.981	1.009	0.488
				OR10	1.005	0.868	1.164	0.942
		Lag 2	Lag 0	OR01	1.011	1.000	1.021	**0.045**
				OR10	1.113	0.967	1.281	0.135
			Lag 1	OR01	1.014	1.001	1.027	**0.038**
				OR10	1.114	0.968	1.282	0.132
			Lag 2	OR01	1.002	0.990	1.014	0.753
				OR10	1.091	0.947	1.257	0.228
			Lag 3	OR01	0.994	0.980	1.009	0.446
				OR10	1.099	0.955	1.265	0.188
		Lag 3	Lag 0	OR01	1.010	1.000	1.020	0.056
				OR10	1.085	0.942	1.249	0.259
			Lag 1	OR01	1.012	0.999	1.025	0.076
				OR10	1.081	0.938	1.245	0.283
			Lag 2	OR01	0.999	0.986	1.012	0.877
				OR10	1.050	0.911	1.210	0.503
			Lag 3	OR01	0.993	0.978	1.008	0.340
				OR10	1.069	0.927	1.232	0.360
	Renal	Lag 0	Lag 0	OR01	0.942	0.870	1.019	0.136
				OR10	1.130	0.854	1.497	0.393
			Lag 1	OR01	0.998	0.948	1.052	0.953
				OR10	1.065	0.804	1.410	0.663

			Lag 2	OR01	0.999	0.964	1.035	0.960
				OR10	1.087	0.820	1.440	0.563
			Lag 3	OR01	1.015	0.980	1.052	0.402
				OR10	1.169	0.886	1.543	0.270
		Lag 1	Lag 0	OR01	0.932	0.856	1.015	0.104
				OR10	1.166	0.883	1.540	0.278
			Lag 1	OR01	1.002	0.952	1.054	0.952
				OR10	1.137	0.859	1.504	0.370
			Lag 2	OR01	1.003	0.970	1.038	0.863
				OR10	1.207	0.917	1.590	0.180
			Lag 3	OR01	1.011	0.975	1.049	0.543
				OR10	1.163	0.881	1.535	0.285
		Lag 2	Lag 0	OR01	0.937	0.865	1.016	0.115
				OR10	1.125	0.857	1.477	0.398
			Lag 1	OR01	0.999	0.949	1.052	0.974
				OR10	1.060	0.804	1.397	0.679
			Lag 2	OR01	1.002	0.968	1.037	0.898
				OR10	1.134	0.864	1.489	0.366
			Lag 3	OR01	1.016	0.979	1.053	0.403
				OR10	1.144	0.870	1.504	0.335
		Lag 3	Lag 0	OR01	0.927	0.848	1.014	0.097
				OR10	1.107	0.851	1.441	0.448
			Lag 1	OR01	0.998	0.948	1.051	0.950
				OR10	1.066	0.818	1.389	0.636
			Lag 2	OR01	1.005	0.970	1.040	0.797
				OR10	1.143	0.880	1.484	0.315
			Lag 3	OR01	1.017	0.981	1.054	0.365
				OR10	1.158	0.891	1.506	0.273
	Respiratory	Lag 0	Lag 0	OR01	1.003	0.982	1.025	0.751
				OR10	0.952	0.743	1.221	0.700
			Lag 1	OR01	0.998	0.978	1.018	0.841
				OR10	0.929	0.722	1.194	0.563
			Lag 2	OR01	0.996	0.975	1.019	0.741
				OR10	0.963	0.751	1.235	0.769
			Lag 3	OR01	0.985	0.958	1.014	0.302
				OR10	0.928	0.722	1.192	0.558
		Lag 1	Lag 0	OR01	1.003	0.981	1.025	0.814
				OR10	0.998	0.784	1.269	0.984
			Lag 1	OR01	0.996	0.975	1.017	0.720
				OR10	0.960	0.752	1.225	0.740
			Lag 2	OR01	0.997	0.975	1.019	0.788
				OR10	1.025	0.804	1.307	0.840

			Lag 3	OR01	0.985	0.957	1.013	0.290
				OR10	0.984	0.772	1.254	0.893
		Lag 2	Lag 0	OR01	1.002	0.980	1.025	0.844
				OR10	0.995	0.785	1.261	0.967
			Lag 1	OR01	0.997	0.977	1.018	0.781
				OR10	0.987	0.777	1.253	0.912
			Lag 2	OR01	0.998	0.977	1.020	0.885
				OR10	1.066	0.838	1.355	0.603
			Lag 3	OR01	0.985	0.957	1.014	0.298
				OR10	0.997	0.784	1.268	0.982
		Lag 3	Lag 0	OR01	1.003	0.982	1.025	0.762
				OR10	1.005	0.786	1.285	0.969
			Lag 1	OR01	0.997	0.976	1.018	0.771
				OR10	0.965	0.753	1.237	0.778
			Lag 2	OR01	0.995	0.973	1.018	0.689
				OR10	0.996	0.779	1.274	0.976
			Lag 3	OR01	0.984	0.956	1.013	0.290
				OR10	0.979	0.765	1.252	0.866
Black	All-natural	Lag 0	Lag 0	OR01	1.000	0.999	1.001	0.855
				OR10	1.026	1.017	1.036	**0.000**
			Lag 1	OR01	1.001	1.000	1.002	0.055
				OR10	1.027	1.018	1.037	**0.000**
			Lag 2	OR01	1.000	0.999	1.001	0.714
				OR10	1.026	1.016	1.035	**0.000**
			Lag 3	OR01	1.000	0.999	1.001	0.626
				OR10	1.025	1.016	1.035	**0.000**
		Lag 1	Lag 0	OR01	1.000	0.999	1.001	0.864
				OR10	1.030	1.020	1.039	**0.000**
			Lag 1	OR01	1.001	1.000	1.002	0.114
				OR10	1.030	1.021	1.040	**0.000**
			Lag 2	OR01	1.000	0.999	1.001	0.686
				OR10	1.031	1.022	1.041	**0.000**
			Lag 3	OR01	1.000	0.999	1.001	0.627
				OR10	1.030	1.021	1.040	**0.000**
		Lag 2	Lag 0	OR01	1.000	0.999	1.001	0.727
				OR10	1.023	1.014	1.033	**0.000**
			Lag 1	OR01	1.001	1.000	1.002	0.120
				OR10	1.025	1.016	1.035	**0.000**
			Lag 2	OR01	1.000	0.999	1.001	0.575
				OR10	1.027	1.018	1.036	**0.000**
			Lag 3	OR01	1.000	0.999	1.001	0.834
				OR10	1.027	1.018	1.037	**0.000**

		Lag 3	Lag 0	OR01	1.000	0.999	1.001	0.856
				OR10	1.015	1.006	1.024	**0.001**
			Lag 1	OR01	1.001	1.000	1.002	0.148
				OR10	1.016	1.007	1.025	**0.001**
			Lag 2	OR01	1.000	0.999	1.001	0.565
				OR10	1.018	1.009	1.027	**0.000**
			Lag 3	OR01	1.000	0.999	1.001	0.739
				OR10	1.018	1.008	1.027	**0.000**
	Cardiovascular	Lag 0	Lag 0	OR01	0.992	0.967	1.017	0.521
				OR10	1.147	0.956	1.376	0.141
			Lag 1	OR01	1.028	1.003	1.054	**0.029**
				OR10	1.191	0.993	1.428	0.059
			Lag 2	OR01	1.002	0.983	1.022	0.817
				OR10	1.158	0.966	1.389	0.113
			Lag 3	OR01	0.994	0.967	1.022	0.684
				OR10	1.222	1.020	1.462	**0.029**
		Lag 1	Lag 0	OR01	0.992	0.968	1.017	0.547
				OR10	1.084	0.903	1.301	0.389
			Lag 1	OR01	1.025	1.001	1.050	**0.045**
				OR10	1.098	0.914	1.318	0.320
			Lag 2	OR01	0.998	0.976	1.020	0.844
				OR10	1.048	0.869	1.263	0.626
			Lag 3	OR01	0.987	0.958	1.017	0.402
				OR10	1.095	0.911	1.316	0.334
		Lag 2	Lag 0	OR01	0.992	0.968	1.017	0.524
				OR10	1.027	0.853	1.237	0.777
			Lag 1	OR01	1.030	1.004	1.057	**0.026**
				OR10	1.069	0.888	1.287	0.480
			Lag 2	OR01	1.002	0.982	1.022	0.851
				OR10	1.042	0.865	1.257	0.664
			Lag 3	OR01	0.993	0.965	1.021	0.614
				OR10	1.081	0.897	1.304	0.412
		Lag 3	Lag 0	OR01	0.997	0.975	1.019	0.760
				OR10	1.150	0.960	1.377	0.130
			Lag 1	OR01	1.031	1.004	1.057	**0.022**
				OR10	1.163	0.973	1.391	0.098
			Lag 2	OR01	1.004	0.984	1.024	0.696
				OR10	1.149	0.960	1.376	0.129
			Lag 3	OR01	0.993	0.965	1.021	0.609
				OR10	1.168	0.975	1.398	0.092
	Cerebrovascular	Lag 0	Lag 0	OR01	0.986	0.966	1.006	0.175
				OR10	1.042	0.884	1.228	0.623

			Lag 1	OR01	0.992	0.974	1.010	0.389
				OR10	1.037	0.878	1.224	0.669
			Lag 2	OR01	0.987	0.967	1.007	0.203
				OR10	1.028	0.871	1.213	0.743
			Lag 3	OR01	0.990	0.971	1.009	0.309
				OR10	0.994	0.842	1.174	0.943
		Lag 1	Lag 0	OR01	0.986	0.966	1.006	0.174
				OR10	1.137	0.966	1.338	0.122
			Lag 1	OR01	0.991	0.973	1.010	0.347
				OR10	1.121	0.951	1.320	0.174
			Lag 2	OR01	0.988	0.969	1.008	0.248
				OR10	1.138	0.966	1.342	0.122
			Lag 3	OR01	0.991	0.972	1.010	0.341
				OR10	1.095	0.929	1.292	0.278
		Lag 2	Lag 0	OR01	0.985	0.965	1.006	0.153
				OR10	1.030	0.880	1.207	0.709
			Lag 1	OR01	0.990	0.971	1.009	0.285
				OR10	1.024	0.875	1.200	0.764
			Lag 2	OR01	0.988	0.968	1.008	0.246
				OR10	1.042	0.890	1.220	0.609
			Lag 3	OR01	0.990	0.971	1.010	0.324
				OR10	1.022	0.872	1.197	0.789
		Lag 3	Lag 0	OR01	0.983	0.962	1.004	0.118
				OR10	1.089	0.928	1.279	0.295
			Lag 1	OR01	0.993	0.975	1.011	0.436
				OR10	1.136	0.970	1.331	0.114
			Lag 2	OR01	0.987	0.966	1.008	0.222
				OR10	1.134	0.967	1.328	0.121
			Lag 3	OR01	0.991	0.972	1.011	0.375
				OR10	1.124	0.958	1.319	0.152
	Renal	Lag 0	Lag 0	OR01	1.030	0.982	1.079	0.223
				OR10	1.212	0.922	1.595	0.169
			Lag 1	OR01	0.997	0.947	1.050	0.916
				OR10	1.195	0.906	1.577	0.207
			Lag 2	OR01	1.044	0.993	1.098	0.089
				OR10	1.290	0.984	1.692	0.065
			Lag 3	OR01	1.015	0.963	1.071	0.574
				OR10	1.229	0.934	1.617	0.141
		Lag 1	Lag 0	OR01	1.025	0.978	1.075	0.296
				OR10	1.234	0.948	1.606	0.117
			Lag 1	OR01	1.009	0.964	1.056	0.708
				OR10	1.333	1.029	1.728	**0.030**

			Lag 2	OR01	1.048	0.996	1.102	0.073
				OR10	1.355	1.045	1.758	**0.022**
			Lag 3	OR01	1.020	0.968	1.075	0.464
				OR10	1.314	1.012	1.706	**0.041**
		Lag 2	Lag 0	OR01	1.040	0.993	1.089	0.100
				OR10	1.118	0.865	1.446	0.393
			Lag 1	OR01	1.010	0.965	1.056	0.674
				OR10	1.180	0.915	1.521	0.203
			Lag 2	OR01	1.037	0.990	1.087	0.124
				OR10	1.105	0.853	1.433	0.449
			Lag 3	OR01	1.018	0.965	1.073	0.519
				OR10	1.121	0.864	1.454	0.390
		Lag 3	Lag 0	OR01	1.037	0.990	1.086	0.124
				OR10	1.074	0.832	1.388	0.583
			Lag 1	OR01	1.009	0.965	1.056	0.687
				OR10	1.135	0.883	1.459	0.325
			Lag 2	OR01	1.043	0.993	1.094	0.092
				OR10	1.122	0.871	1.445	0.374
			Lag 3	OR01	1.017	0.965	1.072	0.531
				OR10	1.091	0.845	1.410	0.504
	Respiratory	Lag 0	Lag 0	OR01	0.977	0.937	1.018	0.260
				OR10	0.874	0.707	1.080	0.213
			Lag 1	OR01	0.995	0.970	1.021	0.717
				OR10	0.846	0.683	1.048	0.126
			Lag 2	OR01	1.005	0.978	1.033	0.735
				OR10	0.877	0.709	1.084	0.224
			Lag 3	OR01	1.005	0.975	1.035	0.766
				OR10	0.835	0.673	1.035	0.100
		Lag 1	Lag 0	OR01	0.979	0.940	1.019	0.291
				OR10	0.927	0.753	1.141	0.473
			Lag 1	OR01	0.995	0.970	1.022	0.731
				OR10	0.891	0.721	1.101	0.284
			Lag 2	OR01	1.007	0.981	1.035	0.588
				OR10	0.943	0.765	1.161	0.579
			Lag 3	OR01	1.006	0.976	1.036	0.711
				OR10	0.891	0.720	1.103	0.289
		Lag 2	Lag 0	OR01	0.976	0.937	1.017	0.241
				OR10	0.892	0.725	1.097	0.280
			Lag 1	OR01	0.997	0.972	1.022	0.791
				OR10	0.900	0.732	1.106	0.317
			Lag 2	OR01	1.004	0.976	1.033	0.774
				OR10	0.902	0.733	1.110	0.328

			Lag 3	OR01	1.007	0.977	1.037	0.648
				OR10	0.884	0.716	1.091	0.251
		Lag 3	Lag 0	OR01	0.981	0.944	1.019	0.317
				OR10	0.986	0.808	1.202	0.888
			Lag 1	OR01	1.000	0.978	1.023	0.987
				OR10	1.009	0.829	1.228	0.926
			Lag 2	OR01	1.006	0.979	1.034	0.652
				OR10	0.998	0.818	1.217	0.984
			Lag 3	OR01	1.008	0.979	1.038	0.591
				OR10	0.975	0.797	1.193	0.806
Female	All-natural	Lag 0	Lag 0	OR01	1.000	0.999	1.000	0.763
				OR10	1.020	1.016	1.024	**0.000**
			Lag 1	OR01	1.000	0.999	1.000	0.265
				OR10	1.020	1.016	1.024	**0.000**
			Lag 2	OR01	0.999	0.999	1.000	**0.000**
				OR10	1.021	1.018	1.025	**0.000**
			Lag 3	OR01	0.999	0.999	1.000	**0.000**
				OR10	1.020	1.016	1.024	**0.000**
		Lag 1	Lag 0	OR01	1.000	0.999	1.000	0.663
				OR10	1.021	1.017	1.024	**0.000**
			Lag 1	OR01	1.000	0.999	1.000	0.356
				OR10	1.021	1.018	1.025	**0.000**
			Lag 2	OR01	0.999	0.999	1.000	**0.000**
				OR10	1.022	1.019	1.026	**0.000**
			Lag 3	OR01	0.999	0.999	1.000	**0.000**
				OR10	1.021	1.018	1.025	**0.000**
		Lag 2	Lag 0	OR01	1.000	0.999	1.000	0.778
				OR10	1.017	1.013	1.021	**0.000**
			Lag 1	OR01	1.000	0.999	1.000	0.420
				OR10	1.018	1.014	1.022	**0.000**
			Lag 2	OR01	0.999	0.999	1.000	**0.002**
				OR10	1.019	1.016	1.023	**0.000**
			Lag 3	OR01	0.999	0.999	1.000	**0.000**
				OR10	1.018	1.015	1.022	**0.000**
		Lag 3	Lag 0	OR01	1.000	0.999	1.000	0.787
				OR10	1.014	1.011	1.018	**0.000**
			Lag 1	OR01	1.000	0.999	1.000	0.238
				OR10	1.015	1.011	1.018	**0.000**
			Lag 2	OR01	0.999	0.999	1.000	**0.001**
				OR10	1.016	1.013	1.020	**0.000**
			Lag 3	OR01	0.999	0.999	1.000	**0.000**
				OR10	1.015	1.012	1.019	**0.000**

	Cardiovascular	Lag 0	Lag 0	OR01	0.999	0.985	1.013	0.857
				OR10	0.983	0.895	1.079	0.714
			Lag 1	OR01	0.996	0.979	1.013	0.641
				OR10	0.999	0.910	1.097	0.985
			Lag 2	OR01	0.995	0.980	1.011	0.551
				OR10	0.991	0.903	1.088	0.856
			Lag 3	OR01	0.983	0.966	1.000	0.050
				OR10	1.008	0.919	1.106	0.865
		Lag 1	Lag 0	OR01	0.998	0.984	1.012	0.785
				OR10	1.025	0.937	1.120	0.591
			Lag 1	OR01	0.996	0.979	1.013	0.646
				OR10	1.042	0.953	1.139	0.370
			Lag 2	OR01	0.995	0.979	1.011	0.520
				OR10	1.033	0.945	1.129	0.477
			Lag 3	OR01	0.979	0.961	0.997	**0.024**
				OR10	1.032	0.944	1.128	0.494
		Lag 2	Lag 0	OR01	0.996	0.982	1.011	0.603
				OR10	1.040	0.953	1.134	0.376
			Lag 1	OR01	0.999	0.982	1.016	0.934
				OR10	1.073	0.984	1.170	0.112
			Lag 2	OR01	0.996	0.981	1.012	0.648
				OR10	1.064	0.976	1.161	0.160
			Lag 3	OR01	0.982	0.964	1.000	**0.045**
				OR10	1.068	0.980	1.165	0.135
		Lag 3	Lag 0	OR01	0.997	0.983	1.011	0.670
				OR10	1.064	0.975	1.160	0.162
			Lag 1	OR01	0.998	0.981	1.015	0.787
				OR10	1.094	1.004	1.191	**0.041**
			Lag 2	OR01	0.996	0.981	1.012	0.622
				OR10	1.089	0.999	1.187	0.052
			Lag 3	OR01	0.982	0.965	1.000	0.050
				OR10	1.097	1.006	1.196	**0.036**
	Cerebrovascular	Lag 0	Lag 0	OR01	1.003	0.995	1.012	0.413
				OR10	1.041	0.970	1.117	0.264
			Lag 1	OR01	1.001	0.992	1.010	0.841
				OR10	1.041	0.971	1.117	0.259
			Lag 2	OR01	0.997	0.988	1.007	0.558
				OR10	1.048	0.977	1.124	0.191
			Lag 3	OR01	0.992	0.981	1.002	0.110
				OR10	1.035	0.965	1.110	0.341
		Lag 1	Lag 0	OR01	1.003	0.995	1.011	0.450
				OR10	1.072	1.000	1.150	0.051

			Lag 1	OR01	1.000	0.992	1.009	0.926
				OR10	1.072	1.000	1.150	0.050
			Lag 2	OR01	0.996	0.987	1.006	0.437
				OR10	1.073	1.000	1.151	0.050
			Lag 3	OR01	0.992	0.982	1.002	0.118
				OR10	1.070	0.997	1.148	0.059
		Lag 2	Lag 0	OR01	1.003	0.995	1.011	0.516
				OR10	1.019	0.951	1.092	0.599
			Lag 1	OR01	1.000	0.992	1.009	0.938
				OR10	1.024	0.955	1.097	0.509
			Lag 2	OR01	0.997	0.988	1.007	0.554
				OR10	1.032	0.963	1.106	0.378
			Lag 3	OR01	0.991	0.981	1.001	0.086
				OR10	1.017	0.949	1.090	0.637
		Lag 3	Lag 0	OR01	1.003	0.994	1.011	0.533
				OR10	0.956	0.890	1.026	0.208
			Lag 1	OR01	1.001	0.992	1.010	0.848
				OR10	0.967	0.901	1.037	0.348
			Lag 2	OR01	0.997	0.988	1.007	0.590
				OR10	0.973	0.907	1.044	0.447
			Lag 3	OR01	0.991	0.981	1.001	0.088
				OR10	0.959	0.894	1.030	0.252
	Renal	Lag 0	Lag 0	OR01	1.007	0.986	1.029	0.520
				OR10	1.053	0.947	1.170	0.343
			Lag 1	OR01	0.986	0.961	1.011	0.260
				OR10	1.068	0.961	1.186	0.225
			Lag 2	OR01	0.999	0.982	1.017	0.948
				OR10	1.062	0.956	1.180	0.261
			Lag 3	OR01	0.990	0.969	1.012	0.366
				OR10	1.047	0.942	1.163	0.396
		Lag 1	Lag 0	OR01	1.005	0.983	1.028	0.642
				OR10	1.095	0.986	1.216	0.088
			Lag 1	OR01	0.985	0.959	1.010	0.236
				OR10	1.112	1.002	1.234	**0.046**
			Lag 2	OR01	1.000	0.982	1.018	0.981
				OR10	1.112	1.002	1.234	**0.046**
			Lag 3	OR01	0.991	0.971	1.012	0.414
				OR10	1.098	0.989	1.219	0.078
		Lag 2	Lag 0	OR01	1.009	0.988	1.030	0.411
				OR10	1.045	0.943	1.160	0.400
			Lag 1	OR01	0.983	0.958	1.009	0.207
				OR10	1.047	0.944	1.161	0.388

			Lag 2	OR01	0.998	0.980	1.017	0.861
				OR10	1.044	0.941	1.158	0.416
			Lag 3	OR01	0.988	0.966	1.010	0.296
				OR10	1.033	0.931	1.146	0.538
		Lag 3	Lag 0	OR01	1.010	0.989	1.031	0.365
				OR10	0.978	0.882	1.085	0.675
			Lag 1	OR01	0.986	0.962	1.011	0.258
				OR10	0.982	0.885	1.088	0.724
			Lag 2	OR01	0.999	0.981	1.018	0.936
				OR10	0.978	0.882	1.084	0.669
			Lag 3	OR01	0.992	0.970	1.013	0.450
				OR10	0.976	0.880	1.082	0.643
	Respiratory	Lag 0	Lag 0	OR01	0.968	0.943	0.995	**0.020**
				OR10	0.974	0.882	1.075	0.598
			Lag 1	OR01	1.000	0.986	1.015	0.950
				OR10	0.976	0.884	1.076	0.623
			Lag 2	OR01	1.012	0.998	1.027	0.102
				OR10	0.983	0.891	1.084	0.732
			Lag 3	OR01	1.000	0.984	1.016	0.988
				OR10	0.967	0.877	1.067	0.504
		Lag 1	Lag 0	OR01	0.965	0.939	0.992	**0.012**
				OR10	1.012	0.919	1.115	0.803
			Lag 1	OR01	0.999	0.985	1.014	0.917
				OR10	1.011	0.918	1.114	0.823
			Lag 2	OR01	1.012	0.997	1.027	0.118
				OR10	1.023	0.929	1.127	0.644
			Lag 3	OR01	0.999	0.984	1.015	0.922
				OR10	1.006	0.912	1.108	0.911
		Lag 2	Lag 0	OR01	0.960	0.933	0.988	**0.006**
				OR10	1.039	0.945	1.143	0.428
			Lag 1	OR01	0.996	0.981	1.011	0.611
				OR10	1.036	0.943	1.140	0.459
			Lag 2	OR01	1.011	0.996	1.026	0.140
				OR10	1.052	0.956	1.158	0.295
			Lag 3	OR01	0.999	0.983	1.015	0.915
				OR10	1.041	0.946	1.145	0.409
		Lag 3	Lag 0	OR01	0.969	0.944	0.995	**0.022**
				OR10	1.027	0.935	1.128	0.574
			Lag 1	OR01	1.000	0.985	1.014	0.955
				OR10	1.021	0.930	1.121	0.655
			Lag 2	OR01	1.012	0.996	1.027	0.135
				OR10	1.026	0.934	1.126	0.591

			Lag 3	OR01	0.999	0.983	1.015	0.856
				OR10	1.013	0.922	1.113	0.787
Hispanic	All-natural	Lag 0	Lag 0	OR01	1.000	1.000	1.001	0.235
				OR10	1.029	1.023	1.034	**0.000**
			Lag 1	OR01	1.000	0.999	1.000	0.329
				OR10	1.030	1.024	1.035	**0.000**
			Lag 2	OR01	0.999	0.998	1.000	**0.012**
				OR10	1.030	1.025	1.036	**0.000**
			Lag 3	OR01	0.999	0.998	1.000	**0.028**
				OR10	1.029	1.024	1.035	**0.000**
		Lag 1	Lag 0	OR01	1.001	1.000	1.001	0.205
				OR10	1.027	1.022	1.032	**0.000**
			Lag 1	OR01	1.000	0.999	1.000	0.437
				OR10	1.028	1.023	1.034	**0.000**
			Lag 2	OR01	0.999	0.998	1.000	**0.019**
				OR10	1.029	1.024	1.034	**0.000**
			Lag 3	OR01	0.999	0.998	1.000	**0.023**
				OR10	1.027	1.022	1.033	**0.000**
		Lag 2	Lag 0	OR01	1.001	1.000	1.001	0.139
				OR10	1.021	1.016	1.027	**0.000**
			Lag 1	OR01	1.000	0.999	1.000	0.469
				OR10	1.022	1.017	1.028	**0.000**
			Lag 2	OR01	0.999	0.998	1.000	**0.045**
				OR10	1.024	1.019	1.029	**0.000**
			Lag 3	OR01	0.999	0.998	1.000	**0.022**
				OR10	1.022	1.016	1.027	**0.000**
		Lag 3	Lag 0	OR01	1.001	1.000	1.001	0.146
				OR10	1.014	1.009	1.019	**0.000**
			Lag 1	OR01	1.000	0.999	1.000	0.336
				OR10	1.014	1.009	1.020	**0.000**
			Lag 2	OR01	0.999	0.998	1.000	0.066
				OR10	1.017	1.011	1.022	**0.000**
			Lag 3	OR01	0.999	0.998	1.000	**0.042**
				OR10	1.015	1.010	1.020	**0.000**
	Cardiovascular	Lag 0	Lag 0	OR01	1.006	0.987	1.024	0.545
				OR10	0.979	0.857	1.118	0.753
			Lag 1	OR01	1.016	0.996	1.036	0.112
				OR10	0.976	0.855	1.113	0.714
			Lag 2	OR01	1.006	0.981	1.032	0.626
				OR10	1.004	0.880	1.145	0.954
			Lag 3	OR01	1.006	0.987	1.025	0.571
				OR10	0.993	0.871	1.133	0.917

		Lag 1	Lag 0	OR01	1.006	0.988	1.024	0.534
				OR10	1.003	0.882	1.142	0.959
			Lag 1	OR01	1.016	0.996	1.036	0.109
				OR10	0.999	0.878	1.137	0.992
			Lag 2	OR01	1.006	0.980	1.032	0.652
				OR10	1.022	0.898	1.162	0.741
			Lag 3	OR01	1.006	0.987	1.025	0.536
				OR10	1.019	0.896	1.159	0.775
		Lag 2	Lag 0	OR01	1.005	0.987	1.024	0.564
				OR10	1.001	0.883	1.134	0.993
			Lag 1	OR01	1.015	0.996	1.035	0.129
				OR10	0.995	0.877	1.127	0.933
			Lag 2	OR01	1.006	0.980	1.033	0.652
				OR10	1.019	0.899	1.155	0.771
			Lag 3	OR01	1.003	0.984	1.023	0.760
				OR10	0.997	0.879	1.130	0.958
		Lag 3	Lag 0	OR01	1.005	0.987	1.024	0.600
				OR10	0.986	0.869	1.119	0.827
			Lag 1	OR01	1.016	0.997	1.036	0.104
				OR10	0.992	0.875	1.125	0.903
			Lag 2	OR01	1.008	0.983	1.035	0.523
				OR10	1.019	0.899	1.156	0.766
			Lag 3	OR01	1.003	0.983	1.023	0.776
				OR10	0.986	0.869	1.119	0.825
	Cerebrovascular	Lag 0	Lag 0	OR01	1.002	0.986	1.019	0.785
				OR10	1.031	0.929	1.144	0.569
			Lag 1	OR01	1.009	0.993	1.025	0.286
				OR10	1.027	0.926	1.140	0.612
			Lag 2	OR01	1.001	0.984	1.017	0.941
				OR10	1.015	0.914	1.126	0.785
			Lag 3	OR01	0.995	0.979	1.012	0.575
				OR10	1.010	0.910	1.121	0.854
		Lag 1	Lag 0	OR01	1.001	0.984	1.018	0.938
				OR10	1.022	0.921	1.133	0.685
			Lag 1	OR01	1.009	0.993	1.025	0.270
				OR10	1.033	0.932	1.146	0.534
			Lag 2	OR01	1.002	0.985	1.018	0.841
				OR10	1.027	0.926	1.138	0.618
			Lag 3	OR01	0.996	0.980	1.013	0.645
				OR10	1.018	0.919	1.128	0.729
		Lag 2	Lag 0	OR01	1.000	0.983	1.017	0.989
				OR10	0.977	0.881	1.084	0.666

			Lag 1	OR01	1.009	0.993	1.025	0.289
				OR10	0.989	0.892	1.096	0.826
			Lag 2	OR01	1.003	0.987	1.019	0.730
				OR10	0.996	0.898	1.104	0.934
			Lag 3	OR01	0.995	0.979	1.012	0.590
				OR10	0.976	0.880	1.083	0.647
		Lag 3	Lag 0	OR01	1.000	0.983	1.017	0.992
				OR10	0.989	0.894	1.095	0.834
			Lag 1	OR01	1.010	0.995	1.026	0.200
				OR10	1.012	0.914	1.119	0.821
			Lag 2	OR01	1.001	0.985	1.018	0.869
				OR10	0.995	0.900	1.101	0.926
			Lag 3	OR01	0.995	0.978	1.012	0.539
				OR10	0.984	0.888	1.089	0.749
	Renal	Lag 0	Lag 0	OR01	0.906	0.853	0.964	**0.002**
				OR10	1.093	0.941	1.269	0.244
			Lag 1	OR01	0.978	0.939	1.019	0.284
				OR10	1.107	0.954	1.286	0.180
			Lag 2	OR01	1.000	0.974	1.026	0.999
				OR10	1.094	0.943	1.270	0.236
			Lag 3	OR01	0.978	0.942	1.014	0.223
				OR10	1.077	0.928	1.250	0.327
		Lag 1	Lag 0	OR01	0.927	0.877	0.981	**0.008**
				OR10	1.033	0.889	1.201	0.668
			Lag 1	OR01	0.977	0.938	1.018	0.265
				OR10	1.017	0.875	1.182	0.824
			Lag 2	OR01	1.002	0.977	1.028	0.895
				OR10	1.024	0.881	1.190	0.759
			Lag 3	OR01	0.980	0.945	1.015	0.258
				OR10	1.001	0.862	1.163	0.991
		Lag 2	Lag 0	OR01	0.918	0.867	0.972	**0.004**
				OR10	1.121	0.968	1.299	0.126
			Lag 1	OR01	0.977	0.938	1.018	0.277
				OR10	1.123	0.970	1.301	0.119
			Lag 2	OR01	1.000	0.975	1.027	0.981
				OR10	1.121	0.966	1.300	0.133
			Lag 3	OR01	0.976	0.940	1.014	0.218
				OR10	1.103	0.952	1.279	0.192
		Lag 3	Lag 0	OR01	0.917	0.865	0.973	**0.004**
				OR10	1.065	0.922	1.230	0.392
			Lag 1	OR01	0.974	0.935	1.016	0.223
				OR10	1.059	0.918	1.223	0.433

			Lag 2	OR01	0.993	0.964	1.022	0.630
				OR10	1.033	0.894	1.194	0.661
			Lag 3	OR01	0.976	0.939	1.014	0.206
				OR10	1.047	0.905	1.211	0.536
	Respiratory	Lag 0	Lag 0	OR01	0.980	0.947	1.015	0.267
				OR10	1.144	0.987	1.327	0.074
			Lag 1	OR01	0.994	0.968	1.021	0.669
				OR10	1.150	0.993	1.331	0.063
			Lag 2	OR01	1.005	0.974	1.038	0.737
				OR10	1.139	0.983	1.319	0.082
			Lag 3	OR01	0.971	0.936	1.007	0.113
				OR10	1.155	0.998	1.336	0.053
		Lag 1	Lag 0	OR01	0.977	0.942	1.013	0.205
				OR10	1.081	0.937	1.248	0.287
			Lag 1	OR01	0.994	0.968	1.021	0.648
				OR10	1.097	0.951	1.266	0.205
			Lag 2	OR01	1.006	0.974	1.038	0.715
				OR10	1.091	0.945	1.260	0.233
			Lag 3	OR01	0.967	0.932	1.004	0.083
				OR10	1.093	0.947	1.262	0.222
		Lag 2	Lag 0	OR01	0.971	0.934	1.009	0.131
				OR10	1.037	0.898	1.198	0.617
			Lag 1	OR01	0.995	0.968	1.022	0.694
				OR10	1.068	0.926	1.232	0.367
			Lag 2	OR01	1.006	0.974	1.038	0.727
				OR10	1.059	0.917	1.223	0.431
			Lag 3	OR01	0.962	0.924	1.001	0.057
				OR10	1.053	0.912	1.216	0.479
		Lag 3	Lag 0	OR01	0.978	0.944	1.013	0.215
				OR10	0.912	0.790	1.051	0.204
			Lag 1	OR01	0.999	0.974	1.024	0.914
				OR10	0.945	0.821	1.089	0.437
			Lag 2	OR01	1.013	0.982	1.044	0.429
				OR10	0.949	0.824	1.094	0.472
			Lag 3	OR01	0.961	0.923	1.000	0.050
				OR10	0.910	0.789	1.049	0.193
Higher Education (< 50% high schoool education or less)	All-natural	Lag 0	Lag 0	OR01	1.000	0.999	1.000	0.544
				OR10	1.023	1.020	1.027	**0.000**
			Lag 1	OR01	1.000	0.999	1.000	0.241
				OR10	1.024	1.020	1.027	**0.000**
			Lag 2	OR01	0.999	0.999	1.000	**0.000**
				OR10	1.025	1.021	1.028	**0.000**

			Lag 3	OR01	0.999	0.999	0.999	**0.000**
				OR10	1.023	1.020	1.027	**0.000**
		Lag 1	Lag 0	OR01	1.000	0.999	1.000	0.406
				OR10	1.023	1.020	1.027	**0.000**
			Lag 1	OR01	1.000	0.999	1.000	0.369
				OR10	1.024	1.021	1.028	**0.000**
			Lag 2	OR01	0.999	0.999	1.000	**0.000**
				OR10	1.025	1.021	1.028	**0.000**
			Lag 3	OR01	0.999	0.999	0.999	**0.000**
				OR10	1.024	1.020	1.027	**0.000**
		Lag 2	Lag 0	OR01	1.000	0.999	1.000	0.535
				OR10	1.018	1.015	1.021	**0.000**
			Lag 1	OR01	1.000	0.999	1.000	0.370
				OR10	1.019	1.016	1.023	**0.000**
			Lag 2	OR01	0.999	0.999	1.000	**0.001**
				OR10	1.020	1.017	1.024	**0.000**
			Lag 3	OR01	0.999	0.999	0.999	**0.000**
				OR10	1.019	1.016	1.023	**0.000**
		Lag 3	Lag 0	OR01	1.000	0.999	1.000	0.533
				OR10	1.014	1.011	1.018	**0.000**
			Lag 1	OR01	1.000	0.999	1.000	0.257
				OR10	1.015	1.012	1.018	**0.000**
			Lag 2	OR01	0.999	0.999	1.000	**0.001**
				OR10	1.016	1.013	1.020	**0.000**
			Lag 3	OR01	0.999	0.999	0.999	**0.000**
				OR10	1.016	1.012	1.019	**0.000**
	Cardiovascular	Lag 0	Lag 0	OR01	1.000	0.990	1.009	0.920
				OR10	0.991	0.921	1.066	0.805
			Lag 1	OR01	1.004	0.995	1.013	0.417
				OR10	0.993	0.923	1.068	0.848
			Lag 2	OR01	0.999	0.990	1.008	0.839
				OR10	0.994	0.923	1.069	0.866
			Lag 3	OR01	0.998	0.989	1.008	0.695
				OR10	0.999	0.929	1.075	0.981
		Lag 1	Lag 0	OR01	0.999	0.990	1.009	0.858
				OR10	1.037	0.966	1.114	0.316
			Lag 1	OR01	1.004	0.995	1.013	0.396
				OR10	1.043	0.971	1.120	0.251
			Lag 2	OR01	0.998	0.989	1.007	0.710
				OR10	1.035	0.963	1.112	0.349
			Lag 3	OR01	0.998	0.989	1.007	0.680
				OR10	1.046	0.974	1.124	0.216

		Lag 2	Lag 0	OR01	0.999	0.990	1.008	0.835
				OR10	1.032	0.962	1.107	0.384
			Lag 1	OR01	1.005	0.996	1.014	0.314
				OR10	1.043	0.972	1.119	0.240
			Lag 2	OR01	0.999	0.990	1.008	0.838
				OR10	1.039	0.969	1.115	0.283
			Lag 3	OR01	0.998	0.989	1.008	0.702
				OR10	1.043	0.972	1.118	0.244
		Lag 3	Lag 0	OR01	0.999	0.990	1.009	0.845
				OR10	0.997	0.929	1.070	0.925
			Lag 1	OR01	1.004	0.995	1.013	0.382
				OR10	1.006	0.938	1.079	0.870
			Lag 2	OR01	0.999	0.990	1.008	0.849
				OR10	1.006	0.937	1.079	0.878
			Lag 3	OR01	0.997	0.988	1.007	0.598
				OR10	1.004	0.936	1.078	0.908
	Cerebrovascular	Lag 0	Lag 0	OR01	1.003	0.997	1.010	0.322
				OR10	1.029	0.970	1.092	0.342
			Lag 1	OR01	1.002	0.995	1.009	0.579
				OR10	1.034	0.975	1.097	0.268
			Lag 2	OR01	0.997	0.989	1.004	0.378
				OR10	1.027	0.968	1.090	0.377
			Lag 3	OR01	0.994	0.986	1.002	0.152
				OR10	1.023	0.964	1.086	0.445
		Lag 1	Lag 0	OR01	1.003	0.996	1.010	0.361
				OR10	1.034	0.975	1.098	0.262
			Lag 1	OR01	1.001	0.994	1.009	0.700
				OR10	1.037	0.978	1.101	0.226
			Lag 2	OR01	0.997	0.990	1.004	0.433
				OR10	1.040	0.980	1.103	0.200
			Lag 3	OR01	0.995	0.987	1.003	0.211
				OR10	1.038	0.978	1.102	0.215
		Lag 2	Lag 0	OR01	1.003	0.996	1.010	0.353
				OR10	1.061	1.001	1.125	**0.046**
			Lag 1	OR01	1.001	0.994	1.008	0.782
				OR10	1.064	1.004	1.127	**0.037**
			Lag 2	OR01	0.997	0.990	1.005	0.453
				OR10	1.069	1.009	1.133	**0.025**
			Lag 3	OR01	0.995	0.987	1.003	0.188
				OR10	1.065	1.004	1.128	**0.035**
		Lag 3	Lag 0	OR01	1.003	0.996	1.010	0.357
				OR10	1.009	0.951	1.069	0.776

			Lag 1	OR01	1.001	0.994	1.009	0.683
				OR10	1.013	0.956	1.074	0.661
			Lag 2	OR01	0.997	0.989	1.004	0.372
				OR10	1.013	0.956	1.074	0.666
			Lag 3	OR01	0.994	0.986	1.002	0.134
				OR10	1.008	0.951	1.069	0.784
	Renal	Lag 0	Lag 0	OR01	1.001	0.982	1.021	0.923
				OR10	1.111	1.010	1.223	**0.031**
			Lag 1	OR01	0.980	0.956	1.005	0.117
				OR10	1.119	1.017	1.232	**0.022**
			Lag 2	OR01	1.003	0.987	1.019	0.706
				OR10	1.117	1.015	1.229	**0.024**
			Lag 3	OR01	1.002	0.986	1.018	0.847
				OR10	1.113	1.011	1.224	**0.029**
		Lag 1	Lag 0	OR01	1.000	0.980	1.020	0.999
				OR10	1.123	1.023	1.234	**0.015**
			Lag 1	OR01	0.978	0.953	1.004	0.094
				OR10	1.130	1.028	1.241	**0.011**
			Lag 2	OR01	1.005	0.989	1.020	0.550
				OR10	1.145	1.043	1.258	**0.005**
			Lag 3	OR01	1.001	0.985	1.018	0.874
				OR10	1.124	1.024	1.235	**0.014**
		Lag 2	Lag 0	OR01	1.004	0.986	1.023	0.658
				OR10	1.078	0.982	1.184	0.113
			Lag 1	OR01	0.980	0.955	1.005	0.110
				OR10	1.075	0.979	1.181	0.129
			Lag 2	OR01	1.004	0.988	1.020	0.611
				OR10	1.084	0.987	1.190	0.093
			Lag 3	OR01	1.001	0.985	1.018	0.874
				OR10	1.074	0.978	1.180	0.136
		Lag 3	Lag 0	OR01	1.007	0.988	1.025	0.480
				OR10	1.060	0.966	1.163	0.218
			Lag 1	OR01	0.983	0.960	1.007	0.161
				OR10	1.055	0.961	1.158	0.258
			Lag 2	OR01	1.004	0.989	1.020	0.593
				OR10	1.055	0.962	1.157	0.258
			Lag 3	OR01	1.002	0.986	1.019	0.766
				OR10	1.051	0.958	1.154	0.290
	Respiratory	Lag 0	Lag 0	OR01	0.988	0.973	1.004	0.140
				OR10	0.978	0.895	1.069	0.628
			Lag 1	OR01	1.001	0.990	1.011	0.913
				OR10	0.976	0.894	1.067	0.595

			Lag 2	OR01	1.006	0.994	1.017	0.336
				OR10	0.989	0.905	1.080	0.803
			Lag 3	OR01	0.997	0.984	1.010	0.609
				OR10	0.963	0.881	1.052	0.405
		Lag 1	Lag 0	OR01	0.989	0.974	1.005	0.178
				OR10	1.017	0.932	1.110	0.701
			Lag 1	OR01	1.000	0.990	1.011	0.933
				OR10	1.008	0.923	1.099	0.865
			Lag 2	OR01	1.005	0.994	1.017	0.356
				OR10	1.018	0.933	1.110	0.689
			Lag 3	OR01	0.997	0.984	1.010	0.678
				OR10	0.996	0.913	1.088	0.934
		Lag 2	Lag 0	OR01	0.987	0.972	1.003	0.123
				OR10	1.034	0.948	1.128	0.448
			Lag 1	OR01	0.999	0.988	1.010	0.830
				OR10	1.027	0.942	1.120	0.545
			Lag 2	OR01	1.006	0.994	1.017	0.341
				OR10	1.047	0.960	1.141	0.304
			Lag 3	OR01	0.997	0.984	1.010	0.674
				OR10	1.027	0.942	1.121	0.546
		Lag 3	Lag 0	OR01	0.989	0.974	1.005	0.164
				OR10	1.063	0.977	1.157	0.157
			Lag 1	OR01	1.001	0.991	1.011	0.832
				OR10	1.062	0.976	1.156	0.160
			Lag 2	OR01	1.006	0.994	1.017	0.344
				OR10	1.069	0.982	1.163	0.122
			Lag 3	OR01	0.998	0.985	1.011	0.738
				OR10	1.056	0.970	1.150	0.205
Lower Education (> 50% high schoool education or less)	All-natural	Lag 0	Lag 0	OR01	1.001	1.000	1.001	0.135
				OR10	1.025	1.020	1.030	**0.000**
			Lag 1	OR01	1.000	0.999	1.001	0.816
				OR10	1.026	1.020	1.031	**0.000**
			Lag 2	OR01	0.999	0.999	1.000	0.166
				OR10	1.026	1.021	1.031	**0.000**
			Lag 3	OR01	1.000	0.999	1.000	0.237
				OR10	1.026	1.021	1.031	**0.000**
		Lag 1	Lag 0	OR01	1.000	1.000	1.001	0.274
				OR10	1.022	1.017	1.027	**0.000**
			Lag 1	OR01	1.000	0.999	1.001	0.835
				OR10	1.024	1.019	1.029	**0.000**
			Lag 2	OR01	1.000	0.999	1.000	0.191
				OR10	1.024	1.019	1.029	**0.000**

			Lag 3	OR01	0.999	0.999	1.000	0.174
				OR10	1.024	1.019	1.029	**0.000**
		Lag 2	Lag 0	OR01	1.000	1.000	1.001	0.218
				OR10	1.019	1.014	1.024	**0.000**
			Lag 1	OR01	1.000	0.999	1.001	0.651
				OR10	1.020	1.016	1.025	**0.000**
			Lag 2	OR01	1.000	0.999	1.000	0.395
				OR10	1.022	1.017	1.027	**0.000**
			Lag 3	OR01	1.000	0.999	1.000	0.196
				OR10	1.020	1.015	1.025	**0.000**
		Lag 3	Lag 0	OR01	1.000	1.000	1.001	0.186
				OR10	1.012	1.007	1.017	**0.000**
			Lag 1	OR01	1.000	0.999	1.001	0.816
				OR10	1.012	1.008	1.017	**0.000**
			Lag 2	OR01	1.000	0.999	1.000	0.466
				OR10	1.015	1.010	1.020	**0.000**
			Lag 3	OR01	0.999	0.999	1.000	0.154
				OR10	1.013	1.008	1.018	**0.000**
	Cardiovascular	Lag 0	Lag 0	OR01	1.011	0.991	1.032	0.271
				OR10	1.105	0.995	1.227	0.062
			Lag 1	OR01	1.014	0.994	1.035	0.169
				OR10	1.099	0.989	1.220	0.078
			Lag 2	OR01	1.003	0.982	1.025	0.761
				OR10	1.106	0.996	1.228	0.058
			Lag 3	OR01	1.001	0.982	1.021	0.886
				OR10	1.120	1.009	1.242	**0.033**
		Lag 1	Lag 0	OR01	1.011	0.991	1.031	0.302
				OR10	1.100	0.992	1.219	0.072
			Lag 1	OR01	1.014	0.994	1.035	0.172
				OR10	1.095	0.988	1.215	0.085
			Lag 2	OR01	1.002	0.981	1.024	0.821
				OR10	1.101	0.993	1.221	0.067
			Lag 3	OR01	0.999	0.979	1.019	0.910
				OR10	1.106	0.998	1.225	0.055
		Lag 2	Lag 0	OR01	1.007	0.987	1.028	0.475
				OR10	1.021	0.921	1.132	0.692
			Lag 1	OR01	1.017	0.997	1.038	0.091
				OR10	1.045	0.943	1.159	0.400
			Lag 2	OR01	1.006	0.985	1.027	0.581
				OR10	1.050	0.947	1.164	0.355
			Lag 3	OR01	1.001	0.981	1.020	0.951
				OR10	1.048	0.946	1.162	0.367

		Lag 3	Lag 0	OR01	1.009	0.989	1.029	0.399
				OR10	1.060	0.956	1.174	0.269
			Lag 1	OR01	1.017	0.996	1.038	0.113
				OR10	1.079	0.975	1.195	0.140
			Lag 2	OR01	1.010	0.989	1.031	0.365
				OR10	1.106	0.999	1.225	0.052
			Lag 3	OR01	1.000	0.981	1.020	0.992
				OR10	1.086	0.981	1.203	0.112
	Cerebrovascular	Lag 0	Lag 0	OR01	1.004	0.991	1.017	0.541
				OR10	1.014	0.926	1.111	0.761
			Lag 1	OR01	0.998	0.985	1.011	0.753
				OR10	0.999	0.911	1.094	0.977
			Lag 2	OR01	1.003	0.991	1.016	0.597
				OR10	1.004	0.917	1.100	0.923
			Lag 3	OR01	0.999	0.986	1.013	0.909
				OR10	1.006	0.919	1.101	0.902
		Lag 1	Lag 0	OR01	1.003	0.990	1.016	0.611
				OR10	1.028	0.940	1.124	0.550
			Lag 1	OR01	0.998	0.986	1.011	0.811
				OR10	1.022	0.935	1.118	0.631
			Lag 2	OR01	1.003	0.990	1.016	0.641
				OR10	1.020	0.933	1.116	0.662
			Lag 3	OR01	0.998	0.985	1.012	0.819
				OR10	1.019	0.932	1.114	0.678
		Lag 2	Lag 0	OR01	1.004	0.991	1.017	0.540
				OR10	0.997	0.913	1.089	0.945
			Lag 1	OR01	0.998	0.985	1.011	0.785
				OR10	0.986	0.902	1.077	0.753
			Lag 2	OR01	1.004	0.991	1.017	0.548
				OR10	0.992	0.907	1.084	0.854
			Lag 3	OR01	0.998	0.984	1.012	0.742
				OR10	0.979	0.896	1.071	0.649
		Lag 3	Lag 0	OR01	1.002	0.989	1.015	0.735
				OR10	1.031	0.944	1.125	0.497
			Lag 1	OR01	0.997	0.984	1.010	0.643
				OR10	1.027	0.941	1.121	0.550
			Lag 2	OR01	1.002	0.989	1.015	0.778
				OR10	1.024	0.938	1.119	0.593
			Lag 3	OR01	0.998	0.984	1.012	0.796
				OR10	1.030	0.943	1.125	0.509
	Renal	Lag 0	Lag 0	OR01	0.961	0.919	1.005	0.081
				OR10	1.038	0.905	1.190	0.599

			Lag 1	OR01	1.006	0.984	1.029	0.607
				OR10	1.049	0.914	1.204	0.494
			Lag 2	OR01	1.015	0.992	1.039	0.199
				OR10	1.023	0.892	1.173	0.747
			Lag 3	OR01	0.987	0.954	1.021	0.455
				OR10	1.026	0.895	1.176	0.712
		Lag 1	Lag 0	OR01	0.958	0.915	1.002	0.062
				OR10	1.085	0.950	1.238	0.227
			Lag 1	OR01	1.007	0.985	1.030	0.544
				OR10	1.108	0.971	1.264	0.128
			Lag 2	OR01	1.017	0.994	1.040	0.157
				OR10	1.082	0.948	1.234	0.245
			Lag 3	OR01	0.984	0.949	1.019	0.366
				OR10	1.068	0.936	1.219	0.328
		Lag 2	Lag 0	OR01	0.965	0.926	1.007	0.103
				OR10	1.055	0.925	1.203	0.427
			Lag 1	OR01	1.006	0.983	1.029	0.609
				OR10	1.059	0.929	1.208	0.390
			Lag 2	OR01	1.015	0.992	1.038	0.208
				OR10	1.031	0.902	1.177	0.654
			Lag 3	OR01	0.976	0.938	1.014	0.214
				OR10	1.018	0.891	1.162	0.796
		Lag 3	Lag 0	OR01	0.967	0.929	1.008	0.112
				OR10	1.031	0.904	1.176	0.648
			Lag 1	OR01	1.004	0.981	1.027	0.754
				OR10	1.027	0.901	1.171	0.692
			Lag 2	OR01	1.014	0.991	1.038	0.227
				OR10	1.006	0.881	1.149	0.928
			Lag 3	OR01	0.980	0.944	1.018	0.305
				OR10	1.005	0.880	1.147	0.946
	Respiratory	Lag 0	Lag 0	OR01	1.008	0.990	1.026	0.366
				OR10	0.958	0.847	1.084	0.496
			Lag 1	OR01	1.003	0.986	1.021	0.720
				OR10	0.943	0.834	1.067	0.354
			Lag 2	OR01	1.002	0.979	1.027	0.845
				OR10	0.940	0.831	1.063	0.327
			Lag 3	OR01	1.009	0.992	1.028	0.299
				OR10	0.938	0.829	1.060	0.306
		Lag 1	Lag 0	OR01	1.008	0.990	1.026	0.403
				OR10	1.006	0.893	1.135	0.918
			Lag 1	OR01	1.002	0.985	1.021	0.789
				OR10	0.992	0.880	1.119	0.895

			Lag 2	OR01	1.005	0.981	1.029	0.684
				OR10	1.005	0.892	1.133	0.931
			Lag 3	OR01	1.009	0.991	1.027	0.322
				OR10	0.988	0.876	1.114	0.841
		Lag 2	Lag 0	OR01	1.004	0.985	1.023	0.690
				OR10	0.937	0.831	1.056	0.287
			Lag 1	OR01	1.003	0.985	1.022	0.717
				OR10	0.954	0.846	1.075	0.439
			Lag 2	OR01	1.004	0.980	1.028	0.766
				OR10	0.955	0.847	1.076	0.451
			Lag 3	OR01	1.011	0.993	1.029	0.220
				OR10	0.959	0.850	1.081	0.491
		Lag 3	Lag 0	OR01	1.008	0.990	1.026	0.399
				OR10	1.005	0.896	1.126	0.936
			Lag 1	OR01	0.999	0.977	1.020	0.902
				OR10	0.994	0.887	1.112	0.910
			Lag 2	OR01	1.006	0.983	1.030	0.600
				OR10	1.009	0.900	1.130	0.882
			Lag 3	OR01	1.010	0.993	1.028	0.259
				OR10	0.997	0.889	1.118	0.955
Majority Rural (> 50%)	All-natural	Lag 0	Lag 0	OR01	1.002	0.999	1.006	0.210
				OR10	1.007	0.990	1.023	0.441
			Lag 1	OR01	1.001	0.997	1.005	0.671
				OR10	1.012	0.995	1.029	0.154
			Lag 2	OR01	1.001	0.997	1.005	0.514
				OR10	1.010	0.994	1.027	0.236
			Lag 3	OR01	1.000	0.996	1.004	0.874
				OR10	1.008	0.992	1.025	0.328
		Lag 1	Lag 0	OR01	1.002	0.998	1.006	0.272
				OR10	1.008	0.991	1.024	0.353
			Lag 1	OR01	1.001	0.997	1.005	0.492
				OR10	1.013	0.997	1.030	0.110
			Lag 2	OR01	1.001	0.997	1.005	0.508
				OR10	1.013	0.996	1.029	0.135
			Lag 3	OR01	1.001	0.997	1.005	0.583
				OR10	1.013	0.997	1.030	0.115
		Lag 2	Lag 0	OR01	1.003	0.999	1.007	0.212
				OR10	0.996	0.980	1.012	0.613
			Lag 1	OR01	1.002	0.998	1.006	0.444
				OR10	1.000	0.984	1.017	0.963
			Lag 2	OR01	1.002	0.998	1.006	0.367
				OR10	1.000	0.984	1.017	0.997

			Lag 3	OR01	1.001	0.997	1.005	0.561
				OR10	1.001	0.985	1.017	0.906
		Lag 3	Lag 0	OR01	1.002	0.998	1.006	0.416
				OR10	1.003	0.987	1.020	0.683
			Lag 1	OR01	0.999	0.995	1.004	0.733
				OR10	1.004	0.988	1.021	0.601
			Lag 2	OR01	1.001	0.997	1.005	0.640
				OR10	1.006	0.990	1.022	0.471
			Lag 3	OR01	1.001	0.997	1.005	0.736
				OR10	1.006	0.990	1.023	0.435
	Cardiovascular	Lag 0	Lag 0	OR01	1.101	0.983	1.233	0.096
				OR10	1.056	0.756	1.477	0.748
			Lag 1	OR01	1.076	0.991	1.167	0.080
				OR10	1.148	0.820	1.605	0.422
			Lag 2	OR01	1.047	0.918	1.193	0.495
				OR10	1.113	0.796	1.555	0.532
			Lag 3	OR01	1.050	0.928	1.187	0.438
				OR10	1.082	0.776	1.508	0.644
		Lag 1	Lag 0	OR01	1.092	0.978	1.219	0.118
				OR10	0.834	0.593	1.174	0.298
			Lag 1	OR01	1.082	0.994	1.178	0.069
				OR10	0.880	0.624	1.241	0.465
			Lag 2	OR01	1.034	0.905	1.181	0.625
				OR10	0.836	0.593	1.177	0.304
			Lag 3	OR01	1.040	0.919	1.177	0.533
				OR10	0.848	0.605	1.190	0.340
		Lag 2	Lag 0	OR01	1.102	0.982	1.236	0.098
				OR10	0.730	0.516	1.034	0.076
			Lag 1	OR01	1.064	0.982	1.153	0.131
				OR10	0.697	0.487	0.996	**0.048**
			Lag 2	OR01	1.009	0.877	1.162	0.897
				OR10	0.677	0.472	0.973	**0.035**
			Lag 3	OR01	1.051	0.929	1.189	0.429
				OR10	0.761	0.537	1.077	0.123
		Lag 3	Lag 0	OR01	1.101	0.982	1.234	0.099
				OR10	0.919	0.667	1.265	0.605
			Lag 1	OR01	1.089	0.976	1.215	0.129
				OR10	0.887	0.643	1.223	0.464
			Lag 2	OR01	1.047	0.915	1.199	0.504
				OR10	0.936	0.679	1.290	0.685
			Lag 3	OR01	0.958	0.808	1.136	0.622
				OR10	0.857	0.618	1.190	0.357

Cerebrovascular	Lag 0	Lag 0	OR01	0.958	0.841	1.091	0.518
			OR10	0.939	0.692	1.275	0.688
		Lag 1	OR01	0.950	0.847	1.066	0.380
			OR10	0.940	0.693	1.275	0.690
		Lag 2	OR01	0.984	0.918	1.055	0.654
			OR10	0.990	0.733	1.338	0.949
		Lag 3	OR01	1.082	0.959	1.219	0.201
			OR10	0.950	0.702	1.286	0.740
	Lag 1	Lag 0	OR01	0.953	0.829	1.095	0.498
			OR10	1.044	0.790	1.380	0.763
		Lag 1	OR01	0.935	0.825	1.059	0.288
			OR10	1.019	0.771	1.347	0.895
		Lag 2	OR01	0.989	0.928	1.054	0.740
			OR10	1.105	0.840	1.454	0.476
		Lag 3	OR01	1.106	0.984	1.241	0.090
			OR10	1.086	0.826	1.429	0.553
	Lag 2	Lag 0	OR01	0.977	0.860	1.109	0.716
			OR10	1.102	0.835	1.455	0.492
		Lag 1	OR01	0.968	0.865	1.082	0.566
			OR10	1.103	0.835	1.456	0.492
		Lag 2	OR01	0.982	0.911	1.059	0.643
			OR10	1.094	0.828	1.445	0.529
		Lag 3	OR01	1.108	0.986	1.244	0.084
			OR10	1.121	0.850	1.479	0.420
	Lag 3	Lag 0	OR01	0.953	0.829	1.095	0.495
			OR10	0.985	0.733	1.323	0.918
		Lag 1	OR01	0.938	0.829	1.061	0.307
			OR10	0.970	0.721	1.306	0.842
		Lag 2	OR01	0.967	0.878	1.066	0.501
			OR10	0.959	0.709	1.298	0.787
		Lag 3	OR01	1.091	0.971	1.226	0.142
			OR10	1.001	0.746	1.344	0.995
Renal	Lag 0	Lag 0	OR01	0.818	0.540	1.237	0.340
			OR10	0.971	0.601	1.569	0.904
		Lag 1	OR01	0.523	0.259	1.056	0.071
			OR10	0.937	0.575	1.527	0.795
		Lag 2	OR01	1.104	0.916	1.331	0.298
			OR10	0.909	0.558	1.482	0.702
		Lag 3	OR01	0.871	0.646	1.176	0.367
			OR10	0.967	0.599	1.560	0.890
	Lag 1	Lag 0	OR01	0.835	0.574	1.215	0.346
			OR10	1.404	0.898	2.196	0.137

			Lag 1	OR01	0.771	0.532	1.118	0.170
				OR10	1.519	0.968	2.384	0.069
			Lag 2	OR01	1.158	0.995	1.348	0.057
				OR10	1.715	1.085	2.711	**0.021**
			Lag 3	OR01	0.854	0.614	1.186	0.346
				OR10	1.428	0.912	2.235	0.119
		Lag 2	Lag 0	OR01	0.856	0.598	1.224	0.395
				OR10	1.418	0.933	2.155	0.102
			Lag 1	OR01	0.768	0.530	1.114	0.164
				OR10	1.513	0.995	2.303	0.053
			Lag 2	OR01	1.130	0.976	1.308	0.103
				OR10	1.421	0.934	2.160	0.100
			Lag 3	OR01	0.857	0.628	1.170	0.331
				OR10	1.436	0.944	2.186	0.091
		Lag 3	Lag 0	OR01	0.843	0.587	1.212	0.357
				OR10	1.308	0.846	2.023	0.227
			Lag 1	OR01	0.762	0.535	1.085	0.131
				OR10	1.372	0.888	2.119	0.154
			Lag 2	OR01	1.132	0.982	1.304	0.088
				OR10	1.346	0.869	2.083	0.183
			Lag 3	OR01	0.838	0.606	1.158	0.284
				OR10	1.313	0.845	2.039	0.226
	Respiratory	Lag 0	Lag 0	OR01	0.941	0.751	1.179	0.597
				OR10	1.057	0.722	1.546	0.777
			Lag 1	OR01	1.092	0.946	1.260	0.229
				OR10	0.984	0.671	1.443	0.935
			Lag 2	OR01	1.124	0.983	1.285	0.087
				OR10	1.134	0.777	1.656	0.514
			Lag 3	OR01	0.979	0.881	1.089	0.702
				OR10	1.007	0.690	1.468	0.972
		Lag 1	Lag 0	OR01	0.931	0.743	1.167	0.536
				OR10	1.231	0.832	1.822	0.299
			Lag 1	OR01	1.112	0.964	1.282	0.144
				OR10	1.200	0.807	1.785	0.368
			Lag 2	OR01	1.112	0.985	1.255	0.087
				OR10	1.327	0.895	1.968	0.159
			Lag 3	OR01	0.989	0.903	1.083	0.809
				OR10	1.189	0.805	1.757	0.385
		Lag 2	Lag 0	OR01	0.950	0.768	1.176	0.640
				OR10	0.873	0.591	1.289	0.494
			Lag 1	OR01	1.112	0.962	1.285	0.150
				OR10	0.797	0.536	1.184	0.261

			Lag 2	OR01	1.113	0.984	1.259	0.089
				OR10	0.873	0.588	1.297	0.503
			Lag 3	OR01	0.988	0.901	1.083	0.789
				OR10	0.794	0.535	1.179	0.253
		Lag 3	Lag 0	OR01	0.961	0.780	1.183	0.706
				OR10	1.219	0.866	1.717	0.256
			Lag 1	OR01	1.087	0.926	1.275	0.307
				OR10	1.044	0.734	1.484	0.813
			Lag 2	OR01	1.102	0.983	1.234	0.095
				OR10	1.175	0.830	1.664	0.363
			Lag 3	OR01	0.993	0.912	1.081	0.866
				OR10	1.128	0.800	1.591	0.491
Majority Urban	All-natural	Lag 0	Lag 0	OR01	1.000	1.000	1.000	0.936
				OR10	1.024	1.021	1.027	**0.000**
			Lag 1	OR01	1.000	0.999	1.000	0.318
				OR10	1.025	1.022	1.027	**0.000**
			Lag 2	OR01	0.999	0.999	1.000	**0.000**
				OR10	1.025	1.023	1.028	**0.000**
			Lag 3	OR01	0.999	0.999	0.999	**0.000**
				OR10	1.024	1.022	1.027	**0.000**
		Lag 1	Lag 0	OR01	1.000	1.000	1.000	0.764
				OR10	1.023	1.020	1.026	**0.000**
			Lag 1	OR01	1.000	0.999	1.000	0.428
				OR10	1.024	1.022	1.027	**0.000**
			Lag 2	OR01	0.999	0.999	1.000	**0.000**
				OR10	1.025	1.022	1.028	**0.000**
			Lag 3	OR01	0.999	0.999	0.999	**0.000**
				OR10	1.024	1.021	1.027	**0.000**
		Lag 2	Lag 0	OR01	1.000	1.000	1.000	0.947
				OR10	1.019	1.016	1.022	**0.000**
			Lag 1	OR01	1.000	1.000	1.000	0.500
				OR10	1.020	1.017	1.023	**0.000**
			Lag 2	OR01	0.999	0.999	1.000	**0.000**
				OR10	1.021	1.018	1.024	**0.000**
			Lag 3	OR01	0.999	0.999	1.000	**0.000**
				OR10	1.020	1.017	1.023	**0.000**
		Lag 3	Lag 0	OR01	1.000	1.000	1.000	0.998
				OR10	1.014	1.011	1.017	**0.000**
			Lag 1	OR01	1.000	0.999	1.000	0.258
				OR10	1.015	1.012	1.017	**0.000**
			Lag 2	OR01	0.999	0.999	1.000	**0.001**
				OR10	1.016	1.013	1.019	**0.000**

			Lag 3	OR01	0.999	0.999	0.999	**0.000**
				OR10	1.015	1.012	1.018	**0.000**
	Cardiovascular	Lag 0	Lag 0	OR01	1.001	0.993	1.010	0.795
				OR10	1.026	0.965	1.091	0.407
			Lag 1	OR01	1.005	0.997	1.013	0.259
				OR10	1.026	0.965	1.090	0.409
			Lag 2	OR01	1.000	0.991	1.008	0.932
				OR10	1.028	0.967	1.092	0.383
			Lag 3	OR01	0.999	0.990	1.007	0.737
				OR10	1.036	0.975	1.101	0.252
		Lag 1	Lag 0	OR01	1.001	0.992	1.009	0.872
				OR10	1.066	1.004	1.131	**0.037**
			Lag 1	OR01	1.005	0.997	1.013	0.251
				OR10	1.069	1.007	1.134	**0.029**
			Lag 2	OR01	0.999	0.991	1.007	0.797
				OR10	1.064	1.003	1.130	**0.041**
			Lag 3	OR01	0.998	0.990	1.007	0.651
				OR10	1.073	1.011	1.139	**0.020**
		Lag 2	Lag 0	OR01	1.000	0.992	1.009	0.995
				OR10	1.043	0.983	1.106	0.164
			Lag 1	OR01	1.006	0.998	1.014	0.147
				OR10	1.058	0.997	1.121	0.061
			Lag 2	OR01	1.000	0.992	1.008	0.970
				OR10	1.057	0.996	1.121	0.066
			Lag 3	OR01	0.998	0.990	1.007	0.720
				OR10	1.058	0.998	1.122	0.059
		Lag 3	Lag 0	OR01	1.000	0.992	1.009	0.943
				OR10	1.023	0.964	1.085	0.458
			Lag 1	OR01	1.006	0.998	1.014	0.169
				OR10	1.037	0.978	1.100	0.226
			Lag 2	OR01	1.001	0.993	1.009	0.871
				OR10	1.040	0.981	1.103	0.190
			Lag 3	OR01	0.998	0.990	1.007	0.669
				OR10	1.037	0.978	1.100	0.225
	Cerebrovascular	Lag 0	Lag 0	OR01	1.004	0.998	1.010	0.235
				OR10	1.025	0.975	1.078	0.330
			Lag 1	OR01	1.001	0.995	1.007	0.702
				OR10	1.026	0.975	1.079	0.325
			Lag 2	OR01	0.998	0.992	1.005	0.629
				OR10	1.022	0.971	1.074	0.408
			Lag 3	OR01	0.995	0.988	1.002	0.168
				OR10	1.019	0.969	1.072	0.453

		Lag 1	Lag 0	OR01	1.003	0.997	1.009	0.285
				OR10	1.030	0.980	1.083	0.242
			Lag 1	OR01	1.001	0.995	1.007	0.779
				OR10	1.032	0.981	1.085	0.226
			Lag 2	OR01	0.999	0.992	1.005	0.659
				OR10	1.032	0.981	1.085	0.224
			Lag 3	OR01	0.995	0.989	1.002	0.194
				OR10	1.030	0.980	1.084	0.243
		Lag 2	Lag 0	OR01	1.003	0.997	1.009	0.268
				OR10	1.037	0.987	1.090	0.145
			Lag 1	OR01	1.000	0.994	1.007	0.895
				OR10	1.036	0.986	1.088	0.162
			Lag 2	OR01	0.999	0.992	1.005	0.736
				OR10	1.042	0.991	1.094	0.107
			Lag 3	OR01	0.995	0.988	1.002	0.158
				OR10	1.035	0.985	1.087	0.175
		Lag 3	Lag 0	OR01	1.003	0.997	1.009	0.318
				OR10	1.015	0.966	1.066	0.553
			Lag 1	OR01	1.001	0.994	1.007	0.844
				OR10	1.018	0.969	1.069	0.485
			Lag 2	OR01	0.998	0.992	1.005	0.568
				OR10	1.018	0.969	1.069	0.480
			Lag 3	OR01	0.995	0.987	1.002	0.130
				OR10	1.015	0.966	1.066	0.567
	Renal	Lag 0	Lag 0	OR01	0.993	0.975	1.011	0.438
				OR10	1.090	1.007	1.180	**0.033**
			Lag 1	OR01	0.993	0.977	1.009	0.393
				OR10	1.104	1.019	1.195	**0.015**
			Lag 2	OR01	1.006	0.994	1.019	0.336
				OR10	1.093	1.009	1.183	**0.028**
			Lag 3	OR01	0.999	0.984	1.014	0.882
				OR10	1.087	1.005	1.177	**0.038**
		Lag 1	Lag 0	OR01	0.992	0.973	1.010	0.370
				OR10	1.106	1.023	1.194	**0.011**
			Lag 1	OR01	0.992	0.976	1.009	0.357
				OR10	1.118	1.035	1.208	**0.005**
			Lag 2	OR01	1.007	0.995	1.020	0.264
				OR10	1.118	1.035	1.208	**0.005**
			Lag 3	OR01	0.998	0.984	1.013	0.802
				OR10	1.101	1.019	1.189	**0.015**
		Lag 2	Lag 0	OR01	0.997	0.979	1.014	0.695
				OR10	1.063	0.984	1.149	0.122

			Lag 1	OR01	0.992	0.976	1.009	0.357
				OR10	1.064	0.985	1.149	0.117
			Lag 2	OR01	1.006	0.994	1.019	0.327
				OR10	1.060	0.981	1.146	0.143
			Lag 3	OR01	0.997	0.982	1.012	0.684
				OR10	1.049	0.970	1.134	0.230
		Lag 3	Lag 0	OR01	0.999	0.982	1.016	0.893
				OR10	1.044	0.966	1.127	0.277
			Lag 1	OR01	0.993	0.977	1.009	0.391
				OR10	1.038	0.962	1.121	0.337
			Lag 2	OR01	1.006	0.993	1.020	0.334
				OR10	1.032	0.956	1.115	0.420
			Lag 3	OR01	0.999	0.984	1.014	0.860
				OR10	1.030	0.953	1.112	0.455
	Respiratory	Lag 0	Lag 0	OR01	0.996	0.984	1.008	0.489
				OR10	0.968	0.900	1.042	0.385
			Lag 1	OR01	0.999	0.990	1.009	0.841
				OR10	0.963	0.895	1.036	0.311
			Lag 2	OR01	1.002	0.992	1.013	0.655
				OR10	0.968	0.900	1.041	0.382
			Lag 3	OR01	1.001	0.991	1.012	0.848
				OR10	0.954	0.887	1.027	0.210
		Lag 1	Lag 0	OR01	0.996	0.985	1.008	0.537
				OR10	1.008	0.938	1.083	0.826
			Lag 1	OR01	0.999	0.989	1.008	0.773
				OR10	0.996	0.927	1.071	0.922
			Lag 2	OR01	1.003	0.992	1.014	0.609
				OR10	1.007	0.938	1.082	0.848
			Lag 3	OR01	1.001	0.991	1.012	0.821
				OR10	0.989	0.920	1.063	0.766
		Lag 2	Lag 0	OR01	0.993	0.981	1.006	0.297
				OR10	1.007	0.938	1.082	0.846
			Lag 1	OR01	0.997	0.988	1.007	0.607
				OR10	1.009	0.940	1.084	0.802
			Lag 2	OR01	1.003	0.992	1.014	0.631
				OR10	1.021	0.950	1.096	0.575
			Lag 3	OR01	1.002	0.991	1.012	0.724
				OR10	1.011	0.941	1.086	0.760
		Lag 3	Lag 0	OR01	0.996	0.984	1.008	0.499
				OR10	1.038	0.968	1.112	0.298
			Lag 1	OR01	1.000	0.991	1.010	0.954
				OR10	1.043	0.973	1.117	0.236

			Lag 2	OR01	1.003	0.992	1.014	0.556
				OR10	1.042	0.972	1.116	0.246
			Lag 3	OR01	1.002	0.991	1.012	0.722
				OR10	1.030	0.961	1.105	0.399
Male	All-natural	Lag 0	Lag 0	OR01	1.000	1.000	1.001	0.535
				OR10	1.029	1.025	1.033	**0.000**
			Lag 1	OR01	1.000	0.999	1.000	0.849
				OR10	1.030	1.025	1.034	**0.000**
			Lag 2	OR01	0.999	0.999	1.000	0.054
				OR10	1.030	1.026	1.034	**0.000**
			Lag 3	OR01	0.999	0.999	1.000	**0.003**
				OR10	1.029	1.025	1.034	**0.000**
		Lag 1	Lag 0	OR01	1.000	1.000	1.001	0.854
				OR10	1.026	1.021	1.030	**0.000**
			Lag 1	OR01	1.000	0.999	1.001	0.949
				OR10	1.028	1.023	1.032	**0.000**
			Lag 2	OR01	0.999	0.999	1.000	0.051
				OR10	1.028	1.023	1.032	**0.000**
			Lag 3	OR01	0.999	0.999	1.000	**0.003**
				OR10	1.027	1.022	1.031	**0.000**
		Lag 2	Lag 0	OR01	1.000	1.000	1.001	0.717
				OR10	1.020	1.015	1.024	**0.000**
			Lag 1	OR01	1.000	0.999	1.001	0.991
				OR10	1.022	1.017	1.026	**0.000**
			Lag 2	OR01	1.000	0.999	1.000	0.089
				OR10	1.022	1.018	1.026	**0.000**
			Lag 3	OR01	0.999	0.999	1.000	**0.006**
				OR10	1.022	1.017	1.026	**0.000**
		Lag 3	Lag 0	OR01	1.000	1.000	1.001	0.687
				OR10	1.013	1.008	1.017	**0.000**
			Lag 1	OR01	1.000	0.999	1.000	0.673
				OR10	1.014	1.010	1.018	**0.000**
			Lag 2	OR01	1.000	0.999	1.000	0.142
				OR10	1.015	1.011	1.020	**0.000**
			Lag 3	OR01	0.999	0.999	1.000	**0.005**
				OR10	1.014	1.010	1.019	**0.000**
	Cardiovascular	Lag 0	Lag 0	OR01	1.003	0.993	1.014	0.531
				OR10	1.058	0.979	1.144	0.155
			Lag 1	OR01	1.009	1.000	1.018	0.064
				OR10	1.049	0.970	1.135	0.227
			Lag 2	OR01	1.002	0.992	1.011	0.722
				OR10	1.056	0.977	1.143	0.171

			Lag 3	OR01	1.006	0.996	1.016	0.232
				OR10	1.060	0.980	1.146	0.144
		Lag 1	Lag 0	OR01	1.003	0.993	1.014	0.577
				OR10	1.083	1.001	1.170	**0.046**
			Lag 1	OR01	1.009	1.000	1.018	0.061
				OR10	1.076	0.995	1.163	0.066
			Lag 2	OR01	1.001	0.991	1.011	0.876
				OR10	1.073	0.992	1.160	0.080
			Lag 3	OR01	1.006	0.996	1.016	0.206
				OR10	1.090	1.008	1.179	**0.030**
		Lag 2	Lag 0	OR01	1.003	0.992	1.013	0.592
				OR10	1.025	0.948	1.108	0.535
			Lag 1	OR01	1.009	1.000	1.019	0.056
				OR10	1.023	0.946	1.106	0.566
			Lag 2	OR01	1.002	0.992	1.011	0.732
				OR10	1.028	0.950	1.111	0.495
			Lag 3	OR01	1.006	0.996	1.016	0.242
				OR10	1.030	0.953	1.113	0.463
		Lag 3	Lag 0	OR01	1.003	0.993	1.013	0.586
				OR10	0.985	0.910	1.066	0.706
			Lag 1	OR01	1.009	1.000	1.018	0.062
				OR10	0.982	0.908	1.062	0.656
			Lag 2	OR01	1.003	0.993	1.012	0.575
				OR10	0.994	0.920	1.075	0.886
			Lag 3	OR01	1.005	0.995	1.015	0.341
				OR10	0.982	0.908	1.062	0.655
	Cerebrovascular	Lag 0	Lag 0	OR01	1.004	0.995	1.013	0.417
				OR10	1.006	0.938	1.079	0.870
			Lag 1	OR01	1.001	0.992	1.010	0.799
				OR10	1.007	0.939	1.081	0.836
			Lag 2	OR01	0.999	0.990	1.008	0.859
				OR10	0.992	0.925	1.065	0.831
			Lag 3	OR01	0.999	0.990	1.008	0.801
				OR10	1.001	0.933	1.073	0.988
		Lag 1	Lag 0	OR01	1.003	0.994	1.012	0.480
				OR10	0.992	0.926	1.064	0.832
			Lag 1	OR01	1.001	0.992	1.010	0.843
				OR10	0.995	0.928	1.067	0.884
			Lag 2	OR01	1.000	0.992	1.009	0.931
				OR10	0.997	0.929	1.069	0.922
			Lag 3	OR01	0.999	0.990	1.009	0.885
				OR10	0.996	0.929	1.068	0.908

		Lag 2	Lag 0	OR01	1.004	0.995	1.013	0.367
				OR10	1.060	0.990	1.135	0.092
			Lag 1	OR01	1.000	0.991	1.009	0.956
				OR10	1.051	0.982	1.126	0.150
			Lag 2	OR01	1.000	0.991	1.009	0.973
				OR10	1.055	0.985	1.129	0.128
			Lag 3	OR01	0.999	0.990	1.009	0.894
				OR10	1.055	0.986	1.130	0.121
		Lag 3	Lag 0	OR01	1.003	0.995	1.012	0.457
				OR10	1.070	1.001	1.144	**0.048**
			Lag 1	OR01	1.000	0.991	1.009	0.980
				OR10	1.066	0.996	1.140	0.064
			Lag 2	OR01	0.998	0.989	1.007	0.700
				OR10	1.062	0.993	1.136	0.081
			Lag 3	OR01	0.999	0.989	1.008	0.760
				OR10	1.067	0.998	1.142	0.058
	Renal	Lag 0	Lag 0	OR01	0.969	0.938	1.001	0.054
				OR10	1.131	1.007	1.271	**0.038**
			Lag 1	OR01	0.998	0.976	1.021	0.857
				OR10	1.139	1.013	1.281	**0.029**
			Lag 2	OR01	1.017	0.997	1.038	0.091
				OR10	1.119	0.995	1.257	0.061
			Lag 3	OR01	1.008	0.987	1.030	0.449
				OR10	1.134	1.009	1.274	**0.034**
		Lag 1	Lag 0	OR01	0.969	0.938	1.000	0.053
				OR10	1.132	1.013	1.266	**0.029**
			Lag 1	OR01	0.998	0.976	1.021	0.879
				OR10	1.144	1.023	1.279	**0.019**
			Lag 2	OR01	1.021	1.001	1.041	**0.042**
				OR10	1.146	1.025	1.281	**0.017**
			Lag 3	OR01	1.006	0.984	1.028	0.611
				OR10	1.122	1.003	1.255	**0.044**
		Lag 2	Lag 0	OR01	0.975	0.946	1.005	0.105
				OR10	1.103	0.986	1.235	0.086
			Lag 1	OR01	0.999	0.978	1.022	0.948
				OR10	1.107	0.989	1.238	0.077
			Lag 2	OR01	1.020	1.000	1.041	0.050
				OR10	1.101	0.983	1.233	0.096
			Lag 3	OR01	1.006	0.984	1.028	0.594
				OR10	1.091	0.974	1.221	0.134
		Lag 3	Lag 0	OR01	0.981	0.953	1.009	0.182
				OR10	1.146	1.025	1.282	**0.017**

			Lag 1	OR01	0.998	0.977	1.021	0.887
				OR10	1.129	1.009	1.262	**0.034**
			Lag 2	OR01	1.019	0.999	1.040	0.064
				OR10	1.118	0.999	1.251	0.052
			Lag 3	OR01	1.006	0.984	1.028	0.604
				OR10	1.114	0.996	1.247	0.060
	Respiratory	Lag 0	Lag 0	OR01	1.005	0.992	1.018	0.444
				OR10	0.964	0.868	1.072	0.499
			Lag 1	OR01	1.002	0.990	1.013	0.782
				OR10	0.952	0.856	1.058	0.361
			Lag 2	OR01	0.998	0.983	1.013	0.809
				OR10	0.961	0.865	1.068	0.463
			Lag 3	OR01	1.001	0.987	1.016	0.844
				OR10	0.940	0.846	1.045	0.254
		Lag 1	Lag 0	OR01	1.006	0.994	1.019	0.329
				OR10	1.017	0.917	1.127	0.752
			Lag 1	OR01	1.002	0.991	1.013	0.737
				OR10	0.993	0.896	1.101	0.896
			Lag 2	OR01	0.999	0.984	1.014	0.924
				OR10	1.007	0.909	1.116	0.889
			Lag 3	OR01	1.002	0.989	1.016	0.730
				OR10	0.983	0.887	1.090	0.749
		Lag 2	Lag 0	OR01	1.004	0.991	1.017	0.554
				OR10	0.954	0.860	1.059	0.380
			Lag 1	OR01	1.002	0.991	1.013	0.708
				OR10	0.962	0.867	1.068	0.470
			Lag 2	OR01	1.000	0.985	1.015	0.951
				OR10	0.972	0.876	1.078	0.588
			Lag 3	OR01	1.004	0.990	1.017	0.615
				OR10	0.961	0.866	1.067	0.454
		Lag 3	Lag 0	OR01	1.005	0.992	1.018	0.434
				OR10	1.055	0.955	1.164	0.292
			Lag 1	OR01	1.001	0.989	1.013	0.840
				OR10	1.057	0.959	1.166	0.263
			Lag 2	OR01	1.000	0.986	1.015	0.949
				OR10	1.069	0.968	1.179	0.186
			Lag 3	OR01	1.004	0.991	1.018	0.544
				OR10	1.057	0.957	1.167	0.275
Other Races Grouped	All-natural	Lag 0	Lag 0	OR01	1.002	1.000	1.004	**0.022**
				OR10	1.017	1.002	1.031	**0.021**
			Lag 1	OR01	1.001	0.999	1.002	0.457
				OR10	1.017	1.003	1.031	**0.020**

			Lag 2	OR01	1.000	0.998	1.002	0.932
				OR10	1.016	1.002	1.031	**0.025**
			Lag 3	OR01	0.999	0.997	1.000	0.140
				OR10	1.017	1.003	1.031	**0.019**
		Lag 1	Lag 0	OR01	1.002	1.000	1.004	**0.029**
				OR10	1.000	0.986	1.015	0.957
			Lag 1	OR01	1.001	0.999	1.003	0.308
				OR10	1.003	0.989	1.017	0.649
			Lag 2	OR01	1.000	0.998	1.002	0.987
				OR10	1.001	0.987	1.015	0.944
			Lag 3	OR01	0.999	0.997	1.001	0.160
				OR10	1.002	0.988	1.016	0.790
		Lag 2	Lag 0	OR01	1.002	1.000	1.004	**0.019**
				OR10	1.016	1.002	1.030	**0.029**
			Lag 1	OR01	1.001	0.999	1.003	0.221
				OR10	1.019	1.005	1.033	**0.009**
			Lag 2	OR01	1.000	0.998	1.002	0.875
				OR10	1.016	1.002	1.030	**0.025**
			Lag 3	OR01	0.999	0.997	1.001	0.204
				OR10	1.017	1.003	1.032	**0.015**
		Lag 3	Lag 0	OR01	1.002	1.001	1.004	**0.010**
				OR10	1.014	1.000	1.028	**0.044**
			Lag 1	OR01	1.001	0.999	1.003	0.410
				OR10	1.013	0.999	1.027	0.063
			Lag 2	OR01	1.000	0.998	1.002	0.876
				OR10	1.013	0.999	1.027	0.062
			Lag 3	OR01	0.999	0.997	1.001	0.299
				OR10	1.016	1.002	1.030	**0.024**
	Cardiovascular	Lag 0	Lag 0	OR01	0.997	0.949	1.047	0.900
				OR10	1.026	0.763	1.379	0.867
			Lag 1	OR01	0.933	0.836	1.041	0.215
				OR10	0.985	0.731	1.328	0.922
			Lag 2	OR01	1.019	0.967	1.073	0.482
				OR10	0.976	0.722	1.320	0.877
			Lag 3	OR01	1.036	0.979	1.098	0.221
				OR10	1.024	0.761	1.378	0.877
		Lag 1	Lag 0	OR01	0.992	0.946	1.041	0.746
				OR10	0.923	0.682	1.249	0.605
			Lag 1	OR01	0.927	0.826	1.040	0.199
				OR10	0.925	0.685	1.249	0.609
			Lag 2	OR01	1.021	0.969	1.076	0.433
				OR10	0.944	0.700	1.274	0.706

			Lag 3	OR01	1.041	0.981	1.104	0.189
				OR10	0.975	0.725	1.311	0.867
		Lag 2	Lag 0	OR01	0.994	0.948	1.041	0.783
				OR10	0.859	0.636	1.161	0.324
			Lag 1	OR01	0.988	0.944	1.035	0.620
				OR10	0.949	0.707	1.274	0.727
			Lag 2	OR01	1.025	0.973	1.080	0.343
				OR10	0.919	0.684	1.234	0.573
			Lag 3	OR01	1.031	0.976	1.088	0.275
				OR10	0.855	0.633	1.156	0.309
		Lag 3	Lag 0	OR01	0.999	0.955	1.045	0.971
				OR10	0.915	0.685	1.222	0.546
			Lag 1	OR01	0.984	0.938	1.033	0.523
				OR10	0.948	0.713	1.260	0.715
			Lag 2	OR01	1.028	0.976	1.083	0.297
				OR10	0.966	0.728	1.282	0.810
			Lag 3	OR01	1.038	0.980	1.099	0.204
				OR10	0.938	0.705	1.248	0.659
	Cerebrovascular	Lag 0	Lag 0	OR01	0.996	0.964	1.028	0.790
				OR10	0.856	0.658	1.113	0.245
			Lag 1	OR01	0.980	0.940	1.022	0.354
				OR10	0.898	0.694	1.162	0.413
			Lag 2	OR01	0.972	0.927	1.020	0.249
				OR10	0.893	0.691	1.154	0.387
			Lag 3	OR01	0.939	0.867	1.016	0.119
				OR10	0.887	0.685	1.147	0.360
		Lag 1	Lag 0	OR01	0.997	0.966	1.029	0.837
				OR10	0.785	0.608	1.013	0.063
			Lag 1	OR01	0.985	0.946	1.025	0.452
				OR10	0.827	0.643	1.063	0.138
			Lag 2	OR01	0.976	0.934	1.020	0.285
				OR10	0.828	0.644	1.065	0.142
			Lag 3	OR01	0.949	0.880	1.024	0.174
				OR10	0.819	0.636	1.053	0.120
		Lag 2	Lag 0	OR01	0.997	0.966	1.029	0.865
				OR10	0.911	0.714	1.161	0.450
			Lag 1	OR01	0.981	0.941	1.023	0.376
				OR10	0.933	0.733	1.188	0.575
			Lag 2	OR01	0.975	0.932	1.021	0.283
				OR10	0.939	0.736	1.198	0.614
			Lag 3	OR01	0.942	0.870	1.020	0.141
				OR10	0.925	0.725	1.180	0.531

		Lag 3	Lag 0	OR01	1.001	0.971	1.031	0.948
				OR10	0.987	0.775	1.257	0.916
			Lag 1	OR01	0.983	0.945	1.024	0.409
				OR10	0.985	0.774	1.253	0.903
			Lag 2	OR01	0.974	0.929	1.021	0.270
				OR10	0.978	0.765	1.251	0.859
			Lag 3	OR01	0.940	0.868	1.018	0.129
				OR10	0.962	0.753	1.229	0.755
	Renal	Lag 0	Lag 0	OR01	1.077	0.917	1.266	0.365
				OR10	1.004	0.649	1.553	0.986
			Lag 1	OR01	0.799	0.603	1.059	0.119
				OR10	1.014	0.655	1.571	0.949
			Lag 2	OR01	0.981	0.894	1.076	0.689
				OR10	0.944	0.608	1.466	0.798
			Lag 3	OR01	0.934	0.796	1.096	0.402
				OR10	1.029	0.671	1.578	0.894
		Lag 1	Lag 0	OR01	1.048	0.924	1.189	0.464
				OR10	0.981	0.638	1.511	0.932
			Lag 1	OR01	0.759	0.554	1.041	0.087
				OR10	0.986	0.640	1.519	0.949
			Lag 2	OR01	0.963	0.835	1.110	0.603
				OR10	0.878	0.566	1.361	0.561
			Lag 3	OR01	0.933	0.794	1.096	0.398
				OR10	1.011	0.665	1.538	0.959
		Lag 2	Lag 0	OR01	1.060	0.944	1.189	0.324
				OR10	1.341	0.906	1.986	0.142
			Lag 1	OR01	0.813	0.628	1.053	0.117
				OR10	1.330	0.896	1.973	0.157
			Lag 2	OR01	0.985	0.904	1.074	0.730
				OR10	1.261	0.845	1.881	0.256
			Lag 3	OR01	0.940	0.807	1.095	0.430
				OR10	1.375	0.932	2.029	0.109
		Lag 3	Lag 0	OR01	1.060	0.945	1.188	0.324
				OR10	1.211	0.804	1.824	0.360
			Lag 1	OR01	0.866	0.699	1.073	0.188
				OR10	1.253	0.832	1.887	0.281
			Lag 2	OR01	0.974	0.871	1.090	0.649
				OR10	1.063	0.695	1.625	0.779
			Lag 3	OR01	0.931	0.790	1.097	0.393
				OR10	1.230	0.819	1.848	0.319
	Respiratory	Lag 0	Lag 0	OR01	1.023	0.919	1.140	0.675
				OR10	0.903	0.636	1.280	0.565

			Lag 1	OR01	0.996	0.944	1.050	0.879
				OR10	0.864	0.608	1.228	0.416
			Lag 2	OR01	1.017	0.898	1.153	0.790
				OR10	0.903	0.638	1.279	0.565
			Lag 3	OR01	0.983	0.921	1.050	0.617
				OR10	0.933	0.660	1.320	0.695
		Lag 1	Lag 0	OR01	1.033	0.926	1.152	0.562
				OR10	0.939	0.653	1.350	0.733
			Lag 1	OR01	1.001	0.954	1.050	0.973
				OR10	0.953	0.664	1.368	0.796
			Lag 2	OR01	1.007	0.891	1.138	0.910
				OR10	0.916	0.638	1.316	0.636
			Lag 3	OR01	0.979	0.909	1.055	0.578
				OR10	0.924	0.645	1.325	0.668
		Lag 2	Lag 0	OR01	1.010	0.902	1.131	0.860
				OR10	1.147	0.799	1.647	0.458
			Lag 1	OR01	1.000	0.952	1.051	0.991
				OR10	1.211	0.847	1.730	0.294
			Lag 2	OR01	1.012	0.898	1.141	0.842
				OR10	1.211	0.850	1.726	0.289
			Lag 3	OR01	0.974	0.892	1.062	0.547
				OR10	1.172	0.820	1.676	0.384
		Lag 3	Lag 0	OR01	1.010	0.901	1.133	0.860
				OR10	1.145	0.811	1.618	0.442
			Lag 1	OR01	0.989	0.927	1.055	0.739
				OR10	1.119	0.793	1.581	0.522
			Lag 2	OR01	0.998	0.879	1.134	0.979
				OR10	1.158	0.822	1.632	0.401
			Lag 3	OR01	0.983	0.918	1.053	0.620
				OR10	1.203	0.854	1.694	0.291
English, preferred language	All-natural	Lag 0	Lag 0	OR01	1.000	1.000	1.000	0.803
				OR10	1.023	1.020	1.026	**0.000**
			Lag 1	OR01	1.000	0.999	1.000	0.163
				OR10	1.023	1.020	1.026	**0.000**
			Lag 2	OR01	0.999	0.999	1.000	**0.000**
				OR10	1.024	1.021	1.027	**0.000**
			Lag 3	OR01	0.999	0.999	0.999	**0.000**
				OR10	1.023	1.020	1.026	**0.000**
		Lag 1	Lag 0	OR01	1.000	0.999	1.000	0.537
				OR10	1.022	1.019	1.025	**0.000**
			Lag 1	OR01	1.000	0.999	1.000	0.275
				OR10	1.024	1.021	1.027	**0.000**

			Lag 2	OR01	0.999	0.999	1.000	**0.000**
				OR10	1.024	1.021	1.027	**0.000**
			Lag 3	OR01	0.999	0.999	0.999	**0.000**
				OR10	1.023	1.020	1.026	**0.000**
		Lag 2	Lag 0	OR01	1.000	1.000	1.000	0.628
				OR10	1.017	1.014	1.020	**0.000**
			Lag 1	OR01	1.000	0.999	1.000	0.304
				OR10	1.019	1.016	1.022	**0.000**
			Lag 2	OR01	0.999	0.999	1.000	**0.001**
				OR10	1.020	1.017	1.023	**0.000**
			Lag 3	OR01	0.999	0.999	1.000	**0.000**
				OR10	1.019	1.016	1.022	**0.000**
		Lag 3	Lag 0	OR01	1.000	1.000	1.000	0.748
				OR10	1.013	1.010	1.016	**0.000**
			Lag 1	OR01	1.000	0.999	1.000	0.083
				OR10	1.014	1.011	1.017	**0.000**
			Lag 2	OR01	0.999	0.999	1.000	**0.001**
				OR10	1.016	1.013	1.019	**0.000**
			Lag 3	OR01	0.999	0.999	0.999	**0.000**
				OR10	1.014	1.011	1.017	**0.000**
	Cardiovascular	Lag 0	Lag 0	OR01	1.000	0.991	1.009	0.947
				OR10	1.037	0.972	1.106	0.276
			Lag 1	OR01	1.003	0.994	1.013	0.458
				OR10	1.039	0.974	1.109	0.242
			Lag 2	OR01	1.001	0.992	1.010	0.805
				OR10	1.039	0.974	1.109	0.245
			Lag 3	OR01	0.999	0.989	1.008	0.811
				OR10	1.049	0.983	1.119	0.150
		Lag 1	Lag 0	OR01	1.000	0.991	1.009	0.964
				OR10	1.053	0.988	1.122	0.114
			Lag 1	OR01	1.004	0.995	1.013	0.419
				OR10	1.058	0.993	1.128	0.083
			Lag 2	OR01	1.001	0.992	1.010	0.898
				OR10	1.052	0.987	1.122	0.120
			Lag 3	OR01	0.998	0.988	1.008	0.678
				OR10	1.059	0.993	1.128	0.080
		Lag 2	Lag 0	OR01	1.000	0.990	1.009	0.917
				OR10	1.057	0.993	1.125	0.083
			Lag 1	OR01	1.005	0.996	1.014	0.310
				OR10	1.073	1.008	1.142	**0.028**
			Lag 2	OR01	1.002	0.993	1.011	0.690
				OR10	1.073	1.007	1.142	**0.029**

		Lag 3	OR01	0.999	0.989	1.008	0.797
			OR10	1.073	1.008	1.143	**0.026**
	Lag 3	Lag 0	OR01	1.000	0.991	1.009	0.994
			OR10	1.038	0.975	1.106	0.243
		Lag 1	OR01	1.004	0.995	1.013	0.353
			OR10	1.052	0.988	1.120	0.114
		Lag 2	OR01	1.002	0.994	1.012	0.586
			OR10	1.057	0.992	1.125	0.085
		Lag 3	OR01	0.998	0.988	1.008	0.719
			OR10	1.052	0.988	1.120	0.115
Cerebrovascular	Lag 0	Lag 0	OR01	1.004	0.997	1.011	0.276
			OR10	1.031	0.976	1.089	0.276
		Lag 1	OR01	0.999	0.991	1.006	0.685
			OR10	1.027	0.972	1.084	0.346
		Lag 2	OR01	0.997	0.990	1.004	0.426
			OR10	1.027	0.972	1.085	0.341
		Lag 3	OR01	0.995	0.987	1.003	0.232
			OR10	1.028	0.973	1.085	0.325
	Lag 1	Lag 0	OR01	1.003	0.996	1.010	0.365
			OR10	1.039	0.984	1.097	0.163
		Lag 1	OR01	0.998	0.991	1.005	0.601
			OR10	1.038	0.983	1.096	0.175
		Lag 2	OR01	0.997	0.990	1.004	0.409
			OR10	1.040	0.985	1.098	0.154
		Lag 3	OR01	0.995	0.987	1.003	0.251
			OR10	1.044	0.989	1.102	0.121
	Lag 2	Lag 0	OR01	1.003	0.997	1.010	0.320
			OR10	1.043	0.989	1.100	0.123
		Lag 1	OR01	0.997	0.990	1.005	0.502
			OR10	1.038	0.984	1.095	0.169
		Lag 2	OR01	0.997	0.990	1.004	0.409
			OR10	1.043	0.989	1.101	0.122
		Lag 3	OR01	0.995	0.987	1.003	0.213
			OR10	1.043	0.988	1.100	0.125
	Lag 3	Lag 0	OR01	1.003	0.997	1.010	0.334
			OR10	1.024	0.971	1.081	0.383
		Lag 1	OR01	0.998	0.991	1.005	0.566
			OR10	1.022	0.968	1.078	0.434
		Lag 2	OR01	0.997	0.990	1.004	0.445
			OR10	1.027	0.974	1.084	0.324
		Lag 3	OR01	0.995	0.986	1.003	0.201
			OR10	1.025	0.972	1.082	0.366

Renal	Lag 0	Lag 0	OR01	0.996	0.979	1.014	0.686
			OR10	1.110	1.019	1.209	**0.017**
		Lag 1	OR01	0.995	0.979	1.012	0.581
			OR10	1.119	1.027	1.220	**0.010**
		Lag 2	OR01	1.008	0.993	1.023	0.286
			OR10	1.108	1.017	1.207	**0.019**
		Lag 3	OR01	0.998	0.982	1.014	0.811
			OR10	1.106	1.015	1.205	**0.022**
	Lag 1	Lag 0	OR01	0.994	0.976	1.013	0.545
			OR10	1.132	1.041	1.230	**0.004**
		Lag 1	OR01	0.996	0.980	1.013	0.670
			OR10	1.151	1.059	1.251	**0.001**
		Lag 2	OR01	1.010	0.996	1.025	0.172
			OR10	1.149	1.057	1.250	**0.001**
		Lag 3	OR01	0.997	0.981	1.014	0.762
			OR10	1.130	1.039	1.228	**0.004**
	Lag 2	Lag 0	OR01	1.000	0.983	1.017	0.990
			OR10	1.084	0.997	1.179	0.058
		Lag 1	OR01	0.996	0.980	1.013	0.672
			OR10	1.087	1.000	1.182	0.051
		Lag 2	OR01	1.008	0.994	1.023	0.273
			OR10	1.077	0.990	1.171	0.085
		Lag 3	OR01	0.995	0.978	1.012	0.551
			OR10	1.065	0.979	1.158	0.145
	Lag 3	Lag 0	OR01	1.002	0.986	1.019	0.772
			OR10	1.054	0.970	1.146	0.216
		Lag 1	OR01	0.996	0.980	1.013	0.644
			OR10	1.046	0.962	1.137	0.293
		Lag 2	OR01	1.010	0.995	1.025	0.209
			OR10	1.042	0.959	1.133	0.331
		Lag 3	OR01	0.996	0.980	1.013	0.683
			OR10	1.034	0.951	1.124	0.435
Respiratory	Lag 0	Lag 0	OR01	0.994	0.980	1.007	0.361
			OR10	0.929	0.858	1.006	0.069
		Lag 1	OR01	1.003	0.994	1.013	0.479
			OR10	0.920	0.850	0.996	**0.039**
		Lag 2	OR01	1.007	0.996	1.019	0.201
			OR10	0.932	0.861	1.008	0.079
		Lag 3	OR01	1.004	0.993	1.015	0.528
			OR10	0.907	0.837	0.982	**0.016**
	Lag 1	Lag 0	OR01	0.995	0.982	1.008	0.469
			OR10	0.995	0.921	1.075	0.903

			Lag 1	OR01	1.004	0.994	1.013	0.459
				OR10	0.979	0.906	1.058	0.593
			Lag 2	OR01	1.008	0.996	1.019	0.188
				OR10	0.990	0.916	1.069	0.792
			Lag 3	OR01	1.005	0.994	1.016	0.423
				OR10	0.969	0.897	1.048	0.433
		Lag 2	Lag 0	OR01	0.991	0.977	1.006	0.233
				OR10	0.998	0.925	1.077	0.957
			Lag 1	OR01	1.002	0.993	1.012	0.652
				OR10	0.996	0.923	1.075	0.917
			Lag 2	OR01	1.007	0.996	1.019	0.218
				OR10	1.009	0.935	1.089	0.822
			Lag 3	OR01	1.005	0.994	1.016	0.343
				OR10	1.001	0.927	1.080	0.985
		Lag 3	Lag 0	OR01	0.994	0.981	1.008	0.385
				OR10	1.063	0.987	1.144	0.105
			Lag 1	OR01	1.003	0.993	1.012	0.610
				OR10	1.056	0.982	1.136	0.140
			Lag 2	OR01	1.008	0.997	1.020	0.161
				OR10	1.067	0.992	1.148	0.083
			Lag 3	OR01	1.006	0.995	1.017	0.326
				OR10	1.056	0.981	1.137	0.150
Other, preferred language	All-natural	Lag 0	Lag 0	OR01	1.001	1.000	1.002	0.173
				OR10	1.031	1.018	1.045	**0.000**
			Lag 1	OR01	1.000	0.999	1.002	0.618
				OR10	1.029	1.016	1.043	**0.000**
			Lag 2	OR01	1.000	0.999	1.002	0.600
				OR10	1.032	1.018	1.046	**0.000**
			Lag 3	OR01	1.000	0.998	1.001	0.738
				OR10	1.033	1.019	1.047	**0.000**
		Lag 1	Lag 0	OR01	1.001	0.999	1.002	0.262
				OR10	1.026	1.013	1.040	**0.000**
			Lag 1	OR01	1.000	0.999	1.001	0.858
				OR10	1.023	1.010	1.037	**0.001**
			Lag 2	OR01	1.000	0.999	1.001	0.856
				OR10	1.026	1.013	1.040	**0.000**
			Lag 3	OR01	1.000	0.998	1.001	0.755
				OR10	1.028	1.015	1.042	**0.000**
		Lag 2	Lag 0	OR01	1.001	1.000	1.002	0.198
				OR10	1.028	1.015	1.041	**0.000**
			Lag 1	OR01	1.000	0.999	1.002	0.661
				OR10	1.028	1.015	1.042	**0.000**

			Lag 2	OR01	1.000	0.999	1.001	0.932
				OR10	1.028	1.014	1.041	**0.000**
			Lag 3	OR01	1.000	0.998	1.001	0.834
				OR10	1.031	1.018	1.045	**0.000**
		Lag 3	Lag 0	OR01	1.001	1.000	1.002	0.148
				OR10	1.017	1.003	1.030	**0.013**
			Lag 1	OR01	1.001	0.999	1.002	0.319
				OR10	1.018	1.005	1.032	**0.006**
			Lag 2	OR01	1.000	0.999	1.002	0.705
				OR10	1.018	1.005	1.031	**0.008**
			Lag 3	OR01	1.000	0.999	1.001	0.905
				OR10	1.020	1.007	1.033	**0.003**
	Cardiovascular	Lag 0	Lag 0	OR01	1.002	0.969	1.035	0.925
				OR10	1.051	0.821	1.345	0.694
			Lag 1	OR01	0.988	0.950	1.027	0.538
				OR10	1.011	0.787	1.298	0.932
			Lag 2	OR01	0.973	0.930	1.017	0.228
				OR10	1.034	0.807	1.325	0.791
			Lag 3	OR01	0.974	0.932	1.018	0.243
				OR10	1.047	0.818	1.339	0.716
		Lag 1	Lag 0	OR01	0.995	0.959	1.031	0.772
				OR10	1.141	0.888	1.465	0.302
			Lag 1	OR01	0.983	0.940	1.027	0.440
				OR10	1.135	0.882	1.461	0.324
			Lag 2	OR01	0.961	0.902	1.023	0.208
				OR10	1.132	0.881	1.454	0.332
			Lag 3	OR01	0.970	0.923	1.019	0.222
				OR10	1.149	0.895	1.475	0.277
		Lag 2	Lag 0	OR01	0.992	0.956	1.029	0.667
				OR10	0.885	0.685	1.142	0.347
			Lag 1	OR01	0.985	0.943	1.029	0.498
				OR10	0.901	0.698	1.163	0.423
			Lag 2	OR01	0.962	0.906	1.022	0.206
				OR10	0.888	0.687	1.147	0.363
			Lag 3	OR01	0.971	0.925	1.019	0.231
				OR10	0.906	0.703	1.169	0.450
		Lag 3	Lag 0	OR01	0.993	0.957	1.030	0.709
				OR10	1.007	0.788	1.286	0.956
			Lag 1	OR01	0.981	0.935	1.028	0.423
				OR10	1.015	0.795	1.296	0.905
			Lag 2	OR01	0.962	0.904	1.024	0.221
				OR10	1.013	0.793	1.294	0.919

			Lag 3	OR01	0.972	0.926	1.020	0.243
				OR10	1.034	0.809	1.321	0.792
	Cerebrovascular	Lag 0	Lag 0	OR01	0.999	0.984	1.015	0.949
				OR10	0.876	0.717	1.071	0.196
			Lag 1	OR01	1.012	0.993	1.030	0.223
				OR10	0.920	0.754	1.122	0.409
			Lag 2	OR01	0.993	0.972	1.015	0.531
				OR10	0.895	0.733	1.093	0.277
			Lag 3	OR01	0.988	0.967	1.009	0.263
				OR10	0.898	0.736	1.097	0.292
		Lag 1	Lag 0	OR01	1.002	0.987	1.017	0.797
				OR10	1.097	0.907	1.326	0.341
			Lag 1	OR01	1.012	0.994	1.031	0.194
				OR10	1.114	0.920	1.349	0.268
			Lag 2	OR01	0.995	0.974	1.016	0.635
				OR10	1.100	0.909	1.331	0.327
			Lag 3	OR01	0.990	0.970	1.010	0.321
				OR10	1.107	0.916	1.338	0.294
		Lag 2	Lag 0	OR01	1.002	0.986	1.017	0.838
				OR10	1.137	0.944	1.369	0.177
			Lag 1	OR01	1.013	0.994	1.032	0.191
				OR10	1.154	0.958	1.390	0.132
			Lag 2	OR01	0.997	0.976	1.017	0.752
				OR10	1.170	0.970	1.411	0.101
			Lag 3	OR01	0.989	0.968	1.010	0.300
				OR10	1.134	0.942	1.367	0.185
		Lag 3	Lag 0	OR01	1.000	0.985	1.016	0.986
				OR10	1.001	0.829	1.209	0.991
			Lag 1	OR01	1.011	0.992	1.030	0.252
				OR10	1.019	0.844	1.230	0.845
			Lag 2	OR01	0.987	0.962	1.012	0.303
				OR10	0.990	0.820	1.195	0.915
			Lag 3	OR01	0.984	0.960	1.008	0.181
				OR10	0.986	0.815	1.192	0.881
	Renal	Lag 0	Lag 0	OR01	0.997	0.893	1.114	0.960
				OR10	1.062	0.737	1.528	0.748
			Lag 1	OR01	0.971	0.853	1.104	0.650
				OR10	1.107	0.773	1.585	0.579
			Lag 2	OR01	0.995	0.938	1.056	0.880
				OR10	1.067	0.741	1.537	0.727
			Lag 3	OR01	1.093	0.975	1.225	0.127
				OR10	1.116	0.781	1.595	0.545

		Lag 1	Lag 0	OR01	1.006	0.902	1.122	0.917
				OR10	1.159	0.815	1.649	0.412
			Lag 1	OR01	0.958	0.835	1.099	0.542
				OR10	1.146	0.808	1.627	0.444
			Lag 2	OR01	1.001	0.948	1.056	0.978
				OR10	1.192	0.842	1.686	0.322
			Lag 3	OR01	1.070	0.951	1.205	0.260
				OR10	1.133	0.800	1.605	0.480
		Lag 2	Lag 0	OR01	0.989	0.890	1.100	0.844
				OR10	1.104	0.796	1.533	0.552
			Lag 1	OR01	0.947	0.827	1.085	0.432
				OR10	1.118	0.808	1.548	0.501
			Lag 2	OR01	1.001	0.948	1.056	0.983
				OR10	1.178	0.852	1.628	0.322
			Lag 3	OR01	1.136	0.995	1.296	0.058
				OR10	1.176	0.850	1.627	0.328
		Lag 3	Lag 0	OR01	0.967	0.863	1.084	0.566
				OR10	1.226	0.891	1.688	0.210
			Lag 1	OR01	0.968	0.852	1.100	0.616
				OR10	1.324	0.969	1.810	0.078
			Lag 2	OR01	0.996	0.938	1.057	0.898
				OR10	1.295	0.946	1.772	0.106
			Lag 3	OR01	1.150	1.012	1.307	**0.032**
				OR10	1.382	1.009	1.893	**0.044**
	Respiratory	Lag 0	Lag 0	OR01	1.023	0.992	1.055	0.149
				OR10	1.191	0.895	1.587	0.231
			Lag 1	OR01	1.005	0.982	1.029	0.682
				OR10	1.180	0.885	1.574	0.259
			Lag 2	OR01	0.996	0.966	1.026	0.783
				OR10	1.155	0.866	1.540	0.327
			Lag 3	OR01	1.006	0.973	1.040	0.745
				OR10	1.157	0.867	1.545	0.322
		Lag 1	Lag 0	OR01	1.021	0.990	1.052	0.190
				OR10	1.143	0.856	1.525	0.366
			Lag 1	OR01	1.001	0.976	1.026	0.942
				OR10	1.093	0.819	1.458	0.546
			Lag 2	OR01	0.998	0.968	1.028	0.875
				OR10	1.176	0.884	1.564	0.266
			Lag 3	OR01	1.003	0.970	1.037	0.871
				OR10	1.097	0.822	1.463	0.531
		Lag 2	Lag 0	OR01	1.021	0.990	1.052	0.192
				OR10	0.955	0.701	1.300	0.768

			Lag 1	OR01	1.001	0.977	1.026	0.922
				OR10	0.912	0.668	1.243	0.559
			Lag 2	OR01	0.997	0.968	1.027	0.836
				OR10	0.977	0.717	1.330	0.881
			Lag 3	OR01	1.001	0.968	1.036	0.945
				OR10	0.885	0.645	1.214	0.450
		Lag 3	Lag 0	OR01	1.020	0.990	1.052	0.198
				OR10	1.052	0.783	1.415	0.736
			Lag 1	OR01	1.004	0.981	1.028	0.716
				OR10	1.116	0.833	1.496	0.462
			Lag 2	OR01	0.994	0.964	1.025	0.705
				OR10	1.050	0.783	1.409	0.744
			Lag 3	OR01	0.999	0.965	1.034	0.972
				OR10	0.967	0.713	1.310	0.828
Spanish, preferred language	All-natural	Lag 0	Lag 0	OR01	1.000	0.999	1.001	0.858
				OR10	1.030	1.021	1.039	**0.000**
			Lag 1	OR01	1.000	0.999	1.002	0.516
				OR10	1.030	1.021	1.039	**0.000**
			Lag 2	OR01	0.999	0.998	1.000	0.103
				OR10	1.030	1.021	1.039	**0.000**
			Lag 3	OR01	0.999	0.998	1.000	0.218
				OR10	1.031	1.022	1.040	**0.000**
		Lag 1	Lag 0	OR01	1.000	0.999	1.001	0.836
				OR10	1.028	1.019	1.037	**0.000**
			Lag 1	OR01	1.000	0.999	1.002	0.440
				OR10	1.028	1.020	1.037	**0.000**
			Lag 2	OR01	0.999	0.998	1.000	0.125
				OR10	1.029	1.020	1.038	**0.000**
			Lag 3	OR01	0.999	0.998	1.000	0.160
				OR10	1.028	1.019	1.036	**0.000**
		Lag 2	Lag 0	OR01	1.000	0.999	1.002	0.652
				OR10	1.025	1.017	1.034	**0.000**
			Lag 1	OR01	1.000	0.999	1.002	0.456
				OR10	1.025	1.016	1.033	**0.000**
			Lag 2	OR01	0.999	0.998	1.000	0.170
				OR10	1.026	1.017	1.035	**0.000**
			Lag 3	OR01	0.999	0.998	1.000	0.157
				OR10	1.024	1.016	1.033	**0.000**
		Lag 3	Lag 0	OR01	1.000	0.999	1.001	0.922
				OR10	1.014	1.006	1.023	**0.001**
			Lag 1	OR01	1.000	0.999	1.002	0.569
				OR10	1.016	1.007	1.024	**0.000**

			Lag 2	OR01	0.999	0.998	1.000	0.197
				OR10	1.018	1.009	1.026	**0.000**
			Lag 3	OR01	0.999	0.998	1.001	0.295
				OR10	1.017	1.009	1.026	**0.000**
	Cardiovascular	Lag 0	Lag 0	OR01	1.021	0.988	1.056	0.216
				OR10	0.930	0.760	1.138	0.479
			Lag 1	OR01	1.035	1.007	1.064	**0.016**
				OR10	0.917	0.750	1.122	0.402
			Lag 2	OR01	1.029	0.991	1.069	0.130
				OR10	0.935	0.765	1.143	0.512
			Lag 3	OR01	1.020	0.993	1.047	0.149
				OR10	0.929	0.759	1.137	0.475
		Lag 1	Lag 0	OR01	1.022	0.988	1.057	0.204
				OR10	1.055	0.873	1.276	0.581
			Lag 1	OR01	1.035	1.007	1.065	**0.015**
				OR10	1.039	0.860	1.256	0.693
			Lag 2	OR01	1.027	0.989	1.067	0.169
				OR10	1.047	0.867	1.263	0.634
			Lag 3	OR01	1.022	0.995	1.050	0.107
				OR10	1.072	0.887	1.295	0.473
		Lag 2	Lag 0	OR01	1.023	0.989	1.057	0.188
				OR10	0.909	0.750	1.102	0.332
			Lag 1	OR01	1.036	1.007	1.065	**0.015**
				OR10	0.897	0.740	1.086	0.266
			Lag 2	OR01	1.030	0.991	1.070	0.129
				OR10	0.902	0.745	1.092	0.289
			Lag 3	OR01	1.019	0.992	1.047	0.160
				OR10	0.896	0.738	1.087	0.265
		Lag 3	Lag 0	OR01	1.021	0.987	1.055	0.229
				OR10	0.868	0.715	1.055	0.155
			Lag 1	OR01	1.035	1.007	1.065	**0.015**
				OR10	0.868	0.716	1.052	0.149
			Lag 2	OR01	1.032	0.993	1.073	0.105
				OR10	0.881	0.727	1.069	0.199
			Lag 3	OR01	1.017	0.990	1.044	0.216
				OR10	0.851	0.700	1.034	0.104
	Cerebrovascular	Lag 0	Lag 0	OR01	1.011	0.986	1.036	0.396
				OR10	1.069	0.919	1.243	0.390
			Lag 1	OR01	1.011	0.990	1.032	0.314
				OR10	1.074	0.923	1.249	0.357
			Lag 2	OR01	1.020	0.996	1.045	0.105
				OR10	1.048	0.901	1.219	0.543

			Lag 3	OR01	1.005	0.986	1.024	0.620
				OR10	1.025	0.881	1.192	0.749
		Lag 1	Lag 0	OR01	1.007	0.982	1.033	0.573
				OR10	0.924	0.792	1.079	0.318
			Lag 1	OR01	1.010	0.989	1.031	0.353
				OR10	0.942	0.808	1.100	0.451
			Lag 2	OR01	1.022	0.998	1.047	0.072
				OR10	0.942	0.807	1.099	0.447
			Lag 3	OR01	1.005	0.986	1.025	0.590
				OR10	0.907	0.777	1.059	0.217
		Lag 2	Lag 0	OR01	1.008	0.983	1.033	0.551
				OR10	0.948	0.813	1.104	0.490
			Lag 1	OR01	1.011	0.990	1.033	0.313
				OR10	0.967	0.831	1.126	0.670
			Lag 2	OR01	1.024	1.000	1.049	0.053
				OR10	0.972	0.835	1.132	0.720
			Lag 3	OR01	1.005	0.986	1.025	0.583
				OR10	0.930	0.798	1.085	0.357
		Lag 3	Lag 0	OR01	1.005	0.981	1.031	0.673
				OR10	0.945	0.813	1.097	0.457
			Lag 1	OR01	1.011	0.990	1.032	0.325
				OR10	0.981	0.846	1.137	0.798
			Lag 2	OR01	1.022	0.996	1.048	0.101
				OR10	0.968	0.836	1.122	0.668
			Lag 3	OR01	1.006	0.987	1.026	0.514
				OR10	0.953	0.821	1.107	0.532
	Renal	Lag 0	Lag 0	OR01	0.872	0.774	0.983	**0.025**
				OR10	0.968	0.771	1.216	0.781
			Lag 1	OR01	0.945	0.868	1.029	0.191
				OR10	0.985	0.785	1.236	0.895
			Lag 2	OR01	1.005	0.976	1.034	0.744
				OR10	0.959	0.764	1.204	0.719
			Lag 3	OR01	0.992	0.953	1.032	0.676
				OR10	0.946	0.754	1.186	0.630
		Lag 1	Lag 0	OR01	0.883	0.786	0.992	**0.036**
				OR10	1.009	0.807	1.260	0.940
			Lag 1	OR01	0.908	0.825	1.000	**0.049**
				OR10	0.966	0.773	1.207	0.762
			Lag 2	OR01	1.004	0.974	1.034	0.815
				OR10	0.964	0.771	1.206	0.748
			Lag 3	OR01	0.992	0.953	1.032	0.683
				OR10	0.971	0.778	1.213	0.796

		Lag 2	Lag 0	OR01	0.878	0.784	0.983	**0.024**
				OR10	0.990	0.793	1.235	0.927
			Lag 1	OR01	0.917	0.836	1.005	0.065
				OR10	0.967	0.775	1.208	0.769
			Lag 2	OR01	1.006	0.977	1.035	0.689
				OR10	0.991	0.794	1.238	0.939
			Lag 3	OR01	0.994	0.956	1.034	0.775
				OR10	0.983	0.787	1.228	0.879
		Lag 3	Lag 0	OR01	0.886	0.792	0.991	**0.034**
				OR10	0.957	0.769	1.191	0.694
			Lag 1	OR01	0.932	0.855	1.017	0.114
				OR10	0.943	0.758	1.172	0.595
			Lag 2	OR01	1.002	0.972	1.033	0.879
				OR10	0.926	0.744	1.153	0.493
			Lag 3	OR01	0.995	0.957	1.035	0.806
				OR10	0.949	0.761	1.184	0.642
	Respiratory	Lag 0	Lag 0	OR01	0.958	0.903	1.017	0.161
				OR10	1.237	0.994	1.539	0.056
			Lag 1	OR01	0.928	0.867	0.993	**0.030**
				OR10	1.260	1.014	1.565	**0.037**
			Lag 2	OR01	0.988	0.936	1.043	0.665
				OR10	1.244	1.002	1.546	**0.048**
			Lag 3	OR01	0.914	0.844	0.990	**0.028**
				OR10	1.279	1.031	1.587	**0.025**
		Lag 1	Lag 0	OR01	0.947	0.888	1.011	0.103
				OR10	1.115	0.898	1.385	0.323
			Lag 1	OR01	0.929	0.868	0.995	**0.035**
				OR10	1.168	0.942	1.450	0.158
			Lag 2	OR01	0.986	0.933	1.041	0.606
				OR10	1.130	0.910	1.404	0.269
			Lag 3	OR01	0.906	0.835	0.983	**0.018**
				OR10	1.153	0.929	1.432	0.196
		Lag 2	Lag 0	OR01	0.945	0.883	1.011	0.099
				OR10	1.050	0.840	1.313	0.669
			Lag 1	OR01	0.928	0.865	0.996	**0.039**
				OR10	1.095	0.877	1.368	0.423
			Lag 2	OR01	0.992	0.941	1.046	0.761
				OR10	1.085	0.867	1.357	0.477
			Lag 3	OR01	0.909	0.839	0.985	**0.020**
				OR10	1.084	0.865	1.357	0.484
		Lag 3	Lag 0	OR01	0.964	0.913	1.019	0.194
				OR10	0.859	0.687	1.074	0.182

			Lag 1	OR01	0.936	0.877	1.000	0.051
				OR10	0.890	0.714	1.110	0.301
			Lag 2	OR01	0.994	0.944	1.047	0.823
				OR10	0.883	0.707	1.101	0.268
			Lag 3	OR01	0.919	0.851	0.991	**0.028**
				OR10	0.886	0.711	1.105	0.283
Lesser poverty (< 25% households living under poverty)	All-natural	Lag 0	Lag 0	OR01	1.000	1.000	1.000	0.966
				OR10	1.023	1.020	1.026	**0.000**
			Lag 1	OR01	1.000	0.999	1.000	0.401
				OR10	1.023	1.020	1.027	**0.000**
			Lag 2	OR01	0.999	0.999	1.000	**0.000**
				OR10	1.025	1.021	1.028	**0.000**
			Lag 3	OR01	0.999	0.998	0.999	**0.000**
				OR10	1.023	1.020	1.026	**0.000**
		Lag 1	Lag 0	OR01	1.000	1.000	1.000	0.733
				OR10	1.024	1.021	1.027	**0.000**
			Lag 1	OR01	1.000	0.999	1.000	0.532
				OR10	1.025	1.022	1.028	**0.000**
			Lag 2	OR01	0.999	0.999	1.000	**0.000**
				OR10	1.025	1.022	1.028	**0.000**
			Lag 3	OR01	0.999	0.998	0.999	**0.000**
				OR10	1.024	1.021	1.027	**0.000**
		Lag 2	Lag 0	OR01	1.000	1.000	1.000	0.875
				OR10	1.018	1.015	1.021	**0.000**
			Lag 1	OR01	1.000	1.000	1.000	0.632
				OR10	1.019	1.016	1.022	**0.000**
			Lag 2	OR01	0.999	0.999	1.000	**0.000**
				OR10	1.020	1.017	1.023	**0.000**
			Lag 3	OR01	0.999	0.999	0.999	**0.000**
				OR10	1.019	1.016	1.023	**0.000**
		Lag 3	Lag 0	OR01	1.000	1.000	1.000	0.890
				OR10	1.013	1.010	1.017	**0.000**
			Lag 1	OR01	1.000	0.999	1.000	0.322
				OR10	1.014	1.011	1.017	**0.000**
			Lag 2	OR01	0.999	0.999	1.000	**0.000**
				OR10	1.016	1.013	1.019	**0.000**
			Lag 3	OR01	0.999	0.999	0.999	**0.000**
				OR10	1.015	1.012	1.018	**0.000**
	Cardiovascular	Lag 0	Lag 0	OR01	1.003	0.993	1.012	0.577
				OR10	1.045	0.979	1.116	0.184
			Lag 1	OR01	1.003	0.994	1.012	0.519
				OR10	1.042	0.976	1.112	0.221

			Lag 2	OR01	0.998	0.988	1.007	0.608
				OR10	1.046	0.979	1.117	0.181
			Lag 3	OR01	0.998	0.989	1.007	0.633
				OR10	1.051	0.985	1.122	0.135
		Lag 1	Lag 0	OR01	1.002	0.993	1.011	0.684
				OR10	1.075	1.008	1.146	**0.028**
			Lag 1	OR01	1.003	0.994	1.012	0.473
				OR10	1.078	1.010	1.149	**0.023**
			Lag 2	OR01	0.997	0.987	1.006	0.493
				OR10	1.073	1.006	1.145	**0.032**
			Lag 3	OR01	0.998	0.988	1.007	0.603
				OR10	1.083	1.016	1.155	**0.015**
		Lag 2	Lag 0	OR01	1.001	0.992	1.010	0.812
				OR10	1.049	0.984	1.117	0.143
			Lag 1	OR01	1.004	0.996	1.013	0.326
				OR10	1.063	0.998	1.133	0.058
			Lag 2	OR01	0.998	0.989	1.007	0.678
				OR10	1.063	0.997	1.133	0.061
			Lag 3	OR01	0.998	0.989	1.007	0.685
				OR10	1.065	1.000	1.135	0.052
		Lag 3	Lag 0	OR01	1.001	0.992	1.010	0.795
				OR10	1.030	0.966	1.098	0.365
			Lag 1	OR01	1.004	0.995	1.013	0.426
				OR10	1.042	0.978	1.111	0.204
			Lag 2	OR01	0.998	0.989	1.008	0.727
				OR10	1.047	0.982	1.116	0.160
			Lag 3	OR01	0.997	0.988	1.007	0.596
				OR10	1.045	0.980	1.114	0.182
	Cerebrovascular	Lag 0	Lag 0	OR01	1.004	0.998	1.011	0.205
				OR10	1.005	0.951	1.062	0.855
			Lag 1	OR01	1.001	0.995	1.008	0.677
				OR10	1.006	0.953	1.063	0.827
			Lag 2	OR01	0.998	0.992	1.005	0.659
				OR10	1.004	0.951	1.061	0.877
			Lag 3	OR01	0.994	0.987	1.002	0.138
				OR10	1.001	0.948	1.058	0.965
		Lag 1	Lag 0	OR01	1.004	0.997	1.010	0.253
				OR10	1.018	0.965	1.075	0.509
			Lag 1	OR01	1.001	0.995	1.008	0.708
				OR10	1.022	0.968	1.079	0.431
			Lag 2	OR01	0.999	0.992	1.006	0.699
				OR10	1.024	0.970	1.081	0.399

			Lag 3	OR01	0.995	0.988	1.003	0.200
				OR10	1.026	0.971	1.083	0.361
		Lag 2	Lag 0	OR01	1.004	0.998	1.011	0.200
				OR10	1.051	0.996	1.108	0.070
			Lag 1	OR01	1.001	0.994	1.008	0.757
				OR10	1.051	0.996	1.108	0.069
			Lag 2	OR01	0.999	0.992	1.006	0.731
				OR10	1.055	1.000	1.113	0.052
			Lag 3	OR01	0.994	0.987	1.002	0.136
				OR10	1.048	0.994	1.106	0.082
		Lag 3	Lag 0	OR01	1.004	0.997	1.010	0.241
				OR10	1.020	0.967	1.076	0.476
			Lag 1	OR01	1.001	0.995	1.008	0.701
				OR10	1.024	0.971	1.080	0.379
			Lag 2	OR01	0.998	0.991	1.005	0.528
				OR10	1.022	0.969	1.078	0.427
			Lag 3	OR01	0.993	0.986	1.001	0.095
				OR10	1.018	0.965	1.074	0.515
	Renal	Lag 0	Lag 0	OR01	0.986	0.965	1.008	0.202
				OR10	1.087	0.996	1.186	0.062
			Lag 1	OR01	0.990	0.972	1.008	0.286
				OR10	1.091	0.999	1.191	0.052
			Lag 2	OR01	1.007	0.993	1.021	0.351
				OR10	1.086	0.996	1.186	0.063
			Lag 3	OR01	1.000	0.984	1.017	0.966
				OR10	1.079	0.989	1.178	0.087
		Lag 1	Lag 0	OR01	0.983	0.961	1.005	0.133
				OR10	1.113	1.023	1.211	**0.013**
			Lag 1	OR01	0.989	0.971	1.008	0.260
				OR10	1.121	1.029	1.220	**0.009**
			Lag 2	OR01	1.008	0.994	1.022	0.246
				OR10	1.132	1.040	1.232	**0.004**
			Lag 3	OR01	1.000	0.983	1.016	0.956
				OR10	1.107	1.017	1.205	**0.019**
		Lag 2	Lag 0	OR01	0.987	0.966	1.008	0.227
				OR10	1.087	0.999	1.183	0.052
			Lag 1	OR01	0.988	0.970	1.007	0.229
				OR10	1.085	0.996	1.180	0.060
			Lag 2	OR01	1.007	0.993	1.021	0.341
				OR10	1.089	1.000	1.185	**0.049**
			Lag 3	OR01	0.999	0.982	1.016	0.930
				OR10	1.079	0.991	1.175	0.078

		Lag 3	Lag 0	OR01	0.988	0.968	1.009	0.272
				OR10	1.038	0.953	1.129	0.392
			Lag 1	OR01	0.990	0.972	1.008	0.286
				OR10	1.036	0.952	1.128	0.407
			Lag 2	OR01	1.006	0.992	1.020	0.435
				OR10	1.029	0.946	1.120	0.502
			Lag 3	OR01	1.001	0.984	1.018	0.901
				OR10	1.033	0.949	1.125	0.449
	Respiratory	Lag 0	Lag 0	OR01	0.993	0.979	1.007	0.309
				OR10	0.958	0.884	1.039	0.298
			Lag 1	OR01	1.003	0.994	1.012	0.534
				OR10	0.956	0.882	1.036	0.270
			Lag 2	OR01	1.005	0.994	1.017	0.372
				OR10	0.963	0.889	1.044	0.363
			Lag 3	OR01	1.002	0.990	1.014	0.728
				OR10	0.946	0.873	1.025	0.176
		Lag 1	Lag 0	OR01	0.993	0.979	1.007	0.301
				OR10	1.001	0.925	1.083	0.985
			Lag 1	OR01	1.003	0.994	1.012	0.516
				OR10	1.000	0.924	1.082	0.996
			Lag 2	OR01	1.005	0.994	1.017	0.371
				OR10	1.006	0.930	1.088	0.885
			Lag 3	OR01	1.003	0.991	1.015	0.642
				OR10	0.992	0.916	1.073	0.837
		Lag 2	Lag 0	OR01	0.990	0.976	1.005	0.194
				OR10	0.998	0.922	1.080	0.959
			Lag 1	OR01	1.002	0.992	1.011	0.736
				OR10	0.999	0.923	1.080	0.971
			Lag 2	OR01	1.005	0.994	1.017	0.374
				OR10	1.013	0.936	1.096	0.748
			Lag 3	OR01	1.003	0.991	1.015	0.596
				OR10	1.003	0.927	1.085	0.938
		Lag 3	Lag 0	OR01	0.993	0.979	1.007	0.326
				OR10	1.027	0.951	1.110	0.489
			Lag 1	OR01	1.002	0.992	1.012	0.683
				OR10	1.026	0.951	1.107	0.514
			Lag 2	OR01	1.005	0.994	1.017	0.355
				OR10	1.032	0.956	1.114	0.420
			Lag 3	OR01	1.003	0.991	1.016	0.588
				OR10	1.023	0.947	1.105	0.561
Higher poverty (> [illegible]	All-natural	Lag 0	Lag 0	OR01	1.000	0.999	1.001	0.618
				OR10	1.026	1.019	1.033	**0.000**

25% households living under poverty)			Lag 1	OR01	1.000	0.999	1.001	0.634
				OR10	1.027	1.021	1.034	**0.000**
			Lag 2	OR01	1.000	0.999	1.001	0.765
				OR10	1.027	1.020	1.033	**0.000**
			Lag 3	OR01	1.000	0.999	1.001	0.912
				OR10	1.027	1.021	1.034	**0.000**
		Lag 1	Lag 0	OR01	1.000	0.999	1.001	0.822
				OR10	1.019	1.013	1.026	**0.000**
			Lag 1	OR01	1.000	0.999	1.001	0.698
				OR10	1.022	1.015	1.028	**0.000**
			Lag 2	OR01	1.000	0.999	1.001	0.973
				OR10	1.022	1.016	1.029	**0.000**
			Lag 3	OR01	1.000	0.999	1.001	0.741
				OR10	1.021	1.014	1.027	**0.000**
		Lag 2	Lag 0	OR01	1.000	0.999	1.001	0.705
				OR10	1.020	1.013	1.026	**0.000**
			Lag 1	OR01	1.000	0.999	1.001	0.676
				OR10	1.021	1.015	1.028	**0.000**
			Lag 2	OR01	1.000	0.999	1.001	0.811
				OR10	1.023	1.017	1.030	**0.000**
			Lag 3	OR01	1.000	0.999	1.001	0.634
				OR10	1.021	1.014	1.027	**0.000**
		Lag 3	Lag 0	OR01	1.000	0.999	1.001	0.670
				OR10	1.014	1.008	1.021	**0.000**
			Lag 1	OR01	1.000	0.999	1.001	0.537
				OR10	1.015	1.009	1.022	**0.000**
			Lag 2	OR01	1.000	0.999	1.001	0.785
				OR10	1.017	1.011	1.024	**0.000**
			Lag 3	OR01	1.000	0.999	1.001	0.692
				OR10	1.015	1.009	1.022	**0.000**
	Cardiovascular	Lag 0	Lag 0	OR01	0.997	0.975	1.019	0.761
				OR10	0.936	0.807	1.086	0.386
			Lag 1	OR01	1.025	1.002	1.049	**0.036**
				OR10	0.958	0.826	1.111	0.571
			Lag 2	OR01	1.012	0.992	1.032	0.252
				OR10	0.951	0.820	1.102	0.500
			Lag 3	OR01	1.004	0.983	1.026	0.680
				OR10	0.971	0.839	1.124	0.693
		Lag 1	Lag 0	OR01	0.997	0.976	1.019	0.812
				OR10	0.975	0.845	1.125	0.729
			Lag 1	OR01	1.024	1.000	1.048	**0.047**
				OR10	0.979	0.848	1.130	0.775

			Lag 2	OR01	1.011	0.991	1.032	0.278
				OR10	0.973	0.842	1.124	0.711
			Lag 3	OR01	1.002	0.981	1.024	0.855
				OR10	0.984	0.853	1.135	0.828
		Lag 2	Lag 0	OR01	0.997	0.976	1.019	0.805
				OR10	0.947	0.823	1.090	0.450
			Lag 1	OR01	1.025	1.001	1.049	**0.044**
				OR10	0.960	0.835	1.104	0.567
			Lag 2	OR01	1.012	0.991	1.032	0.259
				OR10	0.955	0.829	1.099	0.517
			Lag 3	OR01	1.002	0.980	1.024	0.873
				OR10	0.957	0.832	1.102	0.544
		Lag 3	Lag 0	OR01	0.999	0.978	1.020	0.899
				OR10	0.967	0.843	1.108	0.627
			Lag 1	OR01	1.027	1.002	1.052	**0.031**
				OR10	0.978	0.854	1.120	0.746
			Lag 2	OR01	1.014	0.994	1.035	0.166
				OR10	0.983	0.858	1.127	0.806
			Lag 3	OR01	1.001	0.979	1.023	0.919
				OR10	0.969	0.845	1.110	0.648
	Cerebrovascular	Lag 0	Lag 0	OR01	0.999	0.984	1.016	0.950
				OR10	1.117	0.993	1.256	0.067
			Lag 1	OR01	0.998	0.980	1.016	0.831
				OR10	1.118	0.993	1.259	0.065
			Lag 2	OR01	0.997	0.981	1.014	0.725
				OR10	1.099	0.976	1.236	0.118
			Lag 3	OR01	1.003	0.983	1.023	0.789
				OR10	1.099	0.977	1.237	0.115
		Lag 1	Lag 0	OR01	1.000	0.984	1.016	0.953
				OR10	1.096	0.974	1.234	0.127
			Lag 1	OR01	0.996	0.978	1.015	0.682
				OR10	1.082	0.961	1.219	0.194
			Lag 2	OR01	0.997	0.981	1.014	0.749
				OR10	1.082	0.960	1.219	0.198
			Lag 3	OR01	1.000	0.980	1.021	0.997
				OR10	1.064	0.945	1.199	0.303
		Lag 2	Lag 0	OR01	0.998	0.982	1.014	0.815
				OR10	0.985	0.876	1.107	0.798
			Lag 1	OR01	0.995	0.976	1.014	0.597
				OR10	0.976	0.867	1.099	0.691
			Lag 2	OR01	0.998	0.982	1.015	0.851
				OR10	0.990	0.879	1.115	0.870

		Lag 3	OR01	1.003	0.983	1.024	0.759
			OR10	0.983	0.872	1.108	0.779
	Lag 3	Lag 0	OR01	0.998	0.981	1.014	0.766
			OR10	0.989	0.879	1.112	0.852
		Lag 1	OR01	0.994	0.974	1.013	0.517
			OR10	0.981	0.872	1.104	0.753
		Lag 2	OR01	0.998	0.982	1.015	0.853
			OR10	0.997	0.886	1.122	0.966
		Lag 3	OR01	1.005	0.985	1.025	0.626
			OR10	1.001	0.889	1.128	0.981
Renal	Lag 0	Lag 0	OR01	1.008	0.978	1.039	0.597
			OR10	1.100	0.922	1.312	0.292
		Lag 1	OR01	1.003	0.959	1.050	0.892
			OR10	1.136	0.952	1.356	0.158
		Lag 2	OR01	1.007	0.975	1.041	0.654
			OR10	1.092	0.914	1.305	0.331
		Lag 3	OR01	0.992	0.959	1.026	0.634
			OR10	1.110	0.930	1.323	0.248
	Lag 1	Lag 0	OR01	1.009	0.979	1.040	0.547
			OR10	1.112	0.934	1.323	0.234
		Lag 1	OR01	1.005	0.962	1.050	0.825
			OR10	1.152	0.968	1.370	0.111
		Lag 2	OR01	1.009	0.978	1.042	0.564
			OR10	1.112	0.933	1.325	0.235
		Lag 3	OR01	0.991	0.958	1.026	0.613
			OR10	1.117	0.939	1.330	0.212
	Lag 2	Lag 0	OR01	1.016	0.986	1.048	0.299
			OR10	1.012	0.850	1.204	0.897
		Lag 1	OR01	1.011	0.968	1.055	0.636
			OR10	1.030	0.866	1.225	0.738
		Lag 2	OR01	1.011	0.979	1.044	0.516
			OR10	0.993	0.833	1.185	0.940
		Lag 3	OR01	0.985	0.946	1.024	0.445
			OR10	0.975	0.817	1.164	0.781
	Lag 3	Lag 0	OR01	1.020	0.989	1.052	0.206
			OR10	1.107	0.933	1.312	0.244
		Lag 1	OR01	1.004	0.962	1.047	0.860
			OR10	1.083	0.914	1.283	0.356
		Lag 2	OR01	1.017	0.985	1.049	0.301
			OR10	1.087	0.916	1.290	0.341
		Lag 3	OR01	0.987	0.950	1.025	0.499
			OR10	1.048	0.882	1.245	0.594

	Respiratory	Lag 0	Lag 0	OR01	1.004	0.983	1.026	0.710
				OR10	1.019	0.870	1.194	0.813
			Lag 1	OR01	0.987	0.957	1.018	0.407
				OR10	0.999	0.851	1.172	0.985
			Lag 2	OR01	1.004	0.978	1.031	0.758
				OR10	1.010	0.862	1.183	0.902
			Lag 3	OR01	0.997	0.976	1.018	0.766
				OR10	0.996	0.849	1.168	0.961
		Lag 1	Lag 0	OR01	1.006	0.985	1.027	0.576
				OR10	1.068	0.913	1.249	0.413
			Lag 1	OR01	0.981	0.948	1.016	0.282
				OR10	1.013	0.865	1.186	0.873
			Lag 2	OR01	1.006	0.980	1.033	0.650
				OR10	1.052	0.900	1.230	0.525
			Lag 3	OR01	0.996	0.975	1.017	0.704
				OR10	1.011	0.863	1.185	0.891
		Lag 2	Lag 0	OR01	1.002	0.980	1.024	0.878
				OR10	1.002	0.858	1.172	0.977
			Lag 1	OR01	0.984	0.950	1.019	0.359
				OR10	1.005	0.860	1.173	0.954
			Lag 2	OR01	1.005	0.979	1.032	0.705
				OR10	1.015	0.869	1.186	0.848
			Lag 3	OR01	0.997	0.977	1.018	0.790
				OR10	0.999	0.854	1.169	0.990
		Lag 3	Lag 0	OR01	1.004	0.982	1.025	0.734
				OR10	1.090	0.942	1.261	0.246
			Lag 1	OR01	0.989	0.959	1.020	0.488
				OR10	1.087	0.940	1.257	0.261
			Lag 2	OR01	1.007	0.981	1.034	0.598
				OR10	1.097	0.948	1.268	0.214
			Lag 3	OR01	0.997	0.977	1.018	0.805
				OR10	1.074	0.927	1.244	0.342
White	All-natural	Lag 0	Lag 0	OR01	1.000	0.999	1.000	0.075
				OR10	1.020	1.016	1.024	**0.000**
			Lag 1	OR01	1.000	0.999	1.000	0.073
				OR10	1.021	1.017	1.025	**0.000**
			Lag 2	OR01	0.999	0.998	0.999	**0.000**
				OR10	1.022	1.018	1.026	**0.000**
			Lag 3	OR01	0.999	0.998	0.999	**0.000**
				OR10	1.020	1.016	1.024	**0.000**
		Lag 1	Lag 0	OR01	0.999	0.999	1.000	0.051
				OR10	1.021	1.017	1.025	**0.000**

			Lag 1	OR01	1.000	0.999	1.000	0.195
				OR10	1.022	1.018	1.026	**0.000**
			Lag 2	OR01	0.999	0.998	0.999	**0.000**
				OR10	1.022	1.018	1.026	**0.000**
			Lag 3	OR01	0.999	0.998	0.999	**0.000**
				OR10	1.021	1.017	1.025	**0.000**
		Lag 2	Lag 0	OR01	1.000	0.999	1.000	0.078
				OR10	1.016	1.012	1.020	**0.000**
			Lag 1	OR01	1.000	0.999	1.000	0.224
				OR10	1.018	1.014	1.022	**0.000**
			Lag 2	OR01	0.999	0.999	1.000	**0.001**
				OR10	1.019	1.015	1.023	**0.000**
			Lag 3	OR01	0.999	0.998	1.000	**0.000**
				OR10	1.018	1.014	1.022	**0.000**
		Lag 3	Lag 0	OR01	0.999	0.999	1.000	0.059
				OR10	1.013	1.010	1.017	**0.000**
			Lag 1	OR01	0.999	0.999	1.000	0.056
				OR10	1.014	1.010	1.018	**0.000**
			Lag 2	OR01	0.999	0.999	1.000	**0.001**
				OR10	1.016	1.012	1.020	**0.000**
			Lag 3	OR01	0.999	0.998	0.999	**0.000**
				OR10	1.015	1.011	1.018	**0.000**
	Cardiovascular	Lag 0	Lag 0	OR01	1.003	0.991	1.015	0.640
				OR10	1.049	0.968	1.137	0.241
			Lag 1	OR01	1.001	0.990	1.012	0.833
				OR10	1.050	0.969	1.138	0.233
			Lag 2	OR01	1.004	0.992	1.015	0.532
				OR10	1.041	0.961	1.129	0.323
			Lag 3	OR01	1.000	0.989	1.011	0.966
				OR10	1.049	0.969	1.137	0.239
		Lag 1	Lag 0	OR01	1.003	0.991	1.015	0.623
				OR10	1.084	1.002	1.172	**0.044**
			Lag 1	OR01	1.002	0.992	1.013	0.649
				OR10	1.097	1.014	1.186	**0.022**
			Lag 2	OR01	1.004	0.993	1.015	0.484
				OR10	1.080	0.999	1.169	0.054
			Lag 3	OR01	1.000	0.989	1.011	0.979
				OR10	1.083	1.001	1.171	**0.046**
		Lag 2	Lag 0	OR01	1.002	0.990	1.014	0.758
				OR10	1.071	0.991	1.157	0.084
			Lag 1	OR01	1.002	0.991	1.013	0.716
				OR10	1.083	1.002	1.170	**0.043**

			Lag 2	OR01	1.005	0.993	1.016	0.423
				OR10	1.081	1.000	1.168	**0.049**
			Lag 3	OR01	1.001	0.990	1.012	0.844
				OR10	1.085	1.005	1.173	**0.037**
		Lag 3	Lag 0	OR01	1.001	0.989	1.014	0.865
				OR10	1.037	0.959	1.121	0.364
			Lag 1	OR01	1.002	0.991	1.013	0.786
				OR10	1.054	0.976	1.139	0.182
			Lag 2	OR01	1.004	0.993	1.015	0.461
				OR10	1.052	0.973	1.137	0.199
			Lag 3	OR01	1.000	0.989	1.011	0.961
				OR10	1.052	0.974	1.137	0.198
	Cerebrovascular	Lag 0	Lag 0	OR01	1.005	0.995	1.016	0.297
				OR10	1.039	0.969	1.113	0.283
			Lag 1	OR01	0.997	0.987	1.007	0.494
				OR10	1.035	0.966	1.109	0.324
			Lag 2	OR01	1.000	0.991	1.010	0.926
				OR10	1.036	0.967	1.110	0.317
			Lag 3	OR01	1.000	0.990	1.010	0.988
				OR10	1.042	0.973	1.117	0.237
		Lag 1	Lag 0	OR01	1.005	0.995	1.015	0.364
				OR10	1.046	0.976	1.120	0.204
			Lag 1	OR01	0.995	0.984	1.005	0.328
				OR10	1.040	0.971	1.113	0.269
			Lag 2	OR01	1.000	0.990	1.010	0.986
				OR10	1.046	0.976	1.120	0.204
			Lag 3	OR01	1.000	0.990	1.010	0.982
				OR10	1.053	0.983	1.129	0.140
		Lag 2	Lag 0	OR01	1.006	0.996	1.016	0.258
				OR10	1.067	0.997	1.141	0.062
			Lag 1	OR01	0.996	0.985	1.006	0.400
				OR10	1.058	0.989	1.133	0.102
			Lag 2	OR01	1.001	0.991	1.011	0.864
				OR10	1.068	0.997	1.143	0.060
			Lag 3	OR01	1.000	0.989	1.010	0.961
				OR10	1.068	0.998	1.143	0.059
		Lag 3	Lag 0	OR01	1.005	0.995	1.015	0.302
				OR10	0.997	0.931	1.068	0.937
			Lag 1	OR01	0.995	0.985	1.006	0.382
				OR10	0.991	0.925	1.061	0.792
			Lag 2	OR01	1.001	0.992	1.011	0.771
				OR10	1.004	0.938	1.075	0.909

			Lag 3	OR01	1.000	0.989	1.010	0.960
				OR10	1.002	0.936	1.073	0.961
	Renal	Lag 0	Lag 0	OR01	1.009	0.988	1.030	0.420
				OR10	1.073	0.963	1.195	0.200
			Lag 1	OR01	0.999	0.977	1.021	0.930
				OR10	1.095	0.982	1.220	0.102
			Lag 2	OR01	1.010	0.991	1.029	0.321
				OR10	1.068	0.959	1.190	0.232
			Lag 3	OR01	1.003	0.984	1.023	0.757
				OR10	1.067	0.958	1.188	0.241
		Lag 1	Lag 0	OR01	1.005	0.984	1.027	0.621
				OR10	1.136	1.024	1.260	**0.016**
			Lag 1	OR01	0.997	0.974	1.020	0.786
				OR10	1.155	1.041	1.281	**0.007**
			Lag 2	OR01	1.011	0.992	1.030	0.243
				OR10	1.150	1.036	1.275	**0.008**
			Lag 3	OR01	1.002	0.982	1.021	0.877
				OR10	1.126	1.014	1.250	**0.026**
		Lag 2	Lag 0	OR01	1.009	0.989	1.030	0.371
				OR10	1.019	0.917	1.132	0.726
			Lag 1	OR01	0.996	0.973	1.020	0.749
				OR10	1.024	0.921	1.138	0.664
			Lag 2	OR01	1.011	0.992	1.030	0.276
				OR10	1.018	0.916	1.131	0.742
			Lag 3	OR01	0.999	0.979	1.019	0.908
				OR10	0.998	0.897	1.109	0.968
		Lag 3	Lag 0	OR01	1.013	0.993	1.033	0.206
				OR10	1.018	0.916	1.132	0.743
			Lag 1	OR01	0.998	0.976	1.020	0.833
				OR10	1.011	0.909	1.124	0.842
			Lag 2	OR01	1.013	0.994	1.032	0.180
				OR10	1.009	0.907	1.122	0.867
			Lag 3	OR01	1.002	0.982	1.023	0.833
				OR10	0.994	0.894	1.105	0.911
	Respiratory	Lag 0	Lag 0	OR01	0.999	0.983	1.015	0.886
				OR10	0.934	0.845	1.032	0.181
			Lag 1	OR01	1.006	0.993	1.019	0.351
				OR10	0.930	0.841	1.028	0.154
			Lag 2	OR01	1.009	0.995	1.024	0.209
				OR10	0.933	0.844	1.031	0.173
			Lag 3	OR01	1.012	0.998	1.026	0.084
				OR10	0.907	0.820	1.003	0.058

		Lag 1	Lag 0	OR01	1.000	0.985	1.016	0.980
				OR10	1.017	0.922	1.123	0.730
			Lag 1	OR01	1.006	0.993	1.019	0.358
				OR10	1.002	0.908	1.106	0.966
			Lag 2	OR01	1.009	0.994	1.024	0.229
				OR10	1.003	0.910	1.107	0.949
			Lag 3	OR01	1.013	1.000	1.027	0.058
				OR10	0.983	0.891	1.085	0.738
		Lag 2	Lag 0	OR01	0.997	0.981	1.014	0.746
				OR10	0.999	0.906	1.102	0.988
			Lag 1	OR01	1.003	0.990	1.017	0.627
				OR10	0.990	0.898	1.092	0.845
			Lag 2	OR01	1.009	0.994	1.024	0.236
				OR10	1.003	0.910	1.107	0.948
			Lag 3	OR01	1.015	1.001	1.029	**0.036**
				OR10	0.998	0.905	1.102	0.976
		Lag 3	Lag 0	OR01	0.999	0.984	1.015	0.945
				OR10	1.120	1.021	1.230	**0.017**
			Lag 1	OR01	1.004	0.990	1.017	0.619
				OR10	1.110	1.012	1.217	**0.027**
			Lag 2	OR01	1.009	0.995	1.024	0.216
				OR10	1.115	1.017	1.224	**0.021**
			Lag 3	OR01	1.015	1.001	1.029	**0.038**
				OR10	1.110	1.011	1.218	**0.028**

Table S6 Attributable proportion due to interaction (AP) with upper and lower limits (UL and LL, respectively) for 95% confidence intervals (CIs) and p-values (bolded indicates significant p < 0.05) for the joint effects of wildfire smoke and extreme heat, 2011-2019, for all months. Separate models were run for combinations of lag 0-3 and each outcome of interest: all-natural cause, cardiovascular, cerebrovascular, renal, and respiratory morbidity.

OOI	Extreme Heat	Wildfire Smoke	AP	CI LL	CI UL	P-value
All-natural	Lag 0	Lag 0	0.001	-0.001	0.003	0.100
		Lag 1	-0.001	-0.003	0.001	0.165
		Lag 2	-0.003	-0.005	-0.001	**0.002**
		Lag 3	0.000	-0.002	0.002	0.402
	Lag 1	Lag 0	0.003	0.001	0.005	**0.000**
		Lag 1	-0.001	-0.003	0.001	0.110
		Lag 2	-0.002	-0.004	-0.001	**0.005**
		Lag 3	0.000	-0.002	0.002	0.340
	Lag 2	Lag 0	0.004	0.002	0.006	**0.000**
		Lag 1	-0.001	-0.003	0.000	0.080
		Lag 2	-0.004	-0.006	-0.003	**0.000**
		Lag 3	-0.001	-0.003	0.001	0.111
	Lag 3	Lag 0	0.005	0.003	0.007	**0.000**
		Lag 1	0.002	0.000	0.003	**0.025**
		Lag 2	-0.003	-0.005	-0.002	**0.000**
		Lag 3	-0.001	-0.002	0.001	0.217
Cardiovascular	Lag 0	Lag 0	0.000	-0.030	0.031	0.494
		Lag 1	0.005	-0.027	0.037	0.388
		Lag 2	-0.014	-0.066	0.039	0.305
		Lag 3	-0.030	-0.077	0.016	0.101
	Lag 1	Lag 0	0.027	-0.010	0.063	0.075
		Lag 1	0.004	-0.026	0.035	0.389
		Lag 2	0.031	-0.007	0.069	0.057
		Lag 3	-0.005	-0.041	0.030	0.384
	Lag 2	Lag 0	0.045	0.007	0.083	**0.011**
		Lag 1	-0.006	-0.038	0.025	0.346
		Lag 2	-0.009	-0.048	0.030	0.327
		Lag 3	-0.005	-0.036	0.026	0.375
	Lag 3	Lag 0	0.056	0.011	0.101	**0.008**
		Lag 1	0.002	-0.029	0.033	0.445
		Lag 2	-0.010	-0.047	0.027	0.304
		Lag 3	0.008	-0.024	0.040	0.315
Cerebrovascular	Lag 0	Lag 0	-0.006	-0.038	0.027	0.368
		Lag 1	-0.002	-0.033	0.030	0.458
		Lag 2	0.013	-0.029	0.054	0.278

		Lag 3	0.016	-0.025	0.057	0.224
	Lag 1	Lag 0	0.009	-0.022	0.040	0.287
		Lag 1	0.013	-0.014	0.039	0.181
		Lag 2	0.009	-0.025	0.043	0.299
		Lag 3	0.021	-0.020	0.062	0.156
	Lag 2	Lag 0	0.010	-0.025	0.045	0.290
		Lag 1	0.013	-0.011	0.037	0.145
		Lag 2	-0.006	-0.036	0.024	0.353
		Lag 3	0.008	-0.023	0.039	0.308
	Lag 3	Lag 0	0.022	-0.015	0.060	0.121
		Lag 1	0.016	-0.010	0.041	0.116
		Lag 2	0.014	-0.009	0.038	0.114
		Lag 3	0.021	-0.006	0.048	0.061
Renal	Lag 0	Lag 0	0.027	-0.020	0.074	0.130
		Lag 1	0.017	-0.036	0.070	0.267
		Lag 2	0.023	-0.040	0.087	0.235
		Lag 3	0.024	-0.043	0.091	0.245
	Lag 1	Lag 0	0.031	-0.014	0.076	0.089
		Lag 1	-0.009	-0.066	0.049	0.385
		Lag 2	-0.037	-0.111	0.037	0.163
		Lag 3	0.041	-0.022	0.104	0.100
	Lag 2	Lag 0	-0.013	-0.073	0.046	0.332
		Lag 1	0.003	-0.055	0.060	0.462
		Lag 2	-0.013	-0.072	0.047	0.339
		Lag 3	0.032	-0.017	0.082	0.102
	Lag 3	Lag 0	-0.048	-0.129	0.033	0.122
		Lag 1	0.001	-0.072	0.073	0.494
		Lag 2	-0.008	-0.062	0.045	0.380
		Lag 3	0.008	-0.040	0.055	0.378
Respiratory	Lag 0	Lag 0	-0.007	-0.061	0.047	0.403
		Lag 1	0.038	-0.010	0.086	0.060
		Lag 2	0.001	-0.059	0.061	0.483
		Lag 3	0.055	0.004	0.107	**0.017**
	Lag 1	Lag 0	-0.029	-0.090	0.032	0.174
		Lag 1	0.034	-0.015	0.082	0.086
		Lag 2	-0.013	-0.066	0.039	0.309
		Lag 3	0.049	-0.008	0.106	**0.047**
	Lag 2	Lag 0	0.043	-0.003	0.088	**0.032**
		Lag 1	0.036	0.001	0.072	**0.022**
		Lag 2	-0.006	-0.057	0.044	0.402
		Lag 3	0.032	-0.024	0.088	0.129
	Lag 3	Lag 0	0.011	-0.047	0.068	0.359

	Lag 1	0.021	-0.010	0.053	0.093
	Lag 2	-0.008	-0.057	0.042	0.382
	Lag 3	0.028	-0.022	0.078	0.137
